# Bioinspired Asymmetric Total Synthesis of Emeriones A–C**

**DOI:** 10.1002/anie.202205878

**Published:** 2022-06-28

**Authors:** Sven Jänner, Daniel Isak, Yuli Li, Kendall N. Houk, Aubry K. Miller

**Affiliations:** ^1^ Cancer Drug Development Group German Cancer Research Center (DKFZ) Heidelberg Germany; ^2^ Department of Chemistry School of Science Tianjin University Tianjin China; ^3^ Department of Chemistry and Biochemistry University of California Los Angeles, CA USA

**Keywords:** Biomimetic Synthesis, Cascade Reactions, Electrocyclizations, Polyketides, Total Synthesis

## Abstract

We report asymmetric bioinspired total syntheses of the fungal metabolites emeriones A–C via stereoselective oxidations of two bicyclo[4.2.0]octadiene diastereomers. The central bicyclic scaffolds are prepared in an 8π/6π electrocyclization cascade of a stereodefined pentaene, which contains the fully assembled side chains of the emeriones. The anti‐aldol side chain is made using a Paterson‐aldol addition, and the epoxide of the dioxabicyclo[3.1.0]hexane side chain via ring‐closure onto an oxidized acetal. Our work has enabled the structural revision of emerione C, and resulted in the synthesis of a “missing” family member, which we call emerione D. DFT calculations identified two methyl groups that govern torquoselectivity in the 8π/6π cascade.

## Graphical abstract

The emeriones, three highly oxidized and densely functionalized polyketides, were synthesized from two enantioenriched fragments in a Stille‐8π/6π sequence, followed by late‐stage stereo‐ and chemoselective oxidations. This work enabled the structural revision of emerione C.

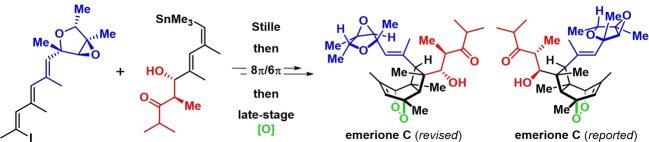

Natural products derived from polyenes that undergo cyclization/isomerization cascades initiated by an 8π electrocyclization have intrigued chemists for decades.^[1]^ The emeriones (Figure 1), one such family of natural products that were isolated from the fungus *E. nidulans*,^[2]^ display oxidized bicyclo[4.2.0]octadiene cores (red) flanked by a seven carbon aldol fragment (blue) and a propenyl‐substituted dioxabicyclo[3.1.0]hexane system (black). The two side chains (blue and black) of emerione A (**1**) and B (**2**) share the same absolute configurations, while the bicyclo[4.2.0]octadieneoxide central scaffolds are enantiomeric with respect to each other. Emerione C has a bridging endoperoxide on the central core, and its proposed structure has a stereochemical configuration similar to emerione B.

**Figure 1:**
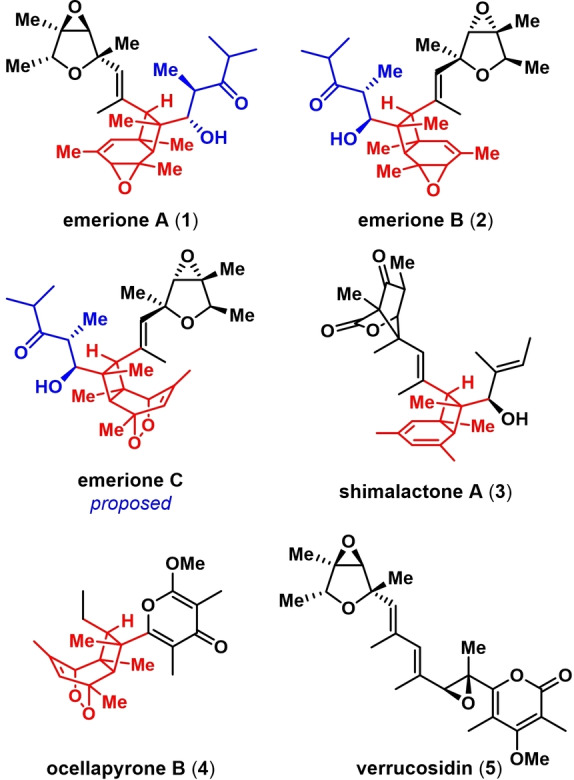
Structures of the emeriones and related natural products.

Related substances like shimalactone A (**3**)^[1p]^ and ocellapyrone B (**4**)[^1m^, ^1n^] have been synthesized, but the emeriones are arguably the most complex examples of such natural products, each containing twelve stereocenters, eight of which are contiguous, and two quaternary. Moreover, the dioxabicyclo[3.1.0]hexane system, also found in natural products like verrucosidin (**5**),^[3]^ is a considerable synthetic challenge alongside the oxidized bicyclo[4.2.0]octadiene scaffolds. Emerione A inhibits NO production in lipopolysaccharide‐induced RAW264.7 cells^[2]^ as well as NDM‐1^[4]^ at low micromolar concentrations, but the emeriones appear not to have been tested in other assays. Motivated both by their striking structures and potentially undiscovered bioactivities, we chose to target the emeriones for synthesis. We describe herein the successful completion of the syntheses, the structural revision of emerione C, and the synthesis of the originally proposed structure of emerione C, which we name emerione D.

It is plausible that the emeriones are biosynthetically derived from the unsaturated polyketide **6**, which after two oxidations gives a diastereomer (**7**) of the natural product emecorrugatin B (**8**) (Figure 2, top).^[5][6]^ Two double‐bond isomerizations then generate (*E,E,Z,Z,E*)‐pentaene **9**, which is poised to undergo an 8π/6π electrocyclization cascade.^[7]^ This provides bicyclo[4.2.0]octadienes **10** and **11**, which are oxidized to the emeriones. In our retrosynthesis (Figure 2, bottom), we modeled the late stages of our approach on the proposed biosynthesis. Therefore, emeriones A and B would be derived from **10** and **11**, respectively, via mono‐epoxidations, and emerione C would be traced back to **11** via [4+2] cycloaddition with ^1^O_2_. Intermediates **10** and **11** would arise from pentaene **9** through an 8π/6π electrocyclization cascade, which would form only two of the four Woodward–Hoffmann compatible stereoisomers. Pentaene **9** would be constructed convergently, in a Stille coupling of iodide **12** and stannane **13**. Stannane **13** could be derived from iodide **14**, which would be prepared in a Paterson *anti*‐aldol of aldehyde **16** and ketone **15**.^[8]^ Iodide **12** can be traced back to aldehyde **17** through a series of olefinations. The trisubstituted epoxide of **17** would be formed via oxidation of *para*‐methoxyphenyl acetal **18**, which would be derived from triol **19**. Sequential asymmetric oxidations would generate **19** from (*Z,Z*)‐dienol **20**.

**Figure 2:**
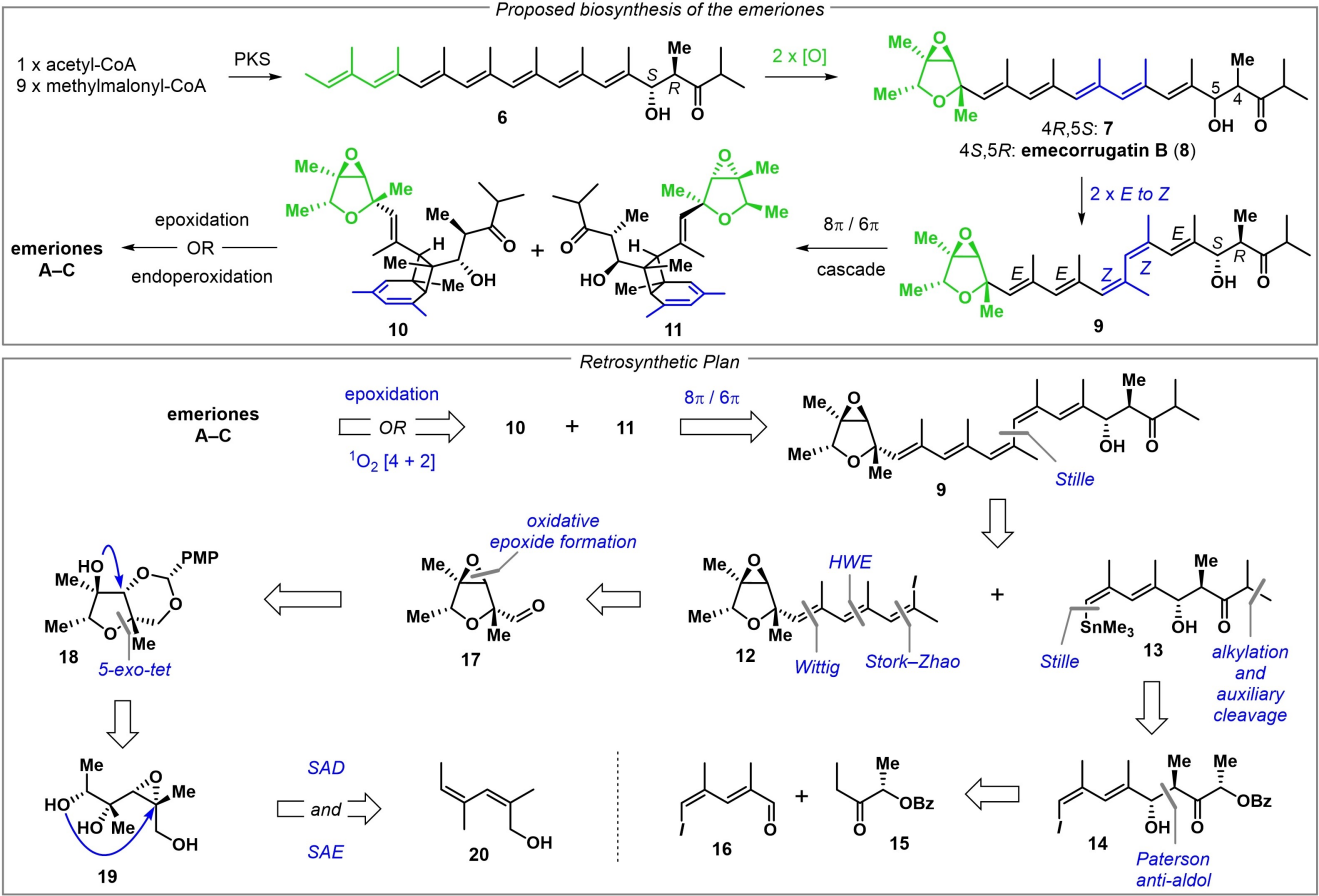
Proposed biosynthesis (top) and retrosynthetic plan (bottom).

Our synthesis began with iodide **22**, which can be prepared in four steps from propargyl alcohol (**21**) (Scheme 1A).^[1l]^ Aldehyde **23**, synthesized by MnO_2_ oxidation of **22**, is prone to isomerization/decomposition. It was therefore used immediately in a Paterson aldol with the *E*‐configured boron enolate of ketone **24** to give **25** in >95 : 5 diastereomeric ratio (dr). The relative and absolute configuration of **25** was confirmed via X‐ray crystallography. Silyl protection of the hydroxyl group gave **26**, followed by reductive removal of the chiral auxiliary.^[9]^ The resulting ethyl ketone (**27**) was converted to isopropyl ketone **28** via kinetic enolate formation and trapping with methyl iodide. ^[10]^ Removal of the silyl protecting group to give **29** could only be realized with HF⋅pyridine; other fluoride sources resulted in significant retro‐aldol reaction, and deprotection was sluggish under acidic conditions. Stille reaction of **29** with Me_6_Sn_2_ gave stannane **13**.

**Scheme 1:**
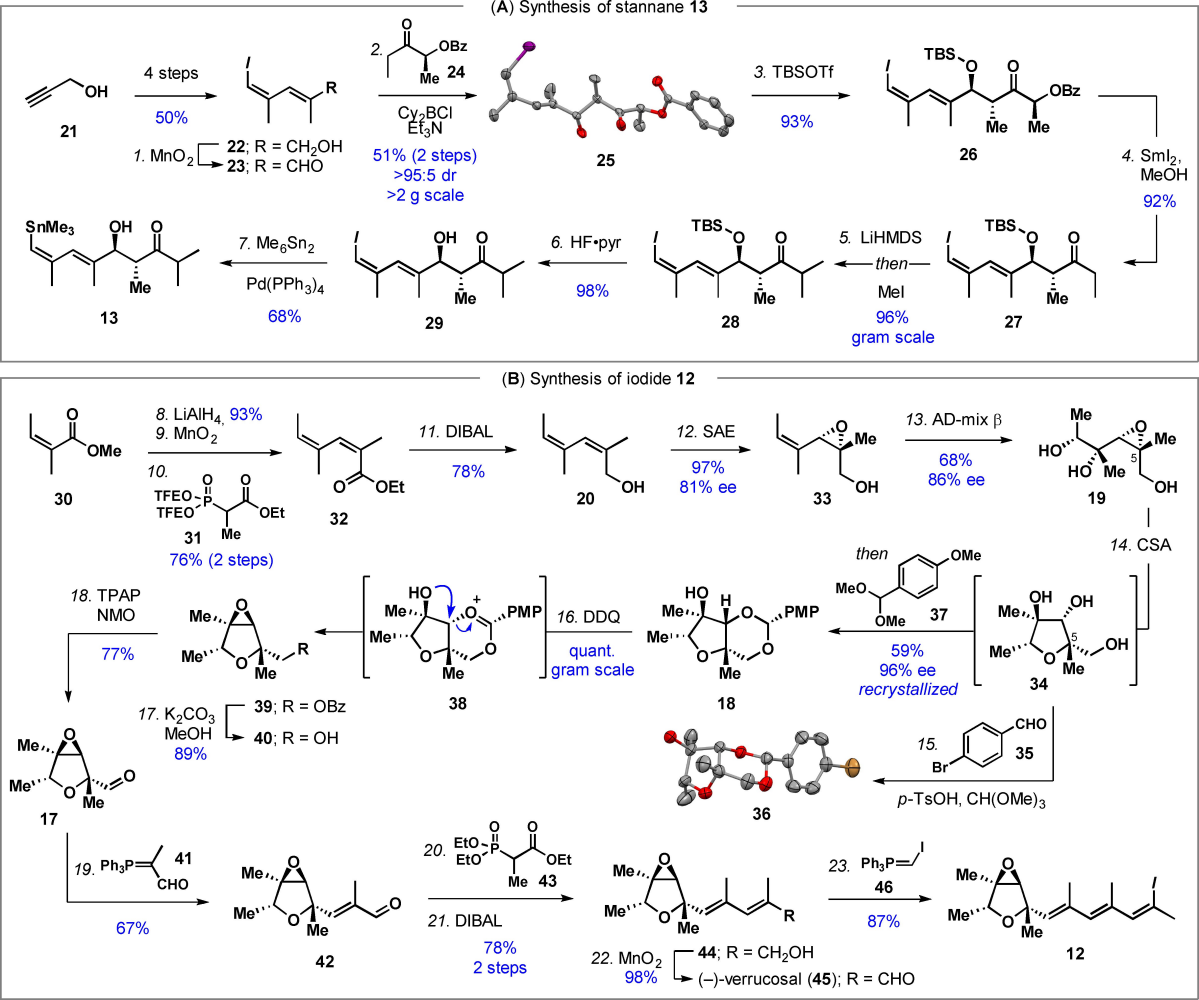
Synthesis of **12** and **13**. Reagents and conditions: *1*. MnO_2_ (21 equiv), CH_2_Cl_2_, rt, 30 min; *2*. Cy_2_BCl (1.8 equiv), Et_3_N (2.2 equiv), **24** (1.6 equiv), Et_2_O, −78 °C→0 °C then **23** (1.0 equiv), −78 °C→−20 °C, 51 % (2 steps); *3*. TBSOTf (3.1 equiv), 2,6‐lutidine (4.3 equiv), CH_2_Cl_2_ −78 °C, 4.5 h, 93 %; *4*. SmI_2_ (4.0 equiv), THF/MeOH, 0 °C, 1 h, 92 %; *5*. LiHMDS (2.0 equiv), THF, −78 °C, then MeI (3.0 equiv), 1.5 h, 96 %; *6*. HF⋅py/THF (1 : 4), 0 °C→rt, 18 h, 98 %; *7*. Pd(PPh_3_)_4_ (5 mol %), Sn_2_Me_6_ (1.2 equiv), THF, 80 °C, 5 h, 68 %; *8*. LiAlH_4_ (2.5 equiv), THF, 0 °C→rt, 2 h, 93 %; *9*. MnO_2_ (16.5 equiv), CH_2_Cl_2_, rt, 18 h; *10*. **31** (1.1 equiv), KHMDS (1.1 equiv), 18‐crown‐6 (3.0 equiv), THF, −78 °C, 1 h, *then* aldehyde (1.0 equiv), −78 °C, 1 h, 76 % (2 steps); *11*. DIBAL (2.7 equiv), CH_2_Cl_2_, 0 °C, 1 h, 78 %; *12*. Ti(O*i‐*Pr)_4_ (0.23 equiv), (−)‐DET (0.27 equiv), 4 Å MS, CH_2_Cl_2_, −25 °C, 0.5 h, *then* TBHP (2.2 equiv), −25 °C, 0.5 h, *then* **20** (1.0 equiv), −40 °C, 24 h, 97 %, 81 % ee; *13*. AD‐mix β (10 mass equiv), MeSO_2_NH_2_ (1.0 equiv), *t‐*BuOH/H_2_O (1 : 1), 0 °C, 18 h, 68 %, 86 % ee; *14*. CSA (0.1 equiv), CH_2_Cl_2_, 0 °C, 20 h, *then* **37** (1.5 equiv), 0 °C→rt, 4 h, 59 %, 96 % ee (recrystallized); *15*. **35** (1.0 equiv), *p*‐TsOH (0.2 equiv), HC(OMe)_3_ (1.1 equiv), THF; *16*. DDQ (1.3 equiv), 4 Å MS, DCE, 80 °C, 2 h, quant.; *17*. K_2_CO_3_ (6.0 equiv), MeOH, 0 °C→rt, 2 h, 89 %; *18*. TPAP (0.05 equiv), NMO (1.5 equiv), 4 Å MS, CH_2_Cl_2_, rt, 1.5 h, 77 %; *19*. **41** (1.04 equiv), THF, 100 °C (μ‐wave), 2 d, 67 %; *20*. **43** (1.2 equiv), LiO*t‐*Bu (1.2 equiv), THF, 0 °C→rt, 1 h, *then* **42** (1.0 equiv), THF, rt, 3 h, >95 : 5 dr; *21*. DIBAL (3.5 equiv), CH_2_Cl_2_, 0 °C, 3 h, 78 % (2 steps); *22*. MnO_2_ (25 equiv), CH_2_Cl_2_, rt, 2.5 h, 98 %; *23*. Ph_3_PEt^+^I^−^ (4.0 equiv), *n‐*BuLi, (4.0 equiv), THF, 0 °C→rt, 30 min, *then* I_2_ (4.0 equiv), THF, −78 °C, 10 min, *then* NaHMDS (3.8 equiv), THF, −78 °C, 10 min, *then* **45** (1.0 equiv), THF, −78 °C, 2 h, 87 %, >95 : 5 dr. Ellipsoids of **25** and **36** are depicted at a 50 % probability level.^[14]^ Color code: C, grey; O, red; I, purple, Br, gold.

The synthesis of iodide **12** began with conversion of methyl angelate (**30**) into angelic aldehyde, which was found to be configurationally labile (Scheme 1B).^[11]^ Therefore, angelic aldehyde was immediately used in a Still–Gennari olefination with **31** to give dienoate **32**, which was then reduced to give allylic alcohol **20**.[^12^, ^13^] Sharpless asymmetric epoxidation of **20** proceeded in excellent yield to give **33**, but with a modest 81 % ee,^[15]^ as previously observed with *Z*‐configured allylic alcohols.^[16]^

While Upjohn oxidation of epoxide **33** to give triol **19** was moderately diastereoselective (72 : 28 dr), Sharpless asymmetric dihydroxylation (SAD) proceeded with an improved dr of 86 : 14. Moreover, due to reagent control in the SAD reaction, **19** was isolated with 86 % ee (Scheme S1).^[17]^ Acid‐catalyzed isomerization of triol **19** proceeded with inversion of stereochemistry at C5 to give tetrahydrofuran **34**, which contains the appropriate vicinal *anti*‐diol configuration for epoxide formation.^[18]^ After numerous attempts to advance **34** to aldehyde **17** (Scheme S2), we hypothesized that the epoxide in **17** could be formed via oxidation of an acetal like **18**.^[19]^ Acetal formation was facile: treatment of triol **34** with aldehyde **35** under acidic conditions gave **36**, whose absolute and relative configuration was confirmed via X‐ray crystallography. Noting that the preceding reaction is acid‐catalyzed, we developed a one‐pot procedure from triol **19** to acetal **18**. In the event, after completion of the CSA‐catalyzed isomerization of **19** to **34**, addition of acetal **37** produced **18**, which could be crystallized to 96 % ee.

Pleasingly, oxidation of **18** with DDQ produced epoxide **39**, presumably through the intermediacy of oxonium **38**. To the best of our knowledge, this is the first synthesis of an epoxide from a 1,2‐diol using this approach.[^19^, ^20^] Methanolysis of **39** gave alcohol **40**, which oxidized to aldehyde **17** under Ley–Griffith conditions (TPAP/NMO). Wittig homologation of **17** produced aldehyde **42**, which underwent Horner–Wadsworth–Emmons olefination and reduction to obtain alcohol **44**. Manganese dioxide oxidation gave verrucosal (**45**),^[21]^ which was olefinated using Stork–Zhao conditions to produce iodide **12**.

To complete the synthesis of the emeriones, **12** and **13** were combined in a Stille coupling to give pentaene **9** (Scheme 2). Stille conditions using Pd_2_dba_3_/P(2‐furyl)_3_/CuI or the Liebeskind variant (CuTC/Pd(PPh_3_)_4_) both successfully delivered product. Interestingly, **9** could be purified via chromatography and fully characterized with no apparent isomerization or decomposition. Upon heating in toluene at 55 °C, **9** slowly (3 d) and cleanly isomerized into a roughly equimolar mixture of **10** and **11**, as estimated by ^1^H‐NMR.^[22]^ This outcome must arise via conrotatory 8π electrocyclization of **9** proceeding with essentially no induced diastereocontrol to produce cyclooctatrienes **47** and **48**. These diastereomers then each undergo highly torquoselective 6π disrotatory electrocyclization to **10** and **11**, respectively. Pleasingly, **10** and **11** were chemo‐ and stereoselectively epoxidized with *m*‐CPBA at the least hindered of their three double bonds to give (−)‐emerione A (**1**) and (−)‐emerione B (**2**), respectively.^[23]^ Spectroscopic and optical rotation data were consistent with the values reported by the isolationists (Tables S1, S2).

**Scheme 2:**
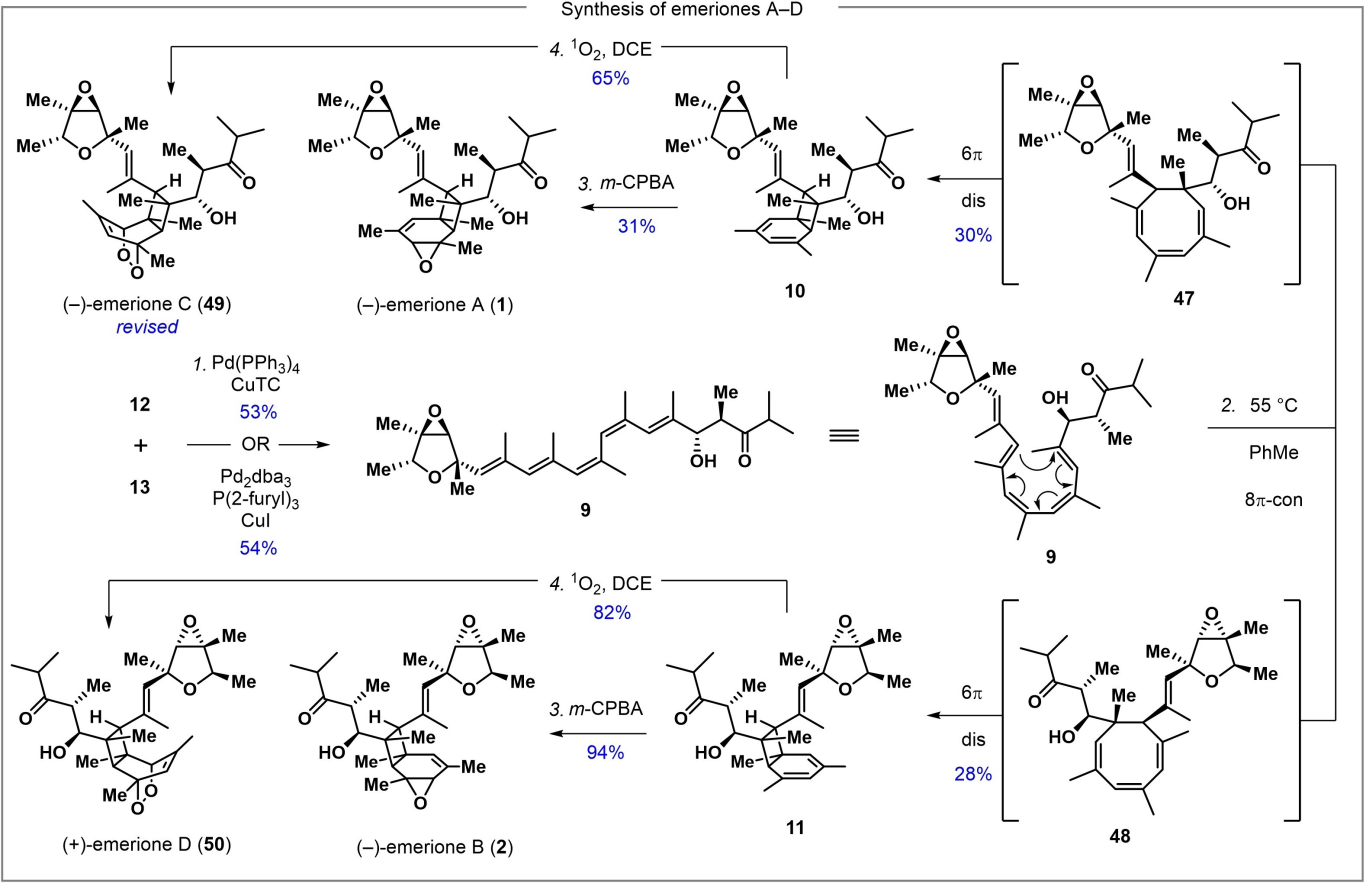
Completion of the synthesis of the emeriones. Reagents and conditions: *1*. **12** (1.0 equiv), **13** (1.5 equiv), Pd_2_(dba)_3_ (0.12 equiv), P(2‐furyl)_3_ (0.48 equiv), CuI (2.1 equiv), NMP, rt, 20 h, 54 % OR **12** (1.0 equiv), **13** (1.5 equiv), Pd(PPh_3_)_4_ (0.10 equiv), CuTC (1.1 equiv), DMF, rt, 1 h, 53 %; *2*. PhMe, 55 °C, 3 d, **10**: 30 %, **11**: 28 %; *3. m*‐CPBA (1.0 equiv), NaHCO_3_ (22 equiv), CH_2_Cl_2_/H_2_O (2 : 1), 0 °C→rt, 45 min, **1**: 31 %, **2**: 94 %; *4*. O_2_, methylene blue (0.03 equiv), hν, DCE, 10 min, **49**: 65 %, **50**: 82 %.

When an O_2_‐saturated dichloroethane solution of **11** with triplet sensitizer was irradiated (400 W, white halogen lamp), a single endoperoxide adduct (**50**) was formed. We expected **50** to be (−)‐emerione C; however, comparison of NMR spectra of **50** and literature data for emerione C (Figure 3A, Table S4) made clear that the two substances are different.^[2]^ We therefore treated **10** under identical ^1^O_2_‐producing conditions to cleanly give endoperoxide **49**. This compound had NMR spectra identical to those reported for emerione C (Figure 3B, Table S3). To unambiguously clarify the chemical structures, we solved the structure of **50** by X‐ray crystallography (Figure 3C), and found that it has the originally proposed structure of emerione C. We, therefore, reassign the structure of emerione C (**49**) as it is depicted in Scheme 2 and name compound **50**, which may also be a natural product, (+)‐emerione D.^[24]^

**Figure 3:**
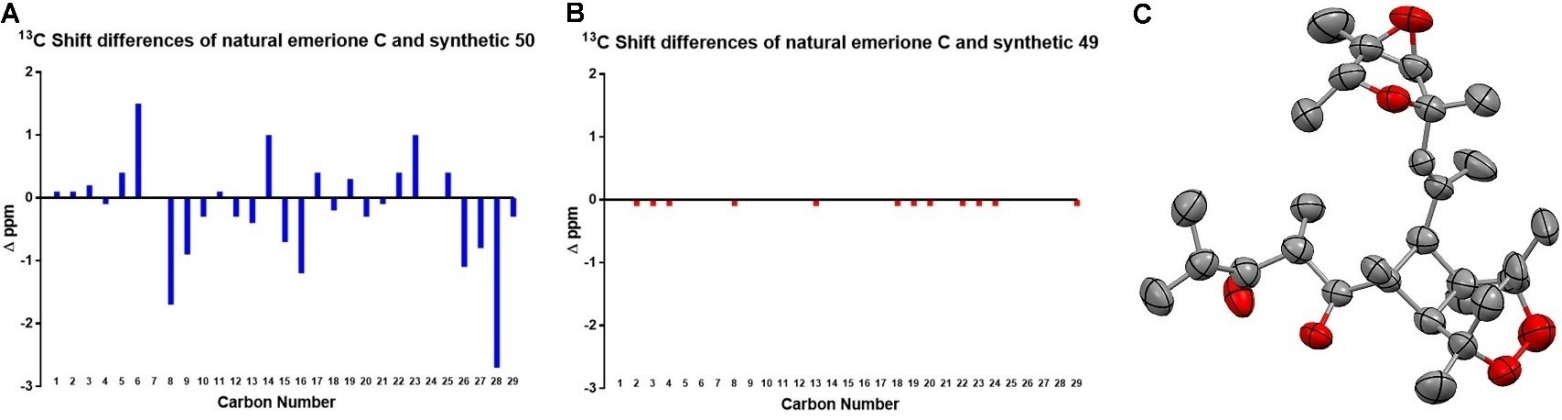
A) Comparison of emerione C and **50** ^13^C shifts. B) Comparison of emerione C and **49** ^13^C shifts. C) Experimental structure of emerione D (**50**). Ellipsoids depicted at a 50 % probability level.^[14]^ Color code: C, grey; O, red.

To gain insight into the stereochemical outcome of the electrocyclization cascade, we employed density functional theory (DFT) calculations at the SMD(toluene)‐M06‐2X/Def2‐TZVP//M06‐2X/Def2‐SVP level of theory. The calculations reveal that the two transition states (TS1 and TS2) leading from **9** to **47** and **48**, respectively, are nearly isoenergetic as are **47** and **48** (Figure 4). Therefore, the rates of formation and thermodynamic stabilities of **47** and **48** are nearly equal, consistent with experimental observations. The subsequent 6π electrocyclizations of **47** to **10** and **48** to **11**, were found to proceed via TS3 and TS4, respectively. These are 1.9 kcal mol^−1^ and 1.8 kcal mol^−1^ lower in Gibbs free energy than the diastereomeric transition states TS3′ and TS4′, respectively (Figure S4). The two methyl groups in **47** and **48**, which end up on the cyclobutane rings of **10** and **11**, were found to have opposing influences on the torquoselectivity of the 6π electrocyclization. Replacing the bridgehead (green) methyl (Figure 4) with a proton results in a reversal of both 6π‐electrocyclization torquoselectivities (Figure S5), indicating that the purple methyl prefers to reside on the convex face of the bicyclo[4.2.0]octadiene. This is consistent with previous calculations.^[7]^ Replacing the purple methyl with a proton had little effect on the torquoselectivity (Figure S6), suggesting a strong and dominant steric penalty when the bridgehead methyl is *syn* to the vinyl dioxabicyclo[3.1.0]hexane system. Removing both methyl groups resulted in an almost complete loss of diastereoselectivity (Figure S7).

**Figure 4:**
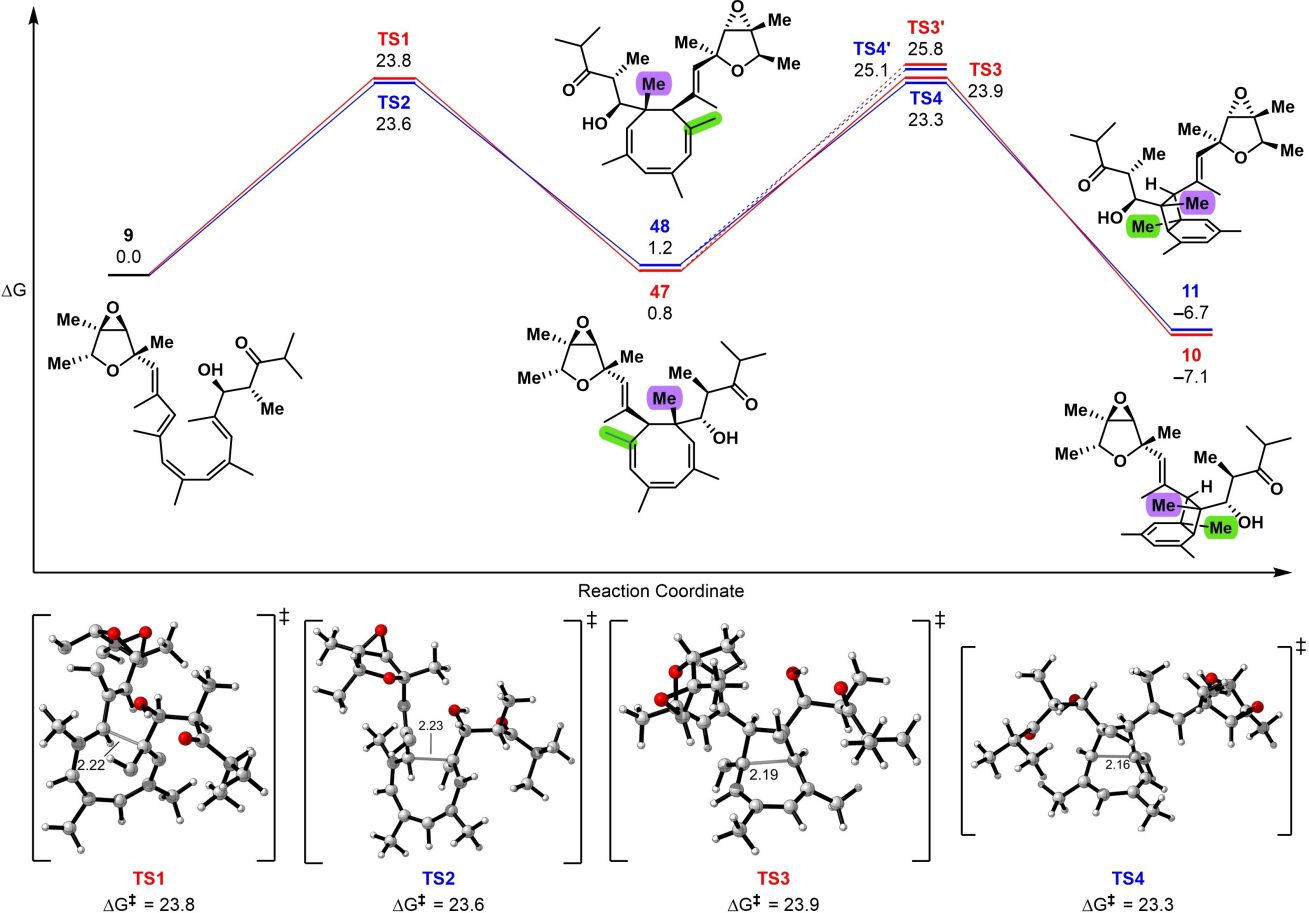
Reaction coordinate diagram and DFT‐calculated transition states of the electrocyclization cascade. The purple and green methyl groups have opposing and unequal effects on the torquoselectivity of the 6π electrocyclization (see Supporting Information).

In conclusion, we have completed an asymmetric bioinspired synthesis of all three emeriones, each with a longest linear sequence of 17 steps. Our synthesis has resulted in the reassignment of the structure of emerione C and the proposal of an additional family member, emerione D. As biological data of the emeriones is limited, current efforts in our lab aim to discover biological activities of these fascinating substances.

## Conflict of interest

The authors declare no conflict of interest.

## Supporting information

As a service to our authors and readers, this journal provides supporting information supplied by the authors. Such materials are peer reviewed and may be re‐organized for online delivery, but are not copy‐edited or typeset. Technical support issues arising from supporting information (other than missing files) should be addressed to the authors.

Supporting Information

Supporting Information

## Data Availability

The data that support the findings of this study are available in the Supporting Information of this article.
