# Supplementary material for: Bioinspired Asymmetric Total Synthesis of Emeriones A–C
Source: Angew Chem Int Ed Engl. 2022 Jun 28;61(32):e202205878. doi: 10.1002/anie.202205878 (PMC9401028; doi:10.1002/anie.202205878)
Supplement: Supplementary file 1 — Supporting Information [file ANIE-61-0-s001.pdf]

Angewandte Chemie
Eine Zeitschrift der Gesellschaft Deutscher Chemiker

Supporting Information

Bioinspired Asymmetric Total Synthesis of Emeriones A–C

*S. Jänner, D. Isak, Y. Li, K. N. Houk, A. K. Miller**

Table of Contents

1 Materials and Methods 3

2 Preparative Procedures and Analytical Data 5

2.1 Synthesis of Stannane **13** 5

2.2 Synthesis of Iodide **12** 16

2.3 Cross-coupling and 8π/6π electrocyclization cascade 37

2.4 Synthesis of Emerione A (**1**) 42

2.5 Table S1: Comparison of synthetic and natural NMR data of emerione A (**1**) 43

2.6 Synthesis of Emerione B (**2**) 44

2.7 Table S2: Comparison of synthetic and natural NMR data of emerione B (**2**) 45

2.8 Synthesis of Emerione C (**49**) 46

2.9 Table S3: Comparison of NMR data for synthetic 49 and natural emerione C 47

2.10 Synthesis of Emerione D (**50**) 48

2.11 Table S4: Comparison of NMR data for synthetic 50 and natural emerione C (**49**) 50

2.12 X-ray Crystallographic Reports 51

2.12.1 Crystallographic Analysis of **25** 51

2.12.2 Table S5: Crystal data and structural refinement for **25** 51

2.12.3 Crystallographic Analysis of **36** 52

2.12.4 Table S6: Crystal data and structural refinement for **36** 53

2.12.5 Crystallographic Analysis of 50 54

2.12.6 Table S7: Crystal data and structural refinement for **50** 55

3 DFT calculations 55

3.1 Methods 55

3.2 Figure S4: Calculated energy levels of the electrocylization cascade 57

3.3 Figure S5: DFT-calculations after replacing the green methyl with a hydrogen 58

3.4 Figure S6: DFT-calculations after replacing the purple methyl with a hydrogen.......... 59

3.5 Figure S7: DFT-calculations after replacing both methyls with hydrogens 60

4 XYZ coordinates of optimized DFT structures and corresponding energies 60

5 Scheme S1: Stereochemical implications for the synthesis of triol **19**................................ 93

6 Scheme S2: Synthetic approaches toward the dioxabicyclo[3.1.0]hexane fragment........... 93

7 References 95

8 NMR Spectra 97

1 Materials and Methods

Unless otherwise stated, reactions were performed under an atmosphere of argon with anhydrous solvents utilizing standard Schlenk techniques. Glassware was oven-dried or dried using a heat gun under high vacuum. Tetrahydrofuran (THF), methylene chloride (CH_2Cl_2), and toluene (PhMe) were dried with an MBraun solvent purification system (Model: SPS 800). All other anhydrous solvents were purchased and used as is unless otherwise noted. All chemicals and reagents were purchased at the highest level of purity and used as received. Reactions were monitored by thin layer chromatography using EMD/Merck silica gel 60 F254 pre-coated plates (0.25 mm) and were visualized by UV or charring plates stained with solutions of *p*-anisaldehyde, $KMnO_4$, or CAM. A Biotage Initiator+ was used for reactions heated with microwave irradiation. Flash column chromatography was performed using silica gel (SiliaFlash® P60, particle size 40-63 microns [230 to 400 mesh]) purchased from Silicycle or with an automated RediSep Rf system (Teledyne Isco) and RediSep Rf columns (Teledyne Isco). NMR spectra were recorded on Bruker Avance 14.1 T and Avance III 9.4 T (German Cancer Research Center) or Bruker Avance III 14.1 T (Department of Organic Chemistry, Heidelberg University) NMR spectrometers operating at 600 or 400 MHz for ^1H nuclei and 151 and 101 MHz for ^{13}C nuclei, NMR data are reported relative to residual solvent peaks ($CHCl_3$; δ = 7.26 or 77.23 ppm and DMSO-d_5; δ = 2.50 or 39.5 ppm). Multiplicities and qualifiers are abbreviated as: s = singlet, d = doublet, t = triplet, q = quartet, p = pentet, m = multiplet, br = broad, app = apparent. High resolution mass spectrometry was conducted on a Bruker ApexQe instrument using electrospray (ESI), a Bruker timsTOFfleX instrument using atmospheric pressure chemical ionization (APCI), or a JEOL AccuTOF GCx instrument using electron impact (EI).

Optical rotations were measured on a Perkin Elmer 341 polarimeter. Analytical LC-MS was carried out on an Agilent 1260 Infinity system using a Kinetex 2.6 μm C18, 100 Å (50 × 2.1 mm) column and an ELSD 1260 Infinity; Temperature: 40 °C; Solvent A = water + 0.01% formic acid; Solvent B = acetonitrile + 0.01% formic acid; Flow rate = 0.60 mL/min; Method: 1% B → 90% B over 6 minutes, 90% B → 99% B over 2 minutes. Preparative HPLC was performed on an Agilent 1260 Infinity system using a Gemini® 5 μm C18, 110 Å (250 × 21.2 mm) column.

2 Preparative Procedures and Analytical Data

2.1 Synthesis of Stannane 13

Synthesis of (*Z*)-3-iodo-2-methylprop-2-en-1-ol (S1):[1]

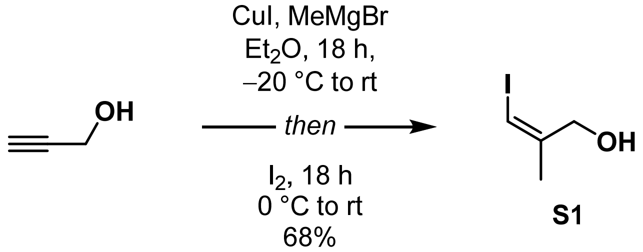

To a suspension of copper(I) iodide (1.14 g, 6.00 mmol, 0.1 eq) and propargyl alcohol (3.45 mL, 59.81 mmol, 1.0 eq) in anhydrous Et_2O (150 mL) was added a 3 M solution of methylmagnesium bromide in Et_2O (60 mL, 180 mmol, 3.0 eq) over 2 h at –20 °C using an addition funnel. After addition, the reaction mixture was allowed to warm to rt. After stirring for 18 h, the reaction was cooled to 0 °C and a solution of iodine (30.4 g, 120 mmol, 2.0 eq) in anhydrous Et_2O (150 mL) was added via cannula over 15 min. The resulting brown suspension was allowed to warm to rt. After 18 h, the reaction was quenched with a saturated solution of NH_4Cl (150 mL). The layers were separated and the aqueous phase was extracted with Et_2O (3 x 150 mL). The combined organic extracts were washed with a 10% solution of $Na_2S_2O_3$ (2 x 100 mL), brine (150 mL) and dried with $MgSO_4$. After filtering, the solvent was removed under reduced pressure and the product was purified by bulb-to-bulb Kugelrohr distillation (10 mbar, 150 °C). Allylic alcohol **S1** was obtained as a pale-yellow oil (8.06 g, 40.6 mmol, 68 %).

$\mathbf{R}_f$ = 0.47 (20% EtOAc in *n*-hexane)

^{1}H NMR (400 MHz, $CDCl_3$, 298 K): δ 5.98–5.96 (m, 1H), 4.23 (s, 2H), 1.97 (d, *J* = 1.5 Hz, 3H) ppm

^{1}H-NMR data was consistent with literature spectra.[1]

Synthesis of Ethyl (2*E*,4*Z*)-5-iodo-2,4-dimethylpenta-2,4-dienoate (S3):[2]

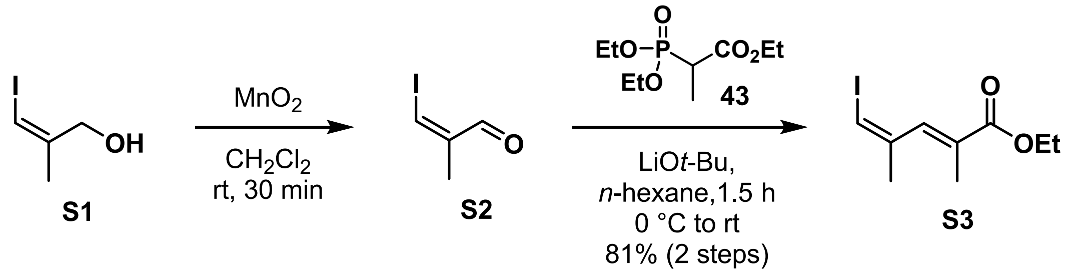

To a solution of **S1** (6.48 g, 32.7 mmol, 1.0 eq) in CH_2Cl_2 (350 mL) was added MnO_2 (70 g, 805 mmol, 24.6 eq) at rt. After stirring for 30 min the mixture was filtered over a short pad of Celite®, which was then washed with CH_2Cl_2 (500 mL). The solvent was removed under reduced pressure to yield aldehyde **S2** as a yellow, crystalline solid. The aldehyde was immediately used in the next reaction.

To a solution of triethyl-2-phosphonopropionate (**43**) in *n*-hexane (15 mL) was added a 1 M solution of lithium tert-butoxide (50 mL, 50 mmol, 1.5 eq) in THF via cannula at 0 °C. The cooling bath was removed and the mixture was stirred for 1 h. The freshly prepared aldehyde **S2** was dissolved in *n*-hexane (30 mL) and slowly added via syringe. The transfer was quantitated with 5 mL of *n*-hexane. After 30 min the reaction mixture was quenched with water (50 mL). The layers were separated and the organic phase was washed with water (80 mL), followed by brine (80 mL). The organic layer was dried with $MgSO_4$ and the solvent was removed under reduced pressure. Ester **S3** (dr >10:1) was obtained as an orange oil (7.39 g, 26.4 mmol, 81% for both steps). Separation of the alkene isomers was much easier after reduction of the ester to an alcohol and, therefore, delayed until the next synthetic step.

$\mathbf{R_f}$ = 0.47 (20% EtOAc in *n*-hexane)

^{1}H NMR (400 MHz, $CDCl_3$, 298 K): δ 7.09–7.07 (m, 1H), 6.24–6.23 (m, 1H), 4.23 (q, J = 7.1 Hz, 2H), 2.01 (dd, J = 1.4, 0.6 Hz, 3H), 1.90 (d, J = 1.4 Hz, 3H) ppm

^{1}H-NMR data was consistent with literature spectra.[2]

Synthesis of (2*E*,4*Z*)-5-iodo-2,4-dimethylpenta-2,4-dien-1-ol (**22**):[2]

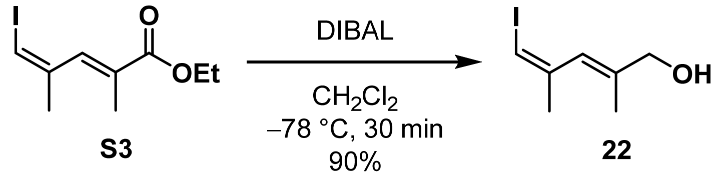

To a solution of **S3** (7.39 g, 26.4 mmol, 1.0 eq) in anhydrous CH_2Cl_2 (250 mL) was added a 1 M solution of diisobutylaluminium hydride (66 mL, 66 mmol, 2.5 eq) in toluene dropwise over 45 min at –78 °C using an addition funnel. After stirring for 30 min, the reaction mixture was warmed to 0 °C and quenched with a 20% solution of Rochelle's salts (400 mL). It was then further diluted with CH_2Cl_2 (300 mL) and transferred to an Erlenmeyer flask, where it was vigorously stirred for 30 min. The mixture was filtered over a pad of Celite®, which was then washed with CH_2Cl_2 (400 mL). The layers were separated and the aqueous phase was extracted with CH_2Cl_2 (2 x 300 mL). The combined organic extracts were dried with $MgSO_4$ and the solvent was removed under reduced pressure. The product was purified by flash chromatography (20 – 30% EtOAc in *n*-hexane) to provide alcohol **22** as a light-yellow oil (5.68 g, 23.9 mmol, 90%)

R_f = 0.38 (30% EtOAc in *n*-hexane)

^{1}H NMR (400 MHz, $CDCl_3$, 298 K): δ 6.05–6.03 (m, 1H), 5.95–5.93 (m, 1H), 4.09 (d, *J* = 4.7 Hz, 2H), 2.00–1.99 (m, 3H), 1.73–1.71 (m, 3H) ppm

^{1}H-NMR data was consistent with literature spectra.[2]

Synthesis of (*S*)-2-hydroxy-*N*-methoxy-*N*-methylpropanamide (**S4**):[3]

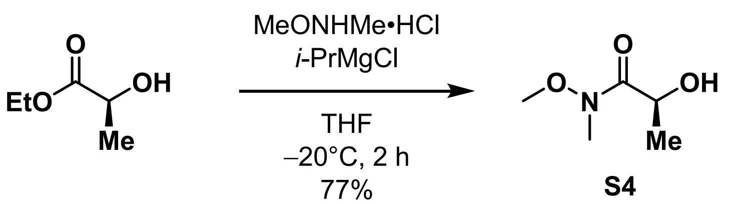

To a solution of (*S*)-ethyl lactate (5.0 g, 42.3 mmol, 1.0 eq) and *N,O*-dimethylhydroxylamine hydrochloride (10.3 g, 106 mmol, 2.5 eq) in anhydrous THF (200 mL) was added 2.0 M solution of

isopropylmagnesium chloride in Et_2O (105 mL, 210 mmol, 5.0 eq) slowly over 1 h using an additional funnel at −20 °C. After 1 h, the reaction mixture was warmed to 0 °C and was quenched with a saturated solution of NH_4Cl (150 mL). Excess salts were dissolved with water (25 mL) and the layers were separated. The aqueous phase was extracted with Et_2O (4 x 100 mL) and then CH_2Cl_2 (4 x 100 mL). The combined organic extracts were dried with $MgSO_4$ and the solvent was removed under reduced pressure. The product was purified by flash chromatography (60% EtOAc in *n*-hexane) to give Weinreb amide **S4** as a pale-yellow oil (4.34 g, 32.6 mmol, 77%).

R*f* = 0.41 (60% EtOAc in *n*-hexane)

^{1}H NMR (400 MHz, $CDCl_3$, 298 K): δ 4.41 (m, 1H), 3.64 (s, 3H), 3.42 (d, *J* = 7.8 Hz, 1H), 3.17 – 3.15 (s, 3H), 1.28 (d, *J* = 6.6 Hz) ppm

^{1}H-NMR data was consistent with literature spectra.[3]

Synthesis of (*S*)-3-oxopentan-2-yl benzoate (24):[3]

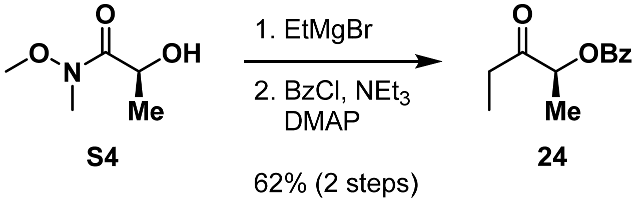

The following procedure slightly differs from the Paterson protocol[3] in that we did not purify the intermediate hydroxyketone. To a solution of **S4** (4.30 g, 32.3 mmol, 1.0 eq) in anhydrous THF (200 mL) was added a 3 M solution of ethylmagnesium bromide (35.5 mL, 107 mmol, 3.3 eq) in Et_2O dropwise at 0 °C under argon. The mixture was allowed to warm to rt and, after 2 h was quenched with a saturated solution of NH_4Cl (100 mL). The layers were separated and the aqueous phase was extracted with CH_2Cl_2 (3 x 100 mL). The combined organic extracts were dried with $MgSO_4$ and the solvent was removed under reduced pressure. The residue was re-dissolved in anhydrous CH_2Cl_2 (150 mL) and benzoyl chloride (5.3 mL, 45.2 mmol, 1.4 eq), triethylamine (6.8 mL, 48.4 mmol, 1.5 eq), and 4-(dimethylamino)pyridine (395 mg, 3.23, 0.1 eq) were added. After stirring for 2.5 d at rt, ethylenediamine (1.3 mL, 19.4 mmol, 0.6 eq) was added to quench excess benzoyl chloride. The mixture was filtered through paper and washed with brine (100 mL). The

aqueous phase was back extracted with EtOAc (3 x 100mL). The combined organic extracts were dried with $MgSO_4$ and the solvent was removed under reduced pressure. The product was purified by flash chromatography (10% EtOAc in *n*-hexane) to give ketone **24** as a colorless oil (4.10 g, 19.9 mmol, 62% for both steps).

R_f = 0.57 (30% EtOAc in *n*-hexane)

^{1}H NMR (400 MHz, $CDCl_3$, 298 K): δ 8.10–8.07 (m, 2H), 7.61–7.59 (m, 1H), 7.49–7.43 (m, 2H), 5.36 (q, J = 7.0 Hz, 1H), 2.65 (dq, J = 18.3, 7.3 Hz, 1H), 2.53 (dq, J = 18.3, 7.3 Hz, 1H), 1.53 (d, J = 7.0 Hz, 3H), 1.10 (t, J = 7.3 Hz, 3H) ppm

^{1}H-NMR data was consistent with literature spectra.[3]

Synthesis of (2*E*,4*Z*)-5-iodo-2,4-dimethylpenta-2,4-dienal (**23**):

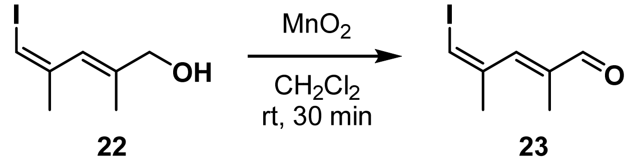

To a solution of **22** (2.50 g, 10.5 mmol, 1.0 eq) in CH_2Cl_2 (125 mL) was added MnO_2 (19 g, 219 mmol, 21 eq) at rt. After stirring for 30 min the mixture was filtered over a short pad of Celite®, which was then washed with CH_2Cl_2 (500 mL). The solvent was removed under reduced pressure, to yield aldehyde **23** as an orange oil, which was used immediately in the next reaction.

Synthesis of (2*S*,4*R*,5*S*,6*E*,8*Z*)-5-hydroxy-9-iodo-4,6,8-trimethyl-3-oxonona-6,8-dien-2-yl benzoate (25):

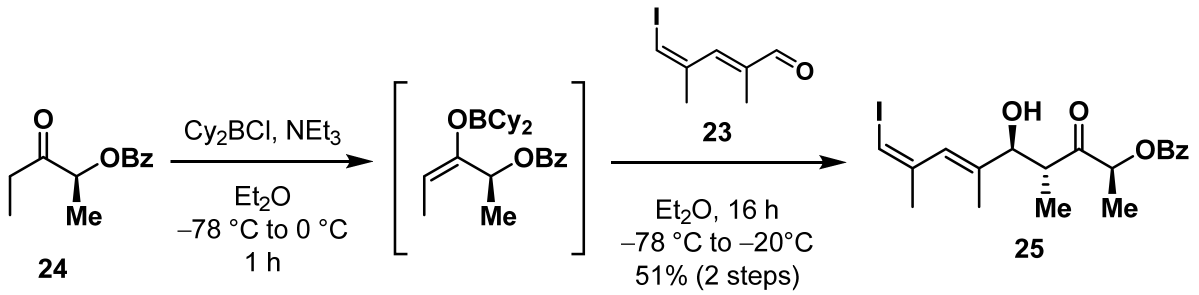

In parallel to the above reaction, triethylamine (3.20 mL, 23.0, 2.2 eq) was added to a solution of chlorodicyclohexylborane (19 mL, 19.0 mmol, 1.8 eq, 1 M in hexanes) in anhydrous Et_2O (45 mL) dropwise at –78 °C. After 5 min, a solution of ketone **24** (3.47 g, 16.8 mmol, 1.6 eq) in Et_2O (7.5 mL) was added dropwise via cannula over 5 min. The transfer was quantitated by rinsing with Et_2O (2 x 4 mL). After the addition was complete the reaction mixture was warmed to 0 °C and stirred for 1 h, at which time the mixture was cooled to −78 °C and a solution of the freshly prepared aldehyde **23** (assumed 10.5 mmol, 1.0 eq) in Et_2O (7.5 mL) was added via cannula. The transfer was quantitated with Et_2O (2 x 4 mL). After stirring for 3 h at −78 °C the reaction mixture was stored in the freezer at −20 °C for 16 h. It was then warmed to 0 °C and quenched with MeOH (20 mL), a 30% H_2O_2 solution (10 mL) and phosphate buffer (pH 7, 100 mL). The layers were separated and the aqueous phase was extracted with Et_2O (4 x 75 mL). The product (dr ~10:1) was purified by flash chromatography (10 to 20% EtOAc in *n*-hexane). It was then further purified to high diastereomeric purity by recrystallization from boiling *n*-hexane and EtOAc to give aldol **25** as colorless needles (2.36 g, 5.34 mmol, 51% for 2 steps).

$\mathbf{R}_f$ = 0.48 (30% EtOAc in *n*-hexane)

$[\alpha]^{20}_{578}$ = +12.5° (*c* = 0.69 in MeOH)

^{1}H NMR (400 MHz, $CDCl_3$, 298 K): δ 8.11–8.07 (m, 2H), 7.61–7.55 (m, 1H), 7.49–7.43 (m, 2H), 6.10–6.05 (m, 1H), 5.89 (s, 1H), 5.48 (q, *J* = 7.0 Hz, 1H), 4.31 (dd, *J* = 9.2, 2.8 Hz, 1H), 3.12–3.05 (m, 1H), 2.17 (d, *J* = 3.5 Hz, 1H), 1.97 (d, *J* = 0.8 Hz, 3H), 1.68 (d, *J* = 1.2 Hz, 3H), 1.17 (d, *J* = 7.1 Hz, 3H) ppm

^{13}C NMR (101 MHz, $CDCl_3$, 298 K): δ 211.0. 166.0, 144.3, 138.5, 133.4, 130.8, 129.9, 129.7, 128.6, 79.8, 77.8, 75.2, 45.8, 24.7, 15.8, 14.8, 12.6 ppm

HRMS (ESI) *m/z*: $[M+Na]^+$ calcd for $C_{19}H_{23}INaO_4^+$: 465.0534; found: 465.0533

Synthesis of (2*S*,4*R*,5*S*,6*E*,8*Z*)-5-((tert-butyldimethylsilyl)oxy)-9-iodo-4,6,8-trimethyl-3-oxonona-6,8-dien-2-yl benzoate (26):

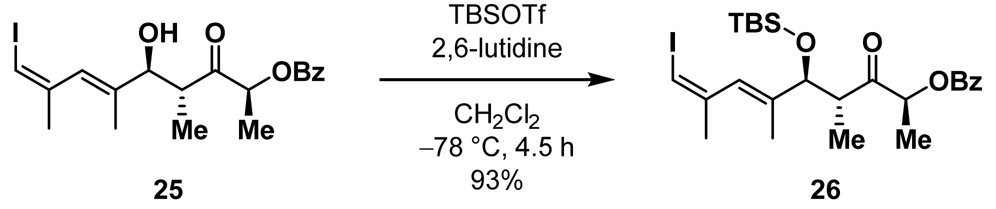

To a solution of **25** (2.15 g, 4.86 mmol, 1 eq) in anhydrous CH_2Cl_2 (500 mL) was added 2,6-lutidine (2.4 mL, 20.7 mmol, 4.3 eq) followed by *tert*-butyldimethylsilyl trifluoromethanesulfonate (3.5 mL, 15.2 mmol, 3.1 eq) at −78 °C. After stirring for 4.5 h at −78 °C, the solution was quenched with MeOH (20 mL). It was then warmed to 0 °C and a sat. solution of NH_4Cl (100 mL) was added. The layers were separated and the aqueous phase was extracted with CH_2Cl_2 (3 x 50 mL). The combined organic extracts were dried with $MgSO_4$ and the solvent was removed under reduced pressure. The product was purified by flash chromatography (5% EtOAc in *n*-hexane) to give silyl ether **26** (2.52 g, 4.53 mmol, 93%) as a pale-yellow oil.

$\mathbf{R_f}$ = 0.49 (10% EtOAc in *n*-hexane)

$[\alpha]^{20}_{578}$ = +31.2 (*c* = 1.0 in MeOH)

^{1}H NMR (400 MHz, $CDCl_3$, 298 K): δ 8.10–8.06 (m, 2H), 7.60–7.56 (m, 1H), 7.48–7.43 (m, 2H), 6.08–6.06 (m, 1H), 5.85 (s, 1H), 5.45 (q, *J* = 7.0 Hz, 1H), 4.34 (d, *J* = 9.7 Hz, 1H), 3.04 (dq, *J* = 7.1 Hz, *J* = 9.7 Hz, 1H), 1.97 (d, *J* = 1.1 Hz, 3H), 1.65 (d, *J* = 1.2 Hz, 3H), 1.55 (d, *J* = 7.0 Hz, 3H), 0.83 (s, 9H), 0.03 (s, 3H), 0.02 (s, 3H) ppm

^{13}C NMR (101 MHz, $CDCl_3$, 298 K): δ 209.3, 165.9, 144.2, 138.2, 133.4, 130.7, 130.0, 129.9, 128.6, 80.9, 77.7, 75.4, 46.6, 26.0, 24.6, 18.2, 15.4, 14.9, 12.4, −4.4, −4.9 ppm

HRMS (ESI) *m/z*: $[M+Na]^+$ calcd for $C_{15}H_{37}INaO_4Si^+$: 573.1398; found: 573.1398

Synthesis of (4*R*,5*S*,6*E*,8*Z*)-5-((tert-butyldimethylsilyl)oxy)-9-iodo-4,6,8-trimethylnona-6,8-dien-3-one (**27**):

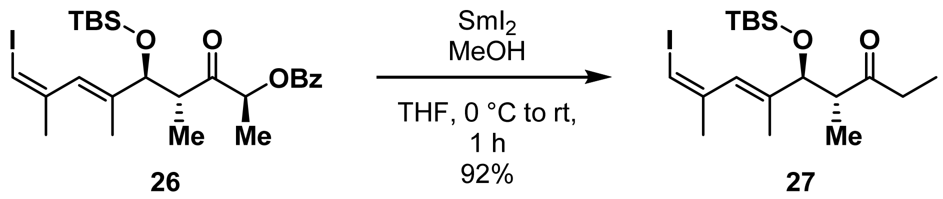

To a suspension of samarium powder (2.64 g, 17.5 mmol, 4.1 eq) in degassed, anhydrous THF (80 mL) was added diiodomethane (1.40 mL, 17.32 mmol, 4 eq) at 0 °C. The mixture was allowed to warm to rt and stirred for 2 h, to afford a deep blue solution of samarium diiodide (SmI_2).[4] The freshly prepared solution of SmI_2 was added to **26** (2.41 g, 4.33 mmol, 1.0 eq) in anhydrous MeOH (12 mL) at 0 °C. After addition, the resulting blue suspension was stirred at 0 °C for 1 h. It was then quenched with a half-saturated solution of K_2CO_3 (100 mL) and the layers were separated. The aqueous layer was extracted with EtOAc (3 x 100 mL). The combined organic extracts were dried with $MgSO_4$ and the solvent was removed under reduced pressure. The product was purified by flash chromatography (5% EtOAc in *n*-hexane) to give ethyl ketone **27** (1.74 g, 3.99 mmol, 92%) as a yellow oil.

$\mathbf{R}_f$ = 0.57 (10% EtOAc in *n*-hexane)

$[\alpha]^{20}_{578}$ = +14.4 (*c* = 1.0 in MeOH)

^{1}H NMR (400 MHz, $CDCl_3$, 298 K): δ 6.09–6.05 (m, 1H), 5.83 (s, 1H), 4.18 (d, *J* = 9.6 Hz, 1H), 2.80 (dq, *J* = 9.7, 7.0 Hz, 1H), 2.55 (dq, *J* = 7.3, 1.8 Hz, 2H), 1.97 (d, *J* = 1.2 Hz, 3H), 1.65 (d, *J* = 1.2, 3H), 1.03 (t, *J* = 7.2, 3H), 0.91 (d, *J* = 7.0 Hz, 3H), 0.82 (s, 9H), 0.00 (s, 3H), –0.01 (s, 3H) ppm

^{13}C NMR (101 MHz, $CDCl_3$, 298 K): δ 214.7, 144.4, 139.2, 130.3, 81.8, 77.5, 49.4, 38.1, 25.9, 24.7, 18.1, 14.3, 12.4, 7.4, −4.3, −5.3 ppm

HRMS (ESI) *m/z*: $[M+Na]^+$ calcd for $C_{18}H_{33}INaO_2Si^+$: 459.1187; found: 459.1187

Synthesis of (4*R*,5*S*,6*E*,8*Z*)-5-((tert-butyldimethylsilyl)oxy)-9-iodo-2,4,6,8-tetramethylnona-6,8-dien-3-one (**28**):

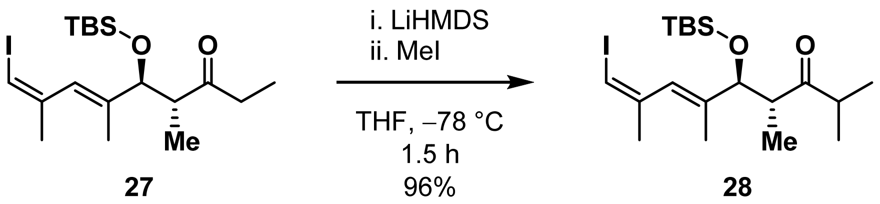

To a solution of **27** (1.70 g, 3.90 mmol, 1.0 eq) in anhydrous THF (250 mL) was added a solution of lithium bis(trimethylsilyl)amide (7.8 mL, 7.80 mmol, 2.0 eq, 1 M in THF) dropwise over 5 min at −78 °C. After 30 min, MeI (0.73 mL, 11.7 mmol, 3.0 eq) was added dropwise. The reaction was deemed complete by TLC after 1.5 h and it was quenched at 0 °C with a saturated solution of NH_4Cl (50 mL). The layers were separated and the aqueous phase was extracted with EtOAc (3 x 50 mL). The combined organic extracts were dried with $MgSO_4$ and the solvent was removed under reduced pressure. The product was purified by flash chromatography (5% EtOAc in *n*-hexane), to give ketone **28** (1.69 g, 3.75 mmol, 96%) as an orange oil.

$\mathbf{R}_f$ = 0.67 (10% EtOAc in *n*-hexane)

$[\alpha]_D^{20}$ = –3.9 (*c* = 1.0 in MeOH)

^{1}H NMR (400 MHz, $CDCl_3$, 298 K): δ 6.06–6.05 (m, 1H), 5.83 (s, 1H), 4.22 (d, *J* = 9.6 Hz, 1H), 2.97 (dq, *J* = 9.7, 7.0 Hz, 1H), 2.68 (sept, *J* = 7.0 Hz, 1H), 1.97 (d, *J* = 0.7 Hz, 3H), 1.66 (d, *J* = 1.3 Hz, 3H), 1.13 (d, *J* = 7.1 Hz, 3H), 1.08 (d, *J* = 6.8 Hz, 3H), 0.91 (d, *J* = 7.0 Hz, 3H), 0.82 (s, 9H), 0.01 (s, 3H), –0.01 (s, 3H) ppm

^{13}C NMR (101 MHz, $CDCl_3$, 298 K): δ 217.1. 144.4, 139.4, 130.3, 81.7, 77.5, 47.6, 42.6, 25.9, 24.7, 18.2, 18.1, 17.5, 15.0, 12.4 −4.3, −5.0 ppm

HRMS (ESI) *m/z*: $[M+Na]^+$ calcd for $C_{19}H_{35}INaO_2Si^+$: 473.1344; found: 473.1341

Synthesis of (4*R*,5*S*,6*E*,8*Z*)-5-hydroxy-9-iodo-2,4,6,8-tetramethylnona-6,8-dien-3-one (**29**):

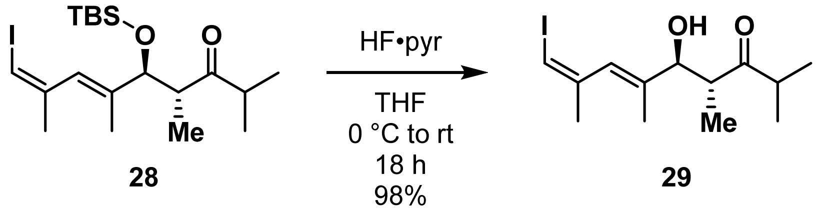

To a solution of **28** (250 mg, 0.55 mmol, 1.0 eq) in THF (20 mL) in a PFA-flask was added HF•pyridine (5 mL) at 0 °C (NOTE: HF can result in severe burns. Appropriate protective equipment must be used for this step). After 1 h the reaction mixture was warmed to rt. After 18 h, the reaction was slowly and carefully quenched with a half-saturated solution of K_2CO_3 (50 mL). The layers were separated and the aqueous phase was extracted with EtOAc (3 x 25 mL). The combined organic extracts were dried with $MgSO_4$ and the solvent was removed under reduced pressure. The product was purified by flash chromatography (20% EtOAc in *n*-hexane) to give aldol **29** (183 mg, 0.49 mmol, 98%) as an orange oil.

R_f = 0.14 (10% EtOAc in *n*-hexane)

$[\alpha]_D^{20}$ = –12.6 (*c* = 1.0 in MeOH)

^{1}H NMR (400 MHz, $CDCl_3$, 298 K): δ 6.07–6.05 (m, 1H), 5.87 (s, 1H), 4.21 (dd, *J* = 8.6, 4.0 Hz, 1H), 3.01–2.94 (m, 1H), 2.77 (sept, *J* = 6.9 Hz, 1H), 2.38 (d, *J* = 4.2 Hz, 1H), 1.96–1.95 (m, 3H), 1.70 (d, *J* = 1.2 Hz, 3H), 1.20 (d, *J* = 6.9 Hz, 3H), 1.11 (d, *J* = 6.9 Hz, 3H) 1.06 (d, *J* = 7.0 Hz, 3H) ppm

^{13}C NMR (101 MHz, $CDCl_3$, 298 K): δ 219.0, 144.5, 138.8, 130.3, 80.1, 77.6, 47.2, 41.4, 24.7, 18.1, 18.1, 15.0, 12.9 ppm

HRMS (ESI) *m/z*: $[M+Na]^+$ calcd for $C_{13}H_{21}INaO_2^+$: 359.0479; found: 359.0478

Synthesis of (4*R*,5*S*,6*E*,8*Z*)-5-hydroxy-2,4,6,8-tetramethyl-9-(trimethylstannyl)nona-6,8-dien-3-one (**13**):

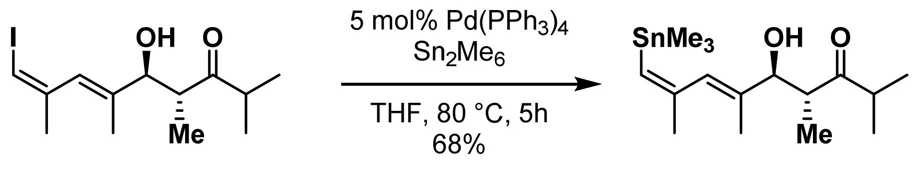

To a solution of **29** (57 mg, 0.17 mmol, 1.0 eq) and hexamethylditin (42 µL, 0.20 mmol, 1.2 eq) was added tetrakis(triphenylphosphine)palladium (9.8 mg, 8.5 µmol, 0.05 eq). The reaction mixture was heated to 80 °C in a microwave reactor for 5 h. To the resulting black suspension was added KF on Celite® (50 mg) and the mixture was stirred for 1 h to remove trimethyltin iodide. The mixture was then filtered through a short pad of Celite®, which was washed with EtOAc (20 mL). The solvent was removed under reduced pressure. Silica gel for chromatography was "deactivated" by making a slurry in 5% Et_3N in *n*-hexane and pouring this into a column. The column was washed with 3 column volumes of 10% EtOAc / 0.5% Et_3N in *n*-hexane. The product was then purified by flash chromatography (10% EtOAc / 0.5% Et_3N in *n*-hexane). Stannane **13** (43 mg, 0.12 mmol, 68%) was obtained as a colorless oil.

$\mathbf{R}_f$ = 0.64 (20% EtOAc in *n*-hexane)

^{1}H NMR (400 MHz, $CDCl_3$, 298 K): δ 6.04 (s, 1H), 5.84 (t, $^2J_{Sn\text{-}H}$ = 39.1 Hz, 1H), 4.12 (dd, J = 8.5, 4.2 Hz, 1H), 3.00–2.95 (m, 1H), 2.75 (sept, J = 6.9 Hz, 1H), 2.19 (d, J = 4.3 Hz, 1H), 2.02 (d, J = 1.3 Hz, 3H), 1.74 (d, J = 1.2 Hz, 3H), 1.12 (d, J = 7.0 Hz, 3H), 1.10 (d, J = 7.0 Hz, 3H), 0.98 (d, J = 7.1 Hz, 3H), 0.11 (t, $^2J_{Sn\text{-}H}$ = 27.5 Hz, 9H) ppm

^{13}C NMR (101 MHz, $CDCl_3$, 298 K): δ 219.1, 151.4, 136.3, 132.6, 131.6, 80.8, 47.1, 41.7, 27.6, 18.1, 18.0, 14.9, 12.6, –8.72 ppm

2.2 Synthesis of Iodide 12

Synthesis of (*Z*)-2-methylbut-2-en-1-ol (S6):[5]

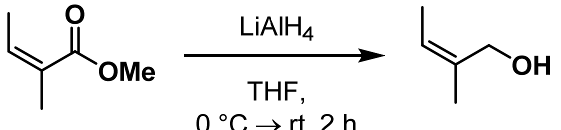

S6

To a suspension of $LiAlH_4$ (12.5 g, 329 mmol, 2.5 eq) in anhydrous THF (100 mL) was added a solution of angelic acid methyl ester (15.0 g, 131 mmol, 1.0 eq) in anhydrous THF (63 mL) in small portions via cannula at 0 °C under argon. After 15 min of stirring at this temperature, the mixture was allowed to warm to rt and stirred for 1 h. The work up was performed according to the procedure of Fieser[6] by cooling to 0 °C, before sequential addition of water (12.5 mL), freshly prepared 15% NaOH aq. (12.5 mL) and water (36 mL), followed by warming to rt and a 15 min stirring interval. $MgSO_4$ was added and the resulting white precipitate was separated via filtration through Celite® on a glass frit, followed by rinsing with Et_2O (300 mL). The filtrate was dried with $MgSO_4$, filtered and concentrated under reduced pressure to furnish angelic alcohol (**S6**) as a colorless liquid.

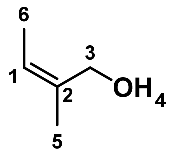

Yield: 10.5 g, 122 mmol, 93%

R*f* = 0.08 (10% EtOAc in hexane)

^{1}H NMR (400 MHz, $CDCl_3$, 298 K): δ 5.38 (q, *J* = 6.6 Hz, 1H, H-1), 4.15 (s, 2H, H-3), 1.79 (d, *J* = 1.0 Hz, 3H, H-5), 1.64 (d, *J* = 6.6 Hz, 3H, H-6) ppm

^{1}H-NMR data was consistent with literature spectra.[5]

Synthesis of (Z)-2-methylbut-2-enal (31):[7]

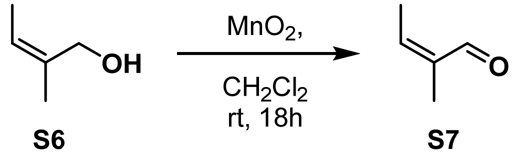

S6 **S7**

To a solution of angelic alcohol (**S6**) (10.5 g, 122 mmol, 1.0 eq) in CH_2Cl_2 (175 mL) was added powdered MnO_2 (175 g, 2.01 mol, 16.5 eq) and the resulting black mixture was stirred for 18 h at rt, while monitoring by TLC (10% EtOAc in *n*-hexane). The mixture was filtered through Celite® on a glass frit and rinsed with CH_2Cl_2 (600 mL), followed by concentration of the filtrate under reduced pressure to give aldehyde **S7** (8.40 g) as a colorless liquid. This was used in the next step without

further purification, due to its propensity to undergo *Z/E* isomerization. A crude ^{1}H NMR of **S7** showed it to be a ~95:5 ratio of *Z* and *E* stereoisomers.

^{1}H NMR (major component) (400 MHz, $CDCl_3$, 298 K): δ 10.19 (s, 1H), 6.61 (qq, J = 7.7, 1.4 Hz, 1H), 2.12 (dq, J = 7.7, 1.4 Hz, 3H), 1.77 (app p, J = 1.4 Hz, 3H) ppm

^{1}H-NMR data was consistent with literature spectra.[7]

Synthesis of Ethyl 2-(bis(2,2,2-trifluoroethoxy)phosphoryl)propanoate (31):

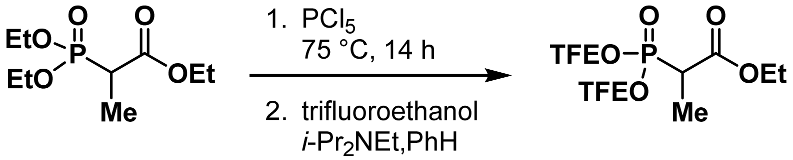

31

35% (2 steps)

To ethyl 2-(diethoxyphosphoryl)propanoate (55.0 g, 231 mmol, 1.00 eq.) was added PCl_5 (125 g, 600 mmol, 2.60 eq.) at 0 °C under argon. The reaction vessel was equipped with a reflux condenser and the lime green slurry was heated at 75 °C overnight. After cooling to ambient temperature, the reflux condenser was replaced with a short distillation bridge. The product (ethyl 2-(dichlorophosphoryl)propanoate) was purified by distillation at 85–90 °C (3.6×10^{-2} mbar). NOTE: Multiple lower boiling components distill/sublime first and patience is recommended to achieve proper separation. The product was subjected to the next step without further purification. To a solution of the intermediate product in benzene (200 mL) was added a solution of trifluoroethanol (24.1 mL, 334 mmol, 2.50 eq.) and *i*-Pr_2NEt (58.3 mL, 334 mmol, 2.50 eq.) in benzene (240 mL) via cannula at 0 °C under argon. After 20 min of stirring at this temperature, the mixture was allowed to warm to rt and stirred for 3 h. The reaction progress was monitored by TLC (30% ethyl acetate in hexane) and ^{31}P NMR. The solvent was removed under reduced pressure and the residual yellow slurry was suspended in 20% EtOAc in hexane, followed by filtration of the crystalline solid impurity and rinsing. The filtrate was concentrated and purified by column chromatography (SiO_2, 30% → 50% EtOAc in hexane), furnishing the desired product as a yellow oil.

Yield: 27.7 g, 80.0 mmol, 35% (2 steps)

R$_f$ = (30% EtOAc in hexane) = 0.36

^{1}H-NMR (400 MHz, $CDCl_3$, 298 K): δ 4.48–4.20 (m, 6H, H-8, 9, 12), 4.23 (m, 2H, H-12) 3.18 (dq, $^{2}J_{P\text{-}H}$ = 22.5 Hz, J = 7.4 Hz, 1H, H-1), 1.51 (dd, $^{3}J_{P\text{-}H}$ = 19.4 Hz, J = 7.4 Hz, 3H, H-4), 1.29 (t, J = 7.2 Hz, 3H, H-13) ppm

^{31}P{^{1}H}-NMR (162 MHz, $CDCl_3$, 298 K): δ 30.54 ppm

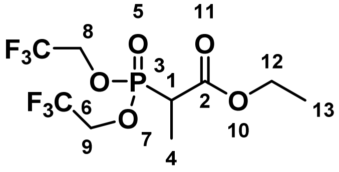

Synthesis of Ethyl (2*Z*,4*Z*)-2,4-dimethylhexa-2,4-dienoate (**32**):

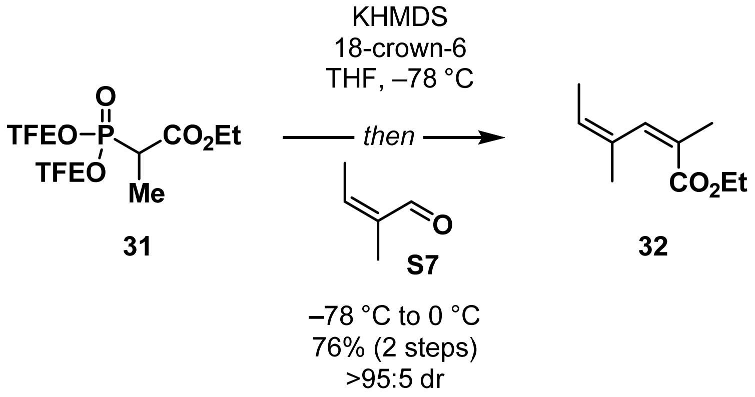

To a solution of ethyl 2-(bis(2,2,2-trifluoroethoxy)phosphoryl)propanoate (**31**) (38.0 g, 110 mmol, 1.10 eq) and 18-crown-6 (79.2 g, 300 mmol, 3.00 eq) in dry THF (1 L) under argon was added a 1 M solution of KHMDS in THF (110 mL, 110 mmol, 1.10 eq) dropwise at –78 °C. The resulting orange-colored mixture was stirred for 1 h at this temperature, followed by dropwise addition of a solution of angelic aldehyde (**S7**) (8.40 g, 100 mmol, 1.00 eq) in THF (133 mL) via cannula. The cloudy orange mixture was stirred for 1 h before warming to 0 °C and quenching with water (30 mL). The mixture was partially concentrated in vacuo and then diluted with hexane (400 mL) and water (400 mL). The layers were separated and the aqueous layer was extracted with hexane (3 × 100 mL). The combined organic layers were washed with water (3 × 100 mL), 5% $NaHCO_3$ (250 mL) and brine (3 × 250 mL), dried with $MgSO_4$, filtered and concentrated under reduced pressure to give a yellow oil. This material was determined to be a 93:7 mixture of alkene isomers by ^{1}H NMR. The two isomers were indistinguishable by TLC, but the corresponding alcohols after the subsequent DIBAL reduction step had reasonably different R_f values, and could be separated by column chromatography. We therefore did not fully purify **32** at this stage.

Yield: 14.2 g, 84.4 mmol, 76% (2 steps), 93:7 dr.

R$_f$ = 0.73 (20% EtOAc in hexane)

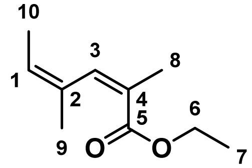

^{1}H NMR (major component) (400 MHz, $CDCl_3$, 298 K)**:** δ 6.28 (s, 1H, H-3), 5.39 – 5.31 (m, 1H, H-1), 4.17 (q, *J* = 7.1, 2H, H-6), 1.99 (d, *J* = 1.4 Hz, 3H, H-9), 1.78 (s, 3H, H-8), 1.58 (d, *J* = 6.9 Hz, 3H, H-10), 1.27 (t, *J* = 7.1 Hz, 3H, H-7) ppm

Synthesis of (2*Z*,4*Z*)-2,4-dimethylhexa-2,4-dien-1-ol (20):

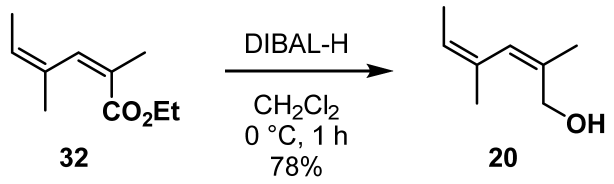

To a solution of ester **32** (5.70 g, 33.9 mmol, 1.00 eq., 93:7 mixture of *Z/E* isomers) in dry CH_2Cl_2 (180 mL) was added a 1 M solution of DIBAL-H in toluene (91.5 mL, 91.5 mmol, 2.70 eq) dropwise over 30 min at 0 °C. The mixture was stirred for 1.5 h, before quenching by slow addition of methanol (40 mL). The resulting white slurry was warmed to ambient temperature and treated with saturated sodium potassium tartrate solution (300 mL). The mixture was transferred to an Erlenmeyer flask and stirred vigorously for 1 h, followed by filtration through a Celite® pad, which was then thoroughly washed with EtOAc (2 x 200 mL). The phases were separated and the aqueous layer was extracted with EtOAc (3 × 100 mL). The combined organic phases were dried with Na_2SO_4, filtered and concentrated under reduced pressure. The oily residue was purified by column chromatography (SiO_2, 20% EtOAc in hexane), furnishing alcohol **20** in high stereoisomeric purity (the minor diastereomer could be separated at this stage) as a colorless liquid.

Yield: 3.34 g, 26.5 mmol, 78%

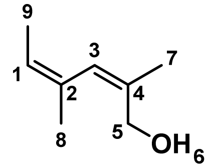

R$_f$ = 0.45 (20% EtOAc in hexane)

^{1}H NMR (400 MHz, $CDCl_3$, 298 K): δ 5.68 (br s, 1H, H-3), 5.34 (qdq, *J* = 6.7, 2.8, 1.4 Hz, 1H, H-1), 4.05 (s, 2H, H-5), 1.87 (d, *J* = 1.5 Hz, 3H, H-7), 1.74 (m, 3H, H-8), 1.50 (ddq, *J* = 6.7, 2.8, 1.4 Hz, 3H, H-9) ppm

^{13}C NMR (101 MHz, $CDCl_3$, 298 K): δ 135.9 (C_q, C-4), 133.5 (C_q, C-2), 126.8 (CH, C-3), 122.6 (CH, C-1), 63.4 (CH_2, C-5), 24.4 (CH_3, C-8), 20.8 (CH_3, C-7), 15.0 (CH_3, C-9) ppm

HRMS (EI): *m/z* $[M]^{+•}$ calcd for $C_8H_{14}O^+$: 126.1045; found: 126.1034

Synthesis of ((2*R*,3*S*)-3-((*Z*)-but-2-en-2-yl)-2-methyloxiran-2-yl)methanol (**33**):

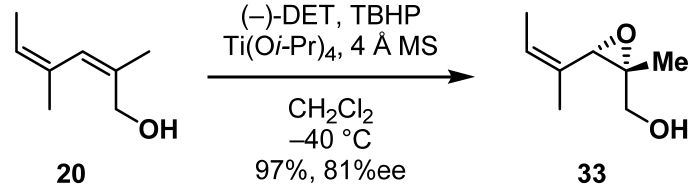

In a dry flask, powdered, activated 4 Å molecular sieves (6.0 g) were suspended in anhydrous CH_2Cl_2 (177 mL) under argon and cooled to –25 °C, before sequential addition of (–)-DET (1.93 mL, 11.3 mmol, 0.27 eq) and Ti(O*i*-Pr)$_4$ (2.85 mL, 9.64 mmol, 0.23 eq). The pale-yellow suspension was stirred for 0.5 h and a solution of TBHP in decane (5.5 M, 17.0 mL, 93.5 mmol, 2.20 eq) was added dropwise. After 30 min, the mixture was cooled to –40 °C and a solution of allylic alcohol **20** (5.29 g, 41.9 mmol, 1.00 eq) in CH_2Cl_2 (175 mL) was added dropwise over 1 h, followed by stirring at –40 °C while monitoring the reaction progress via TLC (20% ethyl acetate in hexane). After 24 h, the reaction mixture was warmed to –10 °C and quenched by slow addition of 40% NaOH in brine (35 mL) and stirred for 20 min. Anhydrous $MgSO_4$ was added and the mixture was stirred for 10 min while warming to ambient temperature, then filtered through a Celite® pad on a glass frit. The filtrate was dried with $MgSO_4$, filtered and concentrated under reduced pressure to give the crude product as a pale-yellow liquid. Purification by column chromatography (SiO_2, 20% ethyl acetate in hexane) furnished epoxide **33** as a colorless liquid.

Yield: 5.77 g, 40.6 mmol, 97%, 81% ee (determined by conversion to the Mosher ester)

$\mathbf{R}_f$ = 0.25 (20% EtOAc in hexane)

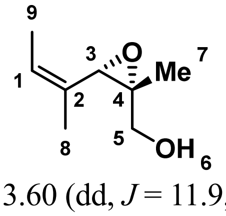

$[\alpha]^{20}_{578}$ = +62.5 (*c* = 1.2 in MeOH)

^{1}H NMR (400 MHz, $CDCl_3$, 298 K)**:** δ 5.34 (qdq, *J* = 6.9, 3.1, 1.2 Hz, 1H, H-1), 3.60 (dd, *J* = 11.9, 7.4 Hz, 1H, H-5), 3.48 (dd, *J* = 11.9, 4.4 Hz, 1H, H-5), 3.43 (d, *J* = 1.2 Hz, 1H, H-3), 1.69 (dq, *J* =

3.1, 1.5 Hz, 3H, H-8), 1.64 (ddq, *J* = 6.9, 3.1, 1.5 Hz, 3H, H-9), 1.53 (dd, *J* = 7.4, 4.4 Hz, 1H, H-6), 1.47 (s, 3H, H-7) ppm

^{13}C NMR (101 MHz, $CDCl_3$, 298 K): δ 130.6 (C_q, C-2), 123.2 (CH, C-1), 65.1 (CH_2, C-5), 64.2 (CH, C-3), 61.6 (C_q, C-4), 20.5 (CH_3, C-8), 19.5 (CH_3, C-7), 14.0 (CH_3, C-9) ppm

HRMS (APCI) *m/z* $[M]^{+\bullet}$ calcd for $C_8H_{15}O_2^+$: 143.1067; found 143.1058

^{1}H and ^{19}F NMR spectra of the Mosher ester with insert showing the integration used for ee determination:

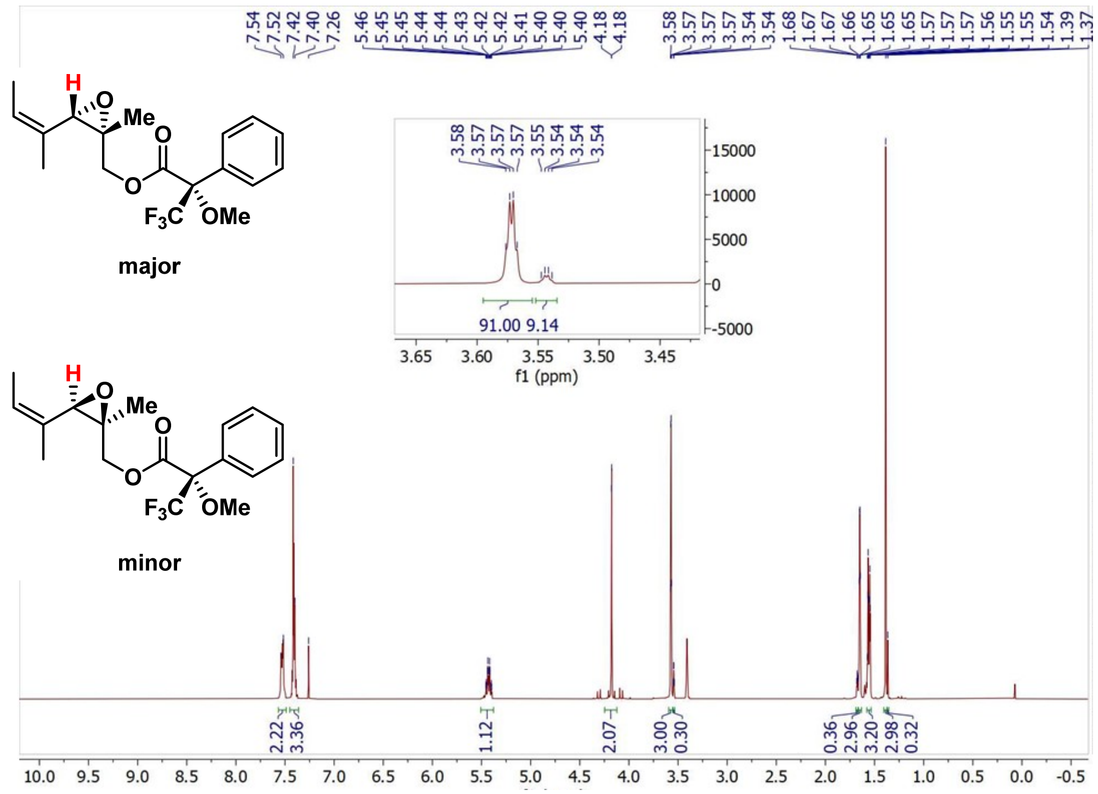

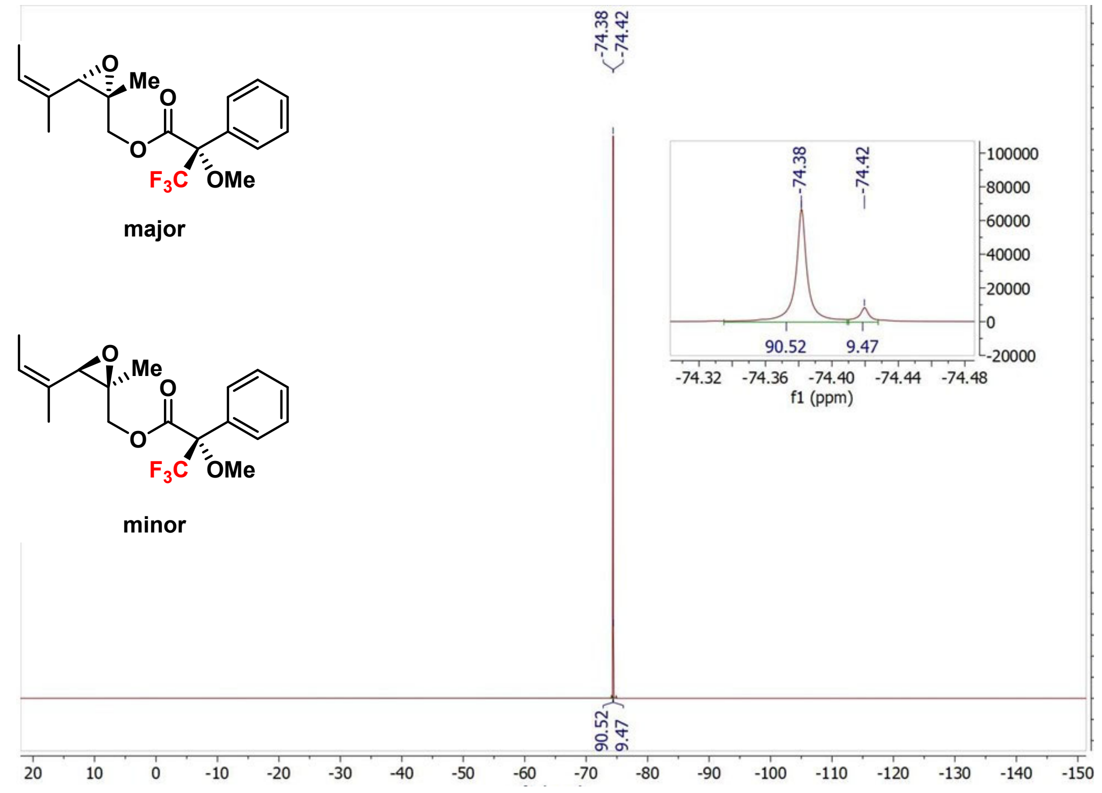

Synthesis of (2*R*,3*R*)-2-((2*S*,3*R*)-3-(hydroxymethyl)-3-methyloxiran-2-yl)butane-2,3-diol (19):

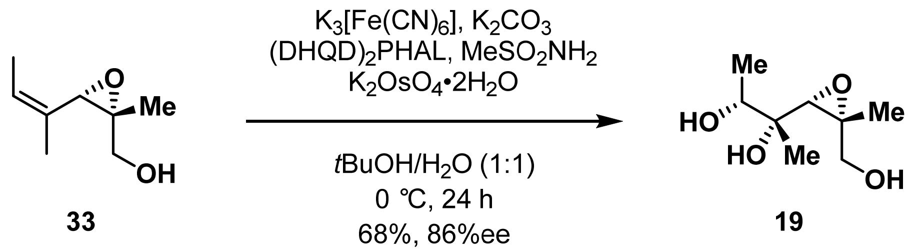

To a mixture of *tert*-butanol and water (1:1, 392 mL) were added potassium ferricyanide (38.7 g, 118 mmol, 3.00 eq), K_2CO_3 (16.2 g, 118 mmol, 3.00 eq), $(DHQD)_2PHAL$ (0.61 g, 0.78 mmol, 0.02 eq), potassium osmate(VI) dihydrate (0.14 g, 0.39 mmol, 0.01 eq) and methyl sulfonamide (3.73 g, 39.2 mmol, 1.00 eq) at 0 °C under an argon atmosphere. The red suspension was stirred for 30 min at this temperature before dropwise addition of olefin **33** (5.57 g, 39.2 mmol, 1.00 eq., 81% ee). The transfer was quantitated by rinsing with *t*-BuOH (2 x 1 mL). Stirring was continued at 0 °C for 24 h, accompanied by a color change of the reaction mixture from red to yellow. The reaction was quenched by addition of a saturated Na_2SO_3 solution (180 mL) and stirred for 30 min without a cooling bath. The layers were separated and the aqueous layer was extracted with EtOAc (4 × 100 mL). The combined organic layers were dried with $MgSO_4$, filtered and concentrated to give an 86:14 mixture of diastereomers (determined by ^{1}H NMR), which was further purified by column chromatography (SiO_2, 5% → 10% → 20% MeOH in CH_2Cl_2) to give triol **19** as a light green oil.

Yield: 4.67 g, 26.5 mmol, 68% yield, 86% ee

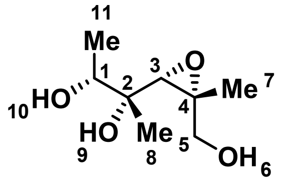

$\mathbf{R_f}$ = 0.38 (10% MeOH in CH_2Cl_2)

$[\alpha]_D^{20}$ = −18.4 (*c* = 0.8 in MeOH)

^{1}H NMR (400 MHz, $CDCl_3$, 298 K): δ 4.05 (d, *J* = 12.1 Hz, 1H H-5), 3.86 (d, *J* = 12.1 Hz, 1H, H-5), 3.66 (q, *J* = 6.5 Hz, 1H, H-1), 3.07 (br s, 1H, OH), 2.88 (s, 1H, H-3), 2.49 (br s, 1H, OH), 2.14 (br s, 1H, OH), 1.43 (s, 3H, H-7), 1.30 (d, *J* = 6.6 Hz, 3H, H-11), 1.27 (s, 3H, H-8) ppm

^{13}C NMR (101 MHz, $CDCl_3$, 298 K): δ 74.9 (CH, C-1), 71.9 (C_q, C-2), 66.9 (CH, C-3), 64.1 (CH_2, C-5), 61.9 (C_q, C-4), 21.7 (CH_3, C-8), 21.5 (CH_3, C-7), 18.0 (CH_3, C-11) ppm

HRMS (EI) *m/z* $[M+H]^+$ calcd for $C_8H_{17}O_4^+$: 177.1121; found 177.1126

The enantiomeric excess of triol **19** was determined by 3-step conversion to alcohol **40** followed by Mosher ester analysis. For this analysis, acetal **18** was not crystallized, to prevent any resolution from taking place. An ee of 86% was calculated.

^{1}H and ^{19}F NMR spectra of the Mosher ester with insert showing the integration used for ee determination:

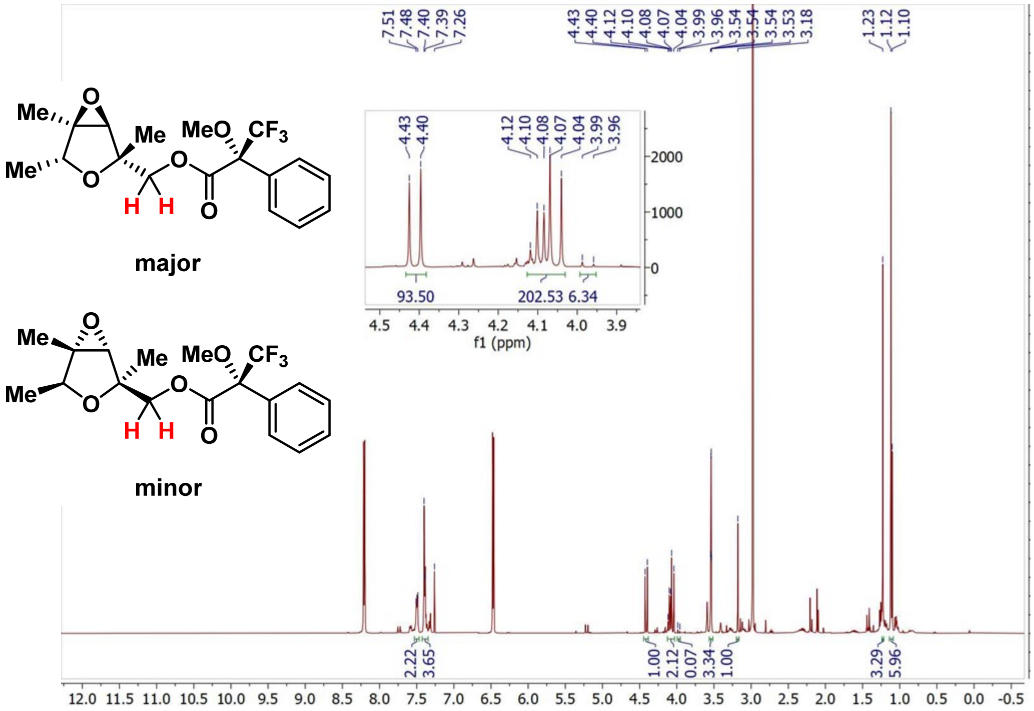

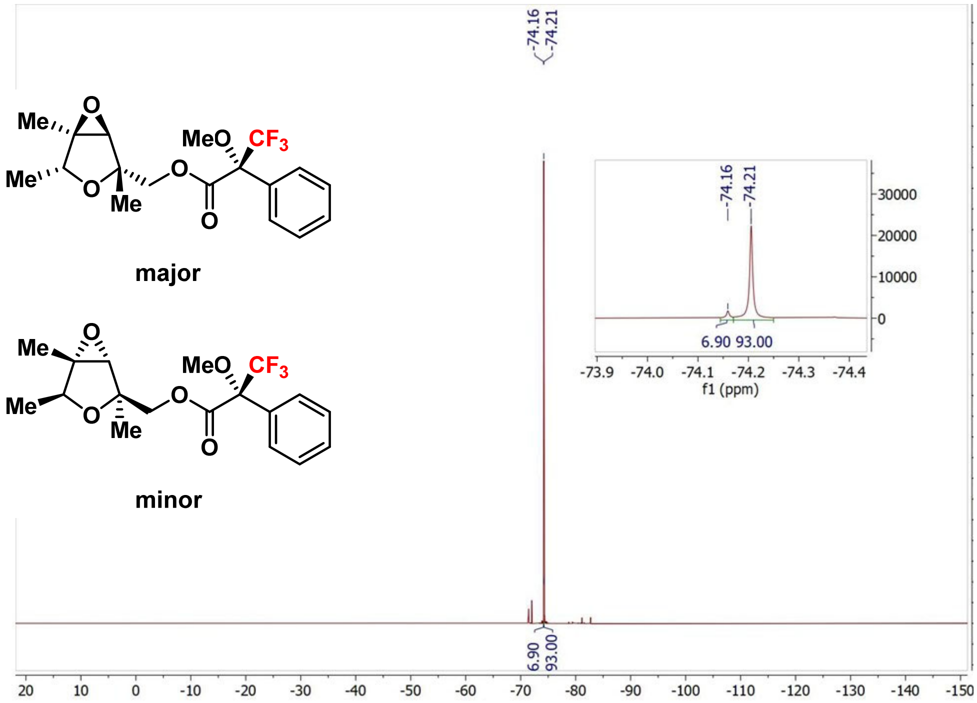

Synthesis of (2*S*,3*S*,4*S*,5*R*)-2-(hydroxymethyl)-2,4,5-trimethyltetrahydrofuran-3,4-diol (34):

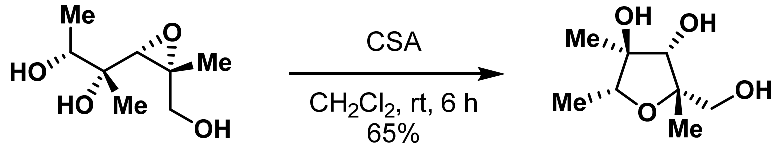

19 **34**

To a solution of epoxide **19** (4.67 g, 26.5 mmol, 1.00 eq) in dry CH_2Cl_2 (530 mL) was added CSA (0.68 g, 2.91 mmol, 0.11 eq) and the solution was stirred at rt for 6 h under argon. After neutralization with Et_3N (0.41 mL, 2.94 mmol, 0.11 eq), the solvent was removed under reduced pressure and the oily residue was purified via column chromatography (SiO_2, 5% MeOH in CH_2Cl_2) to furnish tetrahydrofuan **34** as a colorless oil.

Yield: 3.03 g, 17.2 mmol, 65%

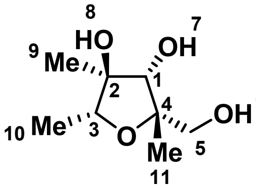

R*f* = 0.38 (10% MeOH in CH_2Cl_2)

$[\alpha]^{20}_{578}$ = +21.9 (*c* = 1.0 in MeOH)

^{1}H NMR (400 MHz, $CDCl_3$, 298 K): δ 3.98 (br d, *J* = 5.6 Hz, 1H, H-1), 3.93 (q, *J* = 6.5 Hz, 1H, H-3), 3.70–3.62 (m, 2H, H-5), 3.20 (br d, *J* = 8.0 Hz, 1H, OH), 2.34 (br s, 1H, OH), 1.70 (br s, 1H, OH), 1.30 (s, 3H, H-11), 1.22 (s, 3H, H-9), 1.20 (d, *J* = 6.5 Hz, 3H, H-10) ppm

^{13}C NMR (101 MHz, $CDCl_3$, 298 K): δ 88.7 (CH, C-1), 81.6 (C_q, C-2), 81.6 (C_q, C-4), 77.7 (CH, C-3), 67.2 (CH_2, C-5), 23.4 (CH_3, C-11), 16.7 (CH_3, C-9), 14.8 (CH_3, C-10) ppm

HRMS (EI) *m/z*: $[M+H]^+$ calcd for $C_8H_{17}O_4^+$: 177.1121; found 177.1110

Synthesis of (4a*S*,6*R*,7*R*,7a*S*)-2-(4-bromophenyl)-4a,6,7-trimethyltetrahydro-4H-furo[3,2-d][1,3]dioxin-7-ol (36):

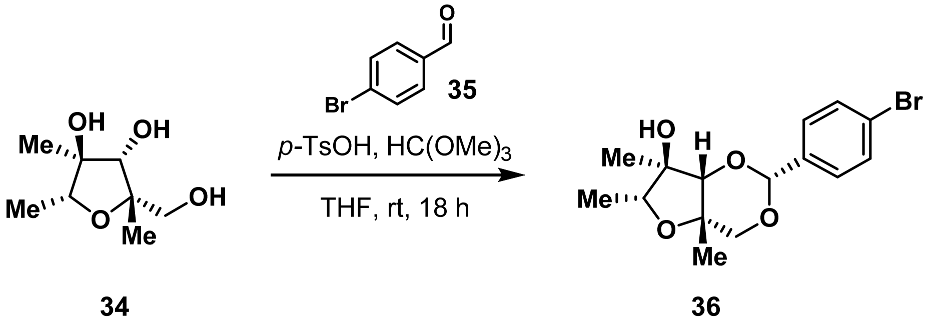

To a solution of alcohol **34** (100 mg, 0.57 mmol, 1.00 eq) in THF (2.8 mL) was added 4-bromo benzaldehyde (**35**) (105 mg, 0.57 mmol, 1.00 eq), *p*-TsOH (122 mg, 0.11 mmol, 0.20 eq) and trimethyl orthoformate (66.3 mg, 0.62 mmol, 1.10 eq) at room temperature. The reaction mixture was stirred for 18 h before quenching with solid $NaHCO_3$ (50 mg). After stirring for 5 min, the light green suspension was filtered through cotton and concentrated to yield the crude product as a dark green foam. ^{1}H NMR analysis of the crude mixture indicated a dr of 95:5 with respect to the newly formed acetal stereocenter. Purification via column chromatography (SiO_2, 3% MeOH in CH_2Cl_2) furnished **36** as a white solid. Crystals suitable for single crystal X-ray structural analysis were obtained by vapor diffusion experiments using an Et_2O/hexane solvent system at 4 °C.

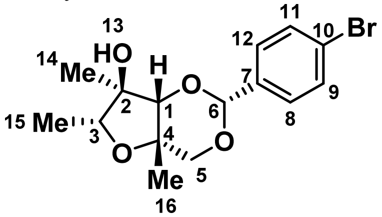

Yield: not determined.

R*f* = 0.55 (75% EtOAc in hexane)

^{1}H NMR (400 MHz, $CDCl_3$, 298 K): δ 7.49 (d, *J* = 8.5 Hz, 2H, H-9, H-11), 7.35 (d, *J* = 8.3 Hz, 2H, H-8, H-12), 5.38 (s, 1H, H-6), 4.17 (q, *J* = 6.7 Hz, 1H, H-3), 4.08 (d, *J* = 12.6 Hz, 1H, H-5), 3.81 (d, *J* = 12.6 Hz, 1H, H-5), 3.90 (s, 1H, H-1), 1.37 (s, 3H, H-16), 1.36 (s, 3H, H-14), 1.20 (d, *J* = 6.7 Hz, 3H, H-15) ppm

^{13}C NMR (101 MHz, $CDCl_3$, 298 K): δ 137.6 (C_q, C-10), 131.6 (CH, C-9, C-11), 128.1 (CH, C-8, C-12), 123.1 (C_q, C-7), 98.4 (CH, C-6), 88.1 (CH, C-1), 84.9 (CH, C-3), 83.6 (C_q, C-2), 76.2 (C_q, C-4), 72.5 (CH_2, C-5), 22.0 (CH_3, C-16), 20.5 (CH_3, C-14), 19.0 (CH_3, C-15) ppm

HRMS (ESI) *m/z*: $[M+Na]^+$ calcd for $C_{15}H_{19}BrNaO_4^+$: 365.0359; found 365.0358

Synthesis of (4a*S*,6*R*,7*R*,7a*S*)-2-(4-methoxyphenyl)-4a,6,7-trimethyltetrahydro-4H-furo[3,2-d][1,3]dioxin-7-ol (18):

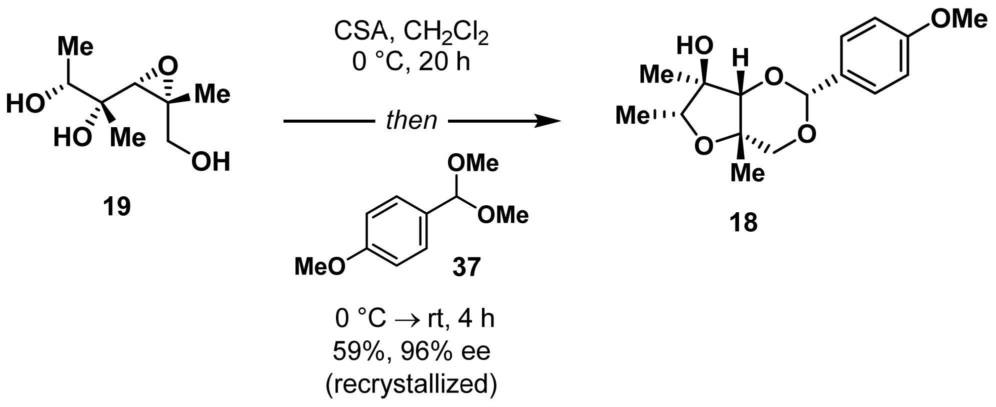

To a solution of triol **19** (1.45 g, 8.23 mmol, 1.00 eq) in anhydrous CH_2Cl_2 (165 mL) was added camphorsulfonic acid (0.19 g, 0.82 mmol, 0.10 eq) under argon at 0 °C, and the colorless solution was stirred for 20 h. Complete conversion of the starting material was verified by ^{1}H NMR of a small aliquot. Then anisaldehyde dimethylacetal (**37**) (2.10 mL, 12.34 mmol, 1.50 eq) was added to the now purple solution at 0 °C. Stirring was continued at this temperature for 1 h, during which a color change to green was observed. The reaction was warmed to ambient temperature and stirred for 3 h, before neutralizing with Et_3N (125 μL, 0.90 mmol, 0.11 eq). To the now yellow solution was added saturated aqueous $NaHCO_3$ solution (300 mL) and CH_2Cl_2 (100 mL). The layers were separated and the aqueous layer was extracted with CH_2Cl_2 (3 × 100 mL). The combined organic phases were dried with $MgSO_4$, filtered and concentrated. ^{1}H NMR of the crude material indicated the stereocenter at the acetal was formed with >95:5 diastereoselectivity. The solid residue was recrystallized twice from boiling EtOAc/hexane to afford acetal **18** as colorless needles, which were determined to be diastereomerically pure by ^{1}H NMR. The ee of this material was determined to be 96%.

Yield: 1.44 g, 4.89 mmol, 59%, 96% ee

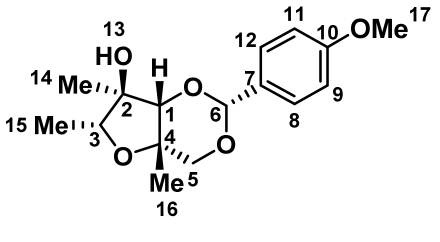

$\mathbf{R_f}$ = 0.20 (40% EtOAc in hexane)

$[\alpha]^{20}_{578}$ = +17.6 (c = 0.9 in MeOH)

^{1}H NMR (600 MHz, $CDCl_3$, 298 K): δ 7.40 (m, 2H, H-8, 12), 6.89 (m, 2H, H-9, 11), 5.38 (s, 1H, H-6), 4.18 (q, J = 6.8 Hz, 1H, H-3), 4.08 (d, J = 12.5 Hz, 1H, H-5), 3.81 (d, J = 12.5 Hz, 1H, H-5),

3.89 (s, 1H, H-1), 3.81 (s, 3H, H-17), 1.58 (s, 1H, OH), 1.37 (s, 3H, H-16), 1.37 (s, 3H, H-14), 1.30 (d, J = 6.8 Hz, 3H, H-15) ppm

^{13}C NMR (151 MHz, $CDCl_3$, 298 K): δ 160.2 (C_q, C-10), 131.1 (C_q, C-7), 127.7 (CH, C-8, 12), 113.8 (CH, C-9, 11), 99.1 (CH, C-6), 88.0 (CH, C-1), 84.9 (CH, C-3), 83.7 (C_q, C-2), 76.2 (C_q, C-4), 72.6 (CH_2, C-5), 55.5 (CH_3, C-17), 22.1 (CH_3, C-16), 20.5 (CH_3, C-14), 19.1 (CH_3, C-15) ppm

HRMS (ESI) *m/z*: $[M+Na]^+$ calcd for $C_{16}H_{22}NaO_5^+$: 317.1359; found 317.1359

Similar to the analysis of **19**, the ee of recrystallized **18** was determined by converting it into alcohol **40**, followed by Mosher ester analysis. An ee of ~96% was calculated.

^{1}H spectrum of the Mosher ester with insert showing the integration used for ee determination:

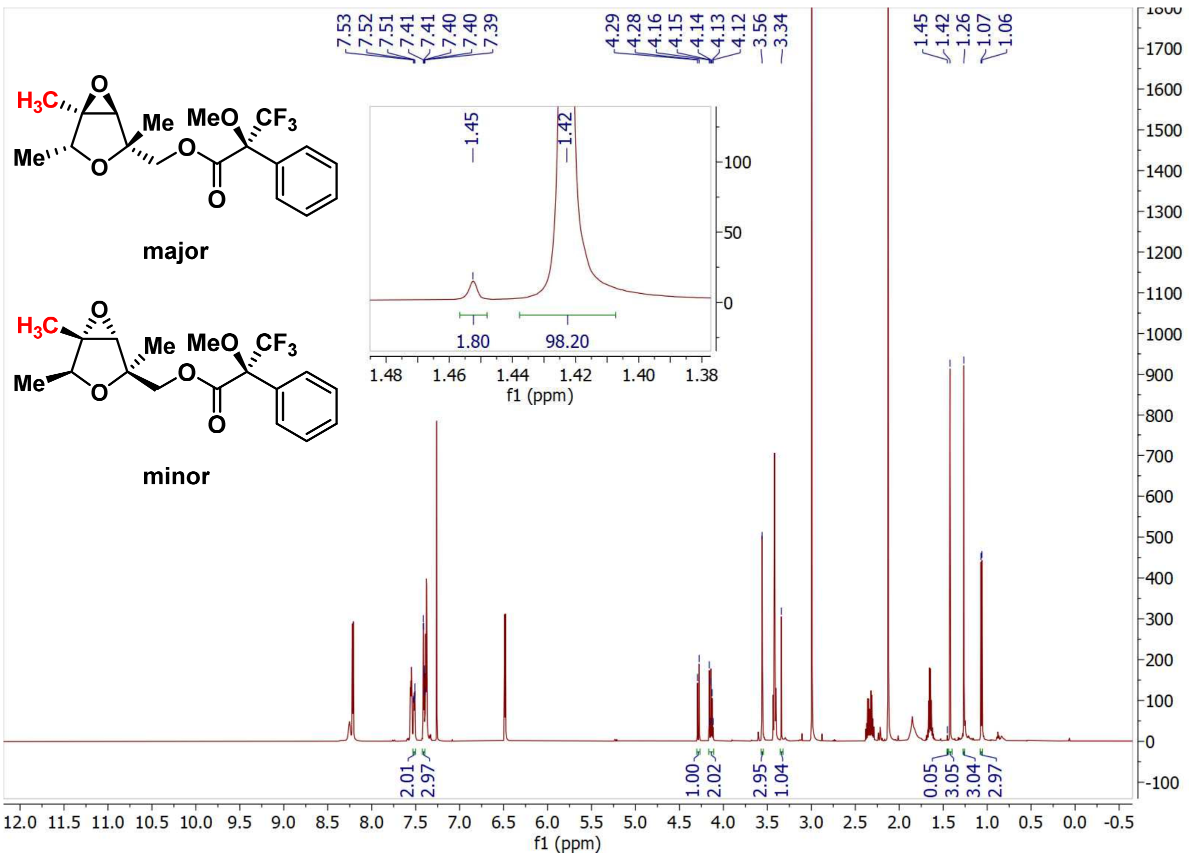

Synthesis of ((1*S*,2*S*,4*R*,5*R*)-2,4,5-trimethyl-3,6-dioxabicyclo[3.1.0]hexan-2-yl)methyl 4-methoxybenzoate (39):

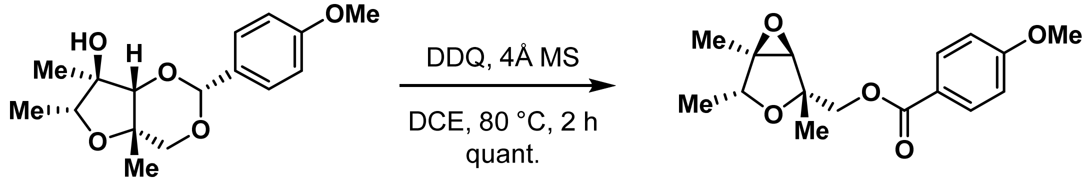

18 39

To a mixture of acetal **18** (1.49 g, 5.05 mmol, 1.00 eq) and powdered, activated 4Å molecular sieves (3.00 g) in anhydrous dichloroethane (125 mL) was added DDQ (1.50 g, 6.61 mmol, 1.30 eq) under argon. The resulting dark green mixture was heated to 80 °C for 2 h. The reaction was allowed to cool to ambient temperature and quenched by addition of aqueous saturated $NaHCO_3$ solution (350 mL) and brine (200 mL). The layers were separated and the aqueous layer was extracted with CH_2Cl_2 (3 × 100 mL). The combined organic phases were washed with brine (2 × 250 mL), dried with $MgSO_4$, filtered and concentrated to afford the desired epoxide **39** as a brown solid. No further purification was required.

Yield: 1.47 g, 5.05 mmol, quantitative

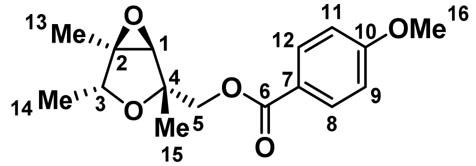

$\mathbf{R}_f$ = 0.62 (40% EtOAc in hexane)

$[\alpha]^{20}_{578}$ = +29.1 (*c* = 1.1 in MeOH)

^{1}H NMR (600 MHz, $CDCl_3$, 298 K): δ 8.01 (d, *J* = 9.0 Hz, 2H, H-8, 12), 6.95 (d, *J* = 9.0 Hz, 2H, H-9, 11), 4.28 (d, *J* = 12.5 Hz, 1H, H-5), 4.26 (d, *J* = 12.5 Hz, 1H, H-5), 4.20 (q, *J* = 6.9 Hz, 1H, H-3), 3.88 (s, 1H, H-16), 3.58 (s, 1H, H-1), 1.46 (s, 3H, H-13), 1.38 (s, 3H, H-15), 1.26 (d, *J* = 6.9 Hz, 3H, H-14) ppm

^{13}C NMR (151 MHz, $CDCl_3$, 298 K): δ 166.1 (C_q, C-6), 163.8 (C_q, C-10), 131.9 (CH, C-8, 12), 122.3 (C_q, C-7), 114.0 (CH, C-9, 11), 80.3 (C_q, C-4), 77.7 (CH, C-3), 68.8 (CH_2, C-5), 68.1 (C_q, C-2), 67.4 (CH, C-1), 55.7 (CH_3, C-16), 20.0 (CH_3, C-14), 19.1 (CH_3, C-15), 14.2 (CH_3, C-13) ppm

HRMS (ESI) *m/z*: $[M+Na]^+$ calcd for $C_{16}H_{20}NaO_5^+$: 315.1203; found 315.1204

Synthesis of ((1*S*,2*S*,4*R*,5*R*)-2,4,5-trimethyl-3,6-dioxabicyclo[3.1.0]hexan-2-yl)methanol (40):

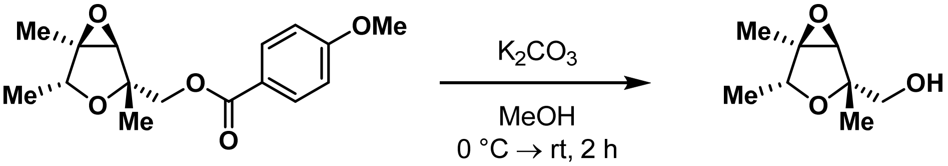

39 **40**

To a solution of ester **39** (0.81 g, 2.77 mmol, 1.0 eq) in anhydrous MeOH (28 mL) was added potassium carbonate (2.30 g, 16.6 mmol, 6.0 eq) at 0 °C under argon. The resulting yellow mixture was allowed to warm to rt over 2 h. The reaction was quenched with a saturated aqueous NH_4Cl solution (250 mL) and diluted with CH_2Cl_2 (250 mL). The phases were separated and the aqueous layer was extracted with CH_2Cl_2 (5 × 100 mL). The combined organic phases were dried with $MgSO_4$, filtered and concentrated to afford the crude product. Purification by flash column chromatography (SiO_2, 50% EtOAc in hexane + 0.5% Et_3N) yielded alcohol **40** as a colorless oil.

Yield: 391 mg, 2.47 mmol, 89%

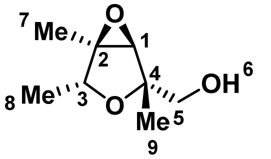

$\mathbf{R}_f$ = 0.15 (40% EtOAc in hexane)

$[\alpha]^{20}_{578} = -6.1$ (*c* = 1.0 in MeOH)

^{1}H NMR (600 MHz, $CDCl_3$, 298 K): δ 4.19 (q, *J* = 6.9 Hz, 1H, H-3), 3.56 (d, *J* = 11.0 Hz, 1H, H-5), 3.54 (d, *J* = 11.0 Hz, 1H, H-5), 3.42 (s, 1H, H-1), 1.46 (s, 3H, H-7), 1.27 (s, 3H, H-9), 1.25 (d, *J* = 6.9 Hz, 3H, H-8) ppm

^{13}C NMR (151 MHz, $CDCl_3$, 298 K): δ 82.0 (C_q, C-4), 77.5 (CH, C-3), 67.9 (C_q, C-2), 67.9 (CH_2, C-5), 67.7 (CH, C-1), 20.1 (CH_3, C-8), 18.4 (CH_3, C-9), 14.2 (CH_3, C-10) ppm

HRMS (EI) *m/z* $[M]^{+\bullet}$ calcd for $C_8H_{14}O_3^{+\bullet}$: 158.0937; found 158.0931

Synthesis of (1*R*,2*R*,4*R*,5*R*)-2,4,5-trimethyl-3,6-dioxabicyclo[3.1.0]hexane-2-carbaldehyde (17):

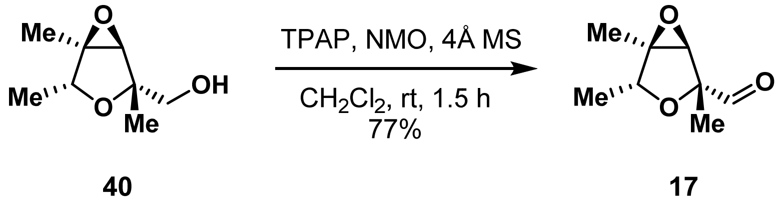

To a mixture of alcohol **40** (0.39 g, 2.47 mmol, 1.00 eq) and powdered activated 4Å molecular sieves (1.50 g) in anhydrous CH_2Cl_2 (25 mL) was added NMO (434 mg, 3.71 mmol, 1.50 eq) and the mixture was stirred for 15 min at rt. To this was added TPAP (43.3 mg, 0.12 mmol, 0.05 eq) and the black mixture was stirred at rt for 1.5 h. The reaction mixture was filtered through a 4 cm plug of silica gel and rinsed with CH_2Cl_2 (600 mL), before removing the solvent under reduced pressure to afford the crude aldehyde **17** as a pale-yellow oil, which was pure enough to be used further without performing chromatography.

Yield: 297 mg, 1.90 mmol, 77%

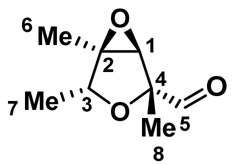

R*f* = 0.46 (40% EtOAc in hexane)

$[\alpha]_{578}^{20}$ = −4.4 (*c* = 0.5 in MeOH).

^{1}H NMR (600 MHz, $CDCl_3$, 298 K): δ 9.70 (s, 1H, H-5), 4.26 (q, *J* = 6.8 Hz, 1H, H-3), 3.63 (s, 1H, H-1), 1.45 (s, 3H, H-6), 1.35 (s, 3H, H-8), 1.20 (d, *J* = 6.8 Hz, 3H, H-7) ppm

^{13}C NMR (151 MHz, $CDCl_3$, 298 K): δ 203.9 (CH, C-5), 85.3 (C_q, C-4), 77.9 (CH, C-3), 67.2 (C_q, C-2), 64.4 (CH, C-1), 19.4 (CH_3, C-7), 16.8 (CH_3, C-8), 13.7 (CH_3, C-6) ppm

Synthesis of (*E*)-2-methyl-3-((1*S*,2*S*,4*R*,5*R*)-2,4,5-trimethyl-3,6-dioxabicyclo[3.1.0]hexan-2-yl)acrylaldehyde (42):

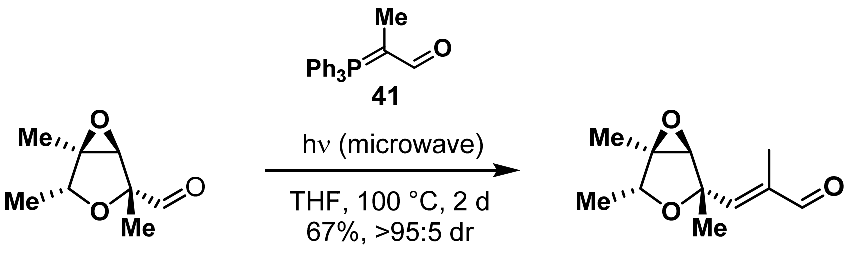

17 42

To a solution of aldehyde **17** (297 mg, 1.90 mmol, 1.00 eq) in anhydrous THF (20 mL) under argon was added 2-(triphenylphosphoranylidene)propionaldehyde (**41**) (632 mg, 3.71 mmol, 1.04 eq) and the mixture was irradiated in the microwave at 100 °C for 2 d. After cooling to ambient temperature, the reaction mixture was diluted with Et_2O (200 mL) and washed with aqueous saturated NH_4Cl solution (200 mL), water (200 mL), dried ($MgSO_4$), filtered and concentrated. Purification by flash column chromatography (SiO_2, 20% EtOAc in hexane) afforded aldehyde **42** as a colorless oil.

Yield: 250 mg, 1.27 mmol, 67%

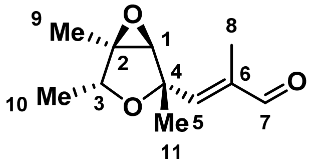

R*f* = 0.33 (20% EtOAc in hexane)

$[\alpha]^{20}_{578}$ = −38.8 (*c* = 0.5 in MeOH)

^{1}H NMR (600 MHz, $CDCl_3$, 298 K): δ 9.39 (s, 1H, H-7), 6.54 (q, *J* = 1.4 Hz, 1H, H-5), 4.18 (q, *J* = 6.9 Hz, 1H, H-3), 3.53 (s, 1H, H-1), 1.95 (d, *J* = 1.4 Hz, 3H, H-8), 1.48 (s, 3H, H-9), 1.46 (s, 3H, H-11), 1.17 (d, *J* = 6.9 Hz, 3H, H-10) ppm

^{13}C NMR (151 MHz, $CDCl_3$, 298 K): δ 195.3 (CH, C-7), 155.1 (CH, C-5), 140.2 (C_q, C-6), 80.4 (C_q, C-4), 77.2 (CH, C-3), 67.8 (C_q, C-2), 66.4 (CH, C-1), 21.0 (CH_3, C-11), 19.0 (CH_3, C-10), 13.9 (CH_3, C-9), 10.8 (CH_3, C-8) ppm

HRMS (EI) *m/z*: $[M+H]^+$ calcd for $C_{11}H_{17}O_3^+$: 197.1172; found 197.1173

Synthesis of Ethyl (2*E*,4*E*)-2,4-dimethyl-5-((1*S*,2*S*,4*R*,5*R*)-2,4,5-trimethyl-3,6-dioxabicyclo[3.1.0]hexan-2-yl)penta-2,4-dienoate (**S8**):

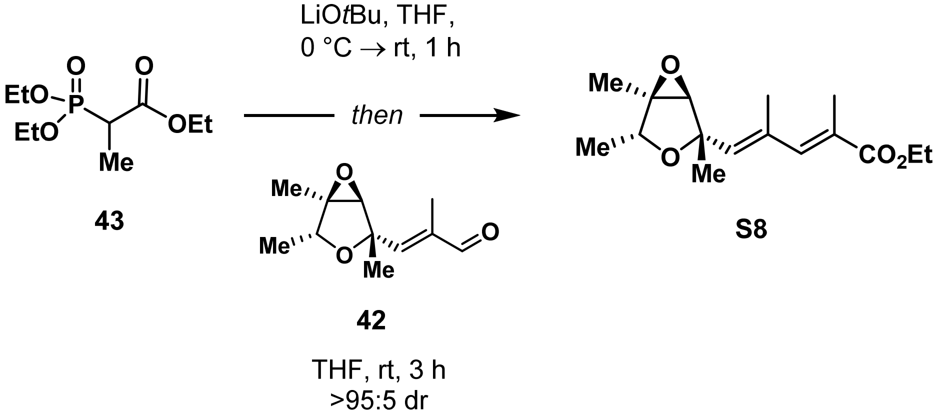

To a solution of triethyl 2-phosphonopropionate (**43**) (353 mg, 1.48 mmol, 1.20 eq) in anhydrous THF (7.4 mL) was added a 1 M LiO*t*-Bu solution in THF (1.48 mL, 1.48 mmol, 1.20 eq) dropwise at 0 °C under argon. The orange solution was allowed to warm to rt over 1 h, before addition of a solution of aldehyde **42** (242 mg, 1.23 mmol, 1.00 eq) in anhydrous THF (3.50 mL) via cannula. The transfer was quantitated with THF (2 x 0.5 mL). The reaction mixture was stirred at rt for 3 h until disappearance of the starting material was confirmed by TLC (30% EtOAc in hexane). After quenching with water (200 mL), the mixture was diluted with EtOAc (200 mL) and the layers were separated. The aqueous layer was extracted with EtOAc (3 × 100 mL) and the combined organic layers were dried with $MgSO_4$, filtered and concentrated to afford the crude ester **S8** as an orange oil, which was pure enough to be used in the next step without further purification. ^{1}H NMR indicated >95:5 *E*/*Z* selectivity for the newly formed double bond.

Yield: 345 mg, quantitative

R*f* = 0.50 (30% EtOAc in hexane)

$[\alpha]_D^{20}$ = −12.0 (*c* = 0.5 in MeOH)

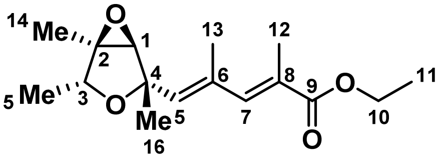

^{1}H NMR (600 MHz, $CDCl_3$, 298 K): δ 7.05 (s, 1H, H-7), 5.65 (s, 1H, H-5), 4.21 (q, *J* = 7.1 Hz, 2H, H-10), 4.14 (q, *J* = 6.9 Hz, 1H, H-3), 3.43 (s, 1H, H-1), 2.02 (d, *J* = 1.5 Hz, 3H, H-12/13), 1.99 (d,

J = 1.6 Hz, 3H, H-12/13), 1.47 (s, 3H, H-16), 1.43 (s, 3H, H-14), 1.31 (t, J = 7.1 Hz, 3H, H-11) 1.19 (d, J = 6.9 Hz, 3H, H-15) ppm

^{13}C NMR (101 MHz, $CDCl_3$, 298 K): δ 169.0, 142.7, 136.5, 134.9, 127.0, 80.1, 77.0, 67.6, 67.4, 60.9, 21.9, 19.0, 18.1, 14.5, 14.1, 13.9 ppm

Synthesis of (2E,4E)-2,4-dimethyl-5-((1S,2S,4R,5R)-2,4,5-trimethyl-3,6-dioxabicyclo[3.1.0]hexan-2-yl)penta-2,4-dien-1-ol (**44**):

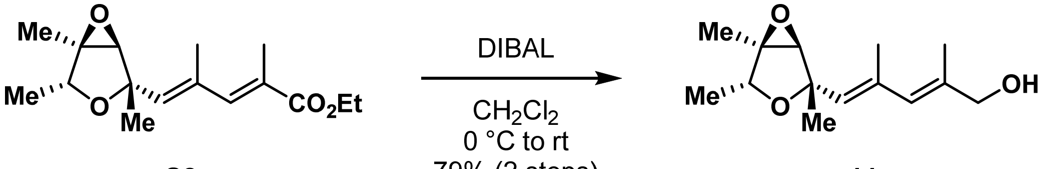

To a solution of **S8** (345 mg, 1.23 mmol, 1.00 eq) in anhydrous CH_2Cl_2 (6.1 mL) was added a 1 M solution of DIBAL in THF (4.28 mL, 4.28 mmol, 3.50 eq) dropwise at 0 °C under argon. The mixture was stirred for 3 h at this temperature, before quenching by slow addition of methanol (4.5 mL). The resulting white slurry was diluted with CH_2Cl_2 (200 mL), before warming to ambient temperature and treating with saturated sodium potassium tartrate solution (250 mL) and water (100 mL). The mixture was stirred vigorously for 30 min in an Erlenmeyer flask, until phase separation was observed. The phases were separated and the aqueous layer was extracted with CH_2Cl_2 (3 × 100 mL). The combined organic phases were dried with $MgSO_4$, filtered and concentrated under reduced pressure. The oily residue was purified by column chromatography (SiO_2, 20% ethyl acetate in hexane) to afford the desired allylic alcohol **44** as a colorless oil.

Yield: 230 mg, 0.97 mmol, 78% over two steps.

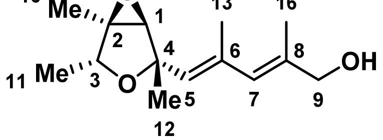

R_f = 0.31 (40% EtOAc in hexane)

$[\alpha]_D^{20}$ = −21.2 (c = 0.5 in MeOH)

^{1}H NMR (400 MHz, $CDCl_3$, 298 K): δ 5.86 (br s, 1H, H-7), 5.44 (br s, 1H, H-5), 4.13 (q, J = 6.8 Hz, 1H, H-3), 4.05 (s, 2H, H-9), 3.42 (s, 1H, H-1), 1.93 (d, J = 1.1 Hz, 3H, H-13), 1.81 (d, J = 1.2 Hz, 3H, H-16), 1.47 (s, 3H, H-10), 1.42 (s, 3H, H-12), 1.20 (d, J = 6.8 Hz, 3H, H-11) ppm

^{13}C NMR (101 MHz, $CDCl_3$, 298 K): δ 135.6 (C_q, C-8), 135.2 (C_q, C-6), 132.6 (CH, C-5), 129.5 (CH, C-7), 80.3 (C_q, C-4), 77.4 (CH, C-3), 69.3 (CH_2, C-9), 67.7 (CH, C-1), 67.7 (C_q, C-2), 22.2 (CH_3, C-12), 19.1 (CH_3, C-11), 18.8 (CH_3, C-13), 15.5 (CH_3, C-16), 14.0 (CH_3, C-10) ppm

HRMS (ESI) *m/z*: $[M+Na]^+$ calcd for $C_{14}H_{22}NaO_3^+$: 261.1461; found 261.1463

Synthesis of (2*E*,4*E*)-2,4-dimethyl-5-((1*S*,2*S*,4*R*,5*R*)-2,4,5-trimethyl-3,6-dioxabicyclo[3.1.0]hexan-2-yl)penta-2,4-dienal; (−)-verrucosal (**45**):[8]

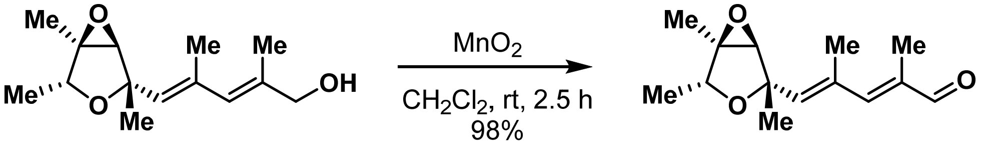

44 **45**

To a solution of alcohol **44** (130 mg, 0.55 mmol, 1.00 eq) in CH_2Cl_2 (5.50 mL) under argon was added MnO_2 (1.19 g, 13.6 mmol, 25.0 eq) and the resulting black mixture was stirred for 2.5 h at rt, while monitoring by TLC (20% ethyl acetate in hexane). The mixture was filtered through a Celite® pad on a glass frit and rinsed with CH_2Cl_2 (300 mL), followed by concentration of the filtrate under reduced pressure. The resulting colorless oil ((−)-verrucosal (**45**))[8] was of sufficient purity to be used in the next step without chromatography.

Yield: 126 mg, 0.53 mmol, 98%

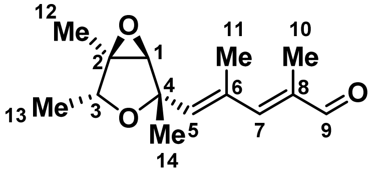

R*f* = 0.30 (20% EtOAc in hexane)

$[\alpha]_D^{20}$ = −15.4 (*c* = 0.5 in MeOH).

^{1}H NMR (400 MHz, $CDCl_3$, 298 K): δ 9.41 (s, 1H, H-9), 6.68 (br s, 1H, H-7), 5.90 (br s, 1H, H-5), 4.15 (q, *J* = 6.8 Hz, 1H, H-3), 3.45 (s, 1H, H-1), 2.16 (d, *J* = 1.3 Hz, 3H, H-11), 1.95 (d, *J* = 1.2 Hz, 3H, H-10), 1.48 (s, 3H, H-12), 1.45 (s, 3H, H-14), 1.19 (d, *J* = 6.8 Hz, 3H, H-13) ppm

^{13}C NMR (101 MHz, $CDCl_3$, 298 K): δ 196.1 (CH, C-9), 154.5 (CH, C-7), 140.5 (CH, C-5), 137.2 (C_q, C-8), 135.5 (C_q, C-6), 80.1 (C_q, C-4), 77.1 (CH, C-3), 67.7 (C_q, C-2), 67.3 (CH, C-1), 22.7 (CH_3, C-14), 19.0 (CH_3, C-13), 17.9 (CH_3, C-11), 13.9 (CH_3, C-12), 11.0 (CH_3, C-10) ppm

Synthesis of (1*R*,2*R*,4*S*,5*S*)-4-((1E,3E,5*Z*)-6-iodo-2,4-dimethylhepta-1,3,5-trien-1-yl)-1,2,4-trimethyl-3,6-dioxabicyclo[3.1.0]hexane (12):

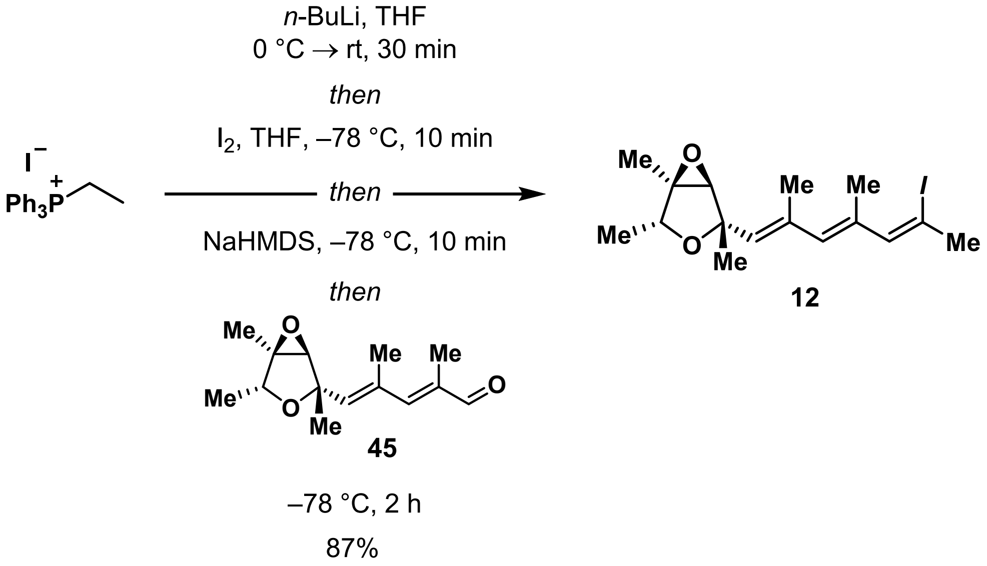

To a suspension of ethyl triphenylphosphonium iodide (177 mg, 0.42 mmol, 4.0 eq) in anhydrous THF (2.8 mL) was added a 2.3 M solution of *n*-BuLi in hexane (0.18 mL, 0.42 mmol, 4.0 eq) dropwise at 0 °C under argon, and the resulting orange solution was allowed to warm to rt over 30 min. This solution was added dropwise to a freshly prepared solution of iodine (107 mg, 0.42 mmol, 4.0 eq) in anhydrous THF (2.8 mL) via cannulation at –78 °C. The resulting beige slurry was stirred for 10 min, before addition of a 1 M NaHMDS solution in THF (0.40 mL, 0.40 mmol, 3.8 eq). After 10 min, a solution of verrucosal (**45**) (25.0 mg, 0.11 mmol, 1.0 eq) in anhydrous THF (0.6 mL) was added to the orange suspension via cannulation, followed by rinsing with THF (2 x 0.1 mL). The resulting yellow mixture was stirred for 2 h at –78 °C. Once complete conversion of the aldehyde was indicated by TLC (20% ethyl acetate in hexane), the mixture was diluted with hexane (20 mL) and stirred vigorously while warming to ambient temperature. The yellow mixture was filtered through a Celite® pad and rinsed with hexane (120 mL). The filtrate was concentrated in vacuo and taken up in toluene and the filtration step was repeated, followed by concentration to afford iodide **12** as a yellow oil. Iodide **12** sometimes spontaneously underwent *E/Z* isomerization and was, therefore, best used immediately in the next step without further purification. *Z/E* ratios of this Stork–Zhao olefination were typically >95:5. The *E*,*E*,*Z*-configuration of **12** was confirmed by 1D nOe measurements (see NMR spectra section).

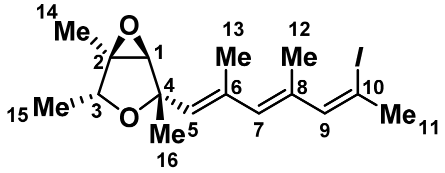

Yield: 34.5 mg, 92.2 µmol, 87%

$\mathbf{R}_f$ = 0.57 (20% EtOAc in hexane)

^{1}H NMR (600 MHz, $CDCl_3$, 298 K): δ 6.01 (br s, 1H, H-9), 5.87 (br s, 1H, H-7), 5.49 (br s, 1H, H-5), 4.14 (q, J = 6.9 Hz, 1H, H-3), 3.45 (s, 1H, H-1), 2.58 (d, J = 1.5 Hz, 3H, H-11), 1.94 (d, J = 1.8 Hz, 3H, H-13), 1.90 (d, J = 1.5 Hz, 3H, H-12), 1.48 (s, 3H, H-14), 1.43 (s, 3H, H-16), 1.21 (d, J = 6.9 Hz, 3H, H-15) ppm

^{13}C NMR (151 MHz, $CDCl_3$, 298 K): δ 138.5 (CH, C-9), 135.3 (CH, C-7), 135.1 (C_q, C-8), 134.4 (C_q, C-6), 133.0 (CH, C-5), 97.8 (C_q, C-10), 80.4 (C_q, C-12), 76.9 (CH, C-3), 67.7 (C_q, C-2), 67.6 (CH, C-1), 35.2 (CH_3, C-11), 22.1 (CH_3, C-16), 19.1 (CH_3, C-15), 18.7 (CH_3, C-13), 17.9 (CH_3, C-12), 14.1 (CH_3, C-14) ppm (data obtained from a partially isomerized sample)

HRMS (ESI) *m/z*: $[M+Na]^+$ calcd for $C_{16}H_{23}INaO_2^+$: 397.0635; found: 397.0643

2.3 Cross-coupling and 8π/6π electrocyclization cascade

Synthesis of (4*R*,5*S*,6*E*,8*Z*,10*Z*,12*E*,14*E*)-5-hydroxy-2,4,6,8,10,12,14-heptamethyl-15-((1*S*,2*S*,4*R*,5*R*)-2,4,5-trimethyl-3,6-dioxabicyclo[3.1.0]hexan-2-yl)pentadeca-6,8,10,12,14-pentaen-3-one (9):

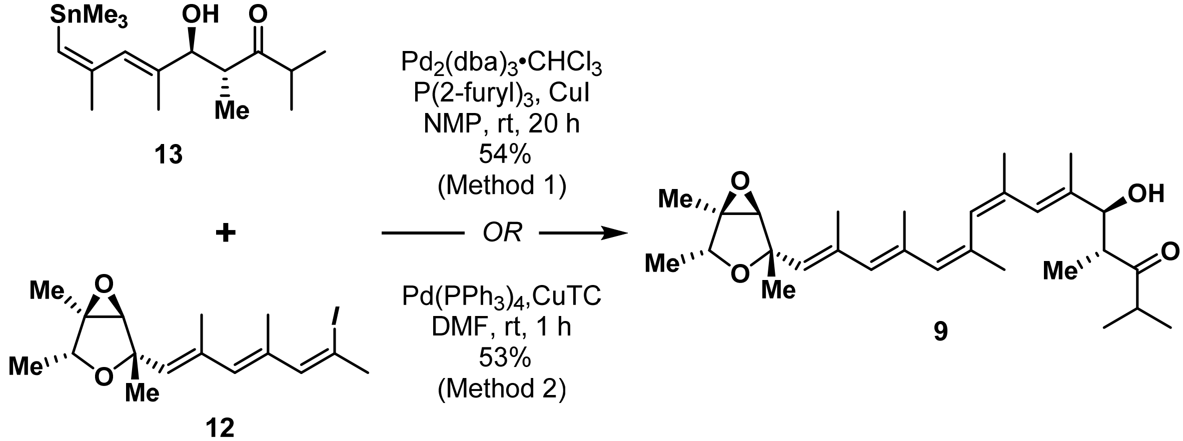

Stille (Method 1): A solution of P(2-furyl)$_3$ (9.3 mg, 40.2 µmol, 0.32 eq) and $Pd_2(dba)_3$•$CHCl_3$ (10.4 mg, 10.1 µmol, 0.08 eq) in anhydrous, freshly distilled (from CaH_2) NMP (1 mL) was sparged with argon for 45 min. In a separate flask wrapped in aluminum foil, vinyl iodide **12** (47.0 mg, 0.13 mmol, 1.00 eq) and vinyl stannane **13** (70.3 mg, 0.18 mmol, 1.50 eq) were dissolved

in anhydrous NMP (1 mL) and the pale-yellow solution was sparged with argon for 30 min, before addition to the catalyst solution via cannulation, followed by rinsing with NMP (0.5 mL). To the dark mixture was added CuI (50.2 mg, 0.26 mmol, 2.10 eq) and the reaction mixture was stirred at ambient temperature for 18 h under the exclusion of light. More P(2-furyl)$_3$ (4.7 mg, 20.1 µmol, 0.16 eq) and $Pd_2(dba)_3$•$CHCl_3$ (5.2 mg, 5.1 µmol, 0.04 eq) were added and the mixture was stirred for another 2 h, before quenching with water (80 mL) and diluting with EtOAc (80 mL). The biphasic mixture was filtered through a Celite® pad, followed by rinsing with ethyl acetate (200 mL). The layers were separated and the aqueous layer was extracted with EtOAc (3 × 50 mL). The combined organic phases were dried with $MgSO_4$, filtered and concentrated to afford the crude product as a brown oil. Purification via column chromatography (SiO_2, conditioned with 5% Et_3N in hexane; eluent: 20% ethyl acetate in hexane + 0.5% Et_3N) afforded pentaene **9** as a yellow oil.

Yield: 31 mg, 68 µmol, 54%

Liebeskind–Stille (Method 2): A Schlenk tube charged with $Pd(PPh_3)_4$ (13.9 mg, 12 µmol, 0.1 eq) and copper(I) thiophene-2-carboxylate (25.2 mg, 0.13 mmol, 1.1 eq), was evacuated and flushed with argon three times. Vinyl iodide **12** (45 mg, 0.12 mmol, 1 eq) was dried by dissolving in benzene (1 mL) followed by concentration in vacuo, re-dissolved in degassed (sparged with nitrogen) DMF (0.5 mL) and added to the catalyst mixture via cannulation. The transfer was quantitated with DMF (2 x 0.1 mL). Stannane **13** (67 mg, 0.18 mmol, 1.5 eq) was dissolved in degassed DMF (0.75 mL) and added via syringe pump over 45 min. After 15 min, the reaction mixture was diluted with water (5 mL) and EtOAc (5 mL), and filtered over a short pad of Celite®, which was then washed with H_2O (15 mL) and EtOAc (50 mL). The layers were separated and the aqueous phase was extracted with EtOAc (4 x 30 mL). The combined organic extracts were washed with brine (2 x 50 mL), dried with $MgSO_4$ and the solvent was removed under reduced pressure. A silica gel slurry was prepared with 5% Et_3N in *n*-hexane and poured into a column. This was washed with 3 column volumes of 10% EtOAc / 0.5% Et_3N in *n*-hexane. The product was then purified by flash chromatography (10% EtOAc / 0.5% Et_3N in *n*-hexane). Pentaene **9** (29 mg, 63 µmol, 53%) was obtained as a yellow oil.

R_f = 0.40 (20% EtOAc in hexane)

^{1}H NMR (600 MHz, $CDCl_3$, 298 K): δ 6.10 (br s, 1H, H-11), 6.02 (br s, 1H, H-13), 5.78 (br s, 1H, H-9), 5.74 (br s, 1H, H-7), 5.42 (br s, 1H, H-5), 4.14–4.10 (m, 3H, H-3, 15, OH), 3.44 (s, 1H, H-1), 2.96 (dq, J = 8.5, 7.1 Hz, 3H, H-16), 2.74 (sep, J = 6.9 Hz, 3H, H-18), 1.92 (br s, 3H, H-26), 1.89 (br s, 3H, H-23), 1.84 (br s, 3H, H-25), 1.82 (br s, 3H, H-24), 1.67 (s, 3H, H-22), 1.47 (s, 3H, H-27), 1.42 (s, 3H, H-29), 1.20 (d, J = 6.9 Hz, 3H, H-28), 1.10 (dd, J = 11.5, 6.9 Hz, 6H, H-19, 20), 0.95 (d, J = 7.1 Hz, 3H, H-21) ppm

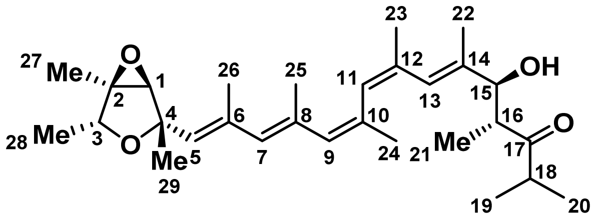

^{13}C NMR (101 MHz, $CDCl_3$, 298 K): δ 219.0 (C_q, C-17), 136.3 (C_q, C-14), 135.7 (C_q, C-6), 134.6 (CH, C-7), 134.4 (C_q, C-8), 134.2 (C_q, C-10), 133.7 (C_q, C-12) 133.3 (CH, C-9), 132.6 (CH, C-5), 130.3 (CH, C-11), 129.6 (CH, C-13), 80.9 (CH, C-15), 80.3 (C_q, C-4), 76.8 (CH, C-3), 67.6 (C_q, C-2), 67.6 (CH, C-1), 47.1 (CH, C-16), 41.6 (CH, C-18), 24.9 (CH_3, C-23), 24.7 (CH_3, C-24), 22.1 (CH_3, C-29), 19.0 (CH_3, C-26), 19.0 (CH_3, C-28), 18.8 (CH_3, C-25), 18.1 (CH_3, C-19/20), 18.0 (CH_3, C-19/20), 14.8 (CH_3, C-21), 14.0 (CH_3, C-27), 12.7 (CH_3, C-22) ppm

HRMS (ESI) *m/z*: $[M+Na]^+$ calcd for $C_{29}H_{44}NaO_4^+$:479.3132; found 479.3131

Synthesis of (1*S*,2*R*)-1-hydroxy-2,4-dimethyl-1-((1*R*,6*S*,7*R*,8*R*)-1,3,5,7-tetramethyl-8-((*E*)-1-((1*S*,2*S*,4*R*,5*R*)-2,4,5-trimethyl-3,6-dioxabicyclo[3.1.0]hexan-2-yl)prop-1-en-2-yl)bicyclo[4.2.0]octa-2,4-dien-7-yl)pentan-3-one (**10**)

and

(1*S*,2*R*)-1-hydroxy-2,4-dimethyl-1-((1*S*,6*R*,7*S*,8*S*)-1,3,5,7-tetramethyl-8-((*E*)-1-((1*S*,2*S*,4*R*,5*R*)-2,4,5-trimethyl-3,6-dioxabicyclo[3.1.0]hexan-2-yl)prop-1-en-2-yl)bicyclo[4.2.0]octa-2,4-dien-7-yl)pentan-3-one (**11**):

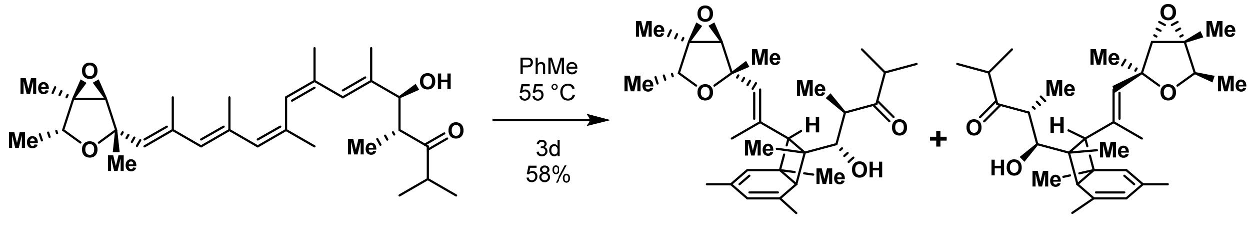

9 **10** 30% **11** 28%

A solution of pentaene **9** (41.0 mg, 89.8 μmol) in anhydrous toluene (5 mL) was sealed in a 20 mL foil-wrapped glass vial under argon and heated to 55 °C for 3 d under argon. After consumption of the starting material was confirmed by TLC (20% EtOAc in hexane) and ^{1}H NMR, the mixture was concentrated. Purification was conducted via preparative TLC (20 cm x 20 cm x 1 mm, plate conditioned with 5% Et_3N in hexane, then run 3 times in 15% EtOAc in hexane). This gave partial separation of the diastereomers **10** and **11** as colorless oils, with mixed fractions making up the bulk of the remaining mass balance.

Compound **10**:

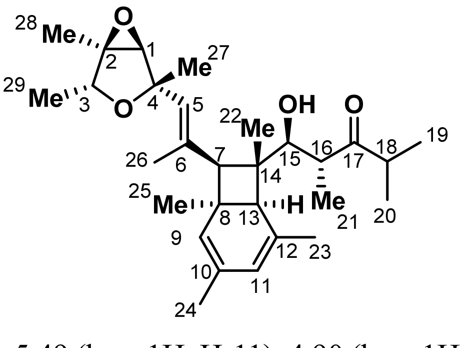

Yield: 12.3 mg, 26.9 mmol, 30%

R*f* = 0.49 (20% EtOAc in hexane)

$[\alpha]^{20}_{578}$ = −15.4 (*c* = 0.6 in MeOH)

¹H NMR (600 MHz, $CDCl_3$, 298 K): δ 5.62 (br s, 1H, H-5), 5.49 (br s, 1H, H-11), 4.90 (br s, 1H, H-9), 4.28 (d, *J* = 8.0 Hz, 3H, OH), 4.11 (q, *J* = 6.9 Hz, 1H, H-3), 3.45 (s, 1H, H-1), 3.21 (dd, *J* = 8.0, 3.0 Hz, 3H, H-15), 2.99 (dq, *J* = 7.2, 3.0 Hz, 3H, H-16), 2.79 (s, 1H, H-7), 2.73 (s, 1H, H-13), 2.69

(sep, J = 6.9 Hz, 3H, H-18), 1.82 (d, J = 1.2 Hz, 3H, H-26), 1.66 (d, J = 1.6 Hz, 3H, H-24), 1.62 (br s, 3H, H-23), 1.48 (s, 3H, H-28), 1.37 (s, 3H, H-27), 1.30 (d, J = 7.2 Hz, 3H, H-21), 1.23 (d, J = 6.9 Hz, 3H, H-29), 1.17 (s, 3H, H-22), 1.13 (d, J = 6.9 Hz, 3H, H-19/20), 1.12 (s, 3H, H-25), 1.06 (d, J = 6.9 Hz, 3H, H-19/20) ppm

^{13}C NMR (151 MHz, $CDCl_3$, 298 K): δ 221.9 (C_q, C-17), 135.7 (C_q, C-6), 132.2 (C_q, C-12), 131.0 (CH, C-5), 129.1 (C_q, C-10), 125.0 (CH, C-9), 124.0 (CH, C-11), 84.2 (CH, C-15), 80.7 (C_q, C-4), 76.6 (CH, C-3), 67.8 (C_q, C-2), 67.3 (CH, C-1), 58.6 (CH, C-7), 50.0 (C_q, C-14), 47.7 (CH, C-13), 42.7 (CH, C-16), 41.9 (C_q, C-8), 41.2 (CH, C-18), 30.9 (CH_3, C-25), 23.9 (CH_3, C-23), 22.5 (CH_3, C-24), 21.9 (CH_3, C-27), 20.3 (CH_3, C-26), 19.3 (CH_3, C-29), 18.7 (CH_3, C-21), 18.6 (CH_3, C-19/20), 18.3 (CH_3, C-19/20), 16.4 (CH_3, C-22), 14.1 (CH_3, C-28) ppm

HRMS (ESI) *m/z*: $[M+Na]^+$ calcd for $C_{29}H_{44}NaO_4^+$: 479.3132; found 479.3130

Compound **11**:

Yield: 11.6 mg, 25.4 µmol, 28%

R_f = 0.50 (20% EtOAc in hexane)

$[\alpha]^{20}_{578}$ = −63.7 (c = 0.4 in MeOH).

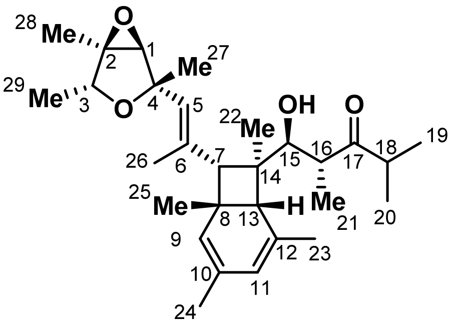

^{1}H NMR (600 MHz, $CDCl_3$, 298 K): δ 5.63 (br s, 1H, H-5), 5.47 (br s, 1H, H-11), 4.86 (br s, 1H, H-9), 4.13 (q, J = 6.9 Hz, 1H, H-3), 3.58 – 3.53 (m, 2H, H-15, OH), 3.39 (s, 1H, H-1), 2. 75 (dq, J = 7.2, 4. 5 Hz, 1H, H-16), 2.69 (sep, J = 6.9 Hz, 3H, H-18), 2.42 (s, 1H, H-7), 2.38 (s, 1H, H-13), 1.90 (d, J = 1.2 Hz, 3H, H-26), 1.70 (br s, 3H, H-23), 1.66 (d, J = 1.6 Hz, 3H, H-24), 1.47 (s, 3H, H-28), 1.37 (s, 3H, H-27), 1.23 (d, J = 6.9 Hz, 3H, H-29), 1.18 (d, J = 6.9 Hz, 3H, H-21), 1.12 (s, 3H, H-25), 1.10 (d, J = 6.9 Hz, 3H, H-19/20), 1.05 (d, J = 6.9 Hz, 3H, H-19/20), 1.03 (s, 3H, H-22) ppm

^{13}C NMR (151 MHz, $CDCl_3$, 298 K): δ 221.4 (C_q, C-17), 135.6 (C_q, C-6), 133.1 (C_q, C-12), 131.7 (CH, C-5), 129.2 (C_q, C-10), 124.7 (CH, C-9), 123.5 (CH, C-11), 87.2 (CH, C-15), 80.3 (C_q, C-4), 76.8 (CH, C-3), 67.7 (CH, C-1), 67.7 (C_q, C-2), 63.7 (CH, C-7), 53.3 (CH, C-13), 49.9 (C_q, C-14),

44.2 (CH, C-16), 42.0 (C_q, C-8), 41.9 (CH, C-18), 32.0 (CH_3, C-25), 23.3 (CH_3, C-24), 22.2 (CH_3, C-23), 22.1 (CH_3, C-27), 20.4 (CH_3, C-26), 19.2 (CH_3, C-29), 18.6 (CH_3, C-19/20), 18.2 (CH_3, C-19/20), 17.0 (CH_3, C-21), 14.0 (CH_3, C-28), 12.6 (CH_3, C-22) ppm

HRMS (ESI) *m/z*: $[M+Na]^+$ calcd for $C_{29}H_{44}NaO_4^+$: 479.3132; found 479.3130

2.4 Synthesis of Emerione A (1)

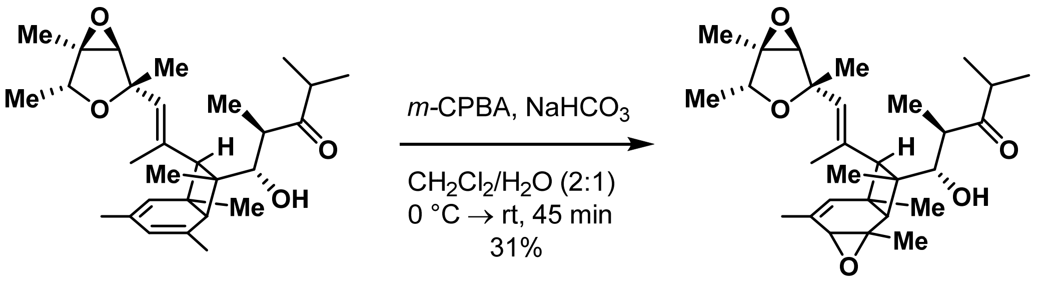

10 (–)-**emerione A** (**1**)

To a solution of **10** (4.0 mg, 8.8 µmol, 1.0 eq) in CH_2Cl_2 (0.9 mL) was added a solution of $NaHCO_3$ (16.2 mg, 193 µmol, 22.0 eq) in water (0.7 mL) at 0 °C. With vigorous stirring, a solution of *m*-CPBA (77%, 2.0 mg, 8.8 µmol, 1.0 eq) in CH_2Cl_2 (0.35 mL) was added dropwise. The reaction mixture was stirred for 10 min at 0 °C. The cooling bath was removed and the reaction mixture was stirred for 45 min (full conversion was observed via TLC) and it was then diluted with a saturated solution of $NaHCO_3$ (10 mL) and CH_2Cl_2 (10 mL). The layers were separated and the aqueous phase was extracted with CH_2Cl_2 (3 x 10 mL). The combined organic extracts were washed with a 1:1 aqueous mixture of saturated $NaHCO_3$ and saturated $Na_2S_2O_3$ (20 mL) and brine (20 mL). After drying with $MgSO_4$ and removal of the solvent under reduced pressure the crude product was purified via column chromatography (SiO_2, conditioned with 5% Et_3N in hexane; eluent: 10% → 20% ethyl acetate in hexane + 0.5% Et_3N) to afford (–)-emerione A as a colorless film.

Yield: 1.3 mg, 2.75 µmol, 31%

$\mathbf{R_f}$ 0.15 (30% EtOAc in *n*-hexane)

$[\alpha]_D^{20}$ = –15.7° (*c* = 0.07 in CH_2Cl_2) [lit: $[\alpha]_D^{25}$ = –7.7° (*c* = 0.13 in CH_2Cl_2)][9]

HRMS (ESI) *m/z*: $[M–H]^-$ calcd for $C_{29}H_{43}O_5^-$: 471.3116; found 471.3114

Reverse phase HPLC purification was performed to obtain an analytically pure sample for NMR. Solvent A = H_2O; Solvent B = MeCN. Method: 1% to 75% B gradient over 1 min; 75% to 90% B gradient over 9 min; 90% to 99% gradient B over 2 min, then hold 99% B for 3 min. Retention time = 8.50 min.

2.5 Table S1: Comparison of synthetic and natural NMR data of emerione A (1)

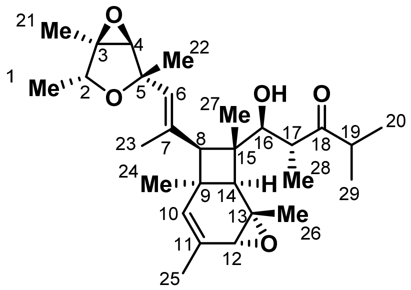

emerione A (1)

#	^{1}H (synthetic)	^{1}H (natural)[9]	Δ	^{13}C (synthetic)	^{13}C (natural)[9]	Δ
1	1.12	1.11	0.01	18.8	18.8	0
2	3.97	3.97	0	75.7	75.7	0
3			0	67.0	67.0	0
4	3.60	3.60	0	66.3	66.3	0
5			0	79.5	79.6	−0.1
6	5.48	5.47	0.01	131.8	131.9	−0.1
7			0	135.2	135.2	0
8	2.68	2.67	0.01	51.4	51.4	0
9			0	39.6	39.7	−0.1
10	5.32	5.32	0	131.3	131.3	0
11			0	129.4	129.4	0
12	2.95	2.94	0.01	58.2	58.2	0
13			0	62.2	62.2	0
14	2.57	2.57	0	43.1	43.1	0
15			0	49.1	49.1	0
16	3.21	3.21	0	77.4	77.5	−0.1
17	2.75	2.75	0	46.5	46.6	−0.1
18			0	216	216.1	−0.1
19	2.70	2.70	0	40.7	40.7	0
20	0.94	0.94	0	18.2	18.2	0
21	1.39	1.38	0.01	13.5	13.5	0
22	1.24	1.23	0.01	21.7	21.7	0
23	1.84	1.83	0.01	19.8	19.9	−0.1
24	1.00	0.99	0.01	30.7	30.7	0
25	1.85	1.84	0.01	22.0	22.0	0

26	1.26	1.25	0.01	21.7	21.7	0
27	1.22	1.22	0	17.1	17.1	0
28	0.95	0.95	0	15.9	15.9	0
29	0.96	0.96	0	17.3	17.3	0

2.6 Synthesis of Emerione B (2)

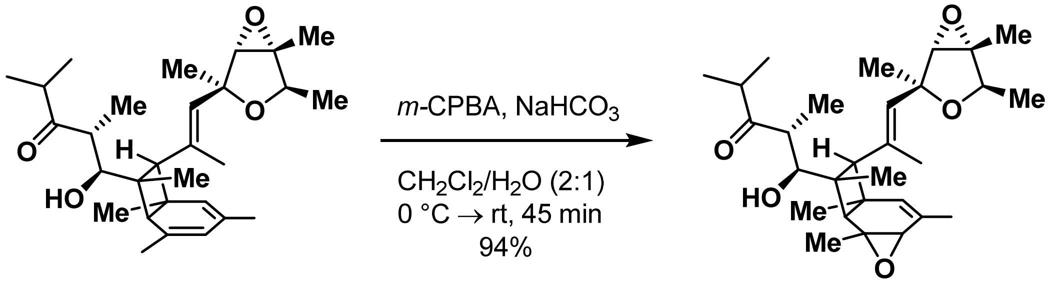

11 (–)-**emerione B** (**2**)

To a solution of **11** (4.3 mg, 9.4 mmol, 1.0 eq) in CH_2Cl_2 (1.0 mL) was added a solution of $NaHCO_3$ (40 mg, 207 mmol, 22.0 eq) in water (0.6 mL). The biphasic mixture was cooled to 0 °C, followed by dropwise addition of a solution of *m*-CPBA (77%, 2.1 mg, 9.4 mmol, 1.0 eq) in CH_2Cl_2 (0.35 mL). After 15 min, the mixture was warmed to rt and stirred vigorously for 30 min. The mixture was diluted with CH_2Cl_2 (10 mL) and saturated aqueous $NaHCO_3$ (10 mL), the layers were separated and the aqueous layer was extracted with CH_2Cl_2 (3 × 10 mL). The combined organic phases were washed with a 1:1 mixture of saturated Na_2SO_3 and saturated $NaHCO_3$ (15 mL) and brine (15 mL), dried ($MgSO_4$), filtered and concentrated. Purification via column chromatography (SiO_2, conditioned with 5% Et_3N in hexane; eluent: 15% ethyl acetate in hexane + 0.5% Et_3N) afforded (–)-emerione B as a colorless solid.

Yield: 4.2 mg, 8.9 µmol, 94%

$\mathbf{R}_{\boldsymbol{f}}$ = 0.50 (40% EtOAc in hexane)

$[\alpha]_D^{20}$ = −10.0 (*c* = 0.12 in CH_2Cl_2) [lit: $[\alpha]_D^{25}$ = –4.9° (*c* = 0.65 in CH_2Cl_2)][9]

HRMS (ESI) *m/z*: $[M–H]^-$ calcd for $C_{29}H_{43}O_5^-$: 471.3116; found 471.7134

Reverse phase HPLC purification using the same gradient as for emerione A was performed to obtain an analytically pure sample for NMR. Retention time = 8.60 min

2.7 Table S2: Comparison of synthetic and natural NMR data of emerione B (**2**)

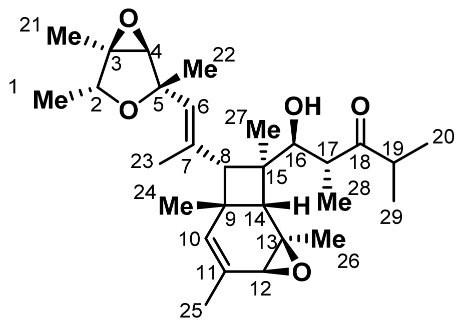

emerione B (2)

#	^{1}H (synthetic)	^{1}H (natural)[9]	Δ	^{13}C (synthetic)	^{13}C (natural)[9]	Δ
1	1.10	1.09	0.01	18.9	18.9	0
2	3.97	3.96	0.01	75.7	75.8	−0.1
3			0	67.0	67.0	0
4	3.56	3.55	0.01	66.5	66.6	−0.1
5			0	79.5	79.5	0
6	5.42	5.41	0.01	130.9	130.9	0
7			0	135.0	135.0	0
8	2.19	2.18	0.01	58.8	58.9	−0.1
9			0	40.4	40.5	−0.1
10	5.17	5.17	0	130.8	130.9	−0.1
11			0	129.2	129.3	−0.1
12	2.90	2.89	0.01	58.8	58.8	0
13			0	62.8	62.8	0
14	2.20	2.20	0	49.3	49.3	0
15			0	48.1	48.1	0
16	3.40	3.39	0.01	84.3	84.3	0
17	2.76	2.76	0	46.1	46.1	0
18			0	217.4	217.5	−0.1
19	2.71	2.7	0.01	40.9	41.0	−0.1
20	0.99	0.99	0	18.0	18.1	−0.1
21	1.37	1.36	0.01	13.5	13.5	0
22	1.20	1.20	0	22.1	22.1	0
23	1.76	1.75	0.01	20.3	20.3	0
24	1.05	1.05	0	31.9	31.8	0.1
25	1.84	1.83	0.01	21.5	21.5	0
26	1.31	1.30	0.01	23.1	23.1	0
27	1.16	1.16	0	12.1	12.1	0
28	0.82	0.81	0.01	13.8	13.7	0.1
29	0.95	0.94	0.01	17.5	17.5	0

2.8 Synthesis of Emerione C (49)

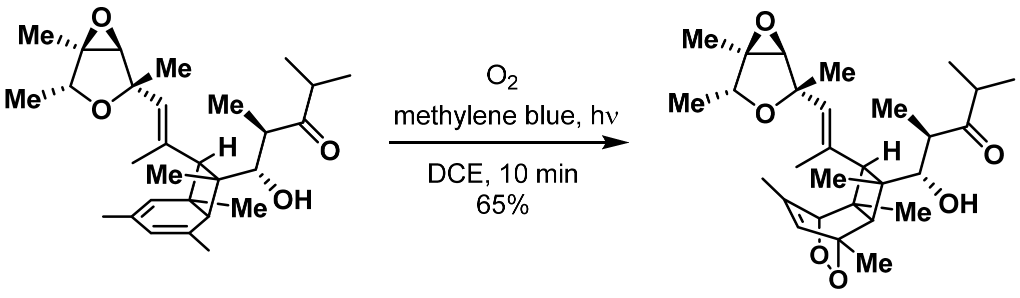

10 (–)-emerione C (49)

In a three-neck flask equipped with a reflux condenser, a solution of **10** (3.0 mg, 6.57 μmol, 1.0 eq) in anhydrous dichloroethane (15 mL) was sparged with O_2 for 10 min. Methylene blue (0.1 mg, 0.31 μmol, 0.03 eq) was added and the mixture was stirred for 10 min under irradiation with a 400 W halogen lamp while sparging with O_2. NOTE: The reaction warms somewhat due to heat produced by the lamp. The mixture was concentrated and taken up in Et_2O (20 mL), followed by filtration through a celite pad and rinsing with Et_2O (100 mL). The solvent was removed under reduced pressure and the resulting orange oil was purified by column chromatography (SiO_2, 25% EtOAc in hexane + 0.5% Et_3N) to afford (–)-emerione C (**49**) as a colorless solid.

Yield: 2.1 mg, 4.30 μmol, 65%

$\mathbf{R}_f$ = 0.54 (40% EtOAc in hexane)

$[\alpha]_D^{20}$ = −27.4 (*c* = 0.14 in CH_2Cl_2) [lit: $[\alpha]_D^{25}$ = –48.5° (*c* = 0.16 in CH_2Cl_2)][9]

HRMS (ESI) *m/z*: $[M+Na]^+$ calcd for $C_{29}H_{44}NaO_6^+$: 511.3030; found 511.3029

Reverse phase HPLC purification was performed to obtain an analytically pure sample for NMR. Solvent A = H_2O; Solvent B = MeCN. Method: 1% to 50% B gradient over 1 min; 50% to 70% B gradient over 14 min; 70% to 99% gradient B over 0.5 min, then hold 99% B for 2.5 min. Retention time = 14.90 min.

2.9 Table S3: Comparison of NMR data for synthetic 49 and natural emerione C

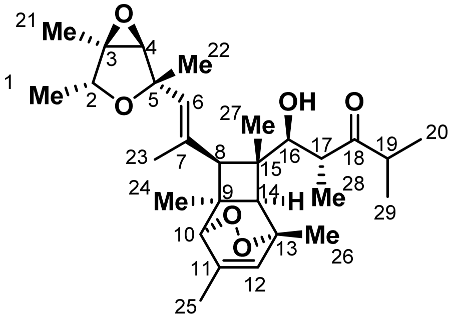

emerione C (49)

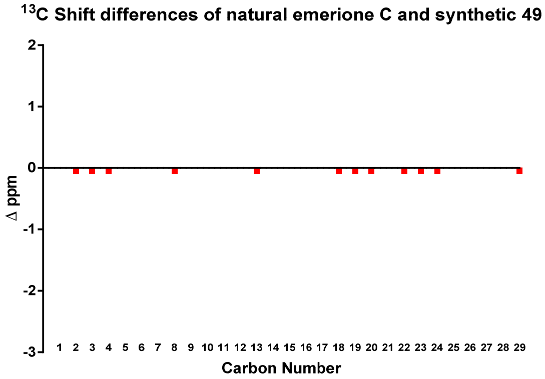

#	^{1}H (synthetic)	^{1}H (natural)[9]	Δ	^{13}C (synthetic)	^{13}C (natural)[9]	Δ
1	1.08	1.08	0	18.9	18.9	0
2	3.93	3.92	0.01	75.3	75.4	−0.1
3			0	67.1	67.2	−0.1
4	3.61	3.61	0	65.8	65.9	−0.1
5			0	80.1	80.1	0
6	5.22	5.22	0	127.8	127.8	0
7			0	135.1	135.1	0
8	2.87	2.86	0.01	58.4	58.5	−0.1
9			0	40.1	40.1	0
10	4.30	4.30	0	80.2	80.2	0
11			0	138.8	138.8	0
12	5.96	5.96	0	129.5	129.5	0
13			0	76.7	76.8	−0.1
14	1.92	1.91	0.01	51.1	51.1	0
15			0	45.3	45.3	0
16	3.46	3.46	0	82.5	82.5	0
17	2.84	2.84	0	46.4	46.4	0
18			0	217.1	217.2	−0.1
19	2.77	2.77	0	40.4	40.5	−0.1
20	0.99	0.99	0	18.2	18.3	−0.1
21	1.36	1.35	0.01	13.6	13.6	0
22	1.17	1.17	0	21.9	22.0	−0.1
23	1.61	1.6	0.01	18.1	18.2	−0.1
24	1.49	1.49	0	28.6	28.7	−0.1
25	1.93	1.93	0	19.1	19.1	0
26	1.27	1.27	0	22.6	22.6	0
27	1.04	1.04	0	13.0	13.0	0
28	1.01	1.00	0.01	15.7	15.7	0
29	0.94	0.94	0	17.7	17.8	−0.1

2.10 Synthesis of Emerione D (50)

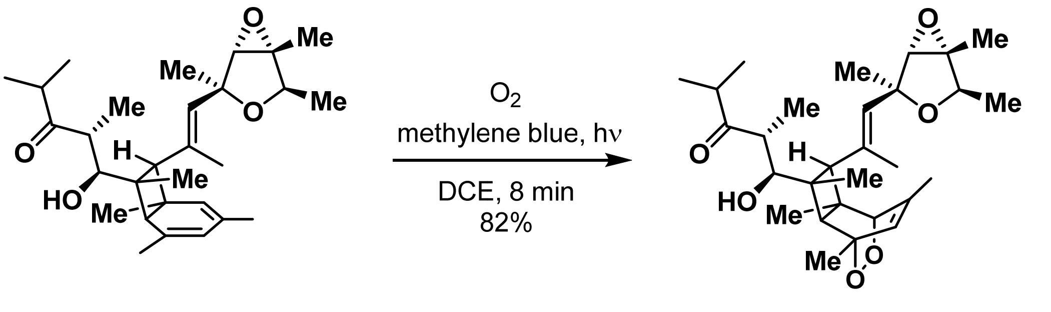

11 (+)-**emerione D** (**50**)

In a three-neck flask equipped with a reflux condenser, a solution of **11** (9.0 mg, 19.7 µmol, 1.0 eq) in dry DCE (15 mL) was sparged with O_2 for 10 min. Methylene blue (0.2 mg, 0.49 µmol, 0.03 eq) was added and the mixture was stirred for 8 min under irradiation with a 400 W halogen lamp while sparging with O_2. NOTE: The reaction warms somewhat due to heat produced by the lamp. The mixture was concentrated and taken up in Et_2O (20 mL), followed by filtration through a celite pad and rinsing with Et_2O (200 mL). The solvent was removed under reduced pressure and the resulting orange oil was purified by column chromatography (SiO_2, 15% EtOAc in hexane + 0.5% Et_3N) to afford (+)-emerione D (**50**) as a colorless solid. Crystals suitable for X-ray crystallography were obtained using vapor diffusion of pentane into a solution of **50** dissolved in a 1:1 mixture of CH_2Cl_2/Et_2O.

Yield: 7.9 mg, 16.2 µmol, 82%

$\mathbf{R}_f$ = 0.49 (40% EtOAc in hexane)

$[\alpha]_D^{20}$ = +29.6 (c = 0.25 in CH_2Cl_2)

Reverse phase HPLC purification using the same gradient as for emerione C was performed to obtain an analytically pure sample for NMR. Retention time = 13.7 min.

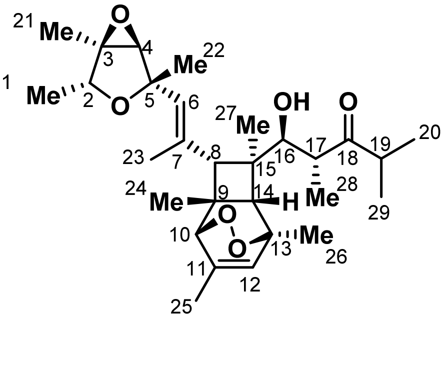

^{1}H NMR (600 MHz, DMSO-d_6, 298 K): δ 6.01 (br s, 1H, H-6), 5.45 (br s, 1H, H-12), 5.21 (d, J = 6.5 Hz 1H, OH), 4.15 (d, J = 1.8 Hz, 1H, H-10), 3.96 (q, J = 6.8 Hz, 1H, H-2), 3.55 – 3.52 (m, 2H, H-4, 16), 2.82 (dq, J = 9.7, 7.1 Hz, 1H, H-17), 2.72 (sep, J = 6.9 Hz, 3H, H-19), 2.63 (s, 1H, H-8), 2.13 (s, 1H, H-14), 1.90 (d, J = 1.7 Hz, 3H, H-25),

1.60 (br s, 3H, H-23), 1.51 (s, 3H, H-24), 1.31 (s, 3H, H-21), 1.29 (s, 3H, H-22), 1.14 (s, 3H, H-26), 1.10 (s, 3H, H-27), 1.07 (d, J = 6.8 Hz, 3H, H-1), 0.99 (d, J = 6.9 Hz, 3H, H-20), 0.97 (d, J = 6.9 Hz, 3H, H-29), 0.87 (d, J = 7.1 Hz, 3H, H-28) ppm

^{13}C-NMR (151 MHz, DMSO-d_6, 298 K): δ 216.9 (C_q, C-18), 138.9 (C_q, C-11), 135.1 (C_q, C-7), 129.3 (CH, C-6), 129.2 (CH, C-12), 81.3 (CH, C-16), 80.5 (C_q, C-5), 79.9 (CH, C-10), 76.5 (C_q, C-13), 75.5 (CH, C-2), 67.4 (C_q, C-3), 65.8 (CH, C-4), 56.8 (CH, C-8), 52.1 (CH, C-14), 46.7 (CH, C-17), 44.5 (C_q, C-15), 39.2 (C_q, C-9), 28.7 (CH_3, C-24), 22.4 (CH_3, C-22), 21.5 (CH_3, C-26), 19.5 (CH_3, C-25), 19.2 (CH_3, C-23), 19.0 (CH_3, C-1), 18.0 (CH_3, C-20), 17.5 (CH_3, C-29), 13.5 (CH_3, C-21), 13.0 (CH_3, C-28), 12.2 (CH_3, C-27) ppm

HRMS (ESI) *m/z*: $[M–H]^-$ calcd for $C_{29}H_{43}O_6^-$: 487.3065; found 487.3063

Emerione D has the structure that was originally proposed for emerione C. The table below compares our data for emerione D with the reported data for natural emerione C. Taken together with Table S3 and the X-ray crystallography data for emerione D, the structure of emerione C is unambiguously defined.

2.11 Table S4: Comparison of NMR data for synthetic 50 and natural emerione C (49)

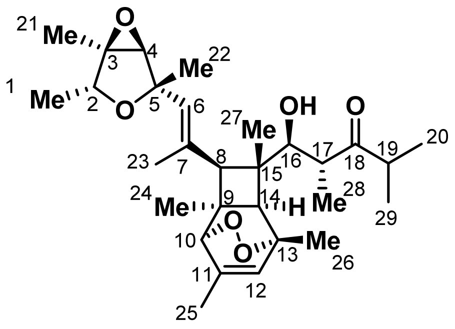

emerione D (50)

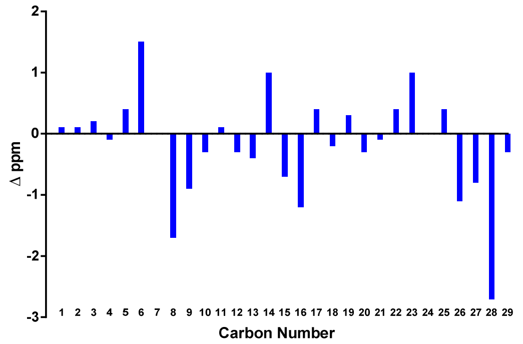

#	^{1}H (synthetic)	^{1}H (natural)[9]	Δ	^{13}C (synthetic)	^{13}C (natural)[9]	Δ
1	1.07	1.08	−0.01	19.0	18.9	0.1
2	3.96	3.92	0.04	75.5	75.4	0.1
3			0	67.4	67.2	0.2
4	3.52	3.61	−0.09	65.8	65.9	−0.1
5			0	80.5	80.1	0.4
6	6.01	5.22	0.79	129.3	127.8	1.5
7			0	135.1	135.1	0
8	2.63	2.86	−0.23	56.8	58.5	−1.7
9			0	39.2	40.1	−0.9
10	4.15	4.30	−0.15	79.9	80.2	−0.3
11			0	138.9	138.8	0.1
12	5.45	5.96	−0.51	129.2	129.5	−0.3
13			0	76.5	76.8	−0.3
14	2.12	1.91	0.21	52.1	51.1	1
15			0	44.6	45.3	−0.7
16	3.54	3.46	0.08	81.3	82.5	−1.2
17	2.82	2.84	−0.02	46.8	46.4	0.4
18			0	217.0	217.2	−0.2
19	2.72	2.77	−0.05	40.8	40.5	0.3
20	0.99	0.99	0	18.0	18.3	−0.3
21	1.31	1.35	−0.04	13.5	13.6	−0.1
22	1.28	1.17	0.11	22.4	22.0	0.4
23	1.60	1.60	0	19.2	18.2	1
24	1.51	1.49	0.02	28.7	28.7	0
25	1.89	1.93	−0.04	19.5	19.1	0.4
26	1.14	1.27	−0.13	21.5	22.6	−1.1
27	1.10	1.04	0.06	12.2	13.0	−0.8
28	0.88	1.00	−0.12	13.0	15.7	−2.7
29	0.96	0.94	0.02	17.5	17.8	−0.3

2.12 X-ray Crystallographic Reports

2.12.1 Crystallographic Analysis of 25

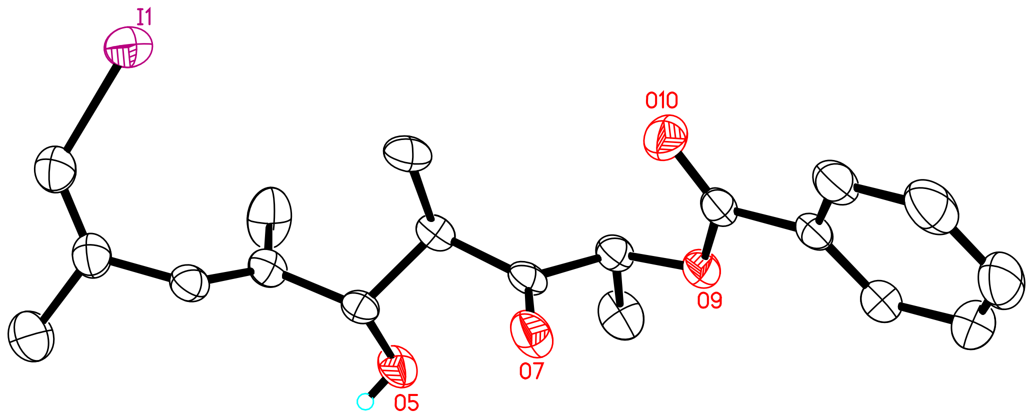

Figure S1: Rendering of **25**

25 crystallized in the orthorhombic space group $P2_12_12_1$, with four molecules per unit cell. T=200(2) K, $Theta_{max}$= 29.231 deg, radiation MoKα, lambda=0.71073 Å, 0.5 deg omega-scans with CCD area detector, covering the asymmetric unit in reciprocal space with a mean redundancy of 7.47 and a completeness of 95.2% to a resolution of 0.73 Å. Intensities were corrected for Lorentz and polarization effects, an empirical scaling and absorption correction was applied using SADABS[10] based on the Laue symmetry of the reciprocal space, mu=1.63mm^{-1}, T_{min}=0.85, T_{max}=0.93, structure solved with SHELXS-97 (Sheldrick 2008)[11] and refined against F^2 with a Full-matrix least-squares algorithm using the SHELXL-2018/3 (Sheldrick, 2018) software[12], 225 parameters refined, hydrogen atoms were treated using appropriate riding models, except H5 of the hydroxy group, which was refined isotropically. Absolute configuration was determined by anomalous dispersion (Flack -0.014(7)).

2.12.2 Table S5: Crystal data and structural refinement for 25

Identification code	dai2
Empirical formula	$C_{19}H_{23}IO_4$
Formula weight	442.27
Temperature	200(2) K
Wavelength	0.71073 Å
Crystal system	orthorhombic
Space group	$P2_12_12_1$
Z	4

Unit cell dimensions	a = 6.0220(2) Å	α = 90 deg.
	b = 13.5278(5) Å	β = 90 deg.
	c = 24.2967(9) Å	γ = 90 deg.
Volume	1979.32(12) $Å^3$	
Density (calculated)	1.48 g/cm^3	
Absorption coefficient	1.63 mm^{-1}	
Crystal shape	plank	
Crystal size	0.152 x 0.126 x 0.060 mm^3	
Crystal colour	colourless	
Theta range for data collection	1.7 to 29.2 deg.	
Index ranges	$-8 \le h \le 8$, $-18 \le k \le 18$, $-31 \le l \le 32$	
Reflections collected	23180	
Independent reflections	4996 (R(int) = 0.0275)	
Observed reflections	4349 ($I > 2\sigma(I)$)	
Absorption correction	Semi-empirical from equivalents	
Max. and min. transmission	0.93 and 0.85	
Refinement method	Full-matrix least-squares on F^2	
Data/restraints/parameters	4996 / 0 / 225	
Goodness-of-fit on F^2	1.02	
Final R indices (I>2sigma(I))	R1 = 0.032, wR2 = 0.072	
Absolute structure parameter	-0.014(7)	
Largest diff. peak and hole	1.08 and -0.88 $eÅ^{-3}$	

2.12.3 Crystallographic Analysis of 36

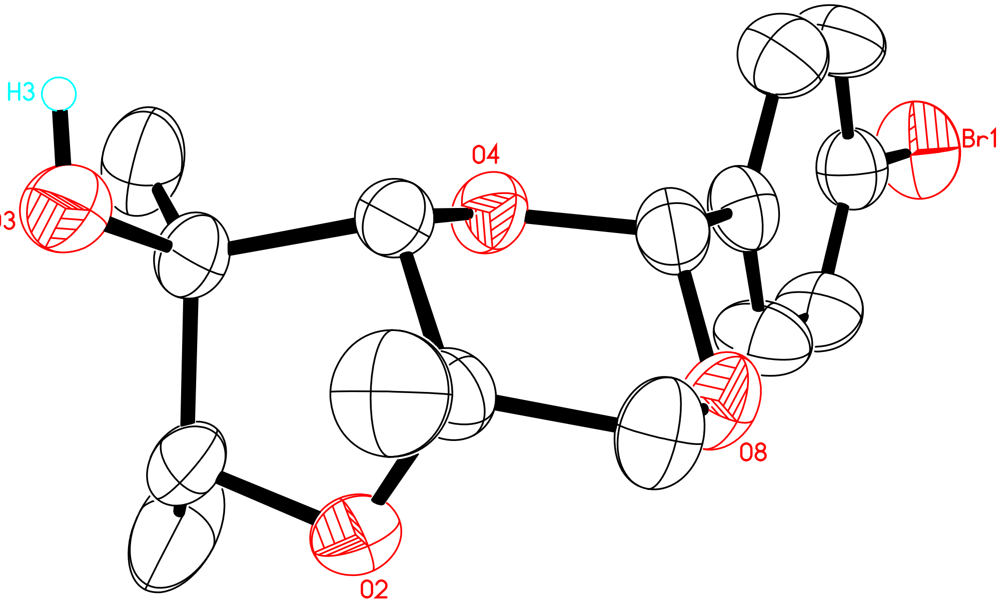

Figure S2: Rendering of **36**

Compound **36** crystallized in the orthorhombic space group $P2_12_12_1$, with four molecules per unit cell. T=200(2) K, $Theta_{max}$= 72.010 deg, radiation CuKα, lambda=1.54178 Å, 0.5 deg omega-scans with CCD area detector, covering the asymmetric unit in reciprocal space with a mean redundancy of 3.28 and a completeness of 96.0% to a resolution of 0.81 Å. Intensities were corrected for Lorentz and polarization effects, an empirical scaling and absorption correction was applied using X-Area LANA 1.70.0.0 (STOE, 2017) based on the Laue symmetry of the reciprocal space, mu=3.85mm^{-1}, T_{min}=0.40, T_{max}=1.72, structure solved with SHELXT-2014 (Sheldrick 2014)[11] and refined against F^2 with a Full-matrix least-squares algorithm using the SHELXL-2018/3 (Sheldrick, 2018) software[12], 188 parameters refined, hydrogen atoms were treated using appropriate riding models, except H3 of the hydroxyl group, which was refined isotropically. Absolute configuration was determined by anomalous dispersion (Flack -0.01(3)).

2.12.4 Table S6: Crystal data and structural refinement for 36

Identification code	ami20	
Empirical formula	$C_{15}H_{19}BrO_4$	
Formula weight	343.21	
Temperature	200(2) K	
Wavelength	1.54178 Å	
Crystal system	orthorhombic	
Space group	$P2_12_12_1$	
Z	4	
Unit cell dimensions	a = 5.9328(3) Å	α = 90 deg.
	b = 11.7150(5) Å	β = 90 deg.
	c = 21.4736(15) Å	γ = 90 deg.
Volume	1492.47(14) Å^3	
Density (calculated)	1.53 g/cm^3	
Absorption coefficient	3.85 mm^{-1}	
Crystal shape	needle	
Crystal size	0.250 x 0.017 x 0.012 mm^3	
Crystal colour	colourless	
Theta range for data collection	4.3 to 72.0 deg.	
Index ranges	$-2\leq h\leq 7$, $-14\leq k\leq 13$, $-20\leq l\leq 26$	
Reflections collected	5734	
Independent reflections	2700 (R(int) = 0.0521)	
Observed reflections	1773 ($I > 2\sigma(I)$)	
Absorption correction	Semi-empirical from equivalents	
Max. and min. transmission	1.72 and 0.40	
Refinement method	Full-matrix least-squares on F^2	
Data/restraints/parameters	2700 / 0 / 188	
Goodness-of-fit on F^2	1.04	

Final R indices (I>2sigma(I))	R1 = 0.048, wR2 = 0.090
Absolute structure parameter	-0.01(3)
Largest diff. peak and hole	0.33 and -0.45 eÅ^{-3}

2.12.5 Crystallographic Analysis of 50

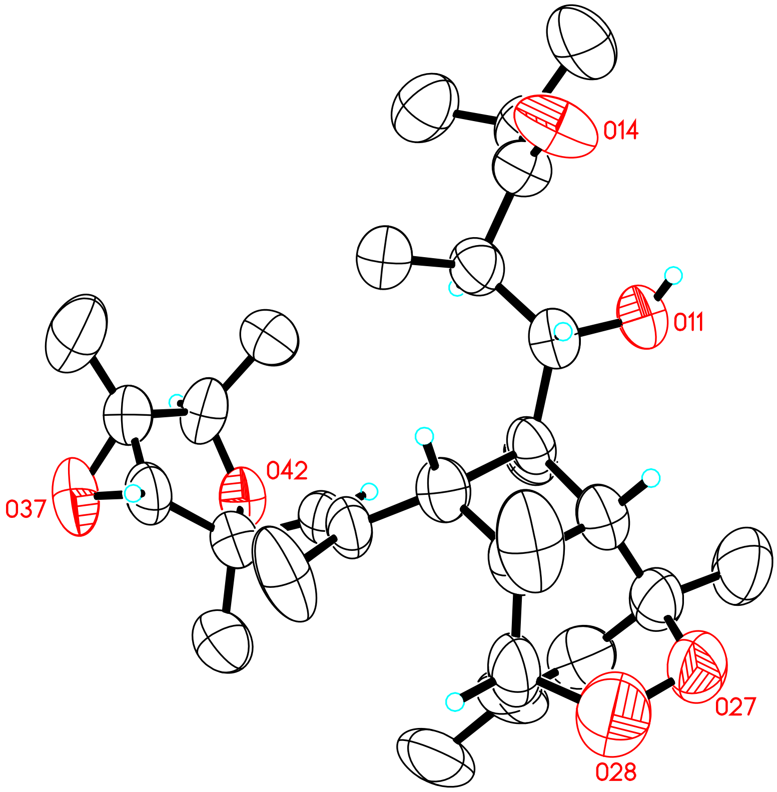

Figure S3: Rendering of **50**

Emerione D (**50**) crystallized in the orthorhombic space group $P2_12_12_1$ with four molecules per unit cell. T=200(2) K, Theta$_{max}$= 56.908 deg, radiation CuKα, lambda=1.54178 Å, 0.5 deg omega-scans with CCD area detector, covering the asymmetric unit in reciprocal space with a mean redundancy of 6.52 and a completeness of 99.4% to a resolution of 0.92 Å. Intensities were corrected for Lorentz and polarization effects, an empirical scaling and absorption correction was applied using X-Area LANA 1.83.8.0 (STOE, 2020) based on the Laue symmetry of the reciprocal space, mu=0.65mm^{-1}, T$_{min}$=0.14, T$_{max}$=0.99, structure solved with SHELXT-2014 (Sheldrick 2014)[11] and refined against F^2 with a Full-matrix least-squares algorithm using the SHELXL-2018/3 (Sheldrick, 2018) software[12], 328 parameters refined, hydrogen atoms were treated using appropriate riding models.

2.12.6 Table S7: Crystal data and structural refinement for 50

Identification code	ami23	
Empirical formula	$C_{29}H_{44}O_6$	
Formula weight	488.64	
Temperature	200(2) K	
Wavelength	1.54178 Å	
Crystal system	orthorhombic	
Space group	$P2_12_12_1$	
Z	4	
Unit cell dimensions	a = 10.0250(6) Å	α = 90 deg.
	b = 13.1087(9) Å	β = 90 deg.
	c = 20.9540(19) Å	γ = 90 deg.
Volume	2753.7(4) $Å^3$	
Density (calculated)	1.18 g/cm^3	
Absorption coefficient	0.65 mm^{-1}	
Crystal shape	plank	
Crystal size	0.175 x 0.045 x 0.015 mm^3	
Crystal colour	colourless	
Theta range for data collection	4.2 to 56.9 deg.	
Index ranges	-8≤h≤10, -14≤k≤9, -22≤l≤22	
Reflections collected	13946	
Independent reflections	3672 (R(int) = 0.1006)	
Observed reflections	2224 (I > 2σ(I))	
Absorption correction	Semi-empirical from equivalents	
Max. and min. transmission	0.99 and 0.14	
Refinement method	Full-matrix least-squares on F^2	
Data/restraints/parameters	3672 / 0 / 328	
Goodness-of-fit on F^2	1.06	
Final R indices (I>2sigma(I))	R1 = 0.111, wR2 = 0.273	
Absolute structure parameter	0.0(5)	
Largest diff. peak and hole	0.42 and -0.29 e$Å^{-3}$	

3 DFT calculations

3.1 Methods

Initial conformational searches were completed using the CREST conformer-rotamer ensemble sampling tool,[13] version 2.10.2 with xtb version 6.4.1.[14] Geometry optimizations, frequency analyses were calculated at the theoretical M06-2X/Def2-SVP level[15,16] using the Gaussian 09 package[17] with default convergence criteria. M06-2X functional gives refined energies for organic systems.[18]

Frequency outcomes were examined to confirm stationary points as minima (no imaginary frequencies) or transition states (only one imaginary frequency). Single point energies at the SMD(toluene)-M06-2X/Def2-TZVP [15,16,19] level of theory were computed. Paton's GoodVibes[20] was used to correct entropy and enthalpy by Grimme's quasi-harmonic approximation[21] and Head-Gordon's method.[22] 3D structures of molecules were generated by CYL view.[23] All energies are in kcal/mol if not labelled otherwise. All bond lengths are in Angstroms (Å).

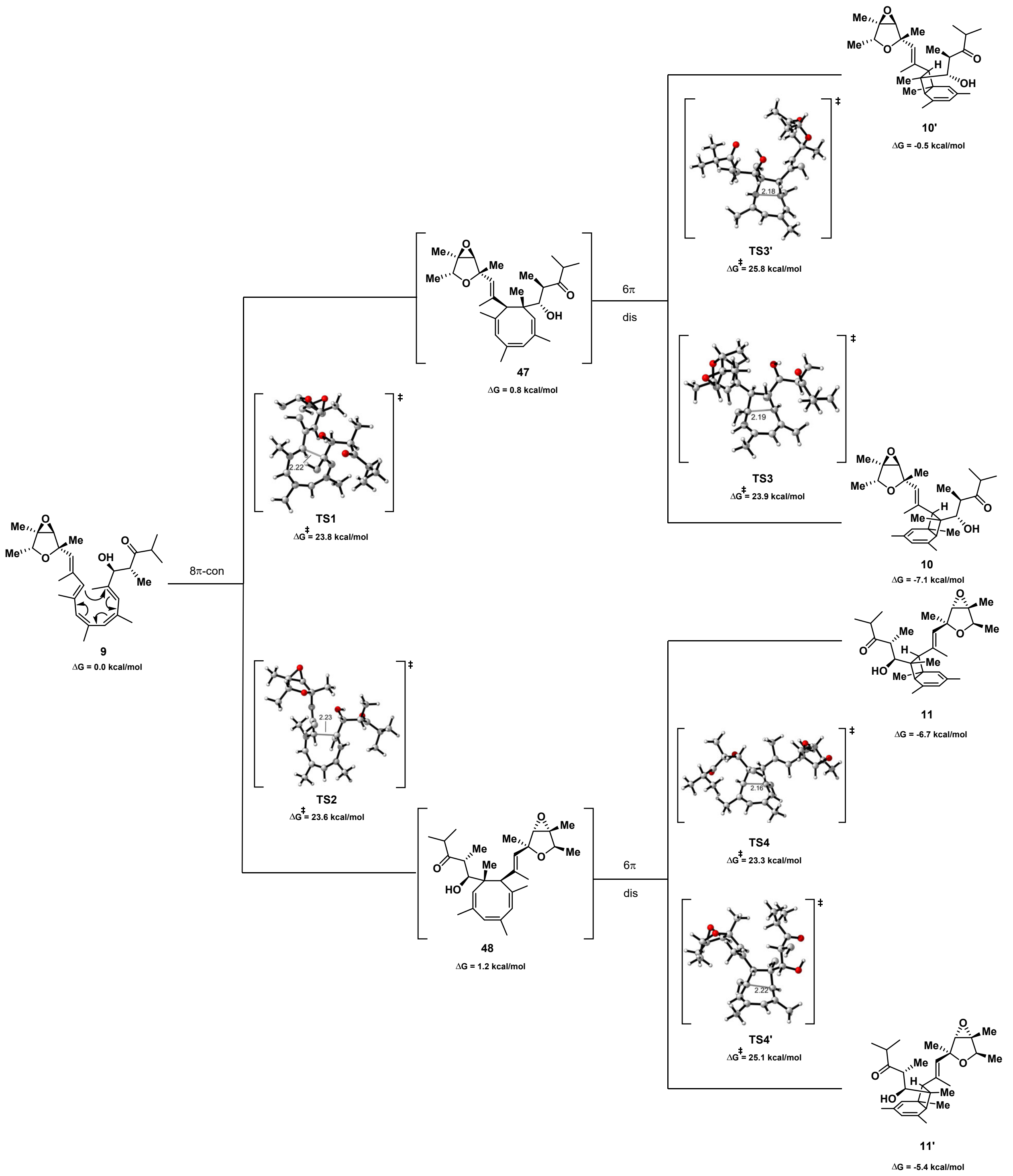

3.2 Figure S4: Calculated energy levels of the electrocylization cascade

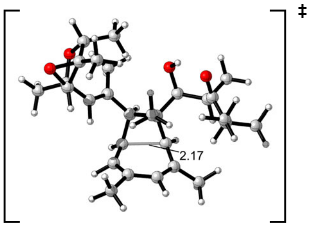

TS5

4.4 kcal/mol

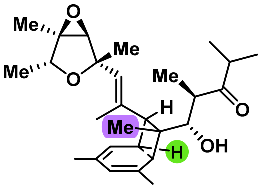

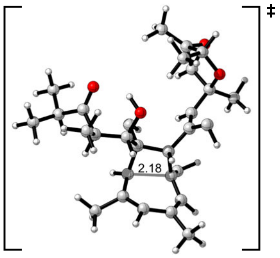

TS5'

0.8 kcal/mol

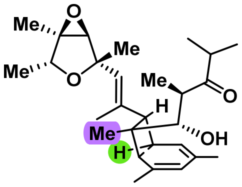

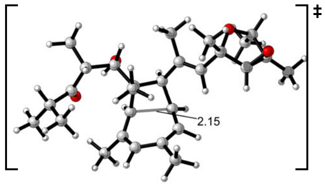

TS6

4.2 kcal/mol

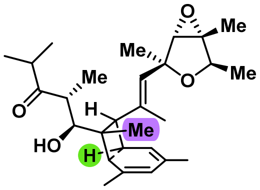

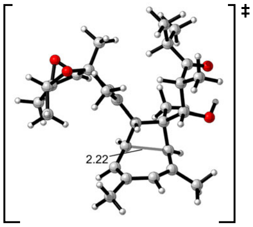

TS6'

0.0 kcal/mol

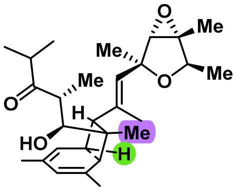

3.3 Figure S5: DFT-calculations after replacing the green methyl with a hydrogen

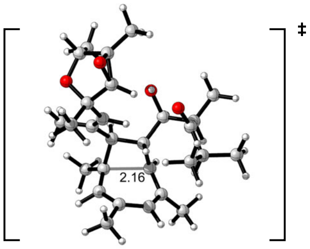

TS7

0.0 kcal/mol

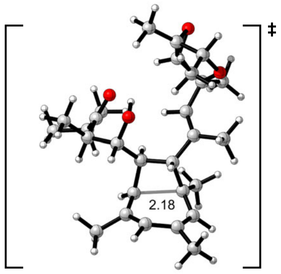

TS7'

1.0 kcal/mol

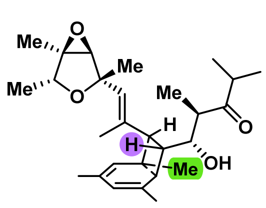

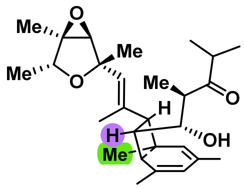

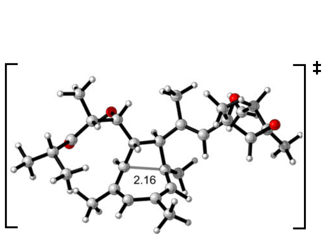

TS8

2.8 kcal/mol

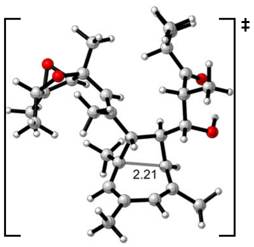

TS8'

6.1 kcal/mol

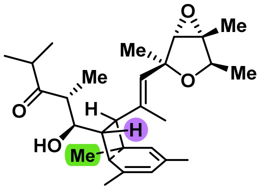

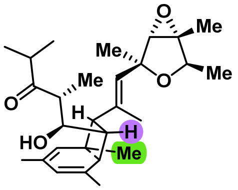

3.4 Figure S6: DFT-calculations after replacing the purple methyl with a hydrogen

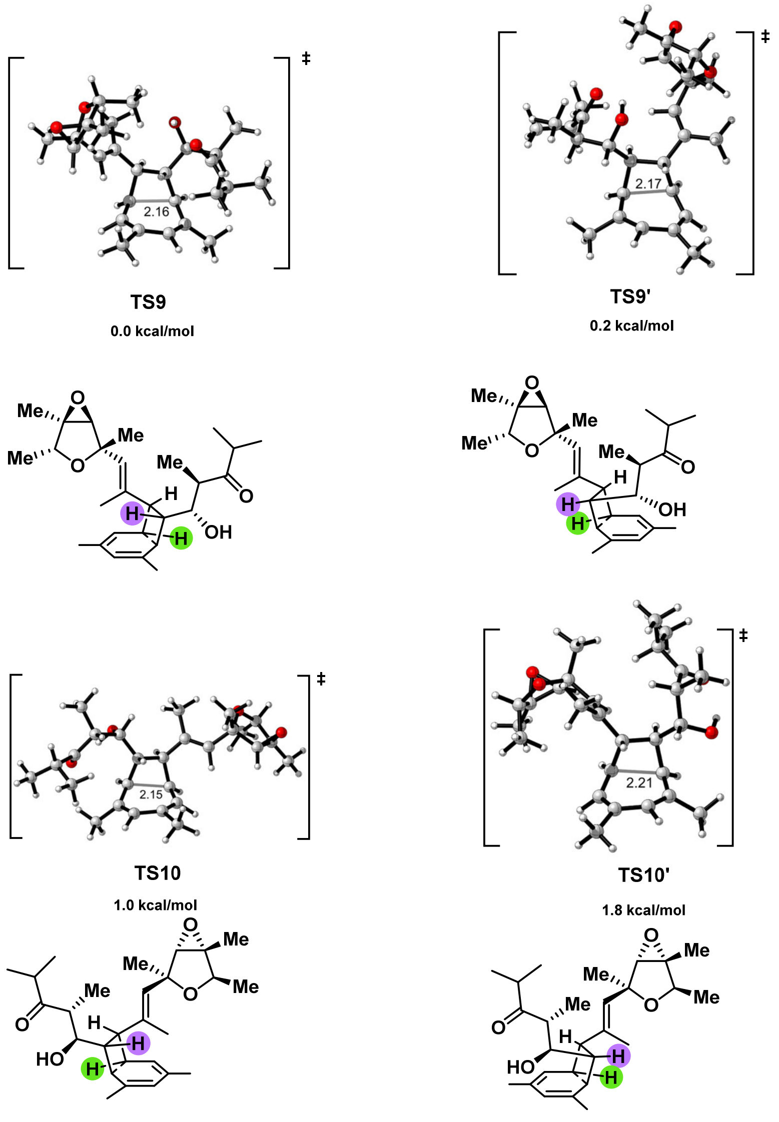

3.5 Figure S7: DFT-calculations after replacing both methyls with hydrogens

4 XYZ coordinates of optimized DFT structures and corresponding energies

Compound 9

E(toluene)[M06-2X/Def2-TZVP, SMD (toluene)] = -1432.312336 a.u.
Gcorr[M06-2X/Def2-SVP] = 0.607291 a.u.
Hcorr[M06-2X/Def2-SVP] = 0.708622 a.u.
G = E(toluene) + Gcorr= -1431.705045 a.u.
H = E(toluene) + Hcorr= -1431.603714 a.u.

C	-1.70020500	0.77281900	0.37425000
C	-2.14646500	0.41011600	-0.83813700
C	-2.09908100	1.94838500	1.17641400
C	-2.04864600	3.23067000	0.76945600

C	1.10531900	2.56301900	0.40144100
C	0.57596400	2.55995400	-0.83991600
C	-0.50132100	3.49959100	-1.20878900
C	-1.62773300	3.79921200	-0.53109600
C	-3.11976700	1.20662900	-1.65762400
H	-3.65812700	1.93946600	-1.04318900
H	-3.85240800	0.55783000	-2.16276100
H	-2.57938600	1.76351200	-2.44185700
C	1.02491800	1.66256600	-1.96645500
H	2.04277000	1.28229600	-1.80636800
H	0.36372200	0.78619400	-2.06134300
H	0.99012900	2.21155800	-2.92001500
H	-0.37826700	3.98532600	-2.18554600
C	-2.58639700	4.83159800	-1.07240500
H	-2.71359500	5.65538300	-0.35167900
H	-2.23340600	5.25568300	-2.02142800
H	-3.58581700	4.39495900	-1.23146100
C	-2.53441100	1.60759800	2.57941200
H	-3.43140800	0.96824300	2.56606800
H	-1.74474800	1.03857100	3.09630900
H	-2.75544900	2.50814200	3.16699500
H	-2.41108000	3.98052300	1.48483300
H	-0.99657300	0.09843800	0.87728700
H	0.76127300	3.34504900	1.08814200
C	2.10008700	1.61890900	0.94662100
C	3.13721600	2.22074100	1.86043200
H	3.76701200	2.93370700	1.30405300
H	2.63559900	2.79855600	2.65312300
H	3.78140500	1.46092000	2.31257600
C	1.98958500	0.31343500	0.63856400
H	1.12948100	0.02026100	0.02860400
C	2.84441200	-0.86741000	1.03917600
O	4.19635000	-0.55197800	1.36331700
C	5.05800100	-0.68881800	0.24439200
C	4.33423500	-1.64079000	-0.69630100
C	2.96729400	-1.76532700	-0.18640000
H	5.98523500	-1.15926600	0.61314600
H	2.09197800	-1.96947900	-0.81272700
C	2.24983900	-1.60091000	2.23738000
H	2.30073000	-0.95490200	3.12456500
H	1.19705200	-1.86068500	2.05257800
H	2.82930900	-2.51640300	2.41737200
C	5.37073000	0.65638000	-0.39494100
H	5.78278500	1.33552500	0.36324300
H	6.11098300	0.54912300	-1.20121600
H	4.45675500	1.11180700	-0.80564100
C	4.75303300	-1.81110300	-2.12711000
H	4.65005200	-0.86998800	-2.68428500
H	5.80208200	-2.13804300	-2.18655500
H	4.12579900	-2.57631700	-2.60138900
O	3.90592900	-2.80393200	-0.00579700
C	-1.67756600	-0.89356400	-1.47485500
C	-2.76651900	-1.99157700	-1.33867400
C	-2.98193200	-2.24867300	0.14795000
O	-2.05756900	-2.64161700	0.83017700
C	-4.35020300	-1.98007000	0.73890900
H	-4.59553200	-0.94482800	0.43460600
C	-4.32298300	-2.08766400	2.25610200
H	-5.30247300	-1.82709400	2.68031600
H	-4.06926100	-3.11300600	2.56015900
H	-3.56068600	-1.42321000	2.68387200
C	-5.38335000	-2.92565900	0.11038300
H	-6.38609600	-2.69699200	0.49660900
H	-5.41781900	-2.84067200	-0.98514800
H	-5.15202400	-3.97020100	0.36783000
O	-0.44558100	-1.35861400	-0.99338600

H	-0.61764600	-1.88327000	-0.19288200
H	-1.54496400	-0.70752400	-2.55666800
H	-3.69768700	-1.60165600	-1.77442900
C	-2.35768100	-3.27902900	-2.05395700
H	-1.44068900	-3.68298600	-1.60668400
H	-3.14577600	-4.04214100	-1.98147900
H	-2.16451800	-3.07944900	-3.11736300

TS1

E(toluene)[M06-2X/Def2-TZVP, SMD (toluene)] = -1432.277673 a.u.
Gcorr[M06-2X/Def2-SVP] = 0.610552 a.u.
Hcorr[M06-2X/Def2-SVP] = 0.709186 a.u.
G = E(toluene) + Gcorr= -1431.667121 a.u.
H = E(toluene) + Hcorr= -1431.568487 a.u.

C	1.33080900	0.61294500	1.24424200
C	1.56406700	0.38322700	-0.12526000
C	1.30003300	1.83693500	1.91933900
C	1.22064600	3.15766400	1.40931300
C	-0.37007800	1.30425900	-0.69700900
C	0.11973600	2.25095700	-1.61293300
C	0.68576800	3.43205100	-1.11717100
C	1.02690000	3.84557600	0.19031500
C	2.63199500	1.17966700	-0.82974400
H	2.77311700	2.16732200	-0.38571900
H	2.43483100	1.28977200	-1.90309500
H	3.58436400	0.63475600	-0.73231500
C	0.12922900	2.03251900	-3.10620000
H	-0.88400600	2.03677100	-3.53223900
H	0.71226600	2.81696100	-3.60607900
H	0.58310300	1.05927600	-3.34374500
H	0.87836100	4.19080900	-1.88221900
C	1.25716900	5.34753800	0.32949400
H	2.25110400	5.56763400	0.74573200
H	1.16571800	5.86600100	-0.63260400
H	0.51332800	5.78515300	1.01302500
C	1.31021800	1.76287600	3.43813600
H	2.23986700	2.19783100	3.83742300
H	0.47341300	2.32291100	3.88032400
H	1.25526400	0.72475200	3.79144600
H	1.35003100	3.87931200	2.22665400
H	1.05570100	-0.25867300	1.85158400
H	-0.49453300	1.73040600	0.29498600
C	-1.32495400	0.19535600	-0.91989500
C	-1.48660100	-0.45046100	-2.27171100
H	-1.94700400	0.25222400	-2.98132000
H	-0.50674900	-0.74120900	-2.67271600
H	-2.13994200	-1.32548500	-2.21520900
C	-2.00380900	-0.21429200	0.17339500
H	-1.78573100	0.30141900	1.11696800
C	-2.98964800	-1.36054800	0.29358000
O	-3.88602000	-1.47859700	-0.81067200
C	-5.09824100	-0.77786800	-0.58612800
C	-5.22779600	-0.70010100	0.92778500
C	-3.91936200	-1.07817700	1.46684000
H	-5.90558900	-1.40222700	-1.00395100
H	-3.52411400	-0.71330400	2.42025200
C	-2.27532800	-2.70223100	0.43347400
H	-3.02212700	-3.48793100	0.61116500
H	-1.72612300	-2.92595300	-0.49226800
H	-1.56053300	-2.67379300	1.26912000
C	-5.09038400	0.59190300	-1.24925500
H	-4.33054200	1.24165000	-0.78962900
H	-4.84696600	0.47840400	-2.31403900

H	-6.07362400	1.07778400	-1.16837200
C	-6.22540300	0.20155400	1.59312000
H	-6.03136700	1.25467700	1.34934500
H	-7.24592800	-0.04876100	1.26707900
H	-6.17019100	0.07017200	2.68111000
O	-5.00870000	-1.97046500	1.52110700
C	1.43344000	-1.05821000	-0.62207200
C	2.36370200	-2.06694600	0.09938600
C	3.83820600	-1.88779300	-0.22968400
O	4.20679200	-1.70401500	-1.37181400
C	4.82907300	-1.99012100	0.91523000
H	4.45989900	-2.78842500	1.58265100
C	4.80207000	-0.66687000	1.69651400
H	5.41933000	-0.75243500	2.60158000
H	5.21424100	0.14514100	1.07889200
H	3.78425800	-0.37599000	1.99754000
C	6.22698400	-2.32275900	0.41335000
H	6.24231900	-3.28241600	-0.12115900
H	6.56780700	-1.54850900	-0.28744800
H	6.93503500	-2.37665200	1.25179000
O	1.58375700	-1.14599500	-2.01715100
H	2.53395800	-1.21589600	-2.20585900
H	0.41000700	-1.39667900	-0.38956000
H	2.23057700	-1.95426400	1.18474000
C	1.98980000	-3.50192900	-0.30119300
H	2.65591000	-4.24068100	0.16812000
H	0.95802800	-3.72240300	0.00649000
H	2.05714300	-3.60720100	-1.39177300

TS2
E(toluene)[M06-2X/Def2-TZVP, SMD (toluene)] = -1432.278383 a.u.
Gcorr[M06-2X/Def2-SVP] = 0.610927 a.u.
Hcorr[M06-2X/Def2-SVP] = 0.709267 a.u.
G = E(toluene) + Gcorr= -1431.667456 a.u.
H = E(toluene) + Hcorr= -1431.569116 a.u.

C	-1.01889600	-1.22068100	-0.25578500
C	-5.63726000	-1.53862800	0.52545000
H	-5.82094500	-2.45704000	1.09999400
H	-5.28731400	-0.77008100	1.22850600
H	-6.58409500	-1.20299100	0.08039600
C	-2.03692200	-2.24470200	-0.83805500
C	-3.28145300	-2.24146100	0.04144900
C	-4.59799100	-1.78932900	-0.55632100
H	-4.38154000	-0.84751800	-1.09048700
C	-5.06140200	-2.83277900	-1.58326400
H	-4.31042400	-3.00640400	-2.36779400
H	-5.26756800	-3.79218300	-1.08550000
H	-5.98691200	-2.49721600	-2.07097600
O	-3.19670300	-2.59509900	1.20005500
C	-1.42452300	-3.64526900	-0.88571300
H	-2.12890000	-4.37155000	-1.31627600
H	-0.51285000	-3.64052400	-1.49966300
H	-1.15805400	-3.97820500	0.12528000
H	-2.30535100	-1.92639100	-1.85442700
O	-0.61555100	-1.60778700	1.02592600
H	-1.36466200	-2.03870600	1.46844600
H	-0.12669600	-1.27203900	-0.90398200
C	0.33170000	2.34973000	-0.91907300
C	0.22910500	1.47757500	0.18535600
C	-0.55706500	3.42331400	-1.06653600
C	-1.74606800	3.80594300	-0.40979000
C	-1.53985200	0.21742700	-0.31127700
C	-2.30784600	0.63736400	0.78723300

C	-2.87463100	1.88702200	1.05288500
C	-2.63152400	3.15462800	0.47503800
H	-3.36137600	3.87854200	0.86050200
H	-2.41351100	-0.09155300	1.59667300
H	-0.25283000	4.13461100	-1.84023200
C	-1.87198900	0.68521900	-1.70361200
H	-2.09747500	1.75316600	-1.74742600
H	-1.05039800	0.47090100	-2.40397700
H	-2.76781500	0.15632200	-2.06661700
C	-3.89458700	1.91587600	2.18201100
H	-3.67695100	2.71230700	2.90772000
H	-4.90402400	2.10349400	1.78279500
H	-3.92444200	0.95933900	2.71979500
C	-2.17902700	5.24088500	-0.70134900
H	-1.55654600	5.70778700	-1.47426400
H	-3.22691900	5.28768900	-1.03060100
H	-2.09435000	5.85665900	0.20749500
C	1.40089100	2.21373100	-1.97653600
H	1.19743800	1.37610400	-2.66335500
H	1.44952700	3.12502900	-2.58634900
H	2.38942300	2.02937800	-1.53096200
H	-0.33961900	1.91641900	1.00283700
C	1.27203400	0.54151700	0.66290400
C	1.33723400	0.45409700	2.16662700
H	0.32132200	0.26180500	2.54217300
H	2.00872600	-0.33440200	2.51506300
H	1.67450200	1.41587300	2.58659900
C	2.04297200	-0.18372400	-0.17053900
H	1.85430200	-0.10941200	-1.24452100
C	3.10812700	-1.20333800	0.18607100
O	3.85170400	-0.88542900	1.36108400
C	5.03518800	-0.16366700	1.06510100
C	5.35986000	-0.52149200	-0.37753500
C	4.16753300	-1.18523000	-0.90892600
H	5.81967100	-0.55392300	1.73512000
H	3.88081500	-1.16701800	-1.96562000
C	2.50150500	-2.58739500	0.39146800
H	1.78343200	-2.55716700	1.22119700
H	1.95818800	-2.90294200	-0.51179700
H	3.30849900	-3.30170500	0.60330600
C	4.85597700	1.33232400	1.28075000
H	4.49186200	1.51154900	2.30109700
H	5.80769800	1.86853400	1.15316600
H	4.11617200	1.74083600	0.57589100
C	6.36873100	0.24773800	-1.17862600
H	7.35144100	0.22381300	-0.68437600
H	6.47247200	-0.20749300	-2.17168300
H	6.06500100	1.29690900	-1.29545500
O	5.31706800	-1.92678000	-0.57008400

Compound 47
E(toluene)[M06-2X/Def2-TZVP, SMD (toluene)] = -1432.317445 a.u.
Gcorr[M06-2X/Def2-SVP] = 0.61371 a.u.
Hcorr[M06-2X/Def2-SVP] = 0.711369 a.u.
G = E(toluene) + Gcorr= -1431.703735 a.u.
H = E(toluene) + Hcorr= -1431.606076 a.u.

C	-1.42198700	2.64722600	1.69572800
C	-0.53005000	3.15569300	0.63399600
C	-1.47360700	1.42325400	2.25839400
C	-0.67999500	0.23047400	1.94463400
H	-2.15255800	1.31035800	3.11333900
C	-0.47782400	-0.25685800	0.70937700
C	0.00538100	2.70121000	-0.52140400

H	-0.34839700	4.22623500	0.78810200
C	-0.00396500	1.33975700	-1.19860400
C	-0.94598000	0.21912600	-0.65281700
H	0.15763900	-1.14917500	0.66043400
C	-2.40808600	0.77400600	-0.73622100
C	-0.81698200	-0.99516500	-1.59276600
H	-1.15689800	-0.75262000	-2.60793600
H	0.22884600	-1.32661800	-1.64126300
H	-1.40104200	-1.85041600	-1.22508000
H	-0.38148700	1.53968500	-2.21646800
C	-2.34600900	3.72516800	2.21611700
H	-2.98328900	3.35834400	3.03080800
H	-1.77013500	4.58956700	2.58232700
H	-2.99411200	4.09646800	1.40578500
C	-0.11374400	-0.47887700	3.15099400
H	-0.91804500	-0.76425100	3.84825200
H	0.43594400	-1.38618100	2.86631300
H	0.56650300	0.18458200	3.70780700
C	0.73940300	3.71562700	-1.37411800
H	1.77652600	3.40040000	-1.56366100
H	0.24468900	3.80939100	-2.35452000
H	0.76054100	4.70639700	-0.90364200
C	-3.48448500	-0.04128000	0.01737400
C	-3.72574200	-1.39259900	-0.63312000
O	-4.06059600	-1.46565300	-1.79783700
C	-3.55538700	-2.63323400	0.22513300
H	-2.56871700	-2.52175100	0.71291500
C	-3.59424200	-3.89973300	-0.61652800
H	-4.56591300	-3.98932000	-1.12207300
H	-2.82187100	-3.88144800	-1.39790900
H	-3.43999300	-4.78774900	0.01210500
C	-4.62308500	-2.64066600	1.32798500
H	-4.58400700	-1.73410400	1.94868500
H	-5.62888600	-2.71526700	0.88742400
H	-4.47734700	-3.50742800	1.98757000
C	1.44778900	0.86040900	-1.36458200
C	2.25038800	0.78539200	-0.29419100
H	1.83187800	1.11159700	0.66472400
C	1.87850700	0.59138800	-2.78141200
H	1.18298400	-0.10582100	-3.27397100
H	1.82378800	1.53275300	-3.35449100
H	2.89378900	0.18801700	-2.84150300
C	3.71803800	0.40690200	-0.23829100
O	4.09797000	-0.62066100	-1.15393900
C	4.06170900	-1.90622300	-0.55787700
C	4.21646100	-1.65162800	0.93333100
C	4.00951200	-0.21339800	1.12169100
H	4.93381700	-2.45951200	-0.94501700
H	3.60558300	0.22926200	2.03799700
C	4.60531400	1.61314300	-0.52655700
H	5.65598200	1.32872400	-0.37994800
H	4.45716800	1.93545000	-1.56684700
H	4.34980300	2.45034300	0.13833200
C	2.77400900	-2.64702700	-0.88834100
H	1.91563300	-2.15731300	-0.40370200
H	2.61056800	-2.63391500	-1.97467500
H	2.81952000	-3.69410100	-0.55518400
C	3.94584500	-2.71983800	1.95192300
H	4.61405300	-3.57925800	1.79261700
H	4.13021200	-2.32276300	2.95824200
H	2.90731700	-3.07257900	1.89091600
O	5.30094900	-0.77608600	1.19163600
O	-2.77478300	0.98889700	-2.07772200
H	-3.22268900	0.19122800	-2.40010100
H	-2.41702800	1.76550100	-0.27270600
H	-3.13310600	-0.19671200	1.04500700

C	-4.79790100	0.74833600	0.03958600
H	-5.59776200	0.19246100	0.54989600
H	-4.64779100	1.70277900	0.56515100
H	-5.11973300	0.96329700	-0.98775400

Compound 48
E(toluene)[M06-2X/Def2-TZVP, SMD (toluene)] = -1432.318021 a.u.
Gcorr[M06-2X/Def2-SVP] = 0.614907 a.u.
Hcorr[M06-2X/Def2-SVP] = 0.711934 a.u.
G = E(toluene) + Gcorr= -1431.703114 a.u.
H = E(toluene) + Hcorr= -1431.606087 a.u.

C	-0.72689200	3.26886400	-0.61840200
C	0.20365000	2.93699900	0.48178900
C	-2.03731300	2.95927200	-0.66237600
C	-2.74129900	2.14215500	0.33300000
H	-2.64337200	3.41496000	-1.45558300
C	-2.30746900	0.93351300	0.72511700
C	0.52087400	1.83966000	1.20084800
H	0.77996700	3.82781700	0.76786700
C	0.08072900	0.38858900	1.08047600
C	-1.16791000	0.09649400	0.18526800
H	-2.84239000	0.46931400	1.55889000
H	-0.24167600	0.09825200	2.09336800
C	-1.54711100	-1.40688200	0.39119000
C	-0.86159900	0.27063000	-1.31262600
H	-0.15703300	1.08336000	-1.50892200
H	-0.41157500	-0.65204600	-1.71050300
H	-1.77393800	0.49390900	-1.88298700
C	1.50026700	2.01531700	2.34023900
H	0.99765500	1.80412600	3.29776400
H	2.33697500	1.30688300	2.25706400
H	1.90443200	3.03462800	2.38312600
C	-0.11539100	4.14259900	-1.68450100
H	0.36861900	5.02588300	-1.23869600
H	0.67118000	3.59228900	-2.22704300
H	-0.86499600	4.48173500	-2.41120700
C	-3.97729500	2.76318900	0.93399000
H	-3.71866000	3.66087000	1.51708300
H	-4.67023000	3.09066500	0.14156100
H	-4.50505900	2.05608700	1.58876600
C	-4.97274400	0.16963300	-1.62230800
H	-5.69751200	0.33173100	-2.43243700
H	-5.12419700	0.94516700	-0.85796700
H	-3.96150500	0.29818100	-2.03360800
C	-2.73637000	-1.90612800	-0.45975200
C	-4.11239900	-1.48223500	0.03806400
C	-5.18108300	-1.22269600	-1.01078000
H	-5.02478200	-1.97097300	-1.80765000
C	-6.57580100	-1.37073000	-0.41785200
H	-6.72612100	-2.37327700	0.00558700
H	-6.71689400	-0.64508400	0.39491900
H	-7.34170300	-1.19463500	-1.18579100
O	-4.37535400	-1.44151900	1.22306200
C	1.27920200	-0.51904500	0.75266700
C	1.46975700	-1.69012300	1.68170100
H	0.50563600	-2.16447600	1.91198200
H	2.18431200	-2.42277000	1.29492200
H	1.85869500	-1.31234200	2.64477800
C	2.07635200	-0.22474300	-0.28429400
H	1.84743100	0.68855200	-0.84625000
C	3.22095500	-1.02769000	-0.87398700
O	3.95582200	-1.81035300	0.06543800
C	5.12504500	-1.15033500	0.52053800

C	5.44628200	-0.12920100	-0.55926600
C	4.26676000	-0.06805100	-1.42542500
H	5.92170600	-1.91178900	0.56997300
H	3.97024100	0.82247900	-1.98881600
C	2.70257100	-1.98843500	-1.94087800
H	3.55574400	-2.48707000	-2.42031200
H	2.05766100	-2.74186600	-1.46647300
H	2.11449500	-1.44938200	-2.69750000
C	4.91443600	-0.51684800	1.88697900
H	4.55559400	-1.27752100	2.59282300
H	5.84997300	-0.09214200	2.27904200
H	4.15953000	0.28151000	1.82385700
C	6.42929500	0.98171000	-0.33355700
H	6.52414000	1.57784500	-1.24987400
H	6.10648600	1.63603700	0.48731400
H	7.41996900	0.57298400	-0.08410400
O	5.43909600	-0.73135000	-1.84373000
C	-2.75163800	-3.44556400	-0.46683400
H	-1.84274300	-3.82874700	-0.95080300
H	-2.77666000	-3.81557800	0.56678600
H	-3.62278700	-3.84535400	-1.00608900
H	-2.61744900	-1.55498400	-1.49420600
O	-1.72161500	-1.74004600	1.74394700
H	-2.66511000	-1.64941200	1.95703400
H	-0.67080200	-1.97918400	0.03840100

TS3

E(toluene)[M06-2X/Def2-TZVP, SMD (toluene)] = -1432.27947 a.u.
Gcorr[M06-2X/Def2-SVP] = 0.612549 a.u.
Hcorr[M06-2X/Def2-SVP] = 0.709135 a.u.
G = E(toluene) + Gcorr= -1431.666921 a.u.
H = E(toluene) + Hcorr= -1431.570335 a.u.

C	-0.48997200	2.78923900	1.61006400
C	0.17736700	3.09422000	0.41128700
C	-1.83556300	2.38591200	1.65107900
C	-2.59127700	2.12515500	0.50632400
H	-2.35828200	2.55046600	2.59992900
C	-1.95923800	1.65237700	-0.64748200
C	-0.01041100	2.66014700	-0.91653300
H	0.84135900	3.96558600	0.51047300
C	0.14129700	1.22995000	-1.44762600
C	-0.99302300	0.46617500	-0.72936100
H	-2.52610400	1.77850900	-1.58253700
C	-1.63060800	-0.61677700	-1.65548200
C	-0.55573300	-0.16714100	0.59073100
H	0.16050900	0.45134500	1.13845900
H	-1.40272800	-0.34376200	1.26489400
H	-0.06301700	-1.12595500	0.38812500
H	-0.17893100	1.28958800	-2.50071200
C	0.17538500	3.18818600	2.90453000
H	-0.55322100	3.49079100	3.67069600
H	0.74626000	2.33771800	3.31425100
H	0.89065300	4.01128400	2.75633600
C	-4.04729900	2.52796200	0.45290200
H	-4.70372300	1.68075300	0.19288600
H	-4.39221500	2.94524500	1.40941900
H	-4.20021200	3.30133500	-0.31795900
C	0.33893000	3.67917100	-1.98128900
H	0.39509400	4.70274700	-1.58592300
H	1.30386300	3.42649600	-2.45449700
H	-0.42211700	3.66568400	-2.77673800
C	-3.04171000	-1.09229200	-1.22176400
C	-2.98299200	-1.91951400	0.05296800

O	-2.26382600	-2.89906500	0.10910700
C	-3.85290100	-1.49657100	1.22383300
H	-3.65657000	-0.41719100	1.36162800
C	-3.48856600	-2.25142100	2.49459400
H	-2.42999100	-2.10444100	2.75197000
H	-4.10318800	-1.90517400	3.33836000
H	-3.64817400	-3.33167100	2.36500600
C	-5.33674000	-1.65221300	0.85852600
H	-5.60947900	-1.07233900	-0.03588100
H	-5.58397400	-2.70880400	0.67123400
H	-5.96722800	-1.30001900	1.68775100
C	1.55248200	0.65502600	-1.47024000
C	2.36275600	0.82391300	-0.41793100
H	1.99554200	1.47263000	0.38565100
C	1.89767500	-0.09691100	-2.72750900
H	1.82390300	0.58986700	-3.58816300
H	2.90581300	-0.52020200	-2.69221400
H	1.16706500	-0.90431400	-2.88888100
C	3.73984100	0.26296800	-0.12352500
O	4.08391600	-0.90873900	-0.85361300
C	3.76146400	-2.09962500	-0.15171800
C	3.67024900	-1.68010300	1.30888300
C	3.70758400	-0.22201200	1.32288000
H	4.61813700	-2.78388900	-0.28125600
H	3.24001600	0.39176200	2.10126200
C	4.83171000	1.29608000	-0.38220600
H	4.89510100	1.50253500	-1.45989900
H	4.61210600	2.23884100	0.14029700
H	5.79639700	0.90113500	-0.03404400
C	2.49820000	-2.75726700	-0.68780800
H	1.62153700	-2.10471400	-0.57188600
H	2.61647300	-2.96356500	-1.76006100
H	2.30540700	-3.71276200	-0.17686800
C	3.01716100	-2.54707400	2.34444800
H	3.49998600	-3.53550400	2.38504500
H	3.11056400	-2.07933400	3.33362100
H	1.95080500	-2.69255800	2.11966700
O	4.84376200	-0.96987500	1.69631300
O	-0.77793000	-1.70344000	-1.88173900
H	-0.99201700	-2.39110600	-1.23048000
H	-1.77551000	-0.12662200	-2.63612600
H	-3.65729100	-0.20059200	-1.03701900
C	-3.69063700	-1.92457100	-2.33470100
H	-3.79360800	-1.32129400	-3.24864200
H	-3.06838400	-2.79839600	-2.57070200
H	-4.69057900	-2.27907500	-2.04424700

TS3'
E(toluene)[M06-2X/Def2-TZVP, SMD (toluene)] = -1432.276648 a.u.
Gcorr[M06-2X/Def2-SVP] = 0.612687 a.u.
Hcorr[M06-2X/Def2-SVP] = 0.709333 a.u.
G = E(toluene) + Gcorr= -1431.663961 a.u.
H = E(toluene) + Hcorr= -1431.567315 a.u.

C	-2.50297100	-3.62322700	0.37997700
C	-1.45574700	-3.66608300	-0.54925300
C	-3.37747600	-2.52053900	0.43239700
C	-3.38012100	-1.45128700	-0.48035700
H	-4.31009900	-2.69675700	0.98024000
C	-2.22902800	-0.90301600	-1.07579900
C	-0.76335600	-2.53858000	-1.01018700
H	-1.27458800	-4.63161300	-1.03941400
C	-0.28277400	-1.54620300	0.06753000
C	-1.05208800	-0.26680800	-0.30605500

H	-2.44147900	-0.38263400	-2.02362100
H	-0.76871900	-1.90036000	0.98526000
C	-1.51266100	0.45294700	0.98963900
C	-0.30602000	0.68921700	-1.23500900
H	-0.98130200	1.47075400	-1.61271500
H	0.53581200	1.17364100	-0.72378300
H	0.08526300	0.17453800	-2.12161000
C	0.00151500	-2.70462900	-2.30415600
H	0.98897400	-3.15735100	-2.12185800
H	-0.55554000	-3.35459800	-2.99281800
H	0.17001700	-1.74960900	-2.81835300
C	-2.87834300	-4.87919700	1.12631200
H	-2.58671100	-5.77756400	0.56146400
H	-2.34574100	-4.92232700	2.09033000
H	-3.95581900	-4.93623200	1.33826200
C	-4.72699200	-0.99395300	-1.00560900
H	-5.56055800	-1.54048500	-0.54322800
H	-4.89642100	0.08396800	-0.84829600
H	-4.78013600	-1.17008300	-2.09298700
C	-1.68171500	4.63984900	-1.54624700
H	-0.72413300	4.21842200	-1.88370800
H	-2.18024700	5.10557700	-2.40891000
H	-1.45356000	5.42339300	-0.80937900
C	-2.51743600	1.61725500	0.80866400
C	-1.87349800	2.86653300	0.22765100
C	-2.56886900	3.56537100	-0.93222500
H	-2.76767000	2.77731800	-1.68165700
C	-3.93098200	4.11802800	-0.48717600
H	-4.59997500	3.32814300	-0.11547700
H	-3.80941400	4.87054100	0.30742900
H	-4.43171100	4.60423700	-1.33700700
O	-0.84195300	3.30636500	0.69983500
C	1.20385900	-1.50559200	0.40009100
C	1.50691100	-1.89418600	1.82230900
H	0.99874900	-1.18741200	2.49815400
H	1.08971100	-2.89558000	2.02147800
H	2.57843400	-1.89247300	2.04293600
C	2.12669700	-1.13929100	-0.49993400
H	1.78829300	-0.92259300	-1.51509500
C	3.63022700	-0.97890300	-0.36111600
O	4.08616200	-0.68350300	0.95364600
C	4.18634400	0.71239000	1.18760000
C	4.31379400	1.33147100	-0.19746400
C	4.01391600	0.26844800	-1.15138400
H	5.12437500	0.87071700	1.74755300
H	3.58988300	0.44560700	-2.14644600
C	4.36846200	-2.23132600	-0.82283900
H	4.05681100	-2.51762500	-1.83852300
H	5.45109000	-2.04276500	-0.81298200
H	4.14039500	-3.06385100	-0.14233800
C	3.00786200	1.24319000	1.98944700
H	2.93435500	0.70138300	2.94216800
H	3.13534200	2.31287700	2.21482600
H	2.05933900	1.09783300	1.45320800
C	4.10496400	2.79867800	-0.43224300
H	4.30098500	3.03846400	-1.48586100
H	3.07376100	3.09189000	-0.18718100
H	4.78966400	3.39514100	0.19006300
O	5.34393200	0.68710300	-0.94066900
O	-0.41694100	0.83595700	1.77865500
H	-0.21003000	1.76060100	1.56848700
H	-2.05998400	-0.31151100	1.56671500
C	-3.14890300	1.96327100	2.16566800
H	-3.69869200	1.09247400	2.55152200
H	-2.36415700	2.22054300	2.89008300
H	-3.84999800	2.80713400	2.09596000

H	-3.30759800	1.27787700	0.12398200

TS4
E(toluene)[M06-2X/Def2-TZVP, SMD (toluene)] = -1432.279521 a.u.
Gcorr[M06-2X/Def2-SVP] = 0.611649 a.u.
Hcorr[M06-2X/Def2-SVP] = 0.709359 a.u.
G = E(toluene) + Gcorr= -1431.667872 a.u.
H = E(toluene) + Hcorr= -1431.570162 a.u.

C	-0.36993800	2.89139900	-0.39746200
C	0.41878200	2.28563300	0.59600100
C	-1.76943400	2.76784900	-0.41662000
C	-2.46867300	1.94277300	0.46924600
H	-2.31478600	3.56538000	-0.93510600
C	-1.88012500	0.76028100	0.92455000
C	0.22315400	1.11723800	1.36005200
H	1.22309600	2.94403400	0.95767500
C	0.13350400	-0.31329300	0.81398500
C	-1.21322600	-0.31730600	0.06344400
H	-2.31498100	0.33389900	1.83454500
H	-0.04278400	-0.94877500	1.69620600
C	-1.93399100	-1.69538100	0.23122600
C	-1.04918500	-0.02655200	-1.42822500
H	-0.25302500	0.70012200	-1.62169300
H	-0.76967700	-0.95326800	-1.95526100
H	-1.96987500	0.36764400	-1.87739300
C	0.82597300	1.14451700	2.74955500
H	0.14737100	0.65124500	3.46214900
H	1.77498700	0.58003700	2.76877000
H	1.01197500	2.16553800	3.11092900
C	0.27216400	3.94655100	-1.26429000
H	1.10515300	4.44500700	-0.74443600
H	0.69287400	3.48780300	-2.17472000
H	-0.44738700	4.71349800	-1.58578200
C	-3.72340000	2.46942000	1.12805900
H	-3.46874700	3.04670600	2.03328000
H	-4.26351500	3.15574300	0.45743100
H	-4.40448500	1.66056200	1.42794600
C	-4.88270500	0.54545000	-1.87346700
H	-5.67664700	0.92167400	-2.53470900
H	-4.46111600	1.39870200	-1.32352700
H	-4.09055200	0.13294000	-2.51344600
C	-3.27013500	-1.85816400	-0.52997100
C	-4.41607900	-1.04068300	0.04922900
C	-5.46541800	-0.50096500	-0.91308900
H	-5.76366800	-1.37388900	-1.52410500
C	-6.68719200	0.02644600	-0.17007800
H	-7.08981800	-0.72164500	0.52632800
H	-6.42993200	0.91998600	0.41738900
H	-7.47689800	0.30216500	-0.88408600
O	-4.53842200	-0.90940700	1.25276900
C	1.35864300	-0.87694400	0.11327200
C	1.37902700	-2.38045800	-0.02225800
H	0.92672100	-2.86323300	0.85724100
H	0.78603600	-2.69371100	-0.89863600
H	2.39884200	-2.75532600	-0.16140700
C	2.33042300	-0.08775600	-0.36525300
H	2.19954700	0.99240500	-0.25184300
C	3.59070500	-0.49573700	-1.11054100
O	4.24522000	-1.64163700	-0.56314800
C	5.22149300	-1.28305200	0.39820700
C	5.62549700	0.13831800	0.02902100
C	4.62585300	0.60677200	-0.92770300
H	6.07305300	-1.96964000	0.25263700

H	4.34306400	1.65849300	-1.04960100
C	3.30276700	-0.79763900	-2.57699000
H	4.24671500	-1.00599100	-3.09966300
H	2.64670100	-1.67592600	-2.65537500
H	2.79763200	0.05464400	-3.05493800
C	4.69125300	-1.40663200	1.82098600
H	4.28185900	-2.41467900	1.97483800
H	5.49064000	-1.24392900	2.55968300
H	3.88469700	-0.68067700	2.00362100
C	6.43077600	1.00079700	0.95578700
H	6.62229200	1.97515400	0.48715400
H	5.90371000	1.16231600	1.90673900
H	7.40075400	0.52842900	1.17462100
O	5.91663800	0.23685600	-1.35996900
C	-3.70827300	-3.33357400	-0.51495000
H	-2.95471200	-3.95313900	-1.02259100
H	-3.80306500	-3.69205500	0.51894000
H	-4.66970500	-3.48153300	-1.02910900
H	-3.11723900	-1.56589800	-1.57718700
O	-2.06960200	-2.07528200	1.57425700
H	-2.90694400	-1.69865500	1.88756700
H	-1.25225600	-2.43224000	-0.22266600

TS4’

E(toluene)[M06-2X/Def2-TZVP, SMD (toluene)] = -1432.277991 a.u.
Gcorr[M06-2X/Def2-SVP] = 0.612985 a.u.
Hcorr[M06-2X/Def2-SVP] = 0.709266 a.u.
G = E(toluene) + Gcorr= -1431.665006 a.u.
H = E(toluene) + Hcorr= -1431.568725 a.u.

C	1.99843400	-3.45590300	0.92910500
C	0.81498900	-3.32306500	0.18475700
C	3.18538400	-2.83050900	0.51133800
C	3.36840000	-2.16054600	-0.71450400
H	4.09549700	-3.18981600	1.00469700
C	2.39991500	-1.38790600	-1.37307300
C	0.48790700	-2.18609400	-0.55726000
H	0.20431600	-4.22777500	0.06462200
C	0.68976500	-0.82164100	0.13174900
C	1.72030100	-0.13560500	-0.77479400
H	2.59436200	-1.27934800	-2.44998400
C	2.77195600	0.66351300	0.04891500
C	1.18829000	0.70810700	-1.93449700
H	0.57214800	1.55186700	-1.60079900
H	2.03992200	1.11228400	-2.50006000
H	0.58518200	0.11153300	-2.63216600
H	1.25262600	-1.06191300	1.04453900
C	2.06628600	-4.49735600	2.01858600
H	3.09078600	-4.85631200	2.19160700
H	1.68999700	-4.08540000	2.96936700
H	1.43200200	-5.36402400	1.77522200
C	4.68454800	-2.36767300	-1.43265200
H	5.27015300	-1.43481800	-1.41394200
H	5.28583200	-3.17806500	-0.99772700
H	4.50196400	-2.60809300	-2.49230100
C	-0.57615100	-2.34415200	-1.62091300
H	-1.58825900	-2.27731000	-1.19068500
H	-0.50987500	-1.57147500	-2.39710600
H	-0.47463400	-3.31982400	-2.11532300
C	2.21357100	1.85206200	0.86838900
C	1.82109700	3.02802400	-0.01213300
O	2.61028400	3.47750000	-0.82182900
C	0.43494800	3.61859000	0.16466100
H	-0.25133900	2.75582700	0.10532000

C	0.10139800	4.61728200	-0.93382500
H	-0.92187600	5.00101400	-0.80698300
H	0.79738200	5.46867500	-0.91433300
H	0.18035400	4.15482700	-1.92829200
C	0.27522400	4.21790700	1.56892500
H	0.97868600	5.05049300	1.72608600
H	-0.74454900	4.61109400	1.69272500
H	0.44052100	3.47172900	2.36008300
C	-0.60318100	-0.17766400	0.62260100
C	-1.51862100	0.32203600	-0.22082700
H	-1.26194300	0.34649100	-1.28254000
C	-0.78813800	-0.27369900	2.11506600
H	0.04873700	0.22798100	2.62985600
H	-1.73513800	0.15351000	2.45692700
H	-0.74553700	-1.33214500	2.42317600
C	-2.92528400	0.83432400	0.04524200
O	-3.54806000	0.28290100	1.20079600
C	-4.31126000	-0.87553300	0.90316700
C	-4.62186600	-0.76741400	-0.58346300
C	-3.79707100	0.32240100	-1.09769700
H	-5.24840900	-0.79228800	1.48014600
H	-3.44389600	0.38412100	-2.13332900
C	-2.97559700	2.35146900	0.19091000
H	-4.02344400	2.67265100	0.26786700
H	-2.44151500	2.65538300	1.10267100
H	-2.50580100	2.84585500	-0.67231400
C	-3.58409300	-2.15545300	1.29169100
H	-4.22274300	-3.03661500	1.12932400
H	-2.65476800	-2.27934500	0.71632300
H	-3.32017900	-2.11893500	2.35746100
C	-5.11899200	-1.93905300	-1.37754900
H	-5.32288800	-1.63086700	-2.41147500
H	-4.37522300	-2.74855400	-1.39030400
H	-6.05124800	-2.33420200	-0.94565200
O	-5.17709900	0.50926200	-0.88181900
O	3.86885800	1.04061500	-0.73702400
H	3.71615700	1.95033500	-1.03836700
H	3.15446800	-0.05050700	0.79859600
H	1.32125600	1.50252000	1.40655800
C	3.25984900	2.32369400	1.88796400
H	2.90787000	3.19125800	2.46500000
H	3.49101000	1.51290700	2.59427400
H	4.18812200	2.60219100	1.37097700

Compound 10
E(toluene)[M06-2X/Def2-TZVP, SMD (toluene)] = -1432.330959 a.u.
Gcorr[M06-2X/Def2-SVP] = 0.614621 a.u.
Hcorr[M06-2X/Def2-SVP] = 0.711321 a.u.
G = E(toluene) + Gcorr= -1431.716338 a.u.
H = E(toluene) + Hcorr= -1431.619638 a.u.

C	0.22170100	3.46465900	-1.49035400
C	-0.38753100	3.24304500	-0.31435500
C	1.64649600	3.12198600	-1.66086300
C	2.36013100	2.47028300	-0.72770600
H	2.12851600	3.42221800	-2.59672600
C	1.69284700	2.05519300	0.55576500
C	0.32790700	2.71820600	0.90136600
H	-1.43532800	3.53129400	-0.17970900
C	-0.14411900	1.33398800	1.44732000
C	1.03191600	0.63732600	0.67938400
H	2.39681000	2.19635600	1.39549700
C	1.73085800	-0.36760900	1.61012400
C	0.66547700	0.03214600	-0.66762800

H	0.10701200	0.74225000	-1.28856400
H	1.57330900	-0.22450900	-1.23468400
H	0.05793500	-0.87601700	-0.54424300
H	0.15446900	1.32606600	2.50774800
C	-0.48359100	4.06726300	-2.67289300
H	0.00635200	5.00341400	-2.98361800
H	-0.44821800	3.38633100	-3.53821100
H	-1.53521100	4.28560100	-2.44568100
C	3.80888200	2.12990100	-0.91705800
H	3.95129200	1.03876800	-0.99346800
H	4.21678400	2.58490800	-1.82938600
H	4.40943600	2.46653800	-0.05689200
C	0.36432900	3.82712100	1.95036700
H	0.86742600	4.72117500	1.55217500
H	-0.65791100	4.11208400	2.24510600
H	0.90142200	3.49467400	2.85235700
C	3.00684400	-1.04983700	1.06799300
C	2.71946100	-2.05835300	-0.03796900
O	1.83797900	-2.88238400	0.09486200
C	3.59551000	-2.04426300	-1.28201800
H	3.54371800	-1.01421400	-1.67756700
C	3.07239200	-3.01909700	-2.32689400
H	2.03030100	-2.79247700	-2.59026200
H	3.68590900	-2.97400500	-3.23720600
H	3.09602000	-4.04619900	-1.93675100
C	5.05970300	-2.31991100	-0.91413300
H	5.46085200	-1.57543100	-0.21147200
H	5.16560400	-3.31732800	-0.46110900
H	5.68095900	-2.29451700	-1.82015900
C	-1.55623300	0.79362400	1.39776000
C	-2.24410200	0.73492200	0.24759400
H	-1.78903100	1.19151400	-0.63511200
C	-2.05539800	0.29236600	2.72855000
H	-2.02295300	1.12081200	3.45573900
H	-3.07093200	-0.10846300	2.67925800
H	-1.36616500	-0.48427600	3.09626500
C	-3.58982700	0.10463700	-0.05677000
O	-3.98096500	-0.93582900	0.83366000
C	-3.56043100	-2.21481600	0.38142000
C	-3.37357400	-2.05465500	-1.11928700
C	-3.43575500	-0.61788000	-1.39119400
H	-4.39550000	-2.90861600	0.57754700
H	-2.92271100	-0.13562800	-2.22991800
C	-4.71489500	1.13192800	-0.06704900
H	-4.86158600	1.52153800	0.94960700
H	-4.46901000	1.97214300	-0.73214600
H	-5.63947300	0.64713300	-0.40882500
C	-2.30543300	-2.69175300	1.09666800
H	-1.46378100	-2.00782800	0.91208600
H	-2.48442400	-2.71670200	2.17973800
H	-2.02679000	-3.70457800	0.76999400
C	-2.62934200	-3.06851000	-1.93753700
H	-3.08758800	-4.06329300	-1.83105100
H	-2.66576400	-2.78349500	-2.99679600
H	-1.57942500	-3.13559000	-1.61899000
O	-4.52357800	-1.45713400	-1.70235100
O	0.80668100	-1.31302100	2.09164400
H	0.80979700	-2.07164800	1.48636400
H	2.05899000	0.21306500	2.49181300
H	3.67367100	-0.26643800	0.67524800
C	3.70913900	-1.79254400	2.21585400
H	4.01760600	-1.07940300	2.99294300
H	3.00749900	-2.50822400	2.66369700
H	4.59900100	-2.33824900	1.87410000

Compound 10'
E(toluene)[M06-2X/Def2-TZVP, SMD (toluene)] = -1432.322401 a.u.
Gcorr[M06-2X/Def2-SVP] = 0.61663 a.u.
Hcorr[M06-2X/Def2-SVP] = 0.712196 a.u.
G = E(toluene) + Gcorr= -1431.705771 a.u.
H = E(toluene) + Hcorr= -1431.610205 a.u.

C	-4.07435500	-2.61462500	0.15753000
C	-2.86154300	-2.93655400	-0.31389000
C	-4.54576300	-1.22304400	0.05816100
C	-3.83971600	-0.22560600	-0.50246300
H	-5.55515700	-1.01255800	0.42632000
C	-2.46896800	-0.47257500	-1.08170800
C	-1.91126700	-1.92511900	-0.89024300
H	-2.51088600	-3.97352300	-0.26842200
C	-0.94532500	-1.34769600	0.19687000
C	-1.20700400	0.10298800	-0.32454300
H	-2.48772700	-0.14447200	-2.13741000
H	-1.51500500	-1.40998900	1.13596500
C	-1.42139200	1.05093600	0.87563100
C	-0.17878000	0.65481200	-1.30439100
H	-0.54673500	1.59628700	-1.74030100
H	0.79002300	0.84086300	-0.81797200
H	-0.00497400	-0.02538800	-2.14516800
C	-1.28488500	-2.51690600	-2.15147300
H	-0.67259000	-3.39688000	-1.90308800
H	-2.08565800	-2.83463700	-2.83630900
H	-0.64997400	-1.80619100	-2.69566500
C	-5.01281500	-3.61615200	0.76902900
H	-4.57417100	-4.62220000	0.78068700
H	-5.26269200	-3.33499100	1.80424700
H	-5.96061100	-3.65651900	0.20941600
C	-4.43085200	1.15020600	-0.65333500
H	-5.52144000	1.12844200	-0.52756900
H	-4.02948700	1.85407700	0.09259500
H	-4.20464700	1.56846100	-1.64737900
C	1.32740900	4.49813300	-1.35234200
H	1.87009700	3.58493500	-1.63311200
H	1.34893100	5.19459200	-2.20171700
H	1.86322000	4.95392600	-0.50810300
C	-1.49400900	2.57005200	0.56982600
C	-0.14926400	3.18221400	0.18912000
C	-0.10863800	4.18251400	-0.95923200
H	-0.63174300	3.70955800	-1.80770000
C	-0.89437300	5.44846100	-0.58818100
H	-1.95586200	5.23726200	-0.39720000
H	-0.46541000	5.92378500	0.30687700
H	-0.83946800	6.17117200	-1.41409300
O	0.85745700	2.92040800	0.81677700
C	0.44238100	-1.86483900	0.49949100
C	0.62363700	-2.30713100	1.92830400
H	0.39357200	-1.46069900	2.59455300
H	-0.11534300	-3.09585700	2.14712400
H	1.62966000	-2.68188200	2.13459800
C	1.40394200	-1.88242700	-0.43657400
H	1.12339100	-1.60456500	-1.45474400
C	2.88491800	-2.19202100	-0.32295100
O	3.43139900	-2.03902100	0.98327900
C	3.92953700	-0.72717100	1.20639800
C	4.21236400	-0.17670700	-0.18240700
C	3.60266600	-1.11263300	-1.12818900
H	4.87694400	-0.84077800	1.76005800
H	3.23716600	-0.81982400	-2.11818500
C	3.22002800	-3.60316600	-0.78813800
H	2.80475000	-3.79182300	-1.78839700

H	4.31170600	-3.72285900	-0.81096600
H	2.78886900	-4.33001900	-0.08619500
C	2.95644500	0.13082500	1.99944500
H	2.72178800	-0.35995100	2.95318600
H	3.38625300	1.12027100	2.21348100
H	2.01708000	0.26231500	1.44325900
C	4.43249100	1.28835700	-0.42078200
H	4.64411100	1.46095700	-1.48397000
H	3.54590500	1.86950600	-0.12792600
H	5.29158600	1.64957400	0.16443200
O	4.99690600	-1.09450900	-0.93169900
O	-0.46441100	0.79615800	1.87814300
H	0.29377500	1.38348100	1.73039300
H	-2.39539500	0.77785100	1.31897100
C	-2.02011700	3.29449100	1.81975500
H	-3.04921100	2.97104200	2.03408800
H	-1.39348600	3.02638200	2.68038800
H	-2.01746700	4.38641500	1.70806600
H	-2.18501400	2.73248300	-0.27141700

Compound 11
E(toluene)[M06-2X/Def2-TZVP, SMD (toluene)] = -1432.330963 a.u.
Gcorr[M06-2X/Def2-SVP] = 0.615135 a.u.
Hcorr[M06-2X/Def2-SVP] = 0.711314 a.u.
G = E(toluene) + Gcorr= -1432.330963 a.u.
H = E(toluene) + Hcorr= -1431.619649 a.u.

C	1.03013500	3.18950300	-0.93017300
C	1.17603600	2.37464000	0.12772600
C	-0.27474600	3.81542700	-1.22112200
C	-1.38832700	3.51863100	-0.53110200
H	-0.32010700	4.53702600	-2.04246300
C	-1.31831700	2.50340800	0.57444900
C	0.07813300	2.11028600	1.12442600
H	2.15846700	1.93919800	0.34054300
C	-0.42824000	0.62914700	1.20487300
C	-1.61593000	0.99898900	0.25071500
H	-1.97748800	2.82139700	1.40014600
H	-0.87465000	0.52340700	2.20861400
C	-2.97755500	0.53490400	0.79380200
C	-1.44849700	0.64414400	-1.22027800
H	-2.34762800	0.94583600	-1.77705700
H	-0.59148200	1.16031100	-1.67041800
H	-1.30153900	-0.43782500	-1.35887600
C	0.47809600	2.71703500	2.46625300
H	0.64708100	3.79953500	2.36367300
H	-0.30862600	2.55692800	3.22009900
H	1.41013100	2.26077500	2.83811100
C	2.16239400	3.50530200	-1.86718000
H	1.90631600	3.21622500	-2.89912000
H	2.36531200	4.58765700	-1.88217300
H	3.08435600	2.98308800	-1.57747500
C	-2.72920400	4.12416800	-0.81066600
H	-3.48200600	3.32901000	-0.92193200
H	-3.05573200	4.73655200	0.04603700
H	-2.71401300	4.75695500	-1.70788500
C	-3.85968700	-3.88711200	-1.43979100
H	-4.50218800	-3.39611400	-2.18070400
H	-3.42386600	-4.79115900	-1.88665300
H	-4.48464200	-4.19649500	-0.58753500
C	-3.13507700	-0.99924800	0.80137000
C	-3.38735600	-1.59861200	-0.57408500
C	-2.75406500	-2.93208800	-0.97773600
H	-2.14771200	-2.67291300	-1.86441200

C	-1.84277500	-3.56564700	0.06778200
H	-0.99051600	-2.91733800	0.31829200
H	-2.39283900	-3.79115300	0.99509000
H	-1.43839400	-4.51326700	-0.31613700
O	-4.15868500	-1.06130900	-1.34357700
C	0.51367500	-0.54204500	1.02237500
C	0.63771700	-1.43719400	2.23044700
H	-0.35863300	-1.80973400	2.52393600
H	1.31116500	-2.28308300	2.06448900
H	1.01027300	-0.85481900	3.09063300
C	1.21365100	-0.70350000	-0.11199800
H	1.02410700	0.00927800	-0.91807000
C	2.28855800	-1.71641300	-0.45942200
O	3.02981600	-2.20429800	0.65753300
C	4.20905300	-1.44967400	0.89134300
C	4.52313500	-0.79067600	-0.44262800
C	3.34050800	-0.98354700	-1.28467100
H	5.00194500	-2.17334300	1.14508800
H	3.04536300	-0.29808100	-2.08589200
C	1.72637400	-2.93401800	-1.18340100
H	1.13053100	-3.53211500	-0.48088200
H	1.08409400	-2.63012200	-2.02273800
H	2.56088600	-3.54437600	-1.55445800
C	4.02899500	-0.45235600	2.02621300
H	4.97495200	0.05874000	2.25707700
H	3.27001300	0.30102100	1.76564900
H	3.69441000	-0.98167300	2.92841100
C	5.50947000	0.33372500	-0.55986200
H	6.50030200	0.01569200	-0.20240400
H	5.60240500	0.63207600	-1.61185000
H	5.19031900	1.20281300	0.03184400
O	4.50493400	-1.74961500	-1.48868000
C	-4.32793500	-1.41712800	1.67649100
H	-4.49648900	-2.50398900	1.65135100
H	-4.15483000	-1.11960400	2.72007400
H	-5.23481900	-0.91368100	1.31482800
H	-2.21348200	-1.42218200	1.22301700
O	-4.05095400	1.18788500	0.17064700
H	-4.32625200	0.64371800	-0.58526300
H	-3.00468900	0.84423200	1.85510700

Compound 11'
E(toluene)[M06-2X/Def2-TZVP, SMD (toluene)] = -1432.329163 a.u.
Gcorr[M06-2X/Def2-SVP] = 0.615573 a.u.
Hcorr[M06-2X/Def2-SVP] = 0.711598 a.u.
G = E(toluene) + Gcorr= -1431.71359 a.u.
H = E(toluene) + Hcorr= -1431.617565 a.u.

C	2.20790900	-3.63237900	0.88256700
C	0.99235300	-3.18996100	0.52833000
C	3.40761000	-3.08598800	0.22422300
C	3.37581100	-2.15788200	-0.74915300
H	4.37189800	-3.51976500	0.50687300
C	2.06712300	-1.56230500	-1.20461400
C	0.79145800	-2.07777800	-0.46133500
H	0.09325200	-3.62160100	0.98302600
C	0.69256600	-0.67814100	0.23967700
C	1.70870800	-0.07835200	-0.78695300
H	2.00932600	-1.68505300	-2.30048800
C	2.83178200	0.71639700	-0.08335300
C	1.14579500	0.70469400	-1.96913400
H	0.56760000	1.58761000	-1.65967100
H	1.98255000	1.04261100	-2.59701300

H	0.49129300	0.08626600	-2.59730500
H	1.27008100	-0.79682500	1.17128100
C	2.42025700	-4.71271600	1.90514800
H	2.95173900	-5.56929300	1.46144400
H	3.04410700	-4.34545600	2.73515900
H	1.46813700	-5.07095300	2.31796200
C	4.61067900	-1.75653600	-1.50266500
H	4.77500100	-0.67159800	-1.45892300
H	5.49732300	-2.27793600	-1.11809100
H	4.49231800	-2.01648500	-2.56813900
C	-0.33985400	-2.44471600	-1.41650000
H	-1.28687400	-2.59550400	-0.87619500
H	-0.51119500	-1.67936400	-2.18539300
H	-0.08314100	-3.38335400	-1.93035500
C	2.32339300	1.85797000	0.83093800
C	1.74974400	3.01901600	0.03130100
O	2.40577000	3.54007100	-0.84840500
C	0.35997600	3.51035500	0.38197700
H	-0.28215400	2.61179100	0.33462700
C	-0.12575900	4.55379800	-0.61207000
H	-1.14648400	4.87711200	-0.36334900
H	0.53531900	5.43206600	-0.60045100
H	-0.12148800	4.15712000	-1.63713700
C	0.32510900	4.02725000	1.82681300
H	0.96841800	4.91360000	1.93712400
H	-0.70091900	4.31829100	2.09321200
H	0.65549200	3.26732400	2.54952800
C	-0.64956500	-0.09692200	0.62722600
C	-1.55916100	0.25987200	-0.29473000
H	-1.26046300	0.19933100	-1.34409000
C	-0.88419000	-0.03963200	2.11681100
H	-0.09045600	0.55772000	2.59600200
H	-1.86062600	0.37591800	2.37911500
H	-0.79894500	-1.05353100	2.54357500
C	-2.97967900	0.77009100	-0.13313700
O	-3.62514000	0.38989600	1.07902800
C	-4.39623600	-0.79315000	0.93312500
C	-4.68973900	-0.88854400	-0.55559300
C	-3.82767700	0.09924200	-1.20815700
H	-5.33525200	-0.62905200	1.48812200
H	-3.45694700	0.00094100	-2.23361000
C	-3.04531100	2.29198600	-0.21042700
H	-4.09869100	2.59978900	-0.25780300
H	-2.58582300	2.72273600	0.69115900
H	-2.50749100	2.66546300	-1.09403000
C	-3.66950300	-2.01180000	1.48094400
H	-4.30704500	-2.90666200	1.43553900
H	-2.74618100	-2.19990500	0.91181800
H	-3.39775200	-1.83620600	2.53028900
C	-5.21122500	-2.14835700	-1.18164100
H	-5.39920500	-1.97606500	-2.24885300
H	-4.49101500	-2.97088100	-1.07361500
H	-6.15773700	-2.45003900	-0.70877300
O	-5.20592700	0.34021200	-1.04372300
O	3.80512000	1.19021300	-0.97859900
H	3.56072000	2.09330000	-1.24042000
H	3.34055100	0.00165700	0.58873000
H	1.53587900	1.45485700	1.48326400
C	3.47764000	2.38785100	1.69116200
H	3.15689200	3.21953700	2.33465700
H	3.87192900	1.58571800	2.33057300
H	4.28738600	2.74146000	1.03953400

TS5

E(toluene)[M06-2X/Def2-TZVP, SMD (toluene)] = -1392.967851 a.u.

Gcorr[M06-2X/Def2-SVP] = 0.586494 a.u.
Hcorr[M06-2X/Def2-SVP] = 0.680115 a.u.
G = E(toluene) + Gcorr= -1392.381357 a.u.
H = E(toluene) + Hcorr= -1392.287736 a.u.

C	-0.63138800	3.17271700	0.97605100
C	0.06742100	3.25170900	-0.23881400
C	-1.96471900	2.72711800	1.04959100
C	-2.66817800	2.22028200	-0.04255100
H	-2.53203000	3.06453000	1.92367300
C	-1.97963500	1.56181800	-1.07165200
C	-0.07409800	2.53309600	-1.44032700
H	0.70151000	4.14601400	-0.30966600
C	0.13505200	1.04773100	-1.73285800
C	-0.98983900	0.40482200	-0.89476800
H	-2.51967600	1.48655400	-2.02748100
C	-1.57406000	-0.85874000	-1.58494900
C	-0.56425600	0.07503200	0.53611000
H	0.13947000	0.80830500	0.94181400
H	-1.42201900	0.03639300	1.22064300
H	-0.06020900	-0.90046500	0.55012900
H	-0.18031400	0.92298000	-2.78143600
C	-0.04492300	3.87304200	2.17661800
H	-0.82366200	4.25203300	2.85242200
H	0.58302400	3.17622400	2.75574200
H	0.59987100	4.71061900	1.87363800
C	-4.13771900	2.53487300	-0.20900800
H	-4.50963700	3.18340600	0.59491800
H	-4.30499500	3.05573900	-1.16490800
H	-4.75589400	1.62172500	-0.22801100
C	-2.96948700	-1.28953000	-1.07161400
C	-2.90400500	-1.80496900	0.35811800
O	-2.15520800	-2.71694300	0.64305100
C	-3.82301100	-1.17446600	1.38792700
H	-3.67835300	-0.08231700	1.28997000
C	-3.46818000	-1.62976800	2.79527200
H	-2.42583800	-1.38220000	3.03986500
H	-4.12688100	-1.15198700	3.53355400
H	-3.57400800	-2.72038300	2.88048100
C	-5.28218000	-1.49023000	1.02907000
H	-5.55018700	-1.13307600	0.02408900
H	-5.46209000	-2.57525900	1.06922700
H	-5.95819500	-1.00794300	1.74872200
C	1.55643900	0.51041600	-1.64812000
C	2.35985600	0.88999600	-0.64539300
H	1.97953500	1.66659600	0.02867900
C	1.92336900	-0.44542300	-2.75280100
H	1.80696600	0.07103600	-3.72016100
H	2.94984700	-0.81023500	-2.66262500
H	1.22627700	-1.29734800	-2.75203300
C	3.75256300	0.42530700	-0.26926700
O	4.12496400	-0.84821800	-0.78616000
C	3.83000700	-1.90476000	0.11659200
C	3.75494400	-1.23899800	1.48205000
C	3.74981600	0.20445400	1.23952300
H	4.69427300	-2.59008600	0.09254900
H	3.28058500	0.93255100	1.90969800
C	4.81859100	1.41818000	-0.71770700
H	4.86536600	1.43029900	-1.81509300
H	4.58260300	2.43202100	-0.36490500
H	5.79054300	1.10191800	-0.31551900
C	2.56281400	-2.65074900	-0.27165900
H	1.69303300	-1.97776700	-0.28275700
H	2.67349900	-3.06575300	-1.28225600
H	2.37006000	-3.48286700	0.42151000

C	3.15095600	-1.92743800	2.67001100
H	3.66598900	-2.87917700	2.86911300
H	3.25328000	-1.28767800	3.55578300
H	2.08586100	-2.13731300	2.50082100
O	4.91025300	-0.44257600	1.70724200
O	-0.67212800	-1.93255100	-1.57370300
H	-0.85163200	-2.46931700	-0.78453500
H	-1.72033900	-0.58718500	-2.64684100
H	-3.62995800	-0.40993700	-1.10169900
C	-3.53954800	-2.39447500	-1.96894500
H	-3.63839700	-2.03074700	-3.00139800
H	-2.86099700	-3.25730200	-1.97050000
H	-4.52788300	-2.72798000	-1.62196700
H	0.22893700	3.14139600	-2.30570700

TS5'
E(toluene)[M06-2X/Def2-TZVP, SMD (toluene)] = -1392.972908 a.u.
Gcorr[M06-2X/Def2-SVP] = 0.585918 a.u.
Hcorr[M06-2X/Def2-SVP] = 0.680453 a.u.
G = E(toluene) + Gcorr= -1392.38699 a.u.
H = E(toluene) + Hcorr= -1392.292455 a.u.

C	1.91923400	4.03981500	-0.16026900
C	0.90524200	3.68213200	-1.05526500
C	2.92264700	3.11100100	0.17842900
C	3.13846400	1.86881300	-0.44429700
H	3.78861100	3.54067000	0.69348000
C	2.14804200	1.02423400	-0.99880700
C	0.45274800	2.36784700	-1.16000000
H	0.56126700	4.43789500	-1.76903800
C	0.16252500	1.55588100	0.09364500
C	1.03094500	0.32391600	-0.20979700
H	2.53912600	0.39263800	-1.81304200
H	0.65232200	2.08017700	0.92620000
C	1.55296300	-0.34502100	1.08160500
C	0.32446400	-0.67384200	-1.12548200
H	1.03512100	-1.39531300	-1.55586100
H	-0.45469600	-1.22886200	-0.58565800
H	-0.14954300	-0.15982200	-1.97285900
C	2.10404200	5.48564400	0.22507000
H	1.75704000	6.15029000	-0.57921200
H	1.50537300	5.72397000	1.11816100
H	3.15228500	5.72384200	0.44981000
C	4.58887100	1.48977700	-0.68522000
H	5.24896900	2.36319400	-0.76875400
H	4.97160500	0.86024300	0.13664600
H	4.67790600	0.89566800	-1.60699200
C	2.42479500	-4.23796500	-1.71821300
H	1.41819200	-3.95409400	-2.05481200
H	3.01354500	-4.55461500	-2.59013300
H	2.31179100	-5.09265400	-1.03681400
C	2.72016900	-1.34294400	0.89957100
C	2.28678400	-2.61419200	0.18828700
C	3.10831300	-3.08338000	-1.00132300
H	3.19015500	-2.21015900	-1.67463400
C	4.52690400	-3.44673900	-0.54045500
H	5.03740800	-2.59901400	-0.06137700
H	4.49692800	-4.28308200	0.17416500
H	5.13156400	-3.75975600	-1.40288900
O	1.33076800	-3.24832200	0.58524200
C	-1.29746400	1.38003100	0.48070400
C	-1.55926300	1.53390800	1.95526800
H	-0.97624900	0.77923200	2.50559400
H	-1.19861100	2.52536400	2.27542400

H	-2.61767200	1.43390800	2.20823900
C	-2.22836300	1.12268800	-0.44887300
H	-1.91065900	1.10616800	-1.49733200
C	-3.71505800	0.86050000	-0.29462100
O	-4.10373600	0.35378900	0.97815100
C	-4.13418300	-1.06538000	1.00725800
C	-4.30335200	-1.47908500	-0.44658200
C	-4.07719500	-0.27411500	-1.24663000
H	-5.03472500	-1.34921600	1.57794900
H	-3.69514700	-0.28765000	-2.27275300
C	-4.53668200	2.11997900	-0.54308600
H	-4.27284800	2.57548300	-1.50792200
H	-5.60317000	1.85698700	-0.53642600
H	-4.33523600	2.84831100	0.25421600
C	-2.89355000	-1.65014300	1.66479300
H	-2.78961400	-1.24563800	2.68041600
H	-2.96542400	-2.74559900	1.73601900
H	-1.98378900	-1.38142100	1.10781900
C	-4.07194800	-2.88984300	-0.90082700
H	-4.29619500	-2.97308000	-1.97182200
H	-3.02963700	-3.19122200	-0.72765700
H	-4.72883500	-3.58350300	-0.35495900
O	-5.38025200	-0.76980900	-1.04243800
O	0.50239600	-0.91532900	1.81937200
H	0.41363900	-1.84324600	1.54802200
H	1.96136700	0.47888600	1.69494700
C	3.29167700	-1.72013100	2.27302300
H	3.69140000	-0.82566600	2.77157900
H	2.49315100	-2.13674400	2.90071700
H	4.09789600	-2.46272400	2.19215400
H	3.49961900	-0.85120800	0.29979700
H	-0.14826700	2.11842300	-2.04045900

TS6
E(toluene)[M06-2X/Def2-TZVP, SMD (toluene)] = -1392.968009 a.u.
Gcorr[M06-2X/Def2-SVP] = 0.586359 a.u.
Hcorr[M06-2X/Def2-SVP] = 0.680583 a.u.
G = E(toluene) + Gcorr= -1392.38165 a.u.
H = E(toluene) + Hcorr= -1392.287426 a.u.

C	0.48260400	2.98356700	0.20266400
C	-0.37261200	2.34076800	-0.70661500
C	1.87702400	2.79733500	0.16488400
C	2.50088300	1.88643400	-0.68940300
H	2.47735700	3.60887100	0.59189900
C	1.84211800	0.70364900	-1.04839500
C	-0.23695400	1.11812200	-1.39099300
H	-1.17783200	2.99345500	-1.07214400
C	-0.17623700	-0.30242300	-0.83228500
C	1.17603800	-0.29425900	-0.09434200
H	2.22557500	0.20270000	-1.94382600
H	-0.01282100	-0.95648800	-1.70337200
C	1.87070100	-1.68682800	-0.15813800
C	1.03269200	0.11522600	1.37076900
H	0.23313000	0.84983700	1.51555700
H	0.76949400	-0.77186000	1.97035000
H	1.96121600	0.54491900	1.76971500
C	-0.06479100	4.13590600	1.00753200
H	-0.92412200	4.60162800	0.50323400
H	-0.41938100	3.78236600	1.98919300
H	0.69711900	4.90640100	1.18992800
C	3.75235100	2.30934500	-1.42607900
H	3.48601600	2.82815100	-2.36169500
H	4.33819800	3.02139200	-0.82506200

H	4.38587500	1.45000600	-1.68526800
C	4.90434700	0.67688400	1.70104000
H	5.71497400	1.07241300	2.32903900
H	4.51953900	1.49714500	1.07797900
H	4.09638600	0.34914000	2.36949700
C	3.22031100	-1.79900700	0.58365900
C	4.35861400	-1.03314000	-0.07450300
C	5.44353000	-0.46542400	0.82921900
H	5.72254000	-1.29455100	1.50480000
C	6.66080300	-0.03287700	0.02266300
H	7.02505400	-0.84493200	-0.61992700
H	6.40532000	0.81512200	-0.62839500
H	7.47218300	0.27985800	0.69423600
O	4.44702800	-0.96413100	-1.28406100
C	-1.40274700	-0.83388100	-0.11743700
C	-1.40147600	-2.32685000	0.11769300
H	-0.88115400	-2.84837900	-0.69984200
H	-0.85880100	-2.56827200	1.04735000
H	-2.42021900	-2.71814000	0.21196700
C	-2.40869200	-0.03484400	0.26821300
H	-2.30935200	1.03716200	0.07702200
C	-3.68709000	-0.45248700	0.97762300
O	-4.33100200	-1.57914000	0.37539400
C	-5.28756500	-1.18336000	-0.59285000
C	-5.70332500	0.21973400	-0.17713100
C	-4.71324900	0.65913000	0.80965200
H	-6.13867200	-1.87825400	-0.49668200
H	-4.43427200	1.70573800	0.96927400
C	-3.45061000	-0.80253800	2.44138700
H	-4.41795400	-1.00947100	2.91880500
H	-2.81478500	-1.69541200	2.51262300
H	-2.94828900	0.02641700	2.95973400
C	-4.72210200	-1.23207400	-2.00534000
H	-4.29143300	-2.22514800	-2.19179800
H	-5.50766600	-1.04794300	-2.75273300
H	-3.92527200	-0.48378000	-2.13193000
C	-6.49613100	1.11266300	-1.08555500
H	-6.70892600	2.06015000	-0.57461600
H	-5.94681500	1.32123000	-2.01351100
H	-7.45424700	0.63962100	-1.34786400
O	-6.00870500	0.27121200	1.20698800
C	3.65312700	-3.27439900	0.64409300
H	2.90998800	-3.85898500	1.20403200
H	3.71407700	-3.68278700	-0.37287800
H	4.62811900	-3.39826300	1.13666600
H	3.09399800	-1.43295200	1.61168400
O	1.96767100	-2.18285500	-1.46785400
H	2.78578000	-1.82780800	-1.84876200
H	1.19180700	-2.37292000	0.37331100
H	-0.77103100	1.13570200	-2.35349400

TS6’
E(toluene)[M06-2X/Def2-TZVP, SMD (toluene)] = -1392.972678 a.u.
Gcorr[M06-2X/Def2-SVP] = 0.584352 a.u.
Hcorr[M06-2X/Def2-SVP] = 0.679529 a.u.
G = E(toluene) + Gcorr= -1392.388326 a.u.
H = E(toluene) + Hcorr= -1392.293149 a.u.

C	1.58729300	-3.78495300	0.62720900
C	0.50182300	-3.41457300	-0.18427400
C	2.85103500	-3.20355600	0.42780800
C	3.22691200	-2.38653200	-0.65912400
H	3.67389000	-3.70214500	0.95060300
C	2.41382500	-1.47285600	-1.35130800

C	0.39646500	-2.13870300	-0.72222000
H	-0.18984700	-4.20220500	-0.50248400
C	0.68882000	-0.90879600	0.12810300
C	1.75838500	-0.22631800	-0.73310000
H	2.76558000	-1.28562200	-2.37628400
C	2.82832000	0.53091900	0.08995900
C	1.21359300	0.62282900	-1.88124200
H	0.57507300	1.44704800	-1.53870700
H	2.05610900	1.04821600	-2.44543900
H	0.62271700	0.00579500	-2.57447000
H	1.20781400	-1.26821700	1.02941500
C	1.46954700	-4.99346700	1.52132500
H	2.44207500	-5.47060400	1.70101000
H	1.04548200	-4.71293800	2.49833700
H	0.78979200	-5.73770700	1.08060600
C	4.60999500	-2.60910400	-1.23911900
H	5.21021800	-1.69116700	-1.15039700
H	5.13928100	-3.43896000	-0.75329900
H	4.52806100	-2.83788900	-2.31337700
C	2.32079400	1.76211400	0.87057400
C	1.94104400	2.91163600	-0.04927500
O	2.72296700	3.31293800	-0.88688900
C	0.57583300	3.54521800	0.13768400
H	-0.13940100	2.70333000	0.11858800
C	0.26313500	4.52682100	-0.98072100
H	-0.74217800	4.95246000	-0.85165200
H	0.99397700	5.34798700	-0.98612400
H	0.31739300	4.03665200	-1.96293900
C	0.47514400	4.19230200	1.52561900
H	1.20738200	5.00737600	1.63100100
H	-0.52853400	4.61939800	1.66298700
H	0.64344800	3.46662000	2.33467500
C	-0.57229400	-0.19663800	0.60182500
C	-1.51214700	0.20995900	-0.26585900
H	-1.30916400	0.08550200	-1.33474800
C	-0.73379400	-0.13885100	2.09923000
H	0.17783100	0.25816000	2.57321700
H	-1.60396100	0.44082900	2.42052100
H	-0.85193100	-1.16765000	2.48121500
C	-2.87808100	0.79637500	0.05131500
O	-3.50634500	0.18012800	1.17509700
C	-4.33482800	-0.90568900	0.78814900
C	-4.71318700	-0.61120200	-0.65550900
C	-3.81596100	0.45772000	-1.10057000
H	-5.23062000	-0.86216600	1.42993000
H	-3.49999200	0.59782200	-2.13951600
C	-2.83415600	2.29465300	0.32845900
H	-3.86016300	2.65525700	0.48113900
H	-2.24165600	2.49210700	1.23362800
H	-2.37632800	2.83174300	-0.51529000
C	-3.63422400	-2.24704800	0.95406900
H	-4.32685600	-3.07892300	0.75944500
H	-2.77383900	-2.33117200	0.27220700
H	-3.26541300	-2.34064500	1.98425300
C	-5.35135800	-1.64523200	-1.53535200
H	-5.56865400	-1.20757900	-2.51789400
H	-4.69059800	-2.51240400	-1.66814500
H	-6.29804400	-1.99344800	-1.09596700
O	-5.16442500	0.72713400	-0.79667900
O	3.95149200	0.83688200	-0.69553700
H	3.82869000	1.72927900	-1.05609900
H	3.17036000	-0.18951800	0.85476100
H	1.42925000	1.45991600	1.43889000
C	3.40994000	2.24737600	1.83638000
H	3.09519000	3.14086500	2.39380800
H	3.65368700	1.45521100	2.55808700

H	4.31843500	2.48901700	1.26948700
H	-0.33530300	-1.98273200	-1.52312400

TS7
E(toluene)[M06-2X/Def2-TZVP, SMD (toluene)] = -1392.981055 a.u.
Gcorr[M06-2X/Def2-SVP] = 0.583646 a.u.
Hcorr[M06-2X/Def2-SVP] = 0.679295 a.u.
G = E(toluene) + Gcorr= -1392.397409 a.u.
H = E(toluene) + Hcorr= -1392.30176 a.u.

C	2.54071800	-1.88406300	1.46796800
C	2.03944100	-2.77143900	0.50157500
C	3.39556800	-0.81451800	1.12216200
C	3.69961700	-0.46856900	-0.19932000
H	4.05652900	-0.46069700	1.92110200
C	2.71942400	-0.65624800	-1.18209200
C	1.69034600	-2.52794600	-0.84069300
H	2.08038300	-3.82709600	0.80551100
C	0.55827600	-1.52928400	-1.16901800
C	1.28173800	-0.22252500	-0.94498700
H	3.04429300	-0.60912500	-2.23158700
C	0.83755200	0.94716600	-1.84431700
H	0.34480400	-1.62515700	-2.25064900
C	2.46061200	-2.29388100	2.91777600
H	2.44100100	-3.38860600	3.01961100
H	3.29978000	-1.89764800	3.50559700
H	1.52830000	-1.91456600	3.36456000
C	5.11624200	-0.09498500	-0.56513400
H	5.66736800	-0.97936300	-0.92447500
H	5.15599800	0.66654800	-1.35642900
H	5.65741400	0.28532600	0.31251600
C	1.83873800	-3.67238600	-1.81498900
H	0.87996000	-4.19684300	-1.95934300
H	2.13802100	-3.28674300	-2.80100200
H	2.59593600	-4.39644200	-1.48646000
C	1.38924000	2.29774300	-1.33081200
C	0.89223700	2.49865400	0.09478600
O	-0.30089100	2.49633300	0.32908300
C	1.92027700	2.66984300	1.19392500
H	2.67678900	1.88409800	1.01889300
C	1.29817700	2.49161400	2.57028200
H	0.84186800	1.49532300	2.66740700
H	2.05961500	2.60239900	3.35477500
H	0.50843900	3.23759400	2.73673700
C	2.60251500	4.03536200	1.02639700
H	3.06795700	4.14449800	0.03597400
H	1.87317700	4.84891700	1.15721700
H	3.38924400	4.15824900	1.78346900
C	-0.74343900	-1.78405600	-0.43385600
C	-1.25035200	-0.86158100	0.39365600
H	-0.66733500	0.04411600	0.57902700
C	-1.39244200	-3.11021400	-0.72444000
H	-0.75613200	-3.92342500	-0.33680900
H	-2.38606300	-3.18795100	-0.27340300
H	-1.48612100	-3.26332700	-1.81238400
C	-2.58815200	-0.84560600	1.10954800
O	-3.64235000	-1.46071300	0.37298000
C	-4.29624400	-0.53256000	-0.48121700
C	-4.04671200	0.82434800	0.16037700
C	-3.01216000	0.61711700	1.17699900
H	-5.37119200	-0.77584400	-0.43961500
H	-2.29908200	1.39238000	1.47724800
C	-2.53579300	-1.51712200	2.47478500
H	-1.73529800	-1.08050700	3.08879800

H	-3.50348600	-1.38123500	2.97644000
H	-2.33589300	-2.59066700	2.35211000
C	-3.79294400	-0.61510700	-1.91610800
H	-2.74444600	-0.28987600	-1.99487900
H	-3.86246600	-1.65466700	-2.26491100
H	-4.40841000	0.01002900	-2.57988400
C	-4.29383600	2.10882400	-0.57451600
H	-5.34676500	2.18224600	-0.88559200
H	-4.06549000	2.95650700	0.08372100
H	-3.66122000	2.17395700	-1.47088100
O	-4.36127100	0.79027400	1.54450200
O	-0.55115700	0.99675000	-2.03777600
H	-0.95337700	1.31597800	-1.21544700
H	1.26657900	0.77586200	-2.84792600
H	2.48869900	2.22431800	-1.31364800
C	0.94864100	3.45670400	-2.22530000
H	-0.14651700	3.52275700	-2.23216500
H	1.35836000	4.41473100	-1.87493900
H	1.28830200	3.29032800	-3.25734900
H	1.20210400	0.05076800	0.11813100

TS7’

E(toluene)[M06-2X/Def2-TZVP, SMD (toluene)] = -1392.979439 a.u.
Gcorr[M06-2X/Def2-SVP] = 0.583622 a.u.
Hcorr[M06-2X/Def2-SVP] = 0.679543 a.u.
G = E(toluene) + Gcorr= -1392.395817 a.u.
H = E(toluene) + Hcorr= -1392.299896 a.u.

C	3.84613300	-2.42255800	-0.35975000
C	3.15609800	-2.49003900	0.85255900
C	4.07579300	-1.18133800	-0.99117500
C	3.83674300	0.07671900	-0.41670700
H	4.80881700	-1.20715000	-1.80391900
C	2.79567300	0.38424900	0.48827600
C	2.16193400	-1.57192200	1.21096900
H	3.50150000	-3.22022100	1.59466400
C	1.09526400	-1.19620700	0.16732900
C	1.31245100	0.29554500	0.12983500
H	3.03989300	1.23215300	1.14790600
H	1.41122400	-1.61831700	-0.80104000
C	0.93384700	0.99917700	-1.18765200
C	1.71495800	-1.50350200	2.65002400
H	0.99865600	-2.31315400	2.86827300
H	2.56809100	-1.59501900	3.33462100
H	1.19192000	-0.55985700	2.86281600
C	4.59143800	-3.63493500	-0.85970600
H	4.87802100	-4.28856800	-0.02348400
H	3.94852400	-4.23032600	-1.52681800
H	5.49605300	-3.36419700	-1.42042000
C	4.87986700	1.15225000	-0.65135900
H	5.76446500	0.76868200	-1.17559500
H	4.47189900	1.99469100	-1.23287600
H	5.20944400	1.56240900	0.31646000
C	-0.58656100	3.82773700	2.34420100
H	-0.86309800	2.82643400	2.70260900
H	-0.19825200	4.40891200	3.19178000
H	-1.50341800	4.30976400	1.97717100
C	0.93481900	2.53647900	-1.02865400
C	-0.05276900	2.91145600	0.06875000
C	0.44829800	3.74687400	1.23215900
H	1.35681800	3.23138000	1.59588200
C	0.86829100	5.13384400	0.72527600
H	1.63948500	5.07413800	-0.05579600
H	0.00015400	5.67041700	0.31387900

H	1.27342700	5.73005700	1.55436800
O	-1.20482200	2.52871300	0.01399400
C	-0.28719200	-1.74344600	0.46343000
C	-0.40420200	-3.23004500	0.26607300
H	-0.21669200	-3.48045300	-0.79106600
H	0.38099200	-3.74095800	0.84795600
H	-1.38847300	-3.61689600	0.54523400
C	-1.28495700	-0.93224700	0.83507700
H	-1.05864700	0.12853900	0.98350600
C	-2.75817500	-1.23753600	1.02156200
O	-3.23227000	-2.30676300	0.20950500
C	-3.70346900	-1.84679500	-1.05049600
C	-4.05984700	-0.38563900	-0.82207800
C	-3.50794800	-0.02268800	0.48561300
H	-4.61800700	-2.42308100	-1.26999700
H	-3.18519500	0.99210400	0.74280700
C	-3.11259300	-1.57078900	2.46456200
H	-2.76085800	-0.78077500	3.14306000
H	-4.20299600	-1.67253300	2.54900100
H	-2.63524600	-2.51785000	2.75225000
C	-2.67859000	-2.04581800	-2.15812800
H	-2.36868900	-3.09940000	-2.17972600
H	-3.11498600	-1.79919200	-3.13743200
H	-1.78827500	-1.41793600	-1.99989200
C	-4.28337200	0.56528200	-1.96095500
H	-4.55337100	1.55260600	-1.56546800
H	-3.37459800	0.66166700	-2.57159200
H	-5.10129300	0.21271200	-2.60706500
O	-4.88917000	-0.24594300	0.32237100
O	-0.27630700	0.53960300	-1.73004900
H	-0.99649400	0.86824600	-1.17084900
H	1.70842700	0.73630700	-1.92851700
C	0.56467800	3.23192500	-2.34006000
H	1.27222100	2.94568100	-3.13093700
H	-0.44001100	2.91966600	-2.65140100
H	0.58053000	4.32637300	-2.23829500
H	1.94609900	2.84095900	-0.71292700
H	0.76281600	0.75091000	0.97043800

TS8
E(toluene)[M06-2X/Def2-TZVP, SMD (toluene)] = -1392.977661 a.u.
Gcorr[M06-2X/Def2-SVP] = 0.584687 a.u.
Hcorr[M06-2X/Def2-SVP] = 0.680114 a.u.
G = E(toluene) + Gcorr= -1392.392974 a.u.
H = E(toluene) + Hcorr= -1392.297547 a.u.

C	-0.70732000	2.71131600	-0.69596400
C	0.23143600	2.29568100	0.26062500
C	-2.09546800	2.54866200	-0.50280300
C	-2.63133800	1.82620000	0.56353700
H	-2.73901100	3.25437900	-1.03967700
C	-1.90090100	0.74310700	1.06763200
C	0.19046300	1.25843400	1.22160900
H	1.04886400	3.01939600	0.39659900
C	0.14266700	-0.22461000	0.84855500
C	-1.22429200	-0.28300100	0.17725800
H	-2.21272600	0.33783800	2.03895300
H	0.01692700	-0.78354100	1.79321400
C	-1.87358100	-1.67519800	0.15200600
C	0.93919100	1.51752500	2.51021800
H	0.37670700	1.10018300	3.35857600
H	1.91707400	1.00620000	2.49209700
H	1.09595200	2.58798800	2.69684600
C	-0.24439200	3.66182400	-1.77366600

H	0.63453500	4.23634600	-1.44662200
H	0.05323000	3.10346400	-2.67506600
H	-1.03581600	4.36536400	-2.06660100
C	-3.84943500	2.33108500	1.29863000
H	-3.54473700	2.78039700	2.25782900
H	-4.36609700	3.11423400	0.72583600
H	-4.55495100	1.51811000	1.52448700
C	-4.98066000	0.73565100	-1.69602600
H	-5.70096000	0.99239200	-2.48553300
H	-4.92711300	1.57781900	-0.99330300
H	-3.98931900	0.62287800	-2.15754500
C	-3.18274700	-1.76600500	-0.65346700
C	-4.36136700	-1.03980500	-0.02426700
C	-5.44249100	-0.53575200	-0.97004700
H	-5.56566700	-1.32478600	-1.73234300
C	-6.75874800	-0.31622300	-0.23649300
H	-7.09534300	-1.23278700	0.26644800
H	-6.63914800	0.45861100	0.53366900
H	-7.53971900	0.00798400	-0.93826300
O	-4.47751200	-0.94559000	1.18059500
C	1.34893500	-0.82900600	0.15216400
C	1.36023300	-2.34135500	0.10345600
H	0.76821900	-2.76396900	0.92849400
H	0.90733000	-2.70216500	-0.83469800
H	2.38396400	-2.72965800	0.14184000
C	2.33487500	-0.07782900	-0.36001500
H	2.23224500	1.00927700	-0.29975500
C	3.58875500	-0.57050500	-1.06573800
O	4.25957200	-1.62816000	-0.37357700
C	5.26310100	-1.13775300	0.49901900
C	5.65130200	0.21669200	-0.07491200
C	4.61683000	0.55207400	-1.05686800
H	6.11310000	-1.83700300	0.42789700
H	4.32676000	1.57659300	-1.31132500
C	3.30093900	-1.07417900	-2.47473600
H	4.25137700	-1.33419800	-2.95975600
H	2.66443300	-1.96780700	-2.42720100
H	2.78106900	-0.30380300	-3.06111700
C	4.77035500	-1.04911700	1.93613600
H	4.36816200	-2.02278400	2.24633600
H	5.58667100	-0.77684100	2.62081100
H	3.96645400	-0.30292900	2.02243000
C	6.48128500	1.19824500	0.69905500
H	6.67130900	2.08485600	0.08109000
H	5.97250600	1.50756100	1.62198100
H	7.45029300	0.75260100	0.96913700
O	5.89460900	0.12425800	-1.46936900
C	-3.59568200	-3.24068900	-0.80400700
H	-2.82621300	-3.78990700	-1.36403700
H	-3.68440200	-3.69614100	0.19094100
H	-4.55124000	-3.35366400	-1.33505100
H	-3.00639400	-1.34246800	-1.65496100
O	-2.01459200	-2.21387700	1.44317700
H	-2.81677500	-1.82143800	1.82122300
H	-1.16655400	-2.33521100	-0.37363000
H	-1.15326800	0.10077200	-0.85222600

TS8'
E(toluene)[M06-2X/Def2-TZVP, SMD (toluene)] = -1392.973744 a.u.
Gcorr[M06-2X/Def2-SVP] = 0.586077 a.u.
Hcorr[M06-2X/Def2-SVP] = 0.680246 a.u.
G = E(toluene) + Gcorr= -1392.387667 a.u.
H = E(toluene) + Hcorr= -1392.293498 a.u.

C	2.21448500	-3.45816400	0.73314100
C	1.03448300	-3.28881000	-0.00766200
C	3.35846400	-2.69665700	0.43494700
C	3.52362500	-1.86700500	-0.69319800
H	4.28202500	-3.04777200	0.90648500
C	2.52475100	-1.10581300	-1.32518100
C	0.67761700	-2.06854200	-0.58205000
H	0.47020700	-4.19218100	-0.27159300
C	0.83811200	-0.79567900	0.27465200
C	1.75534000	0.01260300	-0.62164200
H	2.75493900	-0.87983800	-2.37532300
C	2.73626500	0.96467900	0.07945400
H	1.43321200	-1.09006300	1.15095300
C	2.34514200	-4.64422000	1.65574400
H	3.39018400	-4.95621900	1.78296400
H	1.94031800	-4.40616500	2.65199200
H	1.76828700	-5.49914500	1.27305100
C	4.87637300	-1.88671300	-1.37482400
H	5.33604700	-0.88824500	-1.32353900
H	5.55760400	-2.62687200	-0.93581100
H	4.74910300	-2.12874800	-2.44177000
C	-0.34356200	-2.06088800	-1.69602300
H	-1.37642800	-2.06906300	-1.31248500
H	-0.24413600	-1.16410500	-2.32479400
H	-0.20207700	-2.93916300	-2.33925400
C	2.08169500	2.18244100	0.75918200
C	1.39148000	3.07838700	-0.26052900
O	1.89446500	3.28101100	-1.34758000
C	0.05372800	3.68852100	0.10885400
H	-0.58868500	2.80780400	0.28177300
C	-0.50766500	4.52046200	-1.03454600
H	-1.49965100	4.91546900	-0.77294000
H	0.15692700	5.36795800	-1.25627300
H	-0.59263600	3.92546200	-1.95383000
C	0.10320400	4.47082300	1.42584200
H	0.76903600	5.34335700	1.34271300
H	-0.90341900	4.83847200	1.67067200
H	0.44426000	3.85104700	2.26663700
C	-0.49465300	-0.27331200	0.79309100
C	-1.35628800	0.35453700	-0.02215700
H	-0.99502100	0.60620700	-1.02779400
C	-0.75390100	-0.64261200	2.22982500
H	0.02112300	-0.17263000	2.85843900
H	-1.74269300	-0.33752900	2.58065000
H	-0.63655100	-1.73131300	2.36119200
C	-2.82744400	0.68749300	0.16885900
O	-3.45691700	-0.02690900	1.22833900
C	-4.11384700	-1.20658400	0.78525300
C	-4.32575200	-1.00354700	-0.70634000
C	-3.55179700	0.18312800	-1.07695100
H	-5.09224400	-1.23231500	1.29420900
H	-3.13098800	0.34927600	-2.07403900
C	-3.09887900	2.16822300	0.41217000
H	-4.18593000	2.31667600	0.45072100
H	-2.65701900	2.47753600	1.37022000
H	-2.67667300	2.78484400	-0.39384300
C	-3.32602500	-2.46447600	1.11912500
H	-3.89299600	-3.36427100	0.83927200
H	-2.35641900	-2.47756600	0.59801600
H	-3.13387600	-2.50388800	2.19928400
C	-4.68072000	-2.13917800	-1.62034400
H	-4.83267600	-1.75663700	-2.63746300
H	-3.88561500	-2.89715700	-1.63836300
H	-5.61283000	-2.62153600	-1.29027400
O	-4.95332800	0.24597500	-0.95343700
O	3.76943400	1.35368700	-0.79071300

H	3.39139800	2.02385500	-1.38152800
H	3.21900500	0.37256500	0.87759400
H	1.32475500	1.82060700	1.47525500
C	3.15309200	2.99489700	1.50166900
H	2.73938900	3.89009400	1.98398700
H	3.62404000	2.37128200	2.27461100
H	3.93190500	3.29947500	0.79112600
H	1.18162700	0.56637300	-1.38592400

TS9
E(toluene)[M06-2X/Def2-TZVP, SMD (toluene)] = -1353.668219 a.u.
Gcorr[M06-2X/Def2-SVP] = 0.557632 a.u.
Hcorr[M06-2X/Def2-SVP] = 0.650241 a.u.
G = E(toluene) + Gcorr= -1353.110587 a.u.
H = E(toluene) + Hcorr= -1353.017978 a.u.

C	-0.92225400	2.48377400	1.58126600
C	-0.25194100	3.08623600	0.50673200
C	-2.24423000	2.00295300	1.46612600
C	-2.94183100	1.95381400	0.26023100
H	-2.81073900	1.94292200	2.40208000
C	-2.22055000	1.77010200	-0.92934100
C	-0.37459500	2.88529300	-0.88322300
H	0.35105100	3.95548100	0.80233200
C	-0.04457500	1.59434000	-1.63554300
C	-1.14201600	0.71228300	-1.06411900
H	-2.75319000	1.97685900	-1.86946600
C	-1.55213700	-0.49339200	-1.90985500
H	-0.29926300	1.77758500	-2.69332200
C	-0.34513500	2.65617800	2.96483700
H	-1.12872600	2.73438600	3.73105400
H	0.28441500	1.78924400	3.22392900
H	0.29510500	3.54837200	3.02039600
C	-4.42269100	2.24660400	0.22112500
H	-4.83452000	2.37325800	1.23128200
H	-4.61005000	3.18050100	-0.33237900
H	-4.98803300	1.44974500	-0.28745100
C	-2.72733400	-1.28495300	-1.29154200
C	-2.36305300	-1.73236400	0.11900100
O	-1.28671100	-2.25182800	0.33371700
C	-3.37335300	-1.51423000	1.23093800
H	-3.63720600	-0.44333700	1.17777900
C	-2.76595400	-1.82628000	2.58997600
H	-1.87736600	-1.20599600	2.77236000
H	-3.49575800	-1.64156300	3.39070600
H	-2.44953000	-2.87800400	2.63708200
C	-4.64644900	-2.32611400	0.95709000
H	-5.11740200	-2.04807100	0.00334400
H	-4.42459400	-3.40394200	0.93286100
H	-5.38013000	-2.15041900	1.75614900
C	1.39502700	1.09764100	-1.59191100
C	2.06591600	1.04756400	-0.43327800
H	1.56603200	1.43431800	0.46189400
C	1.94747900	0.65631200	-2.91982700
H	1.96340300	1.51207400	-3.61516500
H	2.95175400	0.23072200	-2.83634700
H	1.27850300	-0.10645700	-3.34572600
C	3.47887700	0.53760200	-0.21105900
O	3.76251800	-0.67811900	-0.90512400
C	3.49570200	-1.81527200	-0.10247800
C	3.62728000	-1.32127900	1.33036500
C	3.62504100	0.14160600	1.25227000
H	4.28252900	-2.55361400	-0.33037800
H	3.25226900	0.79495600	2.04805900

C	4.53047200	1.55482400	-0.63810500
H	4.46308600	1.72862400	-1.72059000
H	4.37261500	2.51223700	-0.12202400
H	5.52590300	1.16013800	-0.39374200
C	2.11768200	-2.39982200	-0.37915700
H	1.33279400	-1.72485800	-0.00535700
H	1.98491500	-2.52485400	-1.46287800
H	1.99207900	-3.37792800	0.10786400
C	3.15126500	-2.13982100	2.49440100
H	3.67350900	-3.10783100	2.52189600
H	3.36552800	-1.60739000	3.42984200
H	2.07134600	-2.32963700	2.42680600
O	4.81610200	-0.56810900	1.50816900
O	-0.45581000	-1.32737100	-2.17574700
H	-0.24344200	-1.78744800	-1.34879100
H	-1.89548100	-0.11649500	-2.89114300
H	-3.60289000	-0.61803400	-1.23430100
C	-3.06130300	-2.50680700	-2.15451400
H	-3.31707800	-2.18848700	-3.17520600
H	-2.18572200	-3.16525700	-2.21393500
H	-3.90704100	-3.07713800	-1.74656000
H	-0.12052000	3.78573700	-1.46067700
H	-0.82899700	0.37226100	-0.06281500

TS9’
E(toluene)[M06-2X/Def2-TZVP, SMD (toluene)] = -1353.667427 a.u.
Gcorr[M06-2X/Def2-SVP] = 0.557115 a.u.
Hcorr[M06-2X/Def2-SVP] = 0.6502 a.u.
G = E(toluene) + Gcorr= -1353.110312 a.u.
H = E(toluene) + Hcorr= -1353.017227 a.u.

C	3.93508900	2.50780900	0.02333600
C	3.19290400	2.50326600	-1.16312300
C	4.18093900	1.30129700	0.71029400
C	3.91306200	0.00980600	0.22493800
H	4.94634700	1.36784700	1.49044800
C	2.84109300	-0.35845200	-0.61916100
C	2.18497500	1.56657400	-1.38257500
H	3.50876700	3.17138600	-1.97121900
C	1.15711800	1.23865700	-0.30006000
C	1.36868300	-0.25279900	-0.22548200
H	3.07050800	-1.24524100	-1.23082400
H	1.50204900	1.69194200	0.64492800
C	1.02103300	-0.92437900	1.11643600
C	4.70618700	3.74089600	0.42122500
H	5.61344500	3.49700500	0.98992300
H	4.99244500	4.32450000	-0.46571200
H	4.08175700	4.39613700	1.04855400
C	4.96534900	-1.05110500	0.48720900
H	4.58396200	-1.84324400	1.15154100
H	5.24440000	-1.53673300	-0.46122200
H	5.87611300	-0.63457500	0.93607600
C	-0.66852700	-3.84374500	-2.27960800
H	-0.97077400	-2.85357700	-2.64779500
H	-0.31628500	-4.44241500	-3.13084500
H	-1.56247200	-4.32463500	-1.85818400
C	1.00233400	-2.46413300	0.98801800
C	-0.02826000	-2.85431700	-0.06332000
C	0.41751200	-3.72823900	-1.22084800
H	1.30748900	-3.22547700	-1.64422200
C	0.86194300	-5.09888700	-0.69123800
H	1.66850800	-5.01566800	0.05120600
H	0.01435100	-5.62181900	-0.22303300
H	1.22809800	-5.72139200	-1.51919100

O	-1.17275300	-2.45563600	0.02373800
C	-0.23122900	1.77970200	-0.58213200
C	-0.35193500	3.26834800	-0.40384500
H	-0.17968200	3.53057300	0.65329100
H	0.43865000	3.77196600	-0.98314200
H	-1.33337100	3.65090400	-0.69902100
C	-1.23345600	0.96036300	-0.92341300
H	-1.00992700	-0.10256800	-1.05693000
C	-2.71008100	1.26210700	-1.08910300
O	-3.17148500	2.34040800	-0.28110300
C	-3.62643200	1.89501200	0.99015800
C	-3.98195600	0.43046000	0.78522300
C	-3.44889600	0.05333500	-0.52644300
H	-4.54003400	2.47141400	1.21384000
H	-3.12883200	-0.96418700	-0.77585900
C	-3.09059200	1.57894700	-2.52911700
H	-2.75505900	0.77929700	-3.20449900
H	-4.18196700	1.68455900	-2.59413500
H	-2.61448000	2.51991800	-2.83760800
C	-2.58916200	2.11268700	2.08257100
H	-2.28916100	3.16937100	2.09093100
H	-3.01034400	1.87119600	3.06988600
H	-1.69439700	1.49255800	1.91824900
C	-4.18584300	-0.50775000	1.93796600
H	-4.43488800	-1.50677200	1.55830800
H	-3.27511700	-0.57536300	2.54961400
H	-5.01054900	-0.16220500	2.57939000
O	-4.82810700	0.27502800	-0.34493800
O	-0.16697100	-0.43806300	1.68262100
H	-0.90692000	-0.76606700	1.14929400
H	1.82044700	-0.65644100	1.82905300
C	0.67431100	-3.13211400	2.32433300
H	1.41399800	-2.84128100	3.08355900
H	-0.31454600	-2.80178500	2.66641900
H	0.67345200	-4.22822100	2.24070500
H	1.99925800	-2.78345600	0.64182000
H	1.80251500	1.45131900	-2.40428100
H	0.79979400	-0.72273900	-1.04530100

TS10
E(toluene)[M06-2X/Def2-TZVP, SMD (toluene)] = -1353.665787 a.u.
Gcorr[M06-2X/Def2-SVP] = 0.558042 a.u.
Hcorr[M06-2X/Def2-SVP] = 0.65063 a.u.
G = E(toluene) + Gcorr= -1353.107745 a.u.
H = E(toluene) + Hcorr= -1353.015157 a.u.

C	0.72279400	2.83480900	0.40387800
C	-0.23862100	2.34615000	-0.49566600
C	2.10266100	2.62303100	0.20246100
C	2.61123800	1.79313400	-0.79688500
H	2.76893800	3.36503200	0.65675900
C	1.85897300	0.67957500	-1.19238100
C	-0.22030000	1.21452600	-1.33525500
H	-1.04455000	3.06705300	-0.69245500
C	-0.18875700	-0.23826000	-0.87521000
C	1.18186500	-0.25635800	-0.20778700
H	2.15240900	0.18630300	-2.12750600
H	-0.07328300	-0.85846800	-1.78109400
C	1.81926500	-1.64504400	-0.05695100
C	0.29973800	3.89953800	1.38616800
H	-0.58269800	4.44607100	1.02284100
H	0.02203700	3.44426400	2.34976100
H	1.10540800	4.62095900	1.58070900
C	3.83115700	2.20288700	-1.58697200

H	3.52561000	2.54695100	-2.58844000
H	4.35800000	3.03867000	-1.10478100
H	4.52790500	1.36281800	-1.72529300
C	4.98292600	0.87486200	1.54754200
H	5.73211400	1.19226000	2.28657300
H	4.90166000	1.65938600	0.78306400
H	4.01066300	0.80000300	2.05514500
C	3.13667700	-1.67237100	0.73947900
C	4.31183700	-1.01824000	0.03073600
C	5.41852100	-0.45075000	0.90802000
H	5.55790500	-1.18197400	1.72340300
C	6.71620600	-0.29587400	0.12666200
H	7.02840100	-1.24677600	-0.32563600
H	6.58458900	0.43056900	-0.68759900
H	7.51980900	0.06240100	0.78504600
O	4.40434500	-1.02663000	-1.17984100
C	-1.39767900	-0.78384400	-0.14093200
C	-1.41169800	-2.28767700	0.02431900
H	-0.80813700	-2.77211200	-0.75738200
H	-0.97170900	-2.57404400	0.99370600
H	-2.43551400	-2.67699900	0.00125900
C	-2.38897500	0.00790700	0.29524400
H	-2.28255800	1.08619700	0.15129400
C	-3.66502700	-0.42892600	0.99806600
O	-4.31749800	-1.53095000	0.35994600
C	-5.27851900	-1.09825100	-0.58777700
C	-5.68374500	0.29228900	-0.12313900
C	-4.68674400	0.69262500	0.87332000
H	-6.13346800	-1.79059000	-0.50789700
H	-4.40221900	1.73191000	1.06749800
C	-3.42613000	-0.83211900	2.44742500
H	-4.39381900	-1.04762300	2.92028000
H	-2.79919900	-1.73313900	2.48422200
H	-2.91532800	-0.02631000	2.99288800
C	-4.72454800	-1.10413900	-2.00554100
H	-4.30472400	-2.09409600	-2.22966400
H	-5.51454200	-0.88822000	-2.73969000
H	-3.92234200	-0.35868900	-2.11350600
C	-6.47555900	1.21996300	-0.99702700
H	-6.69391500	2.14420600	-0.44725200
H	-5.92267900	1.46941400	-1.91279600
H	-7.43095600	0.75565100	-1.28384300
O	-5.98224300	0.29756100	1.26349000
C	3.53912800	-3.12997700	1.02371000
H	2.76902900	-3.61822600	1.63686900
H	3.61775300	-3.67756000	0.07532200
H	4.49686200	-3.20042800	1.55807100
H	2.97565100	-1.15554800	1.69883600
O	1.93982100	-2.30534700	-1.29245000
H	2.73795800	-1.95477700	-1.71759700
H	1.11226700	-2.24439900	0.53739900
H	-0.82658400	1.35400500	-2.24288100
H	1.11775600	0.22199500	0.78215900

TS10’

E(toluene)[M06-2X/Def2-TZVP, SMD (toluene)] = -1353.665554 a.u.
Gcorr[M06-2X/Def2-SVP] = 0.556576 a.u.
Hcorr[M06-2X/Def2-SVP] = 0.6498 a.u.
G = E(toluene) + Gcorr= -1353.108978 a.u.
H = E(toluene) + Hcorr= -1353.015754 a.u.

C	2.14615600	-3.59413500	0.57076600
C	0.94738400	-3.34768000	-0.11725500
C	3.29838000	-2.84631800	0.27042700

C	3.45475800	-1.96439200	-0.81873700
H	4.22702600	-3.24455600	0.69240100
C	2.46422600	-1.14309900	-1.38730500
C	0.63409800	-2.07885800	-0.58723200
H	0.33218600	-4.20779200	-0.40346300
C	0.83644200	-0.84295900	0.28722400
C	1.75125600	-0.03507600	-0.61718000
H	2.67837300	-0.87470100	-2.43048700
C	2.76727100	0.88268300	0.07823300
H	1.42350600	-1.15406500	1.16533200
C	2.27467000	-4.83108200	1.42355800
H	3.31548300	-5.17136100	1.50444400
H	1.90106500	-4.64152200	2.44209900
H	1.66942100	-5.65128000	1.00979300
C	4.78784700	-1.98721400	-1.53821300
H	5.27583900	-1.00467400	-1.45247800
H	5.46009000	-2.76487000	-1.15350300
H	4.62555400	-2.17473100	-2.61132700
C	2.12822600	2.10766700	0.75883500
C	1.47494600	3.02014000	-0.27153800
O	2.04970100	3.29034800	-1.30692200
C	0.08954100	3.55586600	0.02185500
H	-0.52142000	2.64040400	0.11664100
C	-0.42924300	4.41097400	-1.12417300
H	-1.45932700	4.73998500	-0.92514400
H	0.20041700	5.30324000	-1.25284000
H	-0.40876600	3.85805500	-2.07256000
C	0.02233700	4.28461700	1.36818600
H	0.64215600	5.19431500	1.35491400
H	-1.01491700	4.58714100	1.57156800
H	0.35387100	3.65088700	2.20297300
C	-0.48994100	-0.28195900	0.77988500
C	-1.39734400	0.18413500	-0.09150600
H	-1.09809500	0.24995700	-1.14656200
C	-0.71834700	-0.43339500	2.25861500
H	0.09090200	0.07390300	2.81004200
H	-1.68932500	-0.05359900	2.58825300
H	-0.65076200	-1.50168700	2.52680100
C	-2.84578600	0.56273300	0.18318400
O	-3.45496700	-0.26192200	1.17657500
C	-4.11597400	-1.38278400	0.60940500
C	-4.42876200	-0.96754200	-0.81999700
C	-3.65278600	0.24938600	-1.07096500
H	-5.05365200	-1.51411700	1.17521100
H	-3.29035200	0.55104300	-2.05914100
C	-3.03543300	2.00641500	0.63428700
H	-4.10735000	2.17918300	0.79717400
H	-2.49124800	2.18466400	1.57331700
H	-2.66662400	2.70734400	-0.12802600
C	-3.27044600	-2.64532000	0.69255200
H	-3.83775600	-3.52300300	0.34966700
H	-2.35648300	-2.54911900	0.08666600
H	-2.97219500	-2.81523200	1.73592400
C	-4.86693900	-1.95972900	-1.85613300
H	-5.06848400	-1.43959500	-2.80104000
H	-4.09480000	-2.72268000	-2.02424200
H	-5.79072400	-2.46488100	-1.53686200
O	-5.04232900	0.31203500	-0.84971600
O	3.80454300	1.25440900	-0.79275300
H	3.46064600	1.97464700	-1.34502000
H	3.23875700	0.27571400	0.87251100
H	1.35224600	1.74903600	1.45492500
C	3.19023800	2.90235900	1.52943100
H	2.76875800	3.79521000	2.01151500
H	3.64279700	2.26953800	2.30582600
H	3.98358000	3.21297500	0.83734800

H	-0.17918700	-1.98188500	-1.31597600
H	1.15255100	0.54654600	-1.34140600

5 Scheme S1: Stereochemical implications for the synthesis of triol 19

In the Upjohn dihydroxylation, no reagent control is operative (Scheme S2). Therefore, both the major and the minor enantiomer undergo the reaction with the same diastereoselectivity. As a result, after separation of the diastereomers, the product triol has the same ee as the starting material. In the SAD reaction, the chiral reagent has a preference for the "back" face of the alkene in both enantiomers. Therefore, the diastereoselectivity for the two enantiomers is different and an increase in ee for the desired product is obtained. While we never measured the ee of the undesired diastereomer, it should have theoretically decreased.

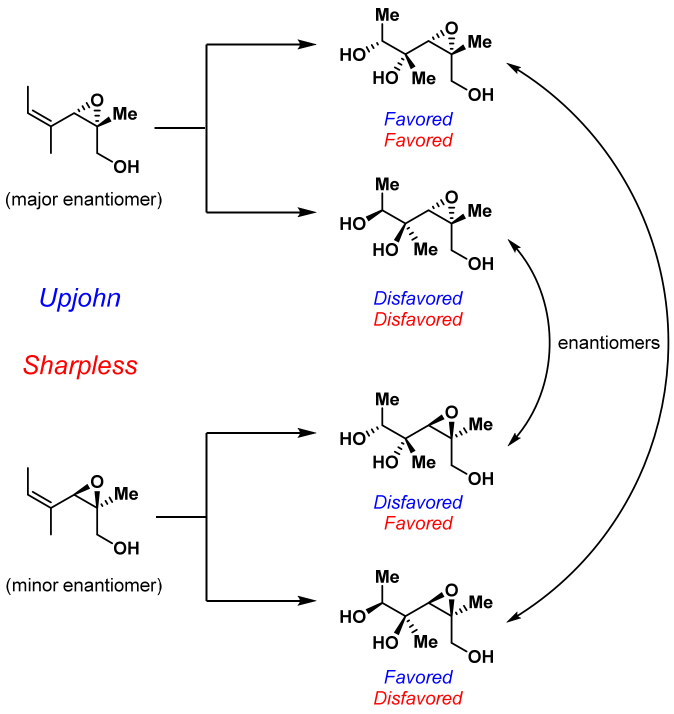

Scheme S1: Reagent-control induced enantiomeric enrichment in the Sharpless asymmetric dihydroxylation

6 Scheme S2: Synthetic approaches toward the dioxabicyclo[3.1.0]hexane fragment

Our originally planned approach is shown at the top of Scheme S1. Selective oxidation of the primary alcohol in **34** using the Stahl protocol was successful to give aldehyde **S9**. Selective mesylation of

the remaining secondary hydroxyl, in the presence of the tertiary hydroxyl, was surprisingly difficult, despite significant literature precedence on similar substrates.[24] The reaction was quite sluggish and over-mesylation was a significant problem. Under optimized conditions using slightly over one equivalent of MsCl in pyridine, 27% of the desired product **S10** could be isolated after careful chromatography. This material cleanly formed epoxide **17** upon treatment with NaOEt in EtOH.

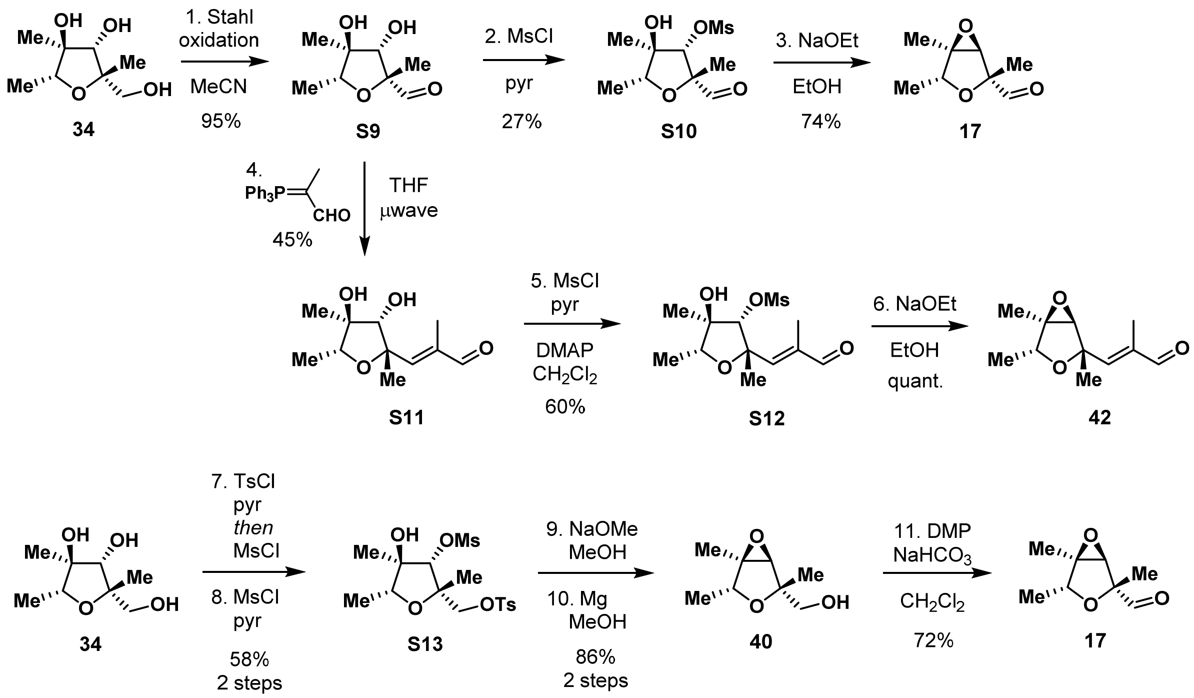

Scheme S2: Three generations of approaches to convert THF triol **34** into epoxy aldehyde **17**. Reagents and conditions: 1. [Cu(MeCN)$_4$]OTf (0.08 equiv), ^{MeO}bpy (0.08 equiv), ABNO (0.02 equiv), NMI (0.15 equiv), O_2, 4 Å MS, MeCN, rt, 0.5 h, 95%; 2. MsCl (1.30 equiv), pyridine, 0 °C → rt, 18 h, 27%; 3. NaOEt (2.00 equiv), EtOH, 0 °C → rt, 2 h, 74%; 4. 2-(triphenylphosphhoranylidene)propionaldehyde (1.10 equiv), THF, 125 °C, 2.5 d, 45 %, 5. MsCl (6.00 equiv), DMAP (0.10 equiv), pyridine, 0 °C → rt, 9 h, 60%, 6. NaOEt (3.00 equiv), EtOH, 0 °C, quant.; 7. TsCl (1.20 equiv), pyridine, 0 °C → rt, 4 h *then* MsCl (1.10 equiv), 0 °C → rt, 18 h; 8. MsCl (1.10 equiv), pyridine, 0°C → rt, 18 h, 58% (2 steps); 9. NaOMe (4.00 equiv), MeOH, 0 °C → rt, 20 h; 10. Mg (10.0 equiv), MeOH, rt, 2 h, 86% (2 steps); 11. DMP (2.00 equiv), $NaHCO_3$ (10.0 equiv), CH_2Cl_2, 0 °C → rt, 1.5 h, 72%.

Unsatisfied with this approach, we chose to take advantage of the fact that the primary hydroxyl group in **34** could be cleanly converted to the corresponding mono-tosylate. After completion of the reaction, MsCl was added, resulting in a mixture of mono, bis, and unmesylated products. After separation, subjection of the unmesylated material to mesylation conditions gave **S13** in a combined two step yield of 58%. While this approach was tedious, it could deliver reasonable quantities of

S13, which could then cleanly be converted to **17** via basic epoxide formation, cleavage of the tosylate, and Dess–Martin oxidation.

In parallel, we hypothesized that the secondary hydroxyl in **S9** was hydrogen-bonding to the carboxyl group, thereby resulting in the low selectivity of the mesylation reaction. We, therefore, first converted the aldehyde to **S11** in a Wittig homologation. This compound then cleanly underwent selective mesylation, even with a large excess of mesyl chloride. Basic ring closure then gave **42**.

While all three approaches were probably "good enough" to complete a total synthesis, we ultimately selected the four-step approach presented in the main text because it was scalable, presented no challenging isolation/purification steps, and enabled us to boost the enantiomeric excess via crystallization. We considered that a high ee was important to avoid diastereomeric products forming in the Stille coupling of the two enantioenriched fragments.

7 References

1 R. Rodriguez, R.M. Adlington, S.J. Eade, M.W. Walter, J.E. Baldwin, J.E. Moses *Tetrahedron* **2007**, *63*, 4500–4509.

2 J.E. Barbarow, A.K. Miller, D. Trauner *Org. Lett.* **2005**, *7*, 2901–2903.

3 I. Paterson, D.J. Wallace, C.J. Cowden *Synthesis* **1998**, *S1*, 639–652.

4 G.A. Molander, D.J. St. Jean *J. Org. Chem.* **2002**, *67*, 3861–3865.

5 P. Wipf, P.C. Fritch *J. Org. Chem.* **1994**, *59*, 4875–4886.

6 M. Fieser *Reagents for Organic Synthesis, Vol. 3*, John Wiley & Sons, **1971**.

7 U. Vogeli, W. von Philipsborn *Org. Mag. Res.* **1975**, *7*, 617–627.

8 L.L. Klein *Tetrahedron Lett.* **1986**, *27*, 4545–4548.

9 Q. Li, C. Chen, Y. He, D. Guan, L. Cheng, X. Hao, M. Wei, Y. Zheng, C. Liu, X.-N. Li *Org. Lett.* **2019**, *21*, 5091–5095.

10. L. Krause, R. Herbst-Irmer, G.M. Sheldrick, D. Stalke *J. Appl. Cryst.* **2015**, *48*, 3–10.

11. G.M. Sheldrick *Acta. Cryst.* **2015**, *A71*, 3–8.

12. G.M. Sheldrick *Acta. Cryst.* **2015**, *C71*, 3–8.

13. a) P. Pracht, F. Bohle, S. Grimme *PCCP* **2020**, *22*, 7169–7192. b) S. Grimme *J. Chem. Theory Comput.* **2019**, *15*, 2847–2862.

14. a) C. Bannwarth, E. Caldeweyher, S. Ehlert, A. Hansen, P. Pracht, J. Seibert, S. Spicher, S. Grimme *WIREs Comput. Mol. Sci.* **2020**, *11*, e01493. b) S. Grimme, C. Bannwarth, P. Shushkov *J. Chem. Theory Comput.* **2017**, *13*, 1989–2009. c) C. Bannwarth, S. Ehlert, S. Grimme. *J. Chem. Theory Comput.* **2019**, *15*, 1652–1671.

15. Y. Zhao, D. G. Truhlar *Theor. Chem. Acc.* **2008**, *120*, 215–241.

16. F. Weigend, R. Ahlrichs *Phys. Chem. Chem. Phys.* **2005**, *7*, 3297–3305.

17. Gaussian 09, Revision D.01, M. J. Frisch, G. W. Trucks, H. B. Schlegel, G. E. Scuseria, M. A. Robb, J. R. Cheeseman, G. Scalmani, V. Barone, G. A. Petersson, H. Nakatsuji, X. Li, M. Caricato, A. Marenich, J. Bloino, B. G. Janesko, R. Gomperts, B. Mennucci, H. P. Hratchian, J. V. Ortiz, A. F. Izmaylov, J. L. Sonnenberg, D. Williams-Young, F. Ding, F. Lipparini, F. Egidi, J. Goings, B. Peng, A. Petrone, T. Henderson, D. Ranasinghe, V. G. Zakrzewski, J. Gao, N. Rega, G. Zheng, W. Liang, M. Hada, M. Ehara, K. Toyota, R. Fukuda, J. Hasegawa, M. Ishida, T. Nakajima, Y. Honda, O. Kitao, H. Nakai, T. Vreven, K. Throssell, J. A. Montgomery, Jr., J. E. Peralta, F. Ogliaro, M. Bearpark, J. J. Heyd, E. Brothers, K. N. Kudin, V. N. Staroverov, T. Keith, R. Kobayashi, J. Normand, K. Raghavachari, A. Rendell, J. C. Burant, S. S. Iyengar, J. Tomasi, M. Cossi, J. M. Millam, M. Klene, C. Adamo, R. Cammi, J. W. Ochterski, R. L. Martin, K. Morokuma, O. Farkas, J. B. Foresman, and D. J. Fox, Gaussian, Inc., Wallingford CT, 2016.

18. N. Mardirossian, M. Head-Gordon *J. Chem. Theory Comput.* **2016**, *12*, 4303–4325.

19. A. V. Marenich, C. J. Cramer, D. G. Truhlar *J. Phys. Chem. B.* **2009**, *113*, 6378–6396.

20. G. Luchini, J. Alegre-Requena, IFunes, J. Rodríguez-Guerra, J. Chen, R. Paton, bobbypaton/GoodVibes: GoodVibes v3.0.0 (2019), doi:10.5281/ZENODO.3346166.

21. S. Grimme *Chem. Eur. J.* **2012**, *18*, 9955–9964.

22. Y. P. Li, J. Gomes, S. M. Sharada, A. T. Bell, M. Head-Gordon *J. Phys. Chem. C.* **2015**, *119*, 1840–1850.

23. C. Y. Legault, CYLview20 (2020), (available at http://www.cylview.org).

24. a) S. Hatakeyama, K. Sakurai, H. Numata, N. Ochi, S. Takano, *J. Am. Chem. Soc.* **1988**, *110*, 5201–5203; b) K. Whang, R. J. Cooke, G. Okay, J. K. Cha, *J. Am. Chem. Soc.* **1990**, *112*, 8985–8987; c) S. Nishiyama, Y. Shizuri, H. Shigemori, S. Yamamura, *Tetrahedron Lett.* **1986**, *27*, 723–726.

8 NMR Spectra

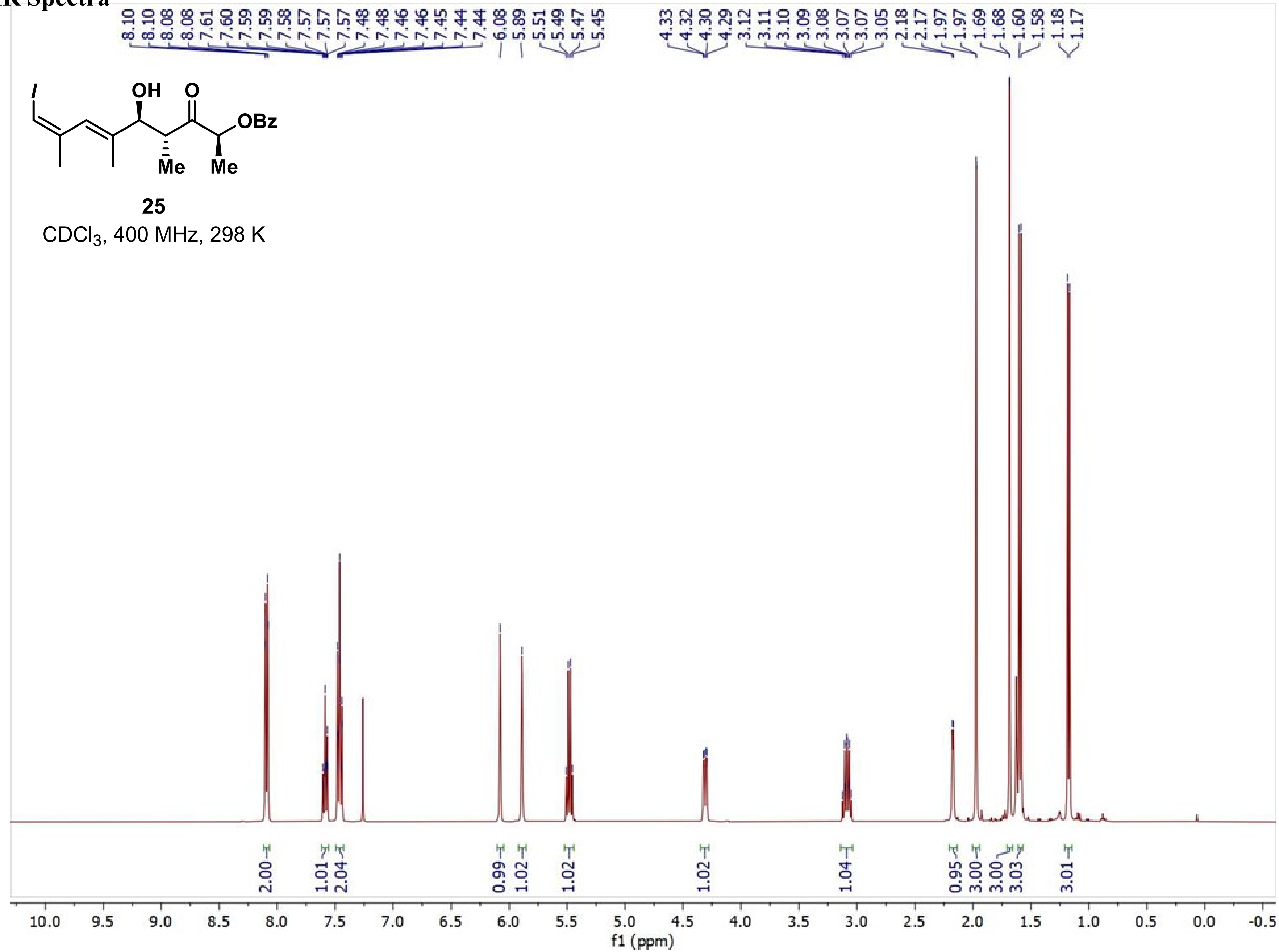

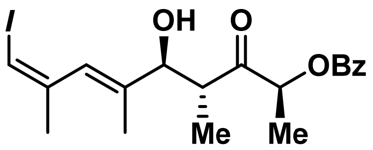

25

$CDCl_3$, 101 MHz, 298 K

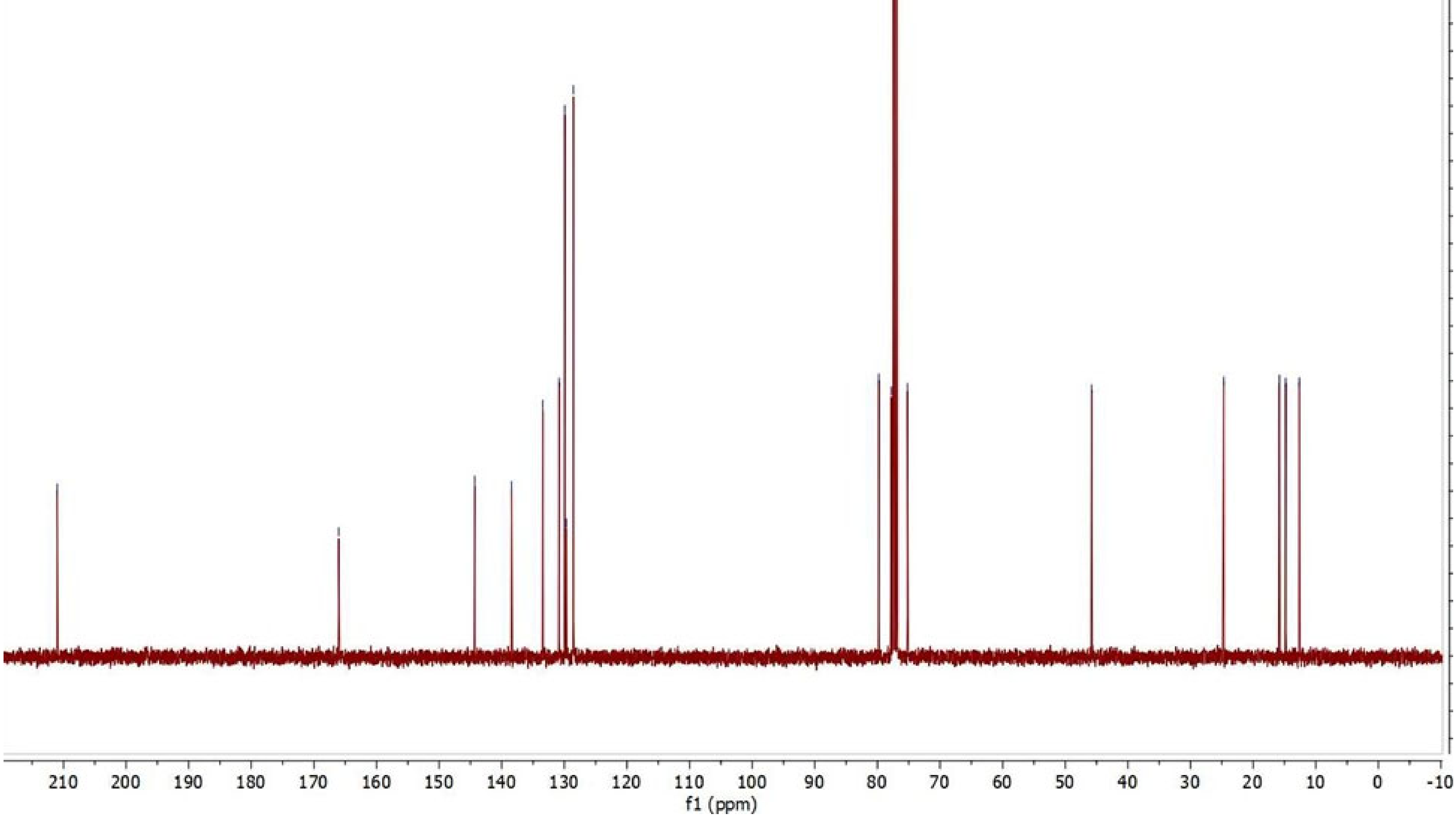

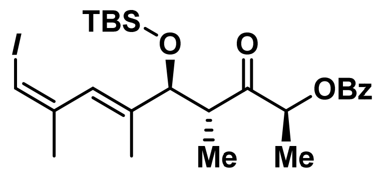

26

$CDCl_3$, 400 MHz, 298 K

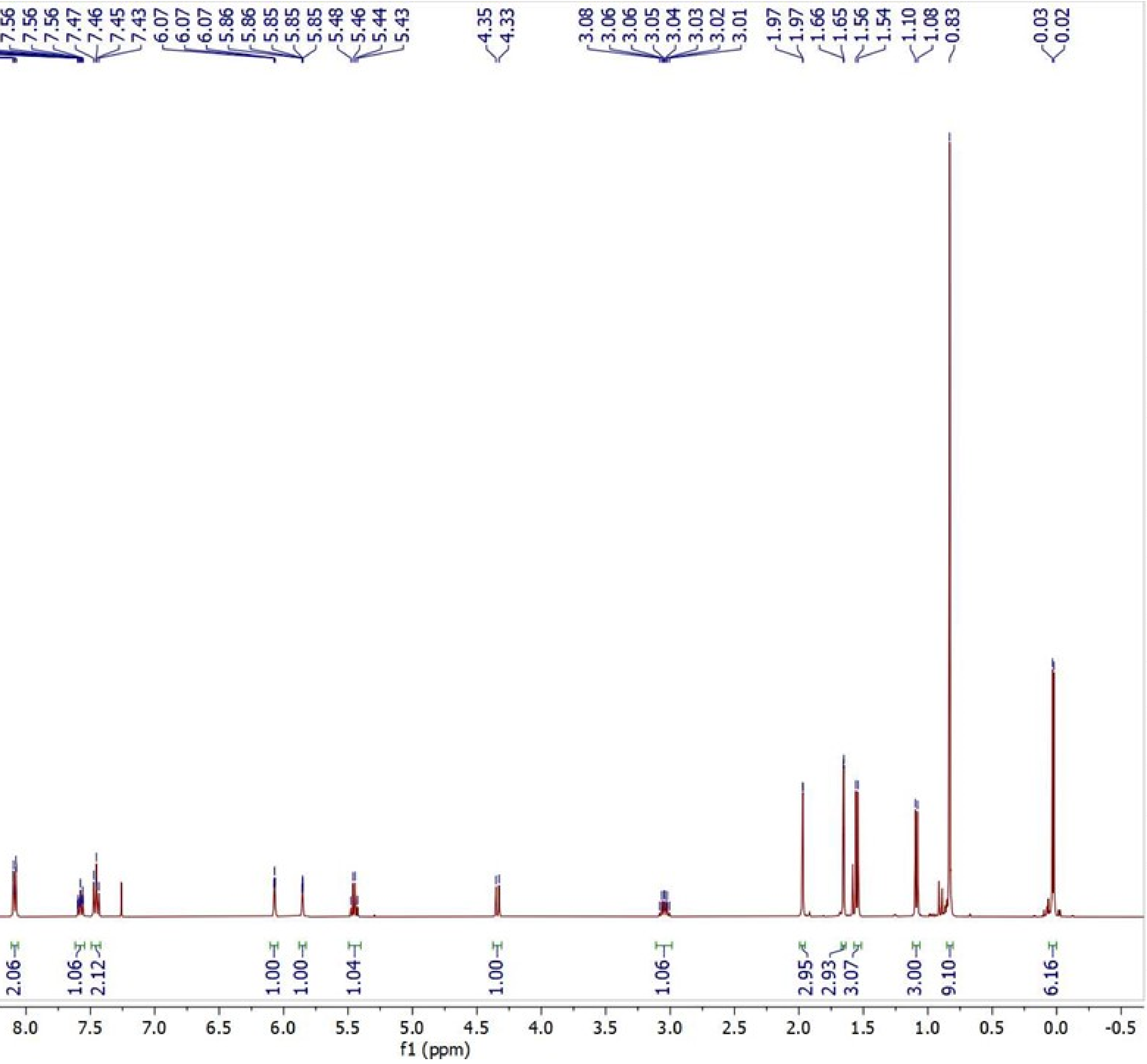

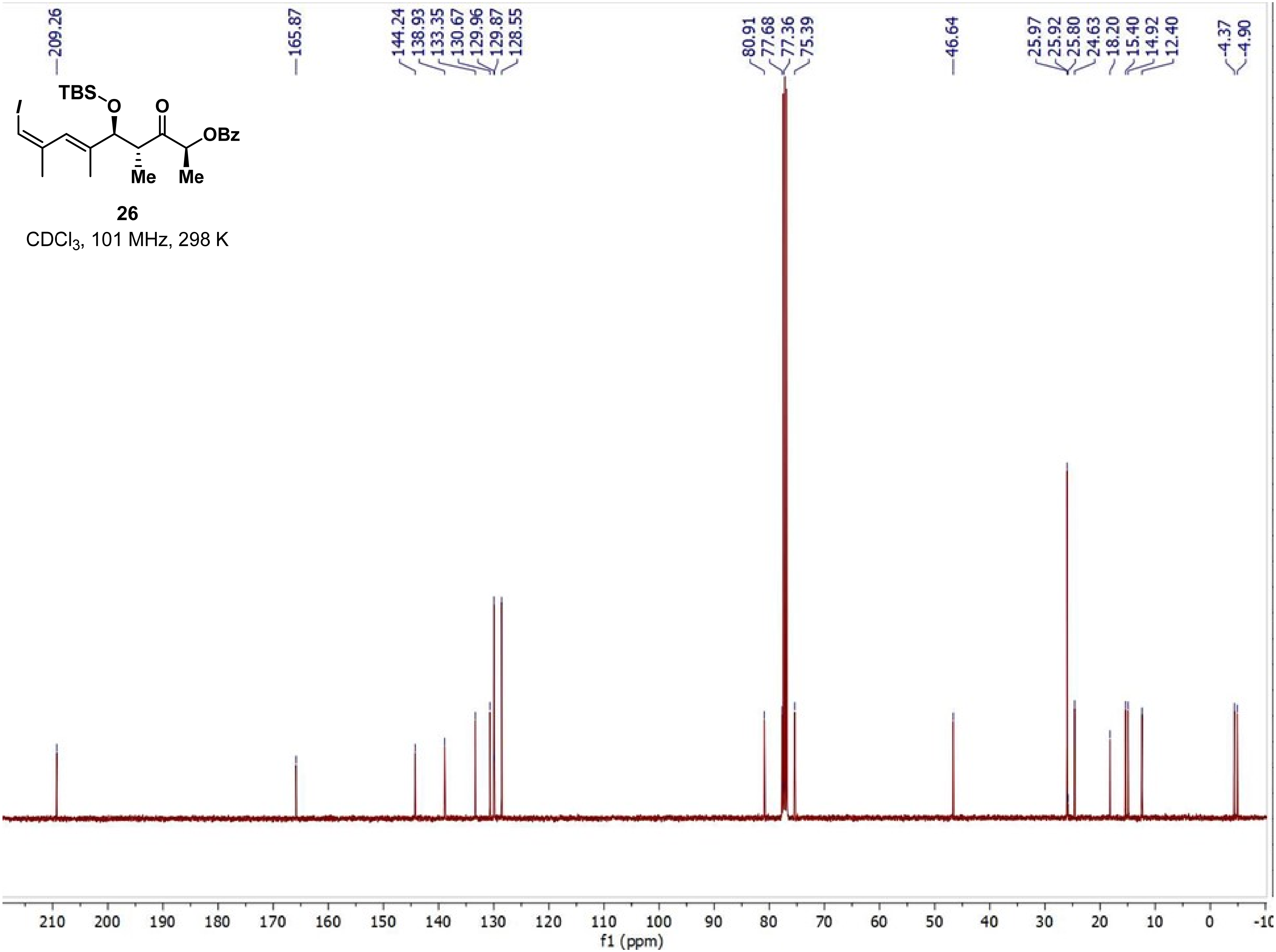
209.26
165.87
144.24
138.93
133.35
130.67
129.96
129.87
128.55
80.91
77.68
77.36
75.39
46.64
25.97
25.92
25.80
24.63
18.20
15.40
14.92
12.40
-4.37
-4.90
TBS
O
O
I
OBz
Me
Me
26
CDCl3, 101 MHz, 298 K
210 200 190 180 170 160 150 140 130 120 110 100 90 80 70 60 50 40 30 20 10 0 -10
f1 (ppm)

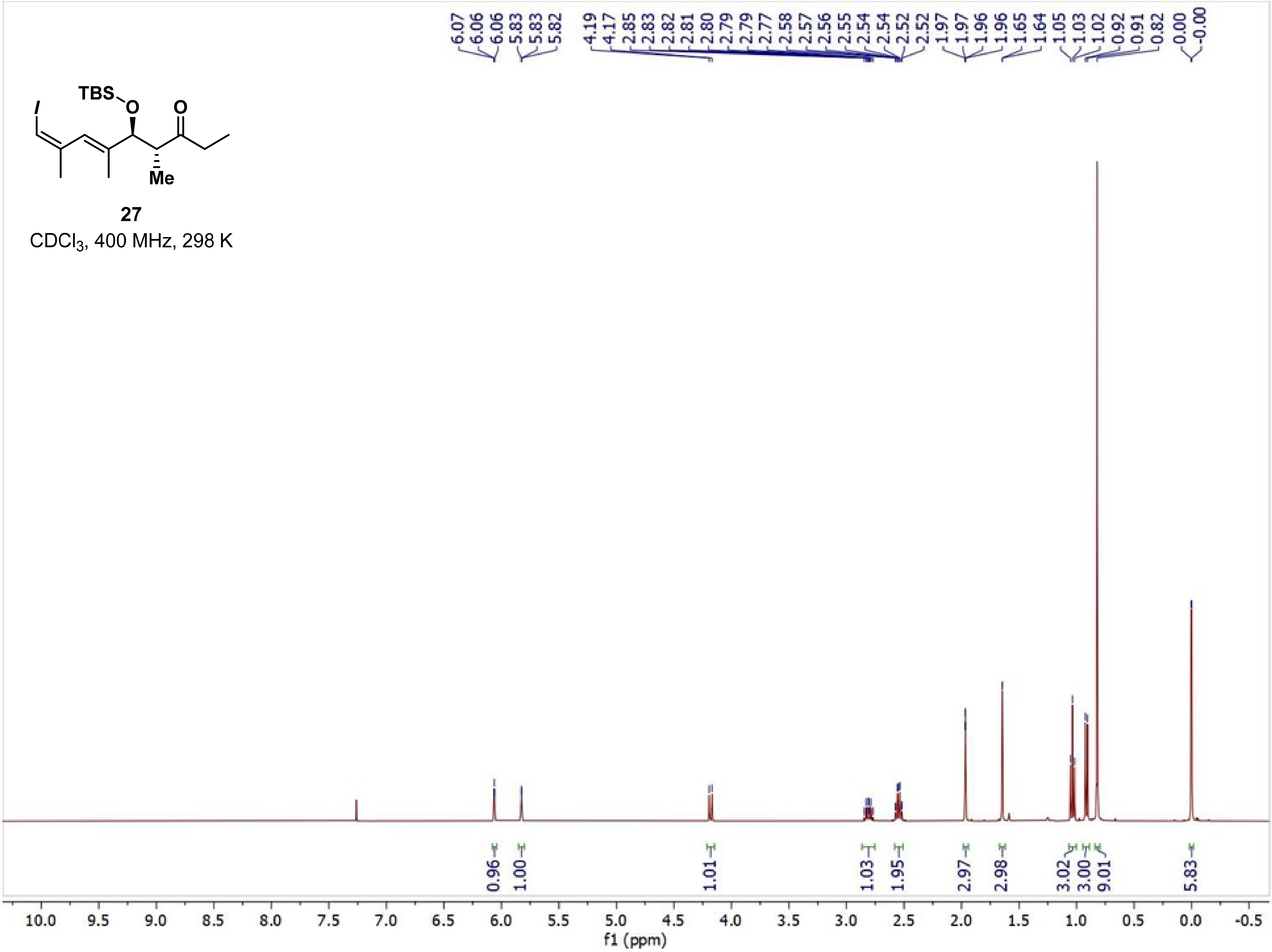

6.07
6.06
6.06
5.83
5.83
5.82
4.19
4.17
2.85
2.83
2.82
2.81
2.80
2.79
2.79
2.77
2.58
2.57
2.56
2.55
2.54
2.54
2.52
2.52
1.97
1.97
1.96
1.96
1.65
1.64
1.05
1.03
1.02
0.92
0.91
0.82
0.00
-0.00
TBS
O
O
I
Me
27
CDCl3, 400 MHz, 298 K
0.96
1.00
1.01
1.03
1.95
2.97
2.98
3.02
3.00
9.01
5.83
10.0 9.5 9.0 8.5 8.0 7.5 7.0 6.5 6.0 5.5 5.0 4.5 4.0 3.5 3.0 2.5 2.0 1.5 1.0 0.5 0.0 -0.5
f1 (ppm)

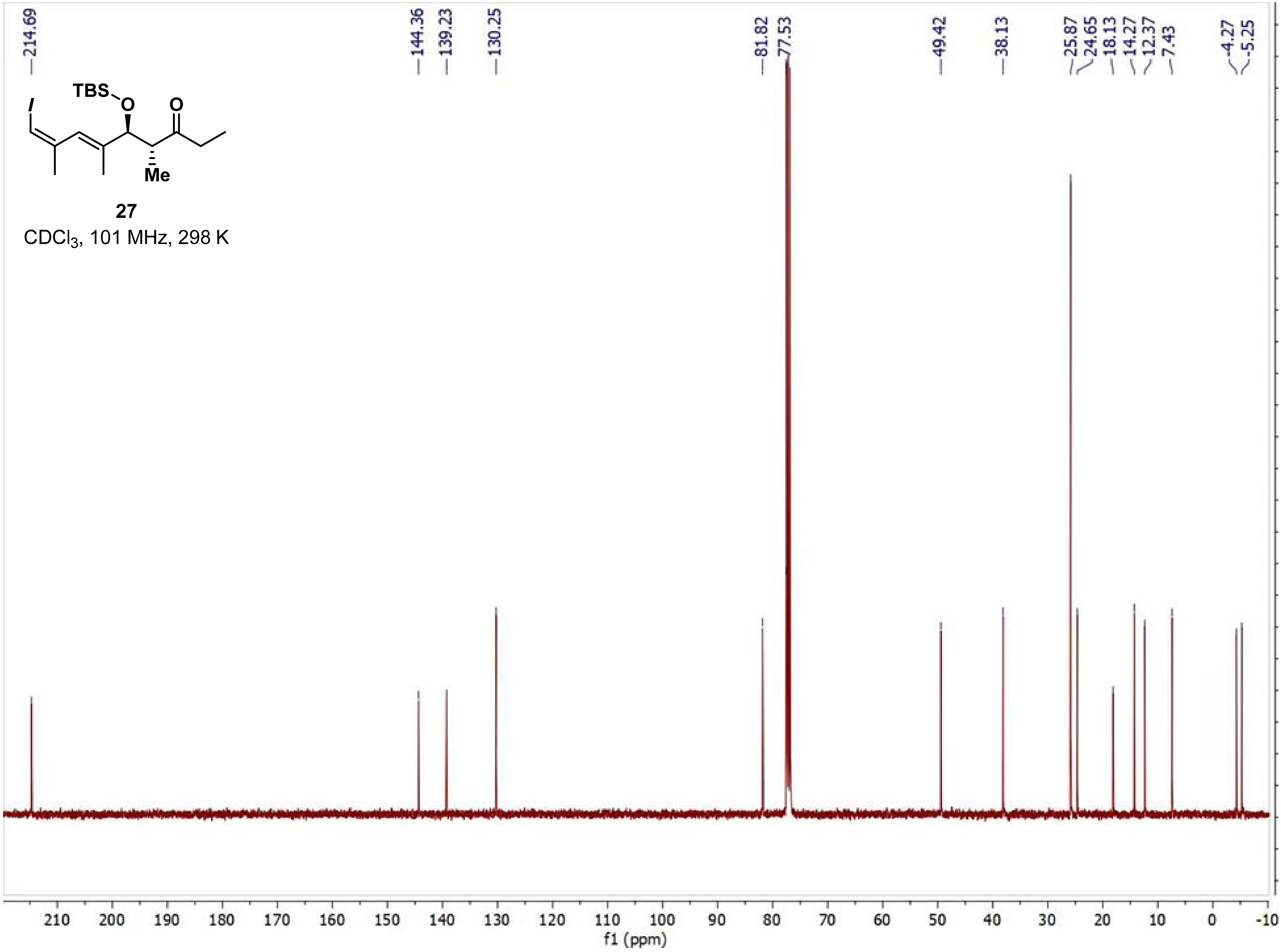
214.69
144.36
139.23
130.25
81.82
77.53
49.42
38.13
25.87
24.65
18.13
14.27
12.37
7.43
-4.27
-5.25
TBS
O
O
I
Me
27
CDCl3, 101 MHz, 298 K
210 200 190 180 170 160 150 140 130 120 110 100 90 80 70 60 50 40 30 20 10 0 -10
f1 (ppm)

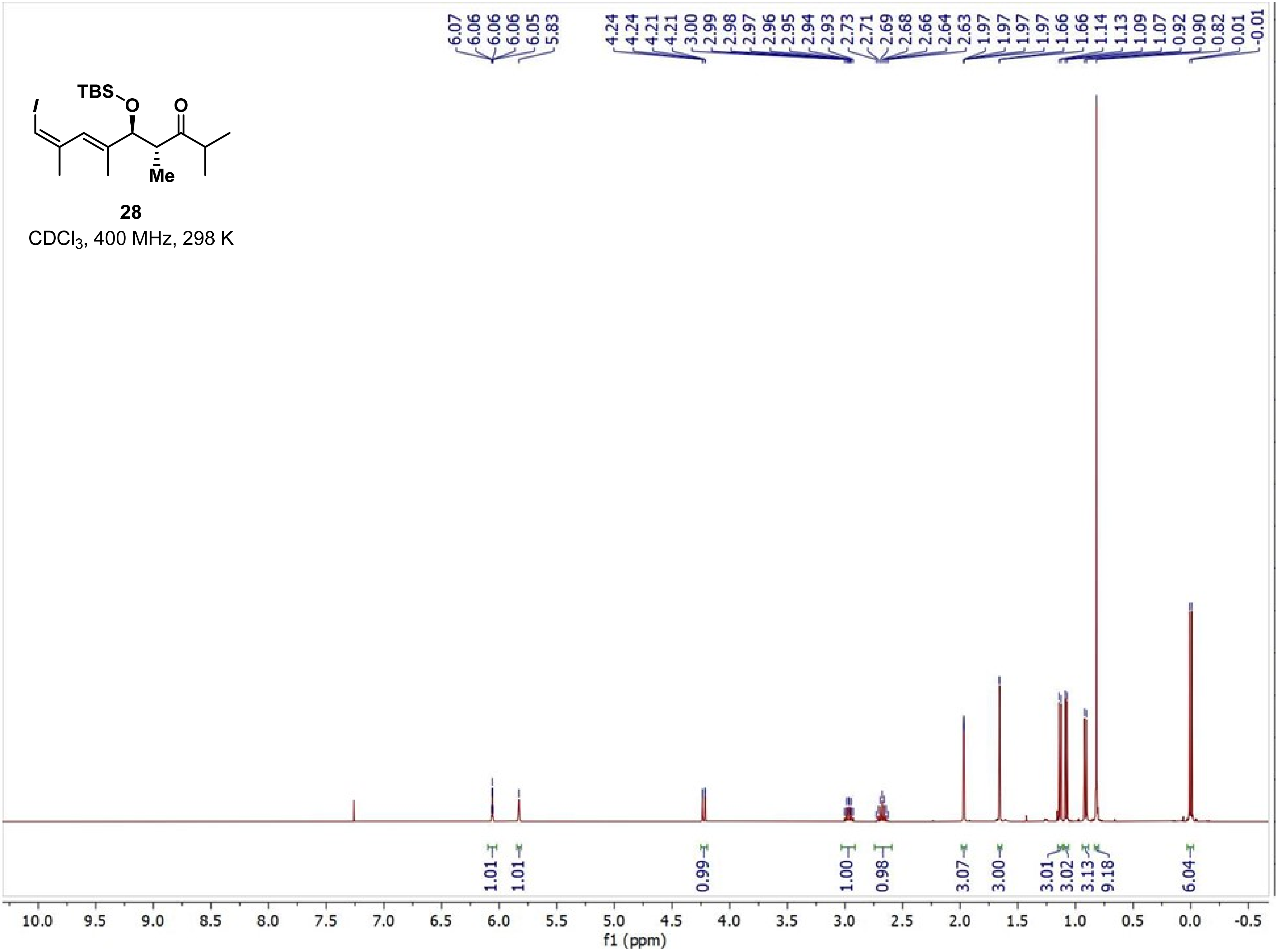
TBS
O
O
I
Me
28
$CDCl_3$, 400 MHz, 298 K
6.07 6.06 6.06 6.06 6.05 5.83
4.24 4.24 4.21 4.21 3.00 2.99 2.98 2.97 2.96 2.95 2.94 2.93 2.73 2.71 2.69 2.68 2.66 2.64 2.63 1.97 1.97 1.97 1.97 1.66 1.66 1.14 1.13 1.09 1.07 0.92 0.90 0.82 0.01 -0.01
1.01 1.01 0.99 1.00 0.98 3.07 3.00 3.01 3.02 3.13 9.18 6.04
10.0 9.5 9.0 8.5 8.0 7.5 7.0 6.5 6.0 5.5 5.0 4.5 4.0 3.5 3.0 2.5 2.0 1.5 1.0 0.5 0.0 -0.5
f1 (ppm)

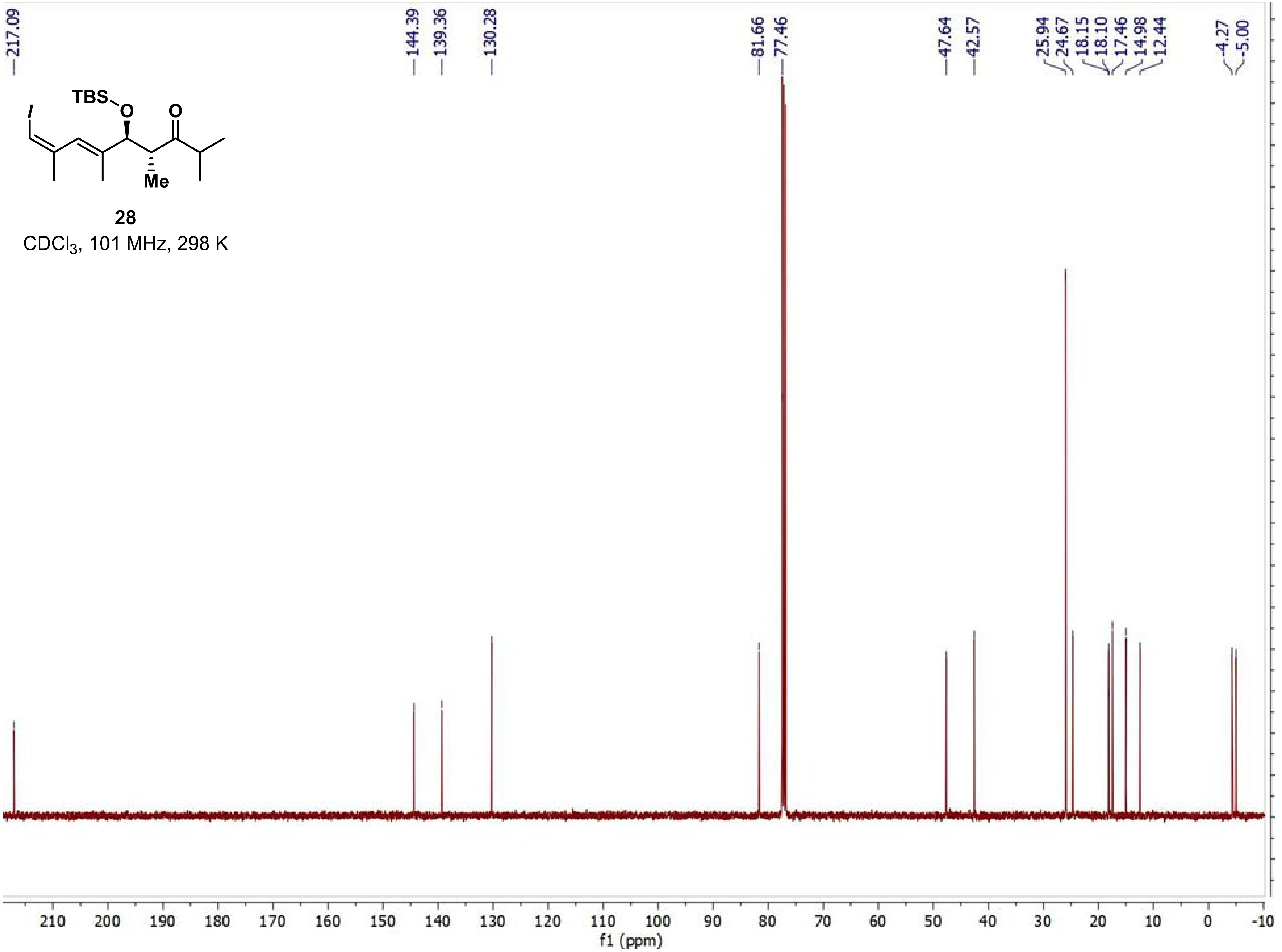

28

$CDCl_3$, 101 MHz, 298 K

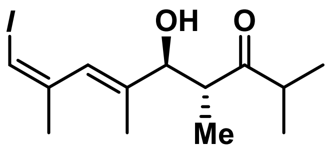

29

$CDCl_3$, 400 MHz, 298 K

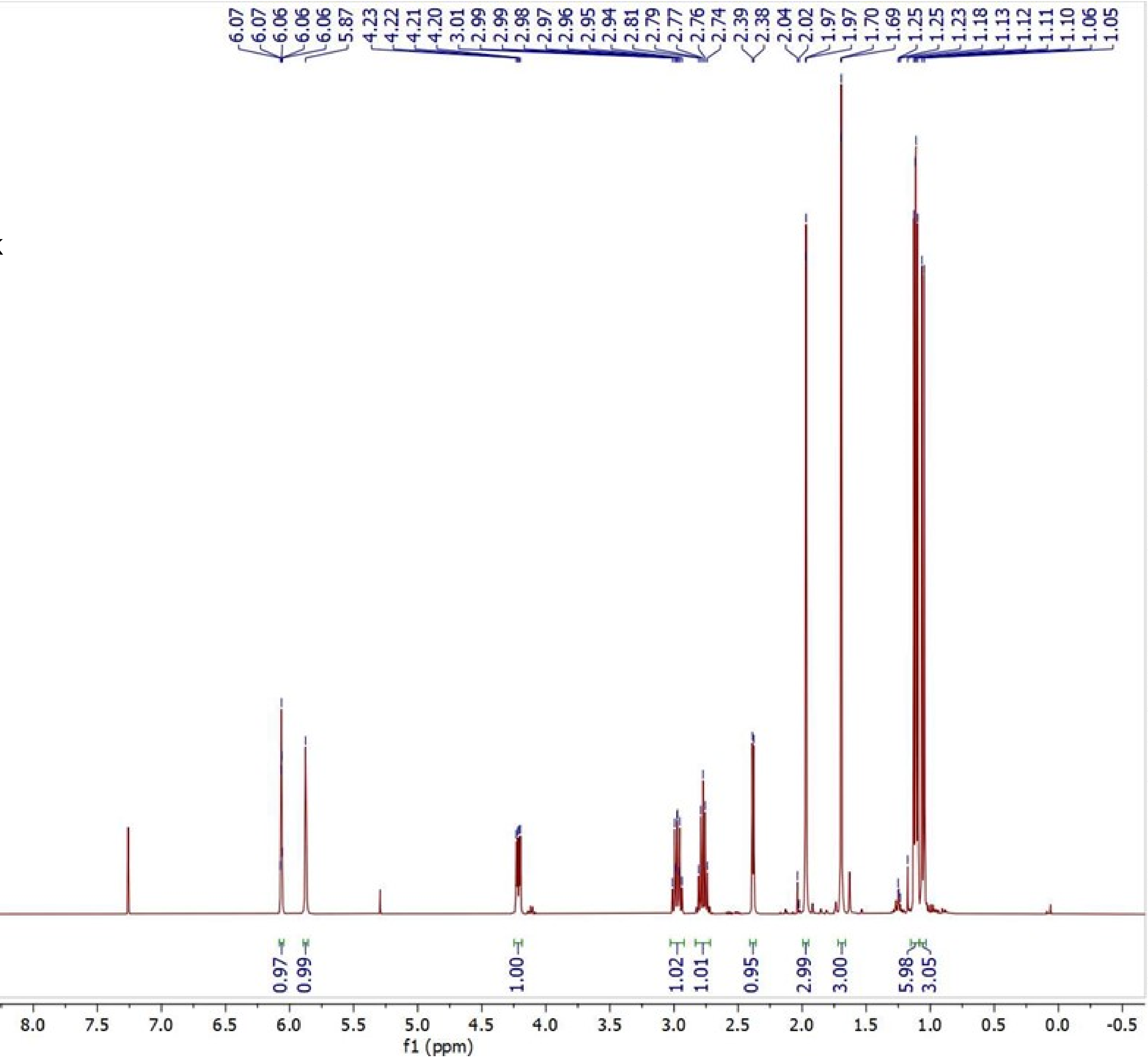

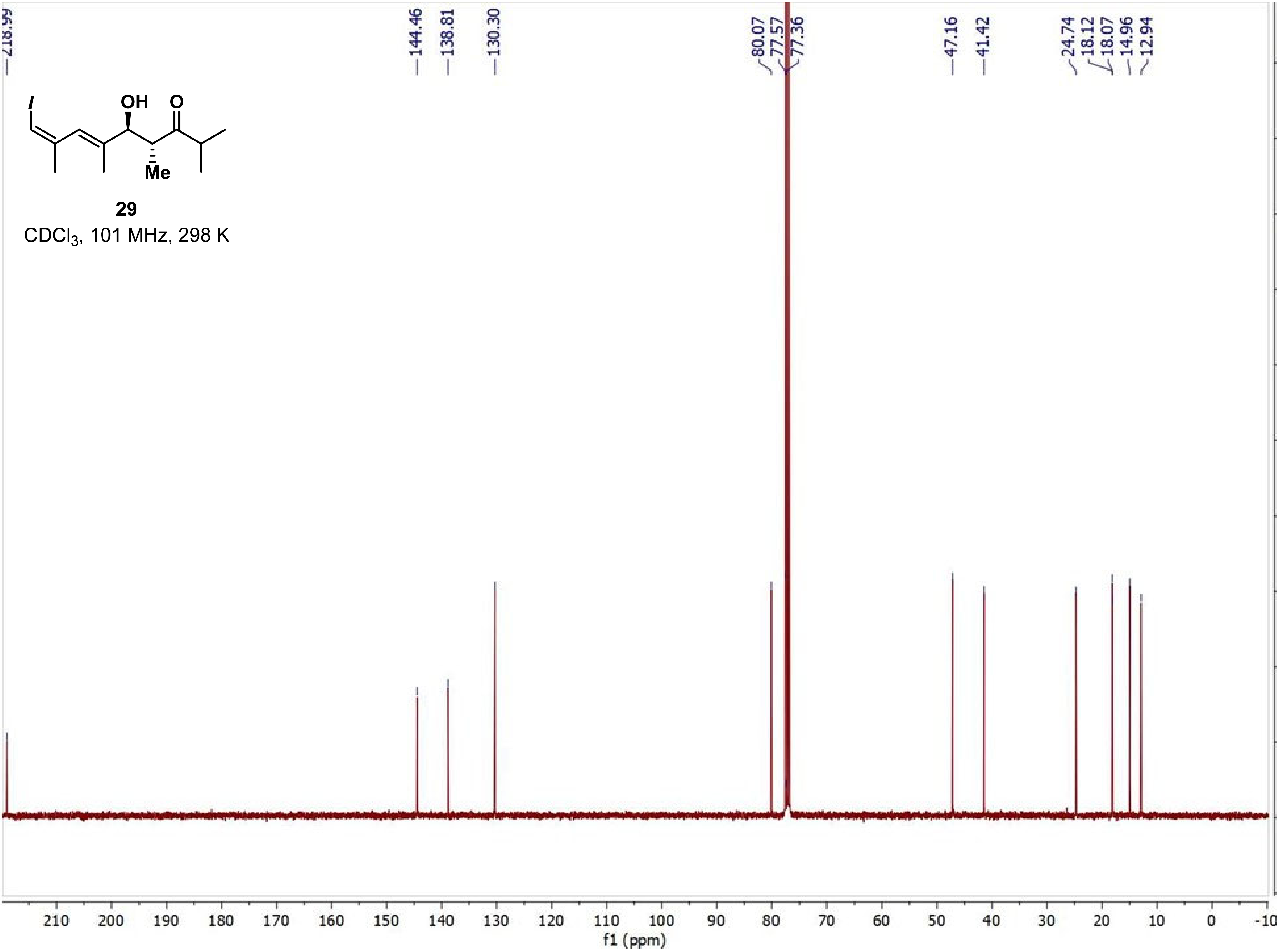

29

$CDCl_3$, 101 MHz, 298 K

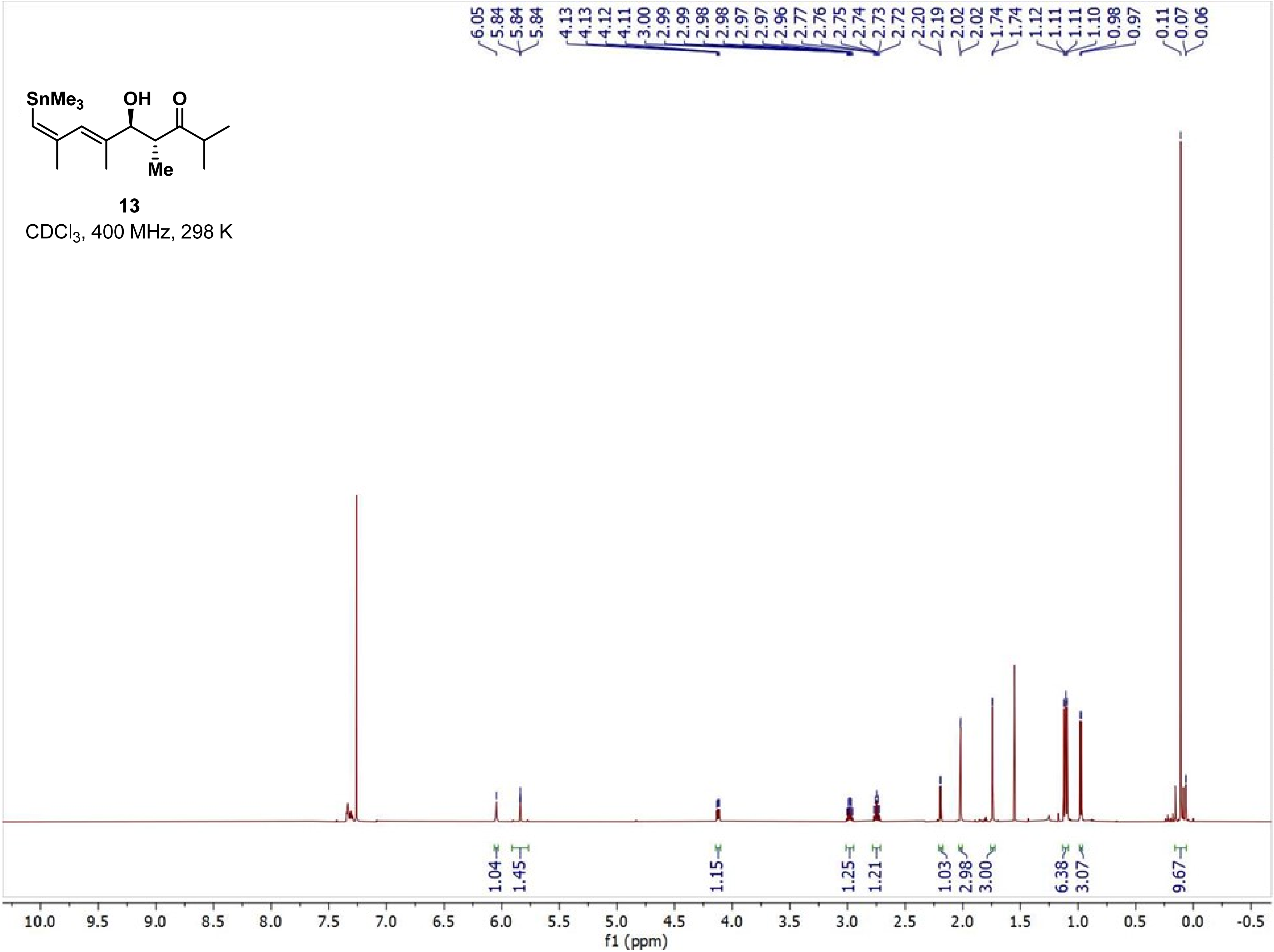
SnMe3
OH
O
Me
13
CDCl3, 400 MHz, 298 K
6.05
5.84
5.84
5.84
4.13
4.13
4.12
4.11
3.00
2.99
2.99
2.98
2.98
2.97
2.97
2.96
2.77
2.76
2.75
2.74
2.73
2.72
2.20
2.19
2.02
2.02
1.74
1.74
1.12
1.11
1.11
1.10
0.98
0.97
0.11
0.07
0.06
1.04
1.45
1.15
1.25
1.21
1.03
2.98
3.00
6.38
3.07
9.67
10.0 9.5 9.0 8.5 8.0 7.5 7.0 6.5 6.0 5.5 5.0 4.5 4.0 3.5 3.0 2.5 2.0 1.5 1.0 0.5 0.0 -0.5
f1 (ppm)

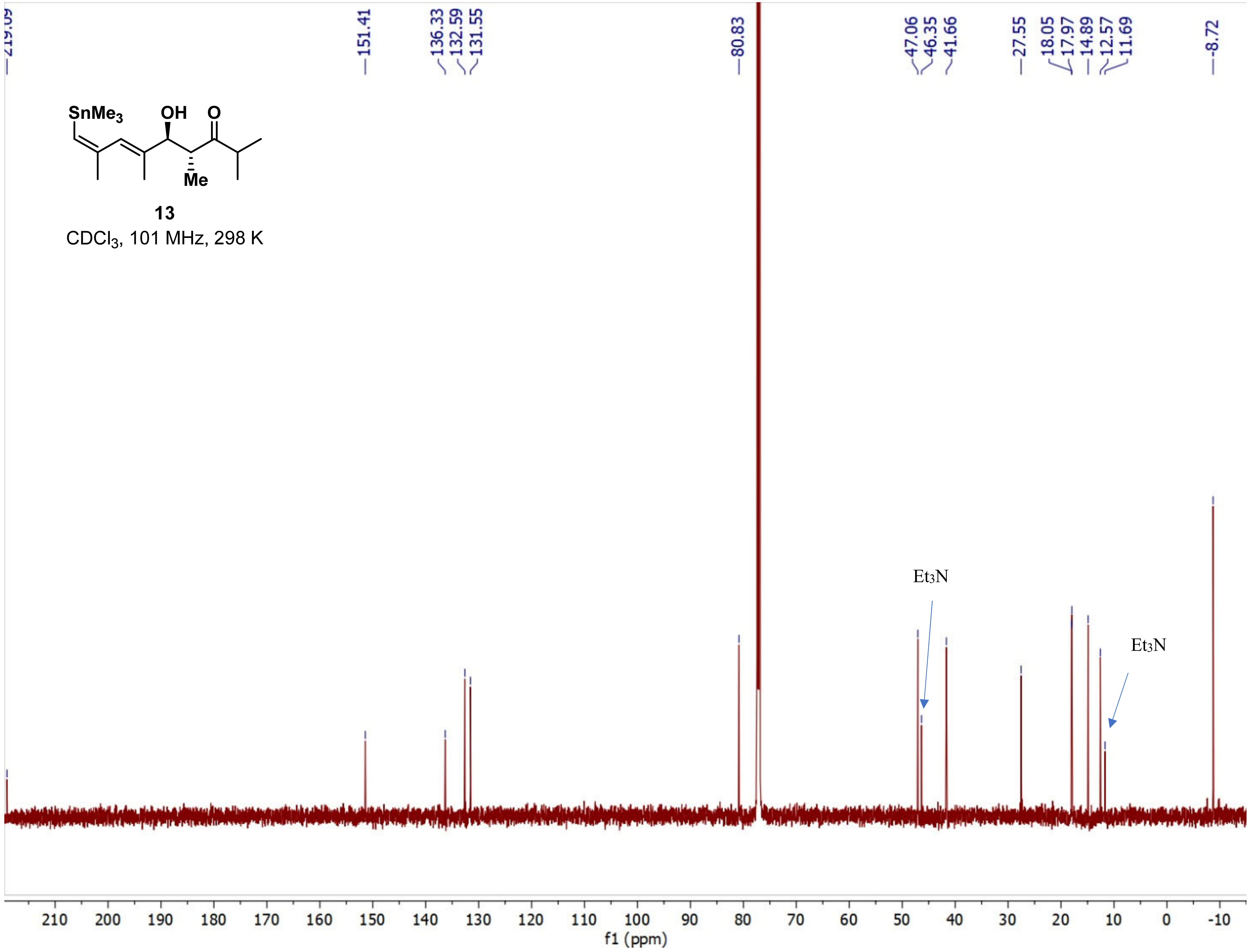

151.41
136.33
132.59
131.55
80.83
47.06
46.35
41.66
27.55
18.05
17.97
14.89
12.57
11.69
-8.72
SnMe3
OH
O
Me
13
CDCl3, 101 MHz, 298 K
Et3N
Et3N
210 200 190 180 170 160 150 140 130 120 110 100 90 80 70 60 50 40 30 20 10 0 -10
f1 (ppm)

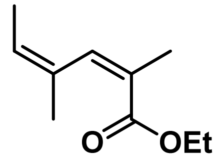

32

$CDCl_3$, 400 MHz, 298 K

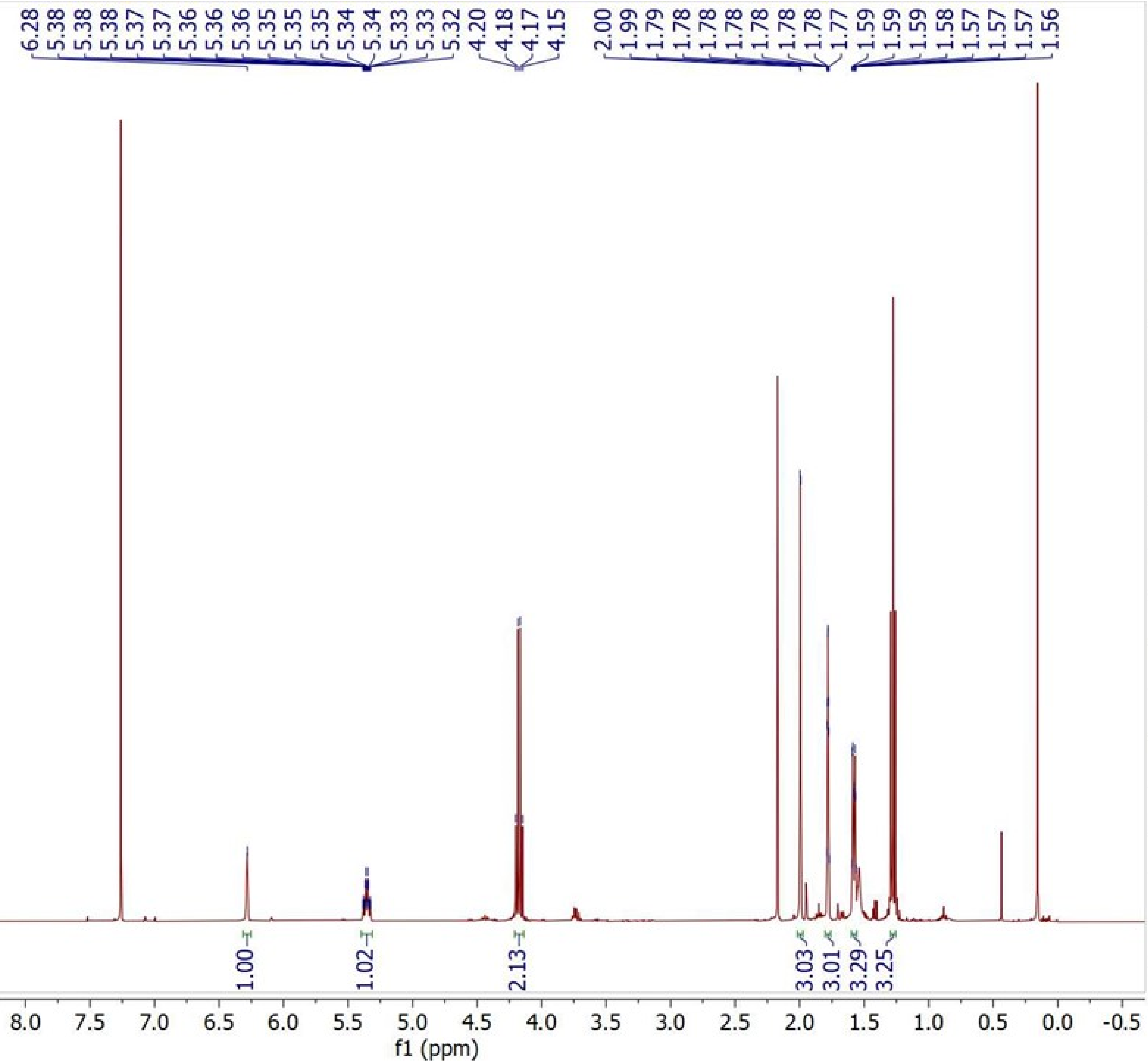

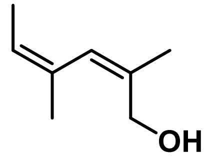

20

$CDCl_3$, 400 MHz, 298K

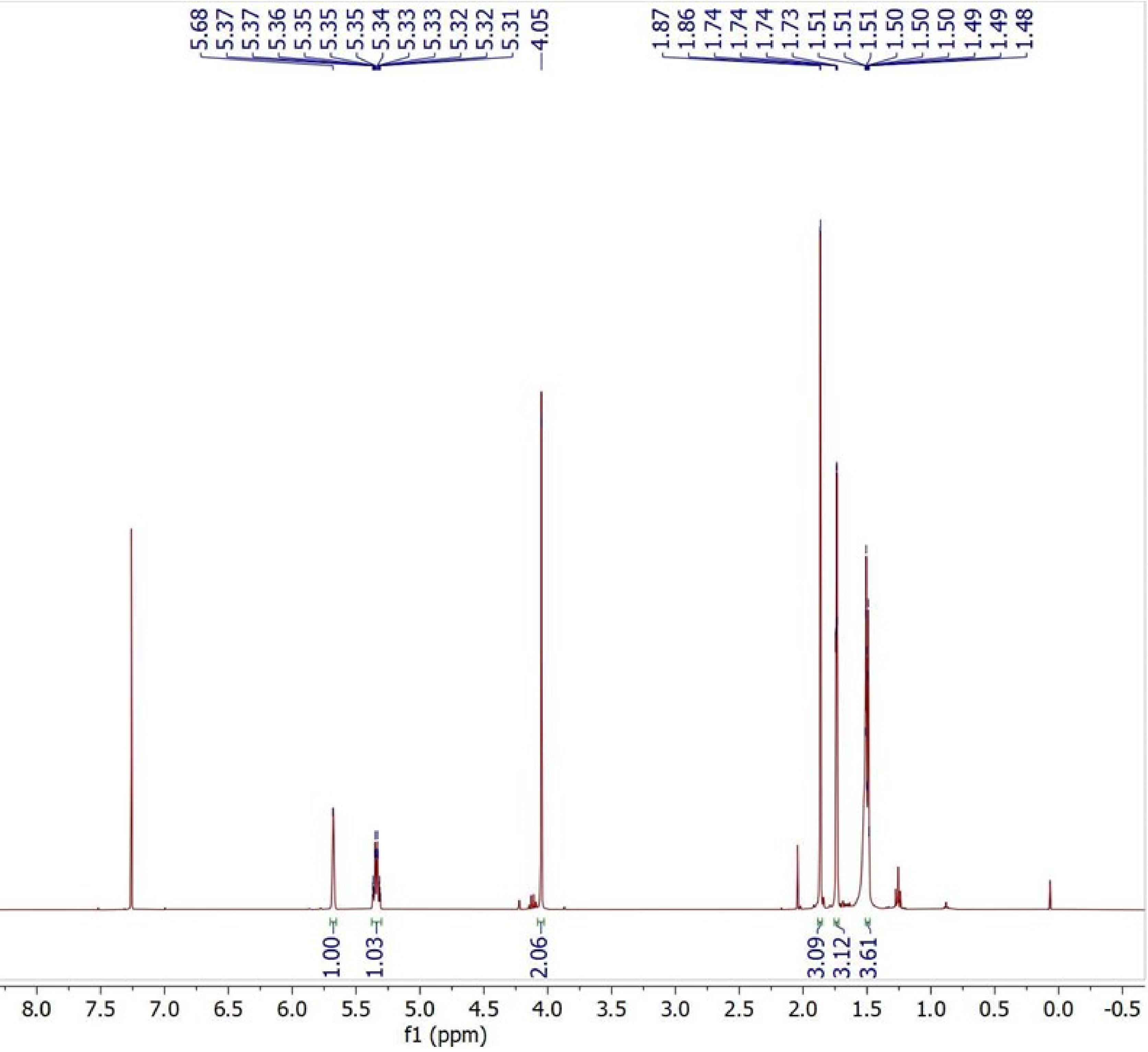

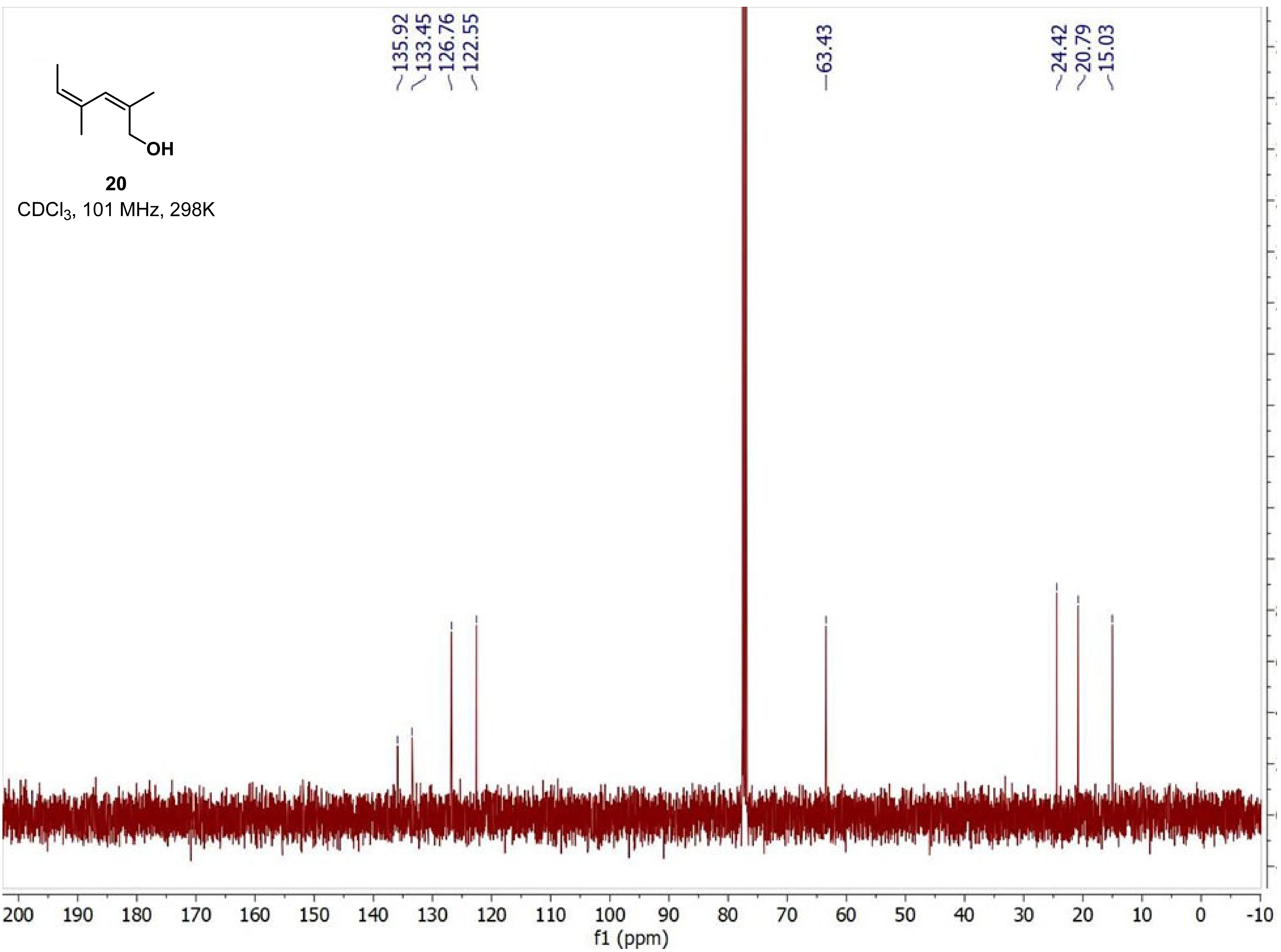

OH
20
CDCl3, 101 MHz, 298K
135.92
133.45
126.76
122.55
63.43
24.42
20.79
15.03
200
190
180
170
160
150
140
130
120
110
100
90
80
70
60
50
40
30
20
10
0
-10
f1 (ppm)

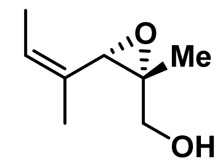

33

$CDCl_3$, 400 MHz, 298 K

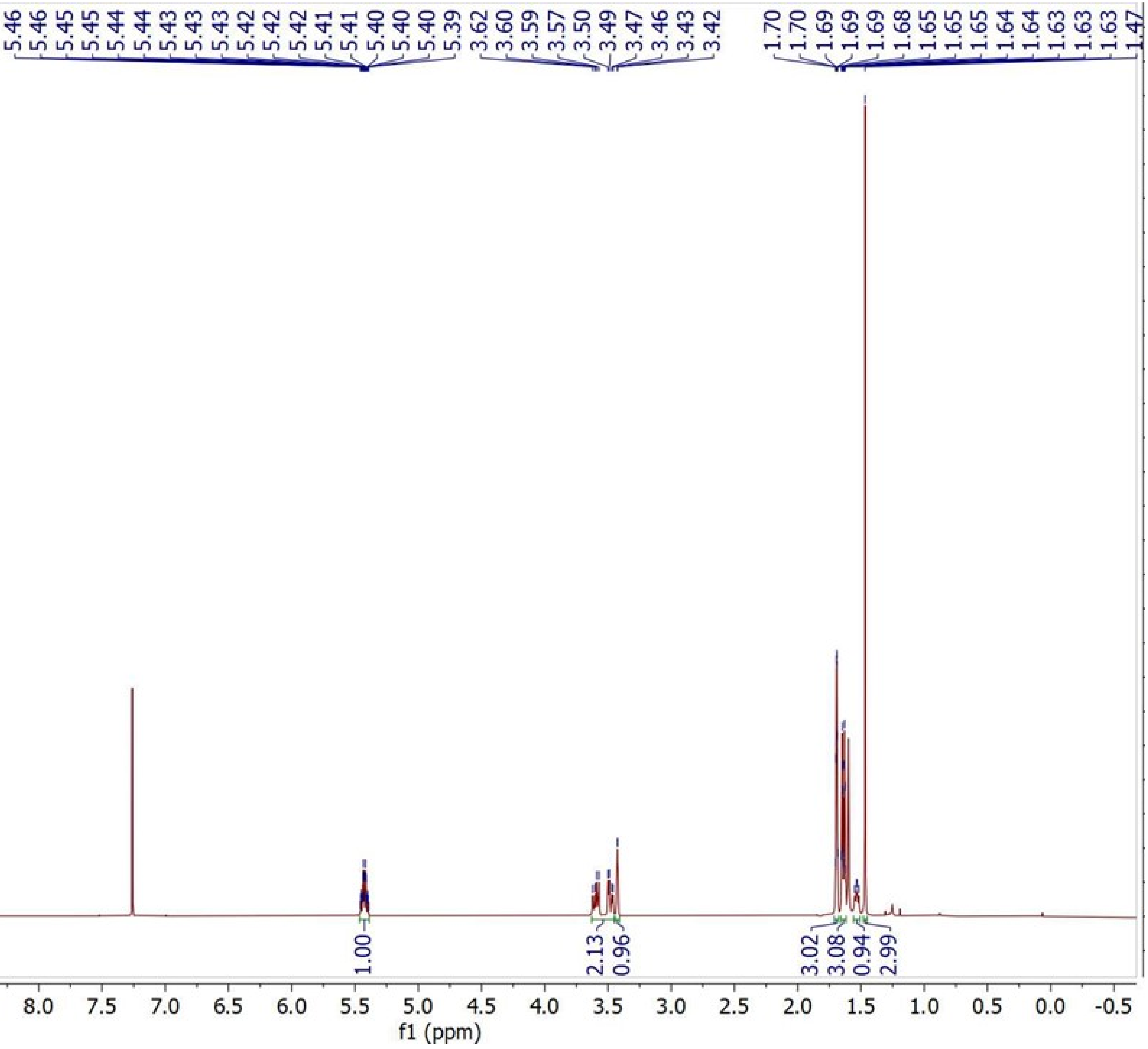

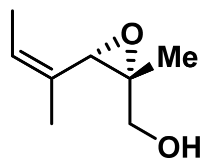

33

$CDCl_3$, 101 MHz, 298 K

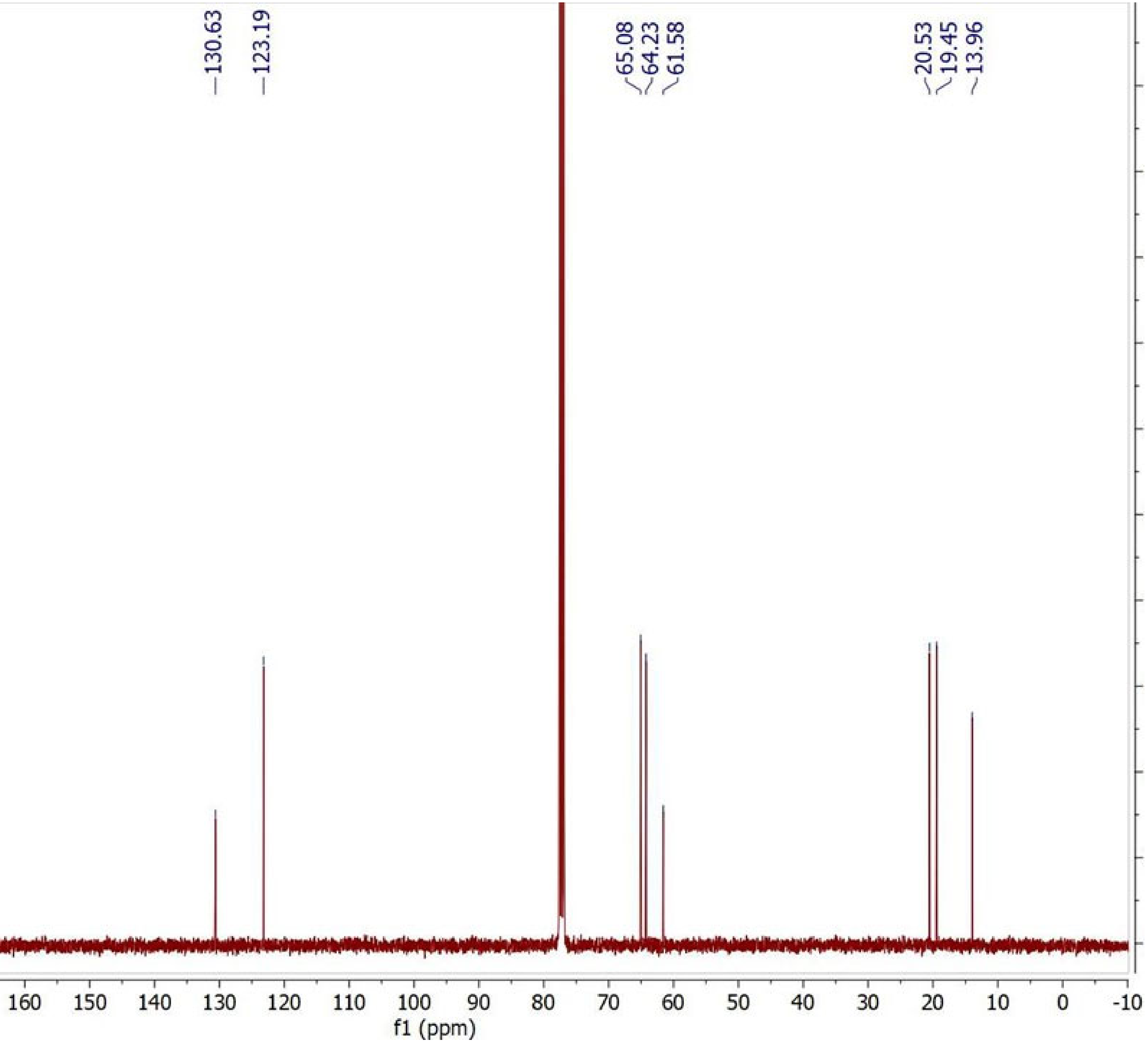

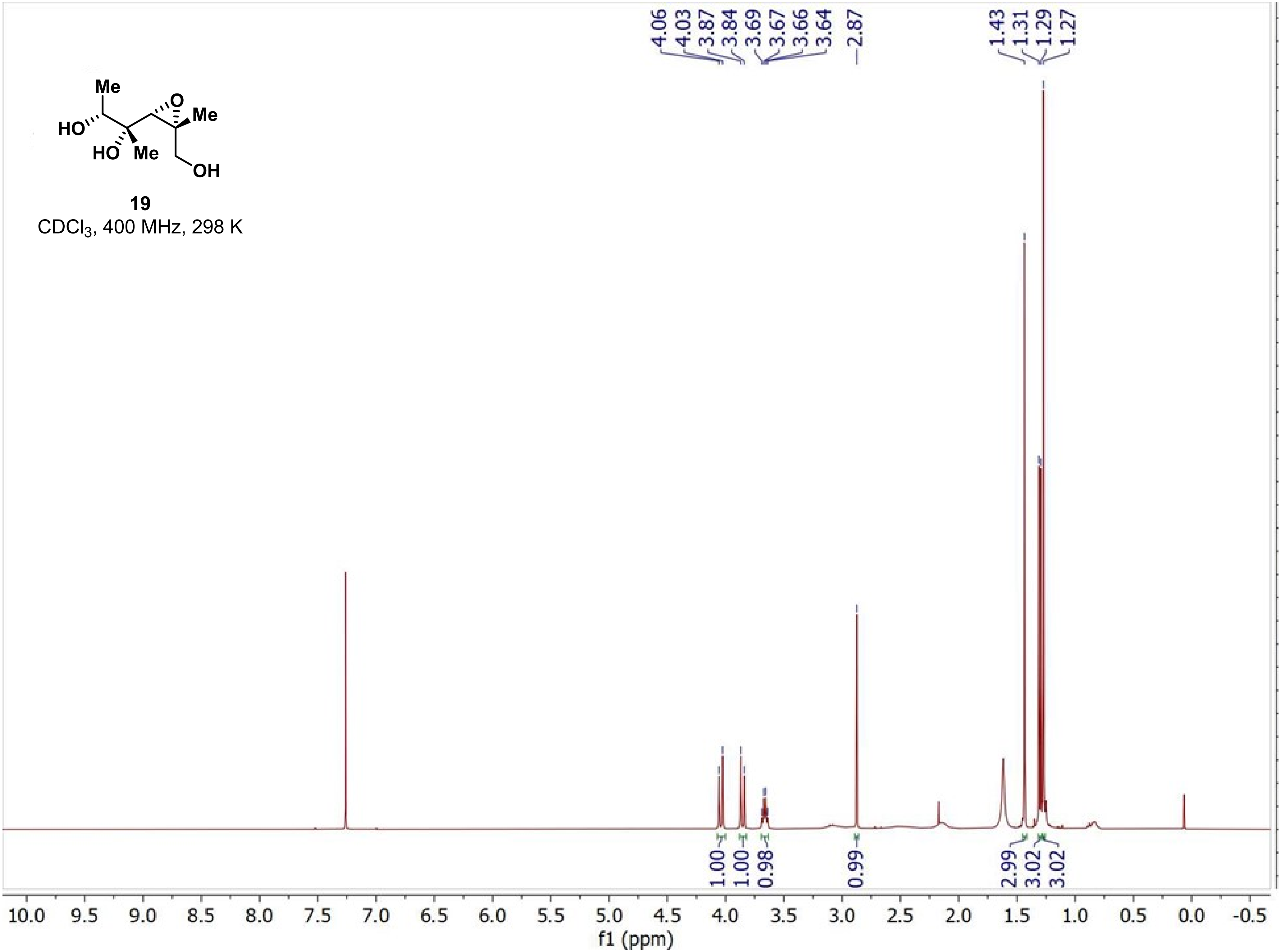

19

$CDCl_3$, 400 MHz, 298 K

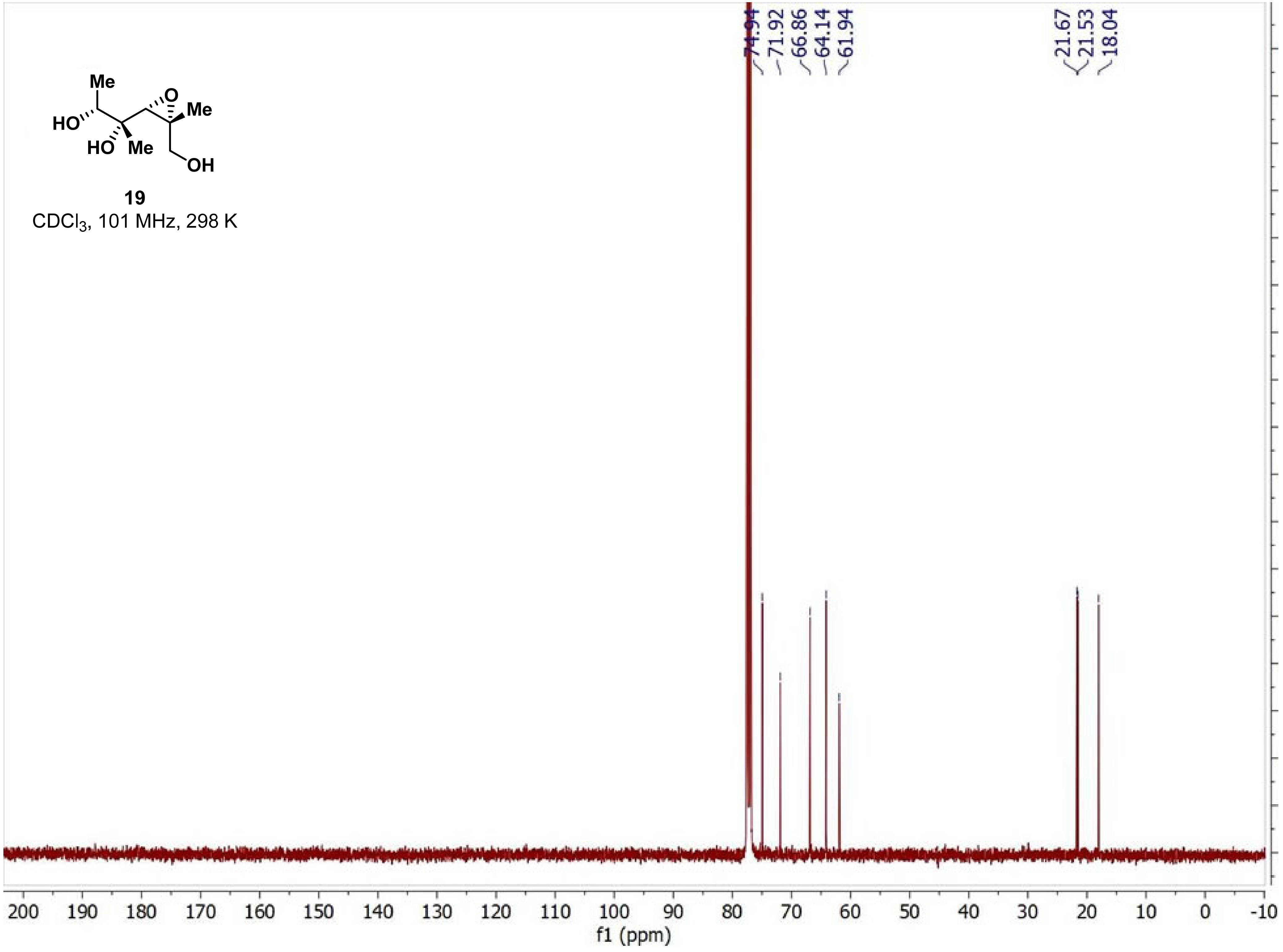

74.94
71.92
66.86
64.14
61.94
21.67
21.53
18.04
Me
HO
O
Me
HO
Me
OH
19
CDCl3, 101 MHz, 298 K
200 190 180 170 160 150 140 130 120 110 100 90 80 70 60 50 40 30 20 10 0 -10
f1 (ppm)

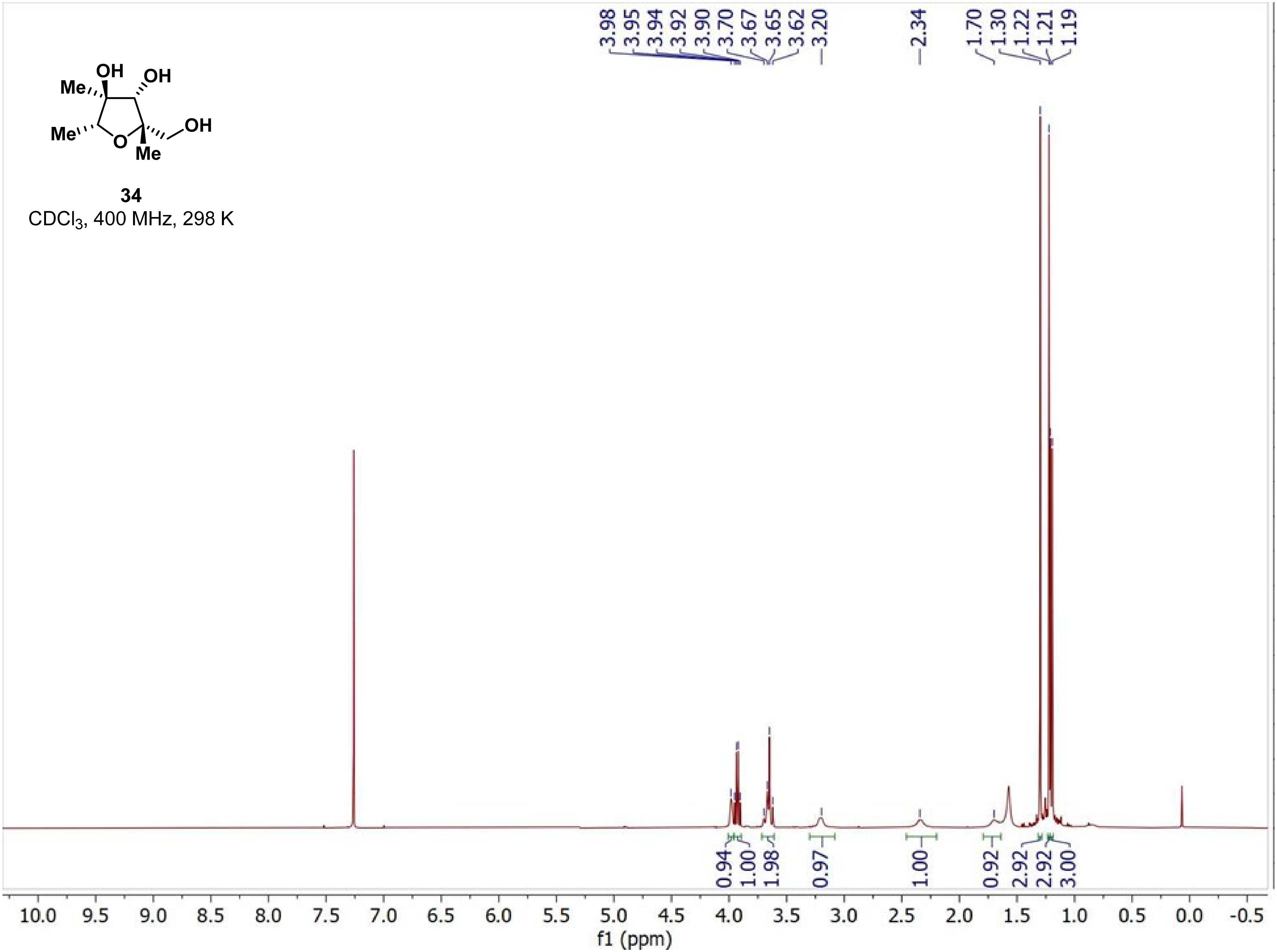

OH
OH
Me
Me
O
Me
OH
34
CDCl3, 400 MHz, 298 K
3.98 3.95 3.94 3.92 3.90 3.70 3.67 3.65 3.62 3.20
2.34
1.70 1.30 1.22 1.21 1.19
0.94 1.00 1.98 0.97 1.00 0.92 2.92 2.92 3.00
10.0 9.5 9.0 8.5 8.0 7.5 7.0 6.5 6.0 5.5 5.0 4.5 4.0 3.5 3.0 2.5 2.0 1.5 1.0 0.5 0.0 -0.5
f1 (ppm)

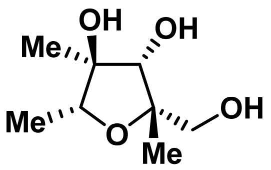

34

$CDCl_3$, 101 MHz, 298 K

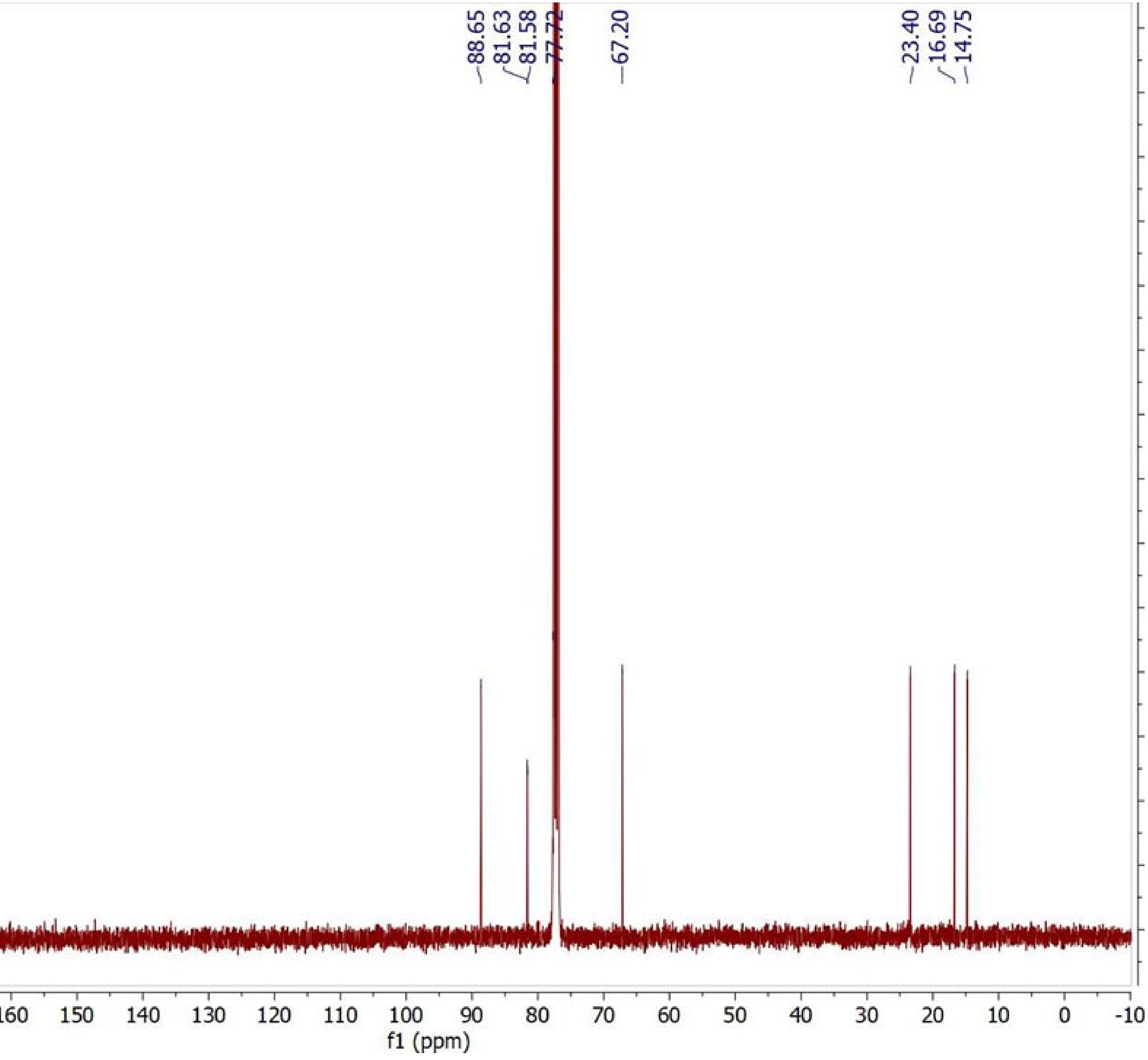

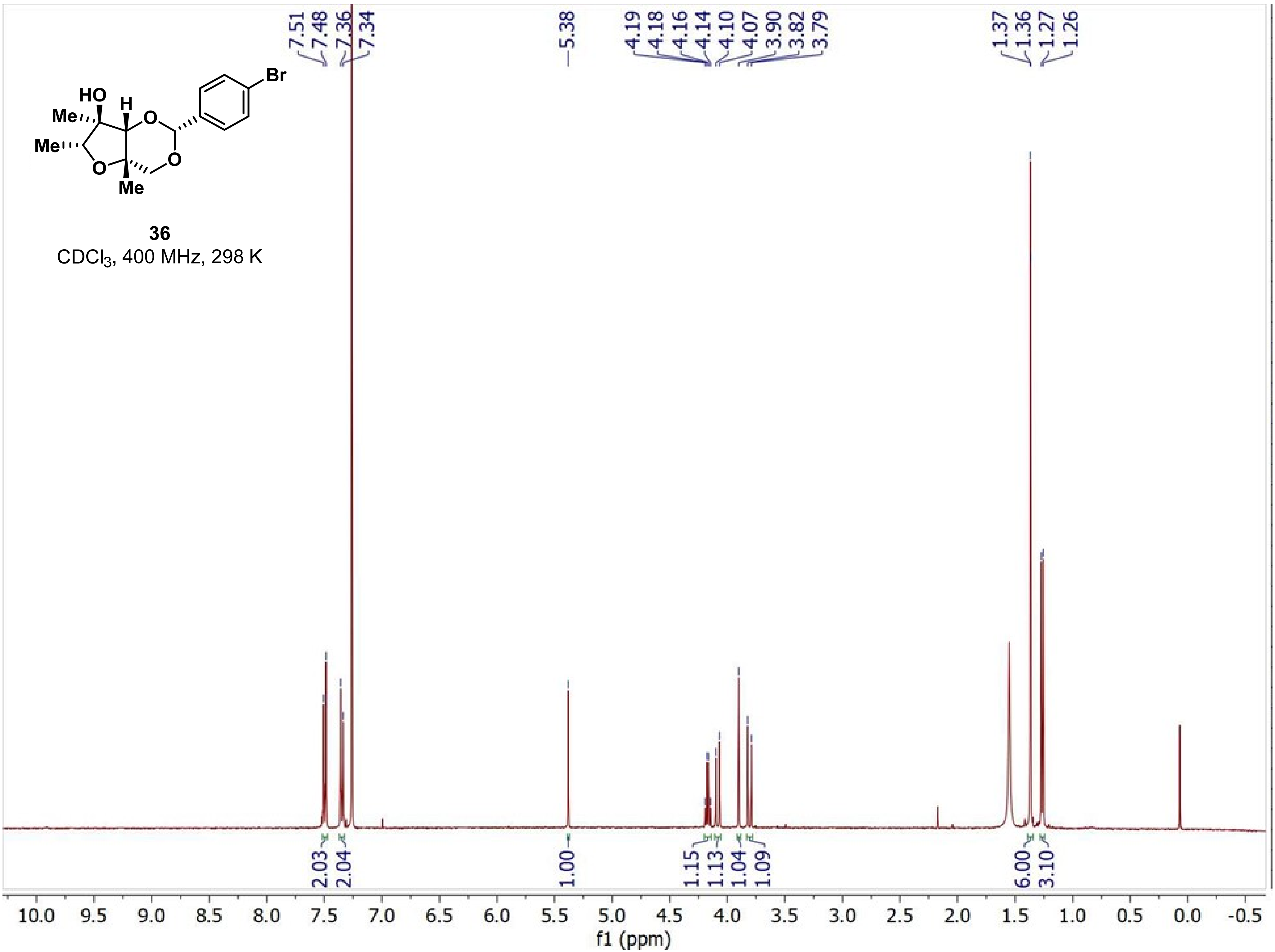
7.51
7.48
7.36
7.34
5.38
4.19
4.18
4.16
4.14
4.10
4.07
3.90
3.82
3.79
1.37
1.36
1.27
1.26
HO
H
Me
Me
Me
O
O
O
Br
36
CDCl3, 400 MHz, 298 K
2.03
2.04
1.00
1.15
1.13
1.04
1.09
6.00
3.10
10.0 9.5 9.0 8.5 8.0 7.5 7.0 6.5 6.0 5.5 5.0 4.5 4.0 3.5 3.0 2.5 2.0 1.5 1.0 0.5 0.0 -0.5
f1 (ppm)

36

$CDCl_3$, 151 MHz, 298 K

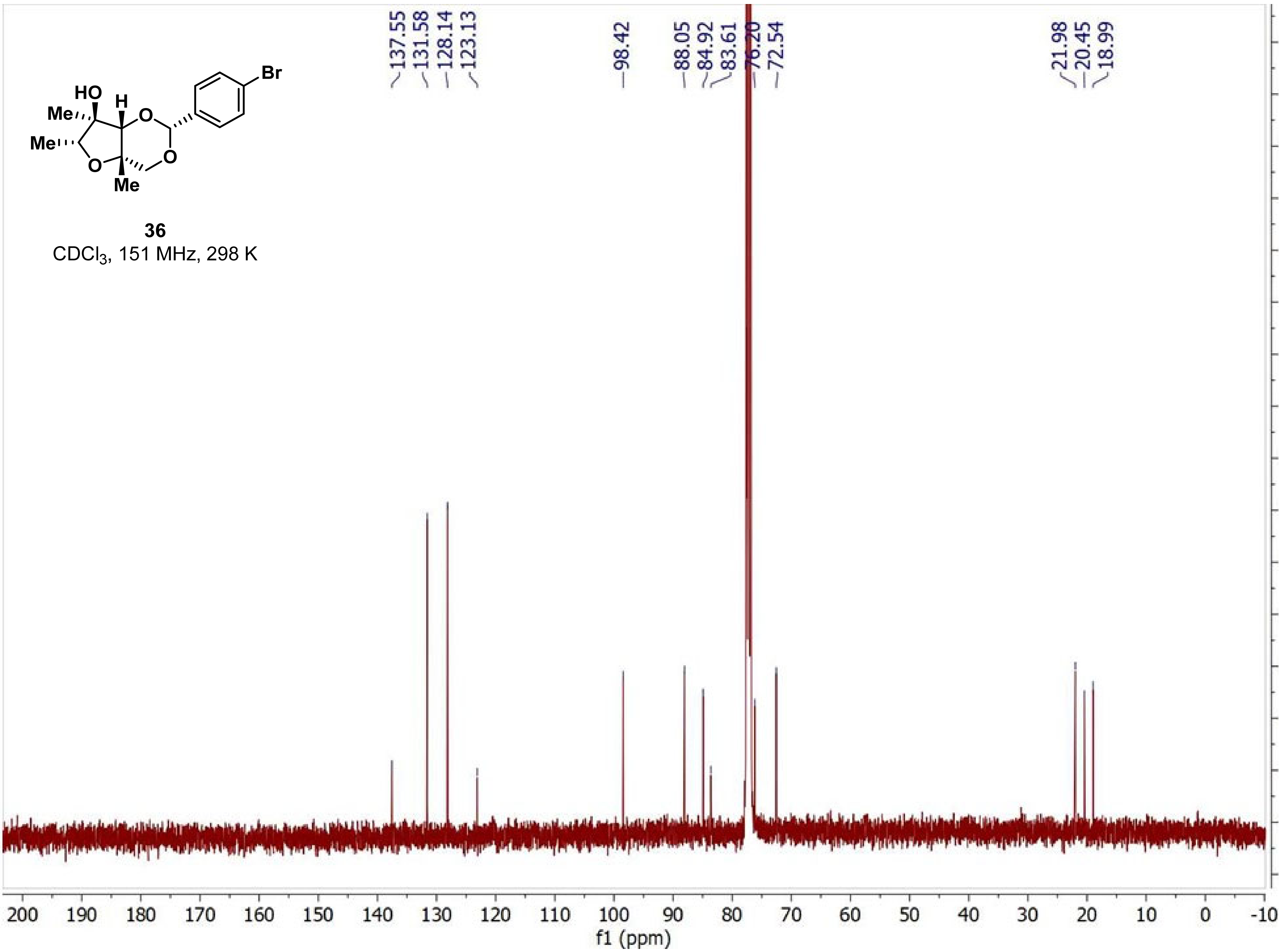

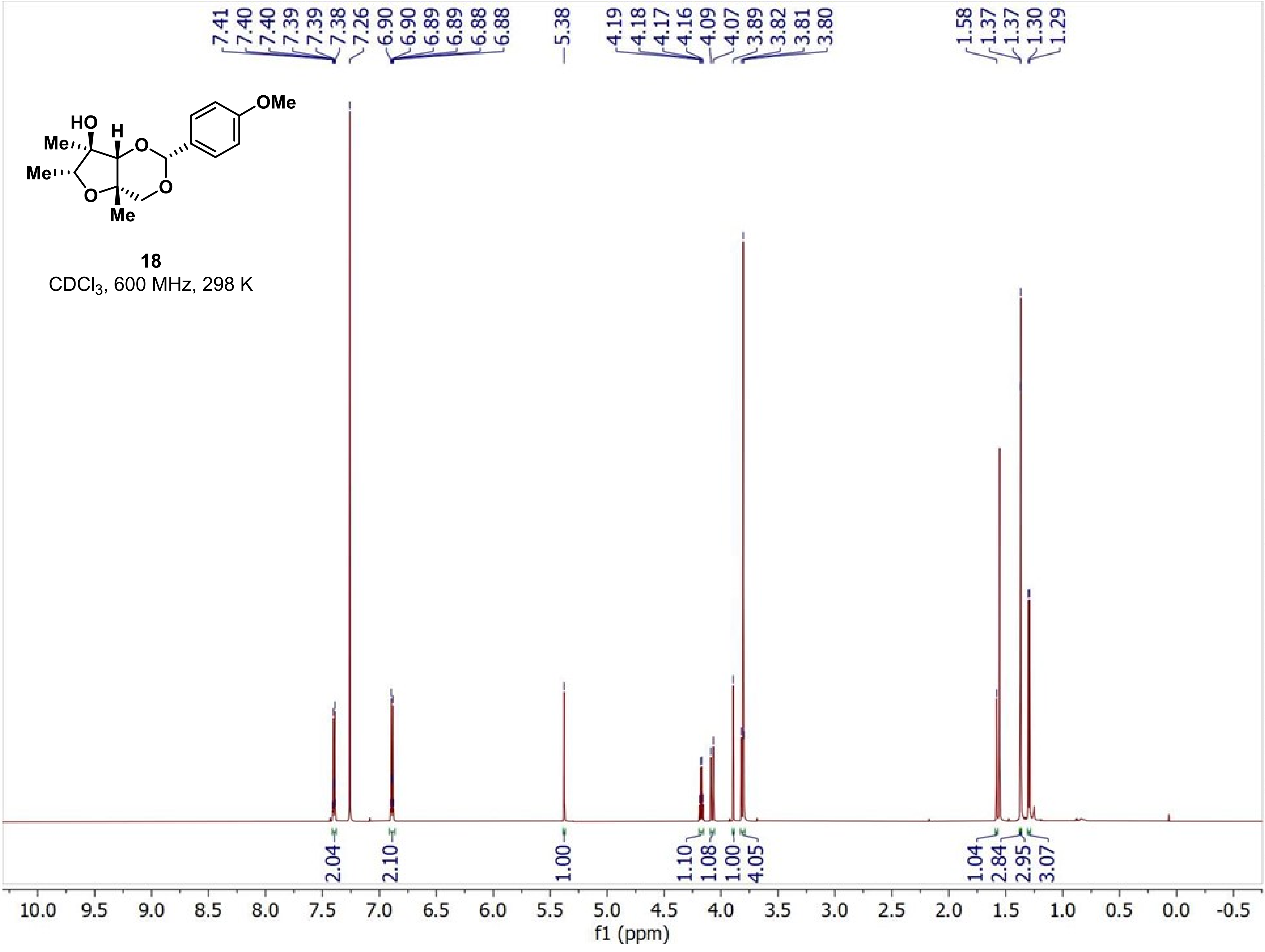

18

$CDCl_3$, 600 MHz, 298 K

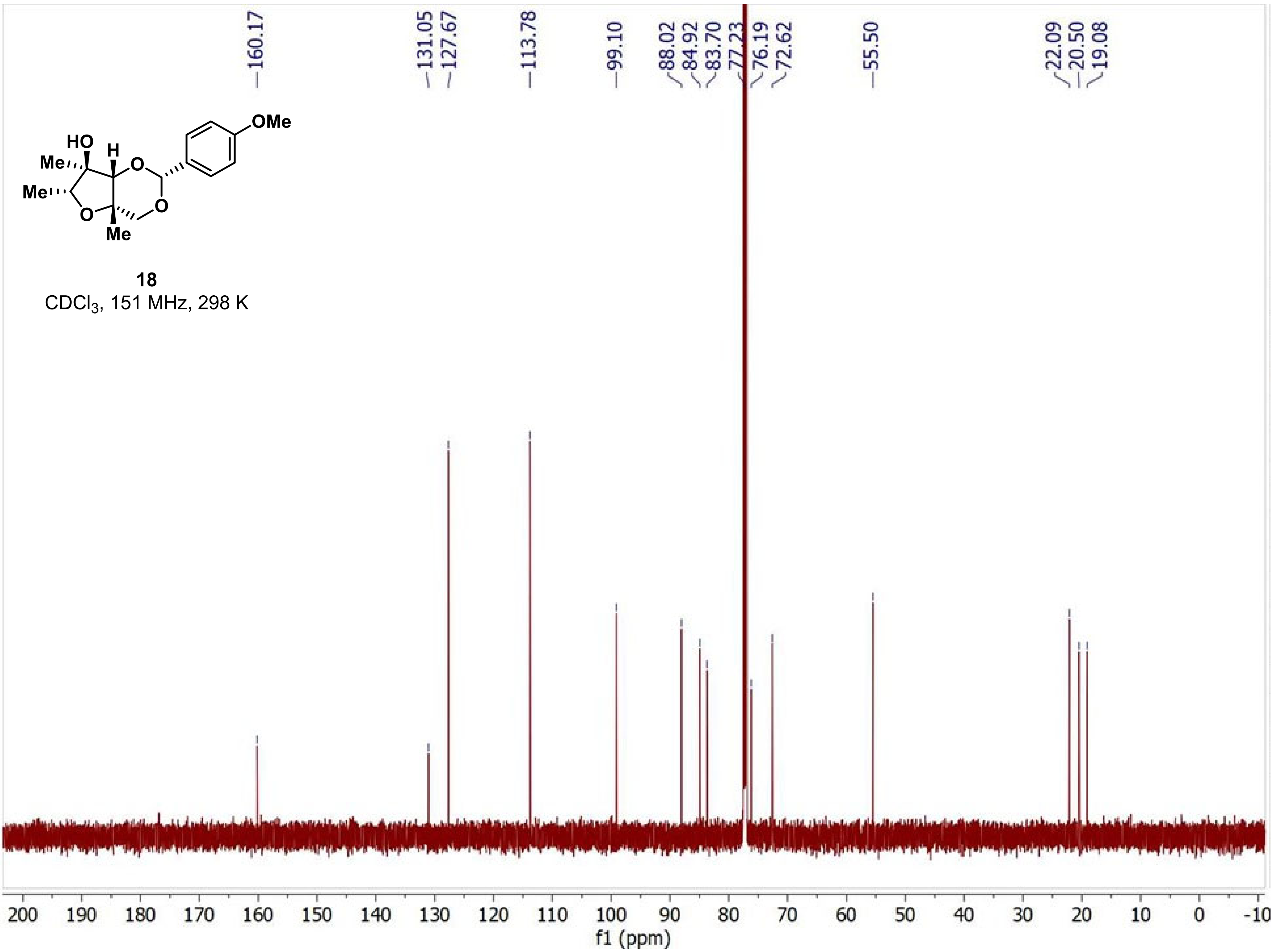

18
$CDCl_3$, 151 MHz, 298 K

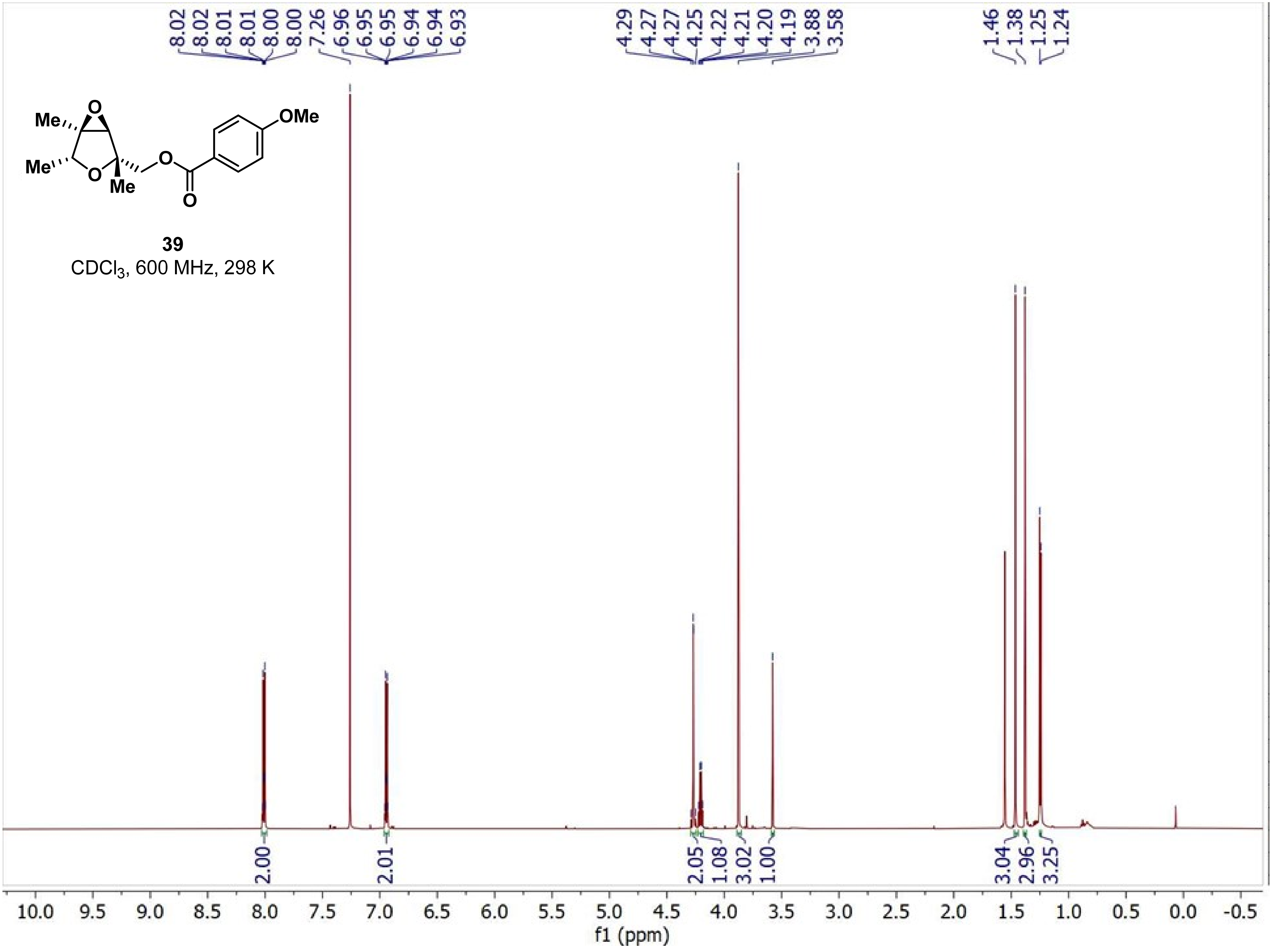

39

$CDCl_3$, 600 MHz, 298 K

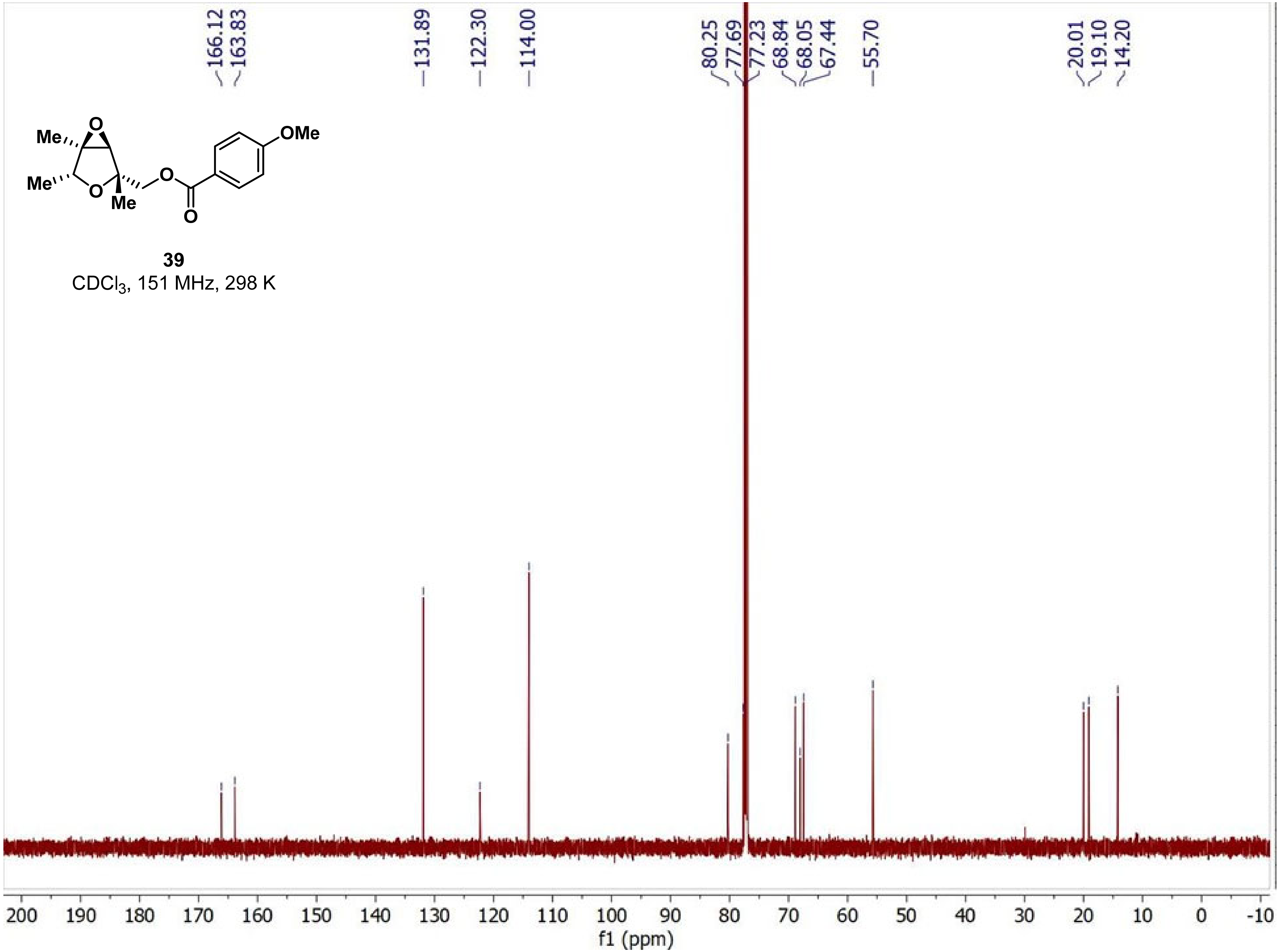

166.12
163.83
131.89
122.30
114.00
80.25
77.69
77.23
68.84
68.05
67.44
55.70
20.01
19.10
14.20
Me
O
Me
O
Me
O
O
OMe
39
CDCl3, 151 MHz, 298 K
200 190 180 170 160 150 140 130 120 110 100 90 80 70 60 50 40 30 20 10 0 -10
f1 (ppm)

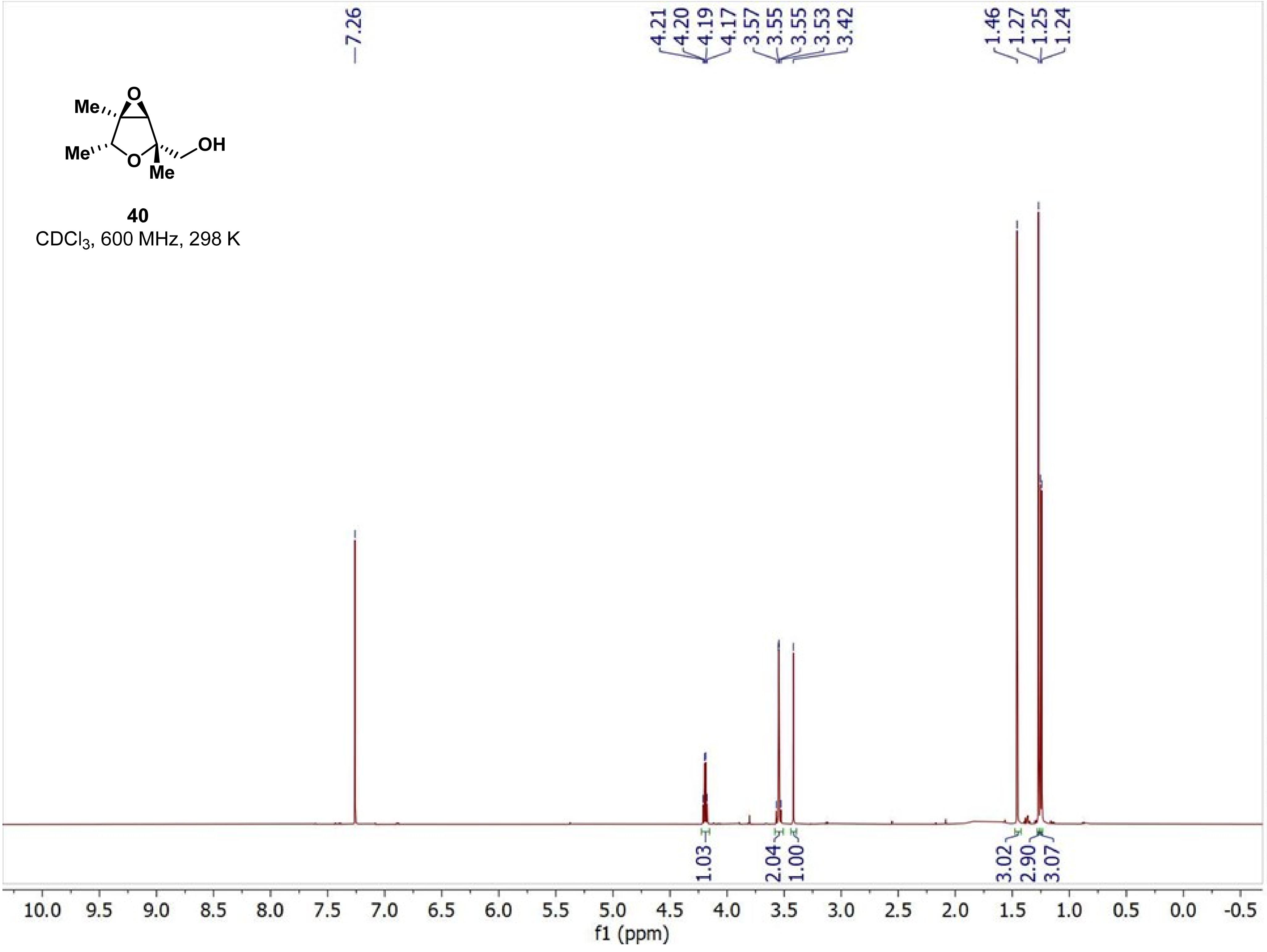

Me
Me
O
O
Me
OH
40
CDCl3, 600 MHz, 298 K
7.26
4.21
4.20
4.19
4.17
3.57
3.55
3.55
3.53
3.42
1.46
1.27
1.25
1.24
1.03
2.04
1.00
3.02
2.90
3.07
10.0 9.5 9.0 8.5 8.0 7.5 7.0 6.5 6.0 5.5 5.0 4.5 4.0 3.5 3.0 2.5 2.0 1.5 1.0 0.5 0.0 -0.5
f1 (ppm)

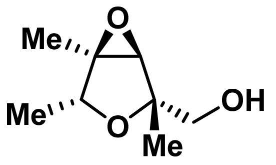

40

$CDCl_3$, 151 MHz, 298 K

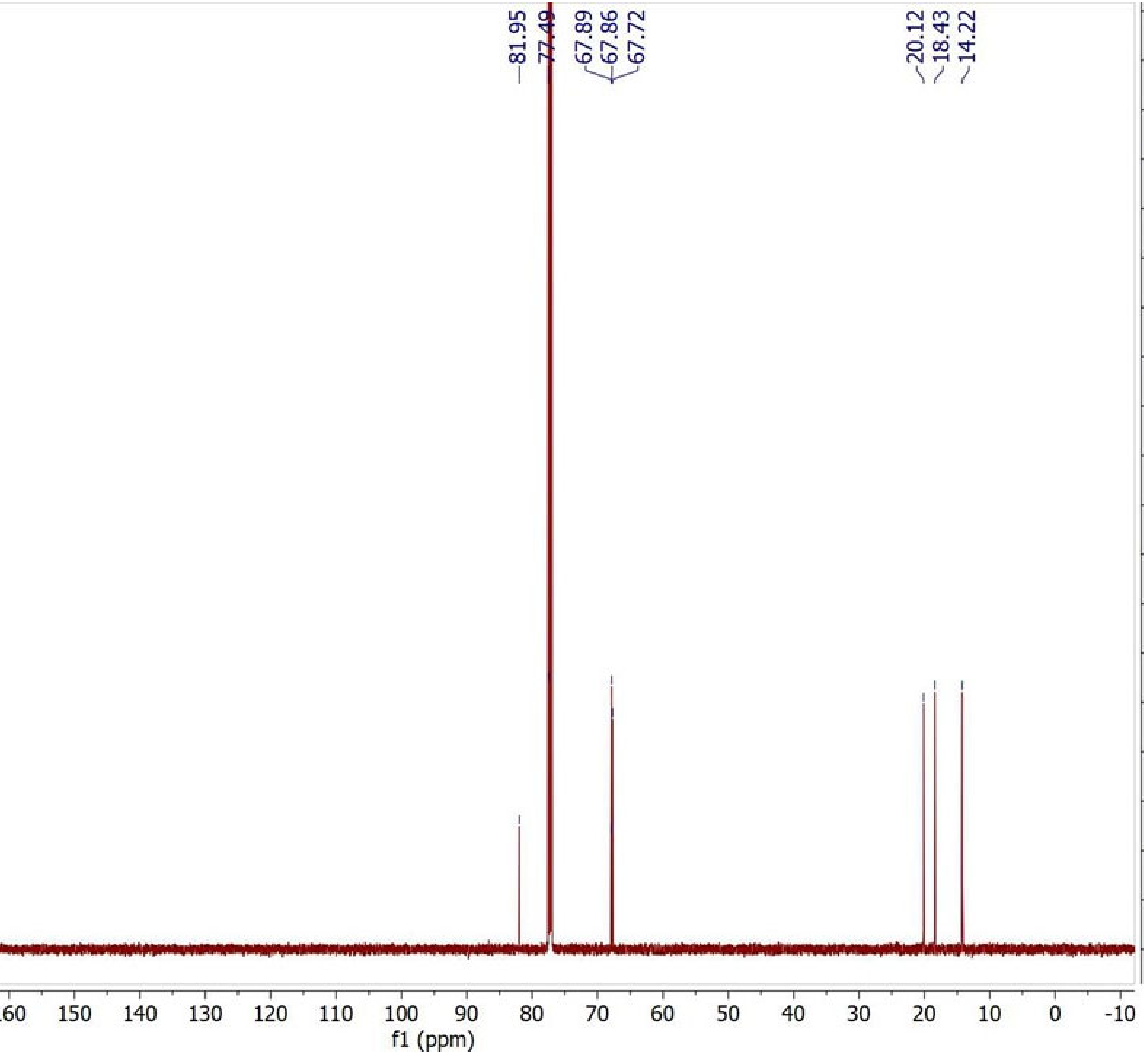

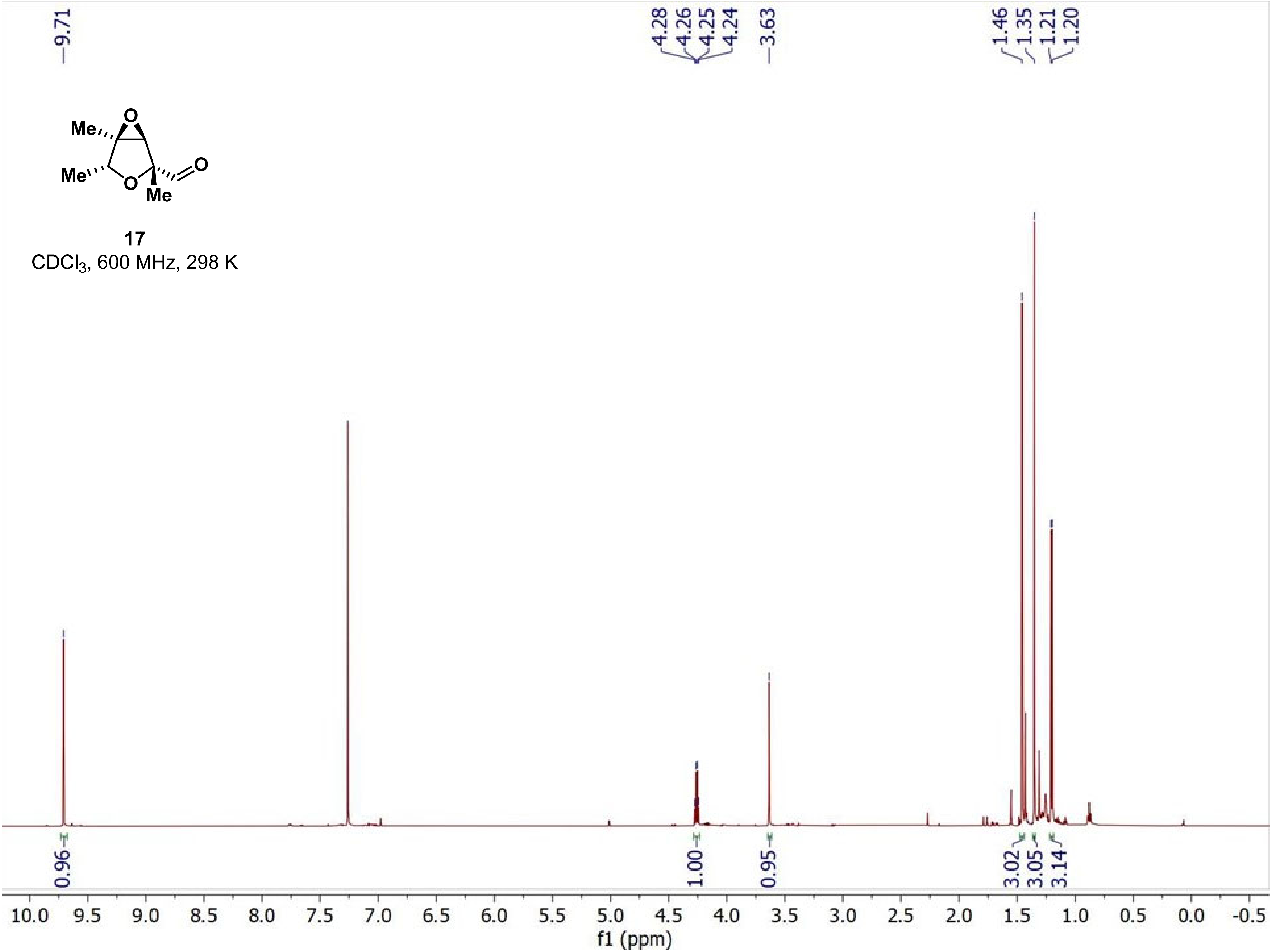
9.71
4.28
4.26
4.25
4.24
3.63
1.46
1.35
1.21
1.20
Me
O
Me
O
O
Me
17
CDCl3, 600 MHz, 298 K
0.96
1.00
0.95
3.02
3.05
3.14
10.0 9.5 9.0 8.5 8.0 7.5 7.0 6.5 6.0 5.5 5.0 4.5 4.0 3.5 3.0 2.5 2.0 1.5 1.0 0.5 0.0 -0.5
f1 (ppm)

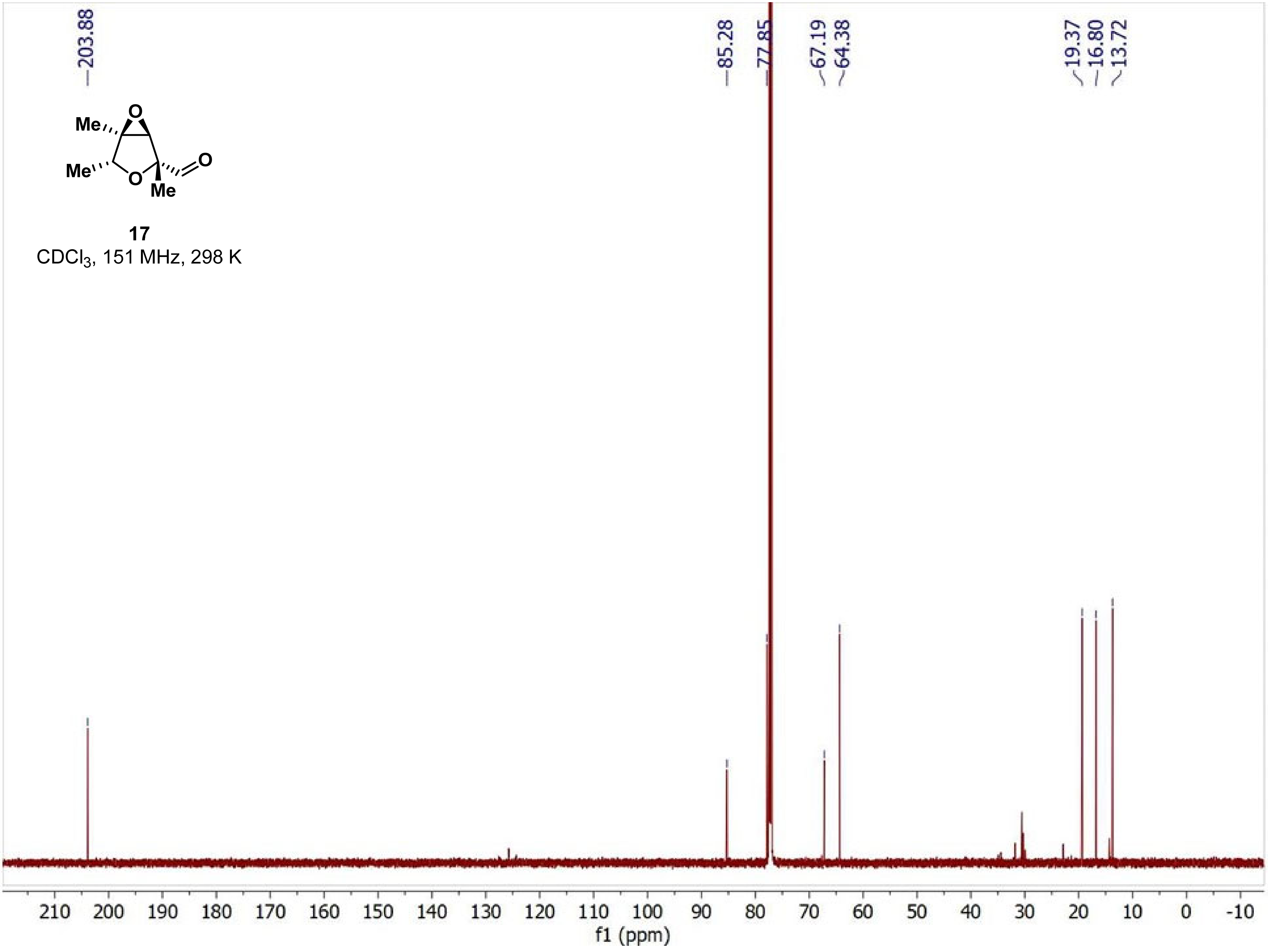
203.88
85.28
77.85
67.19
64.38
19.37
16.80
13.72
17
CDCl3, 151 MHz, 298 K
f1 (ppm)

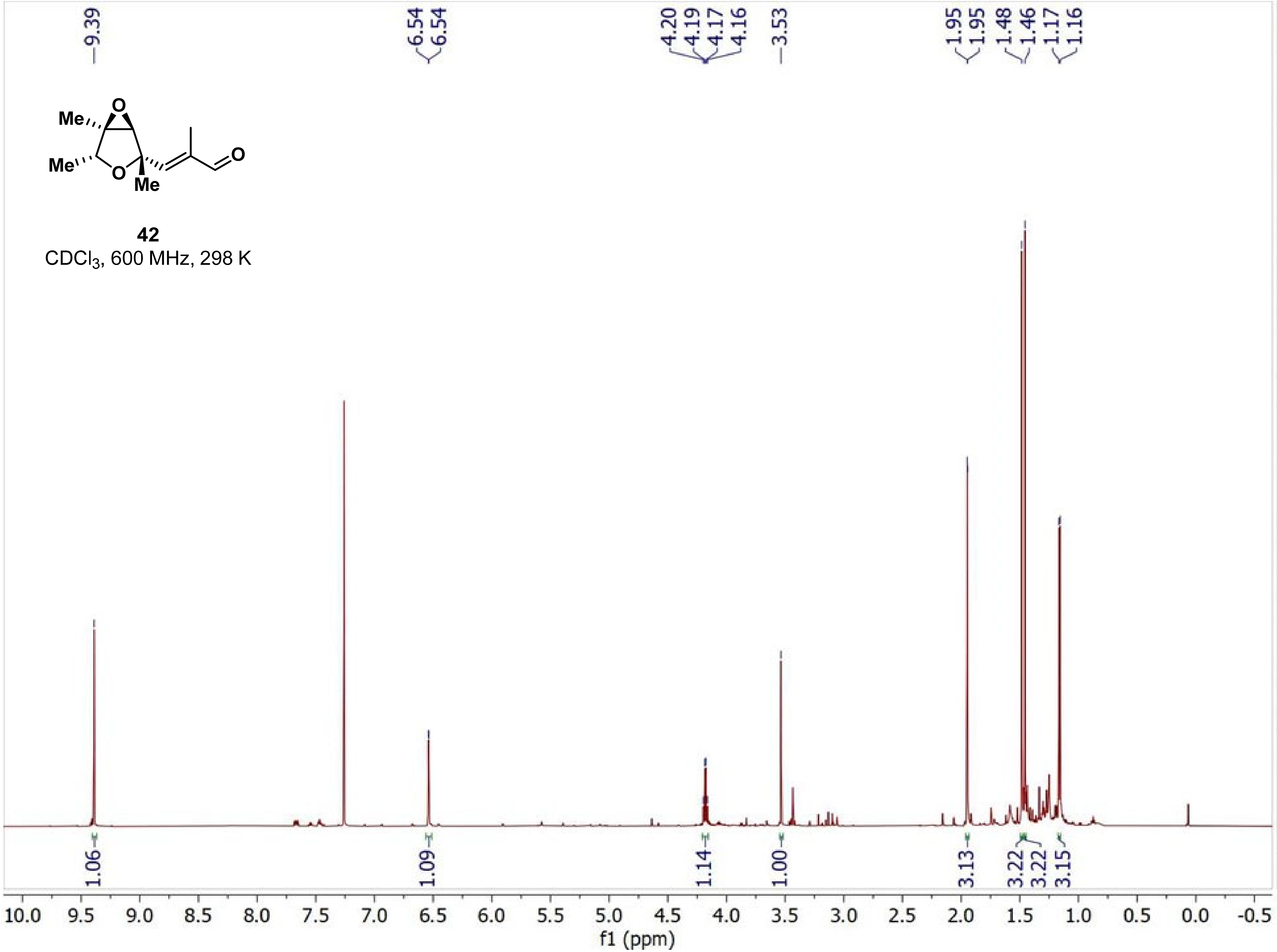

9.39
6.54
6.54
4.20
4.19
4.17
4.16
3.53
1.95
1.95
1.48
1.46
1.17
1.16
O
Me
O
Me
O
Me
Me
42
CDCl3, 600 MHz, 298 K
1.06
1.09
1.14
1.00
3.13
3.22
3.22
3.15
10.0 9.5 9.0 8.5 8.0 7.5 7.0 6.5 6.0 5.5 5.0 4.5 4.0 3.5 3.0 2.5 2.0 1.5 1.0 0.5 0.0 -0.5
f1 (ppm)

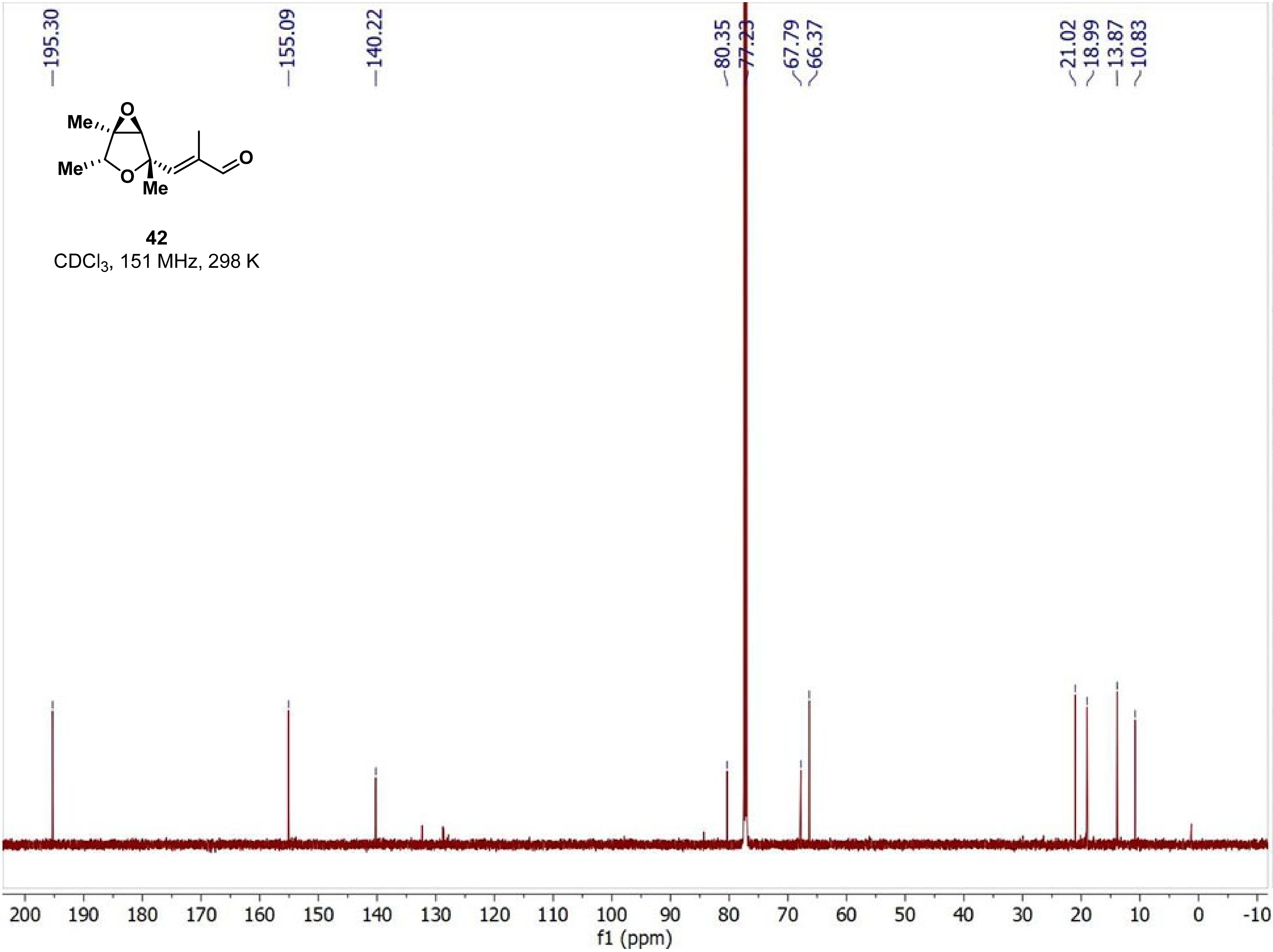

42

$CDCl_3$, 151 MHz, 298 K

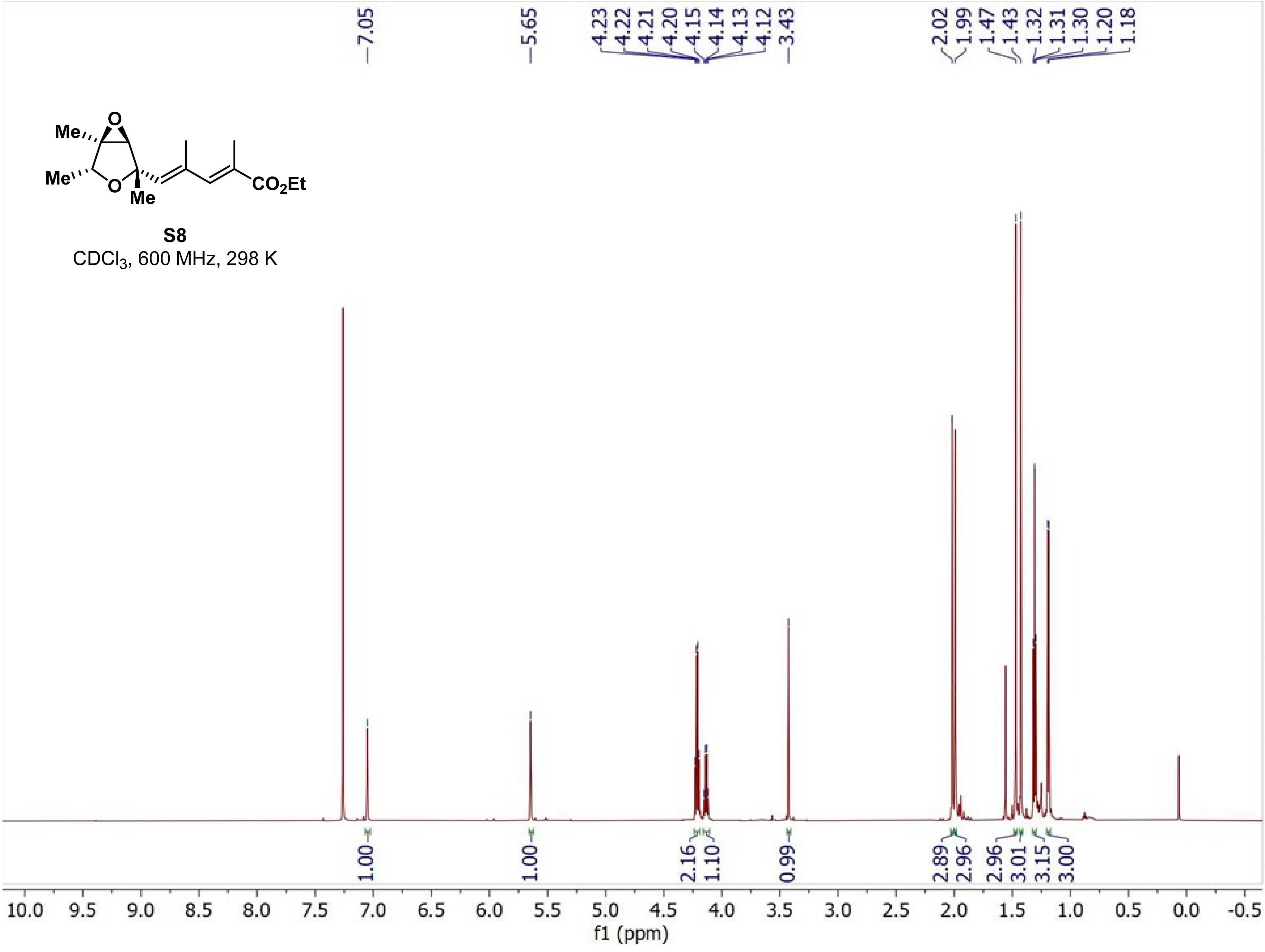

S8
CDCl$_3$, 600 MHz, 298 K
7.05
5.65
4.23 4.22 4.21 4.20 4.15 4.14 4.13 4.12
3.43
2.02 1.99 1.47 1.43 1.32 1.31 1.30 1.20 1.18
1.00
1.00
2.16 1.10
0.99
2.89 2.96 2.96 3.01 3.15 3.00
10.0 9.5 9.0 8.5 8.0 7.5 7.0 6.5 6.0 5.5 5.0 4.5 4.0 3.5 3.0 2.5 2.0 1.5 1.0 0.5 0.0 -0.5
f1 (ppm)

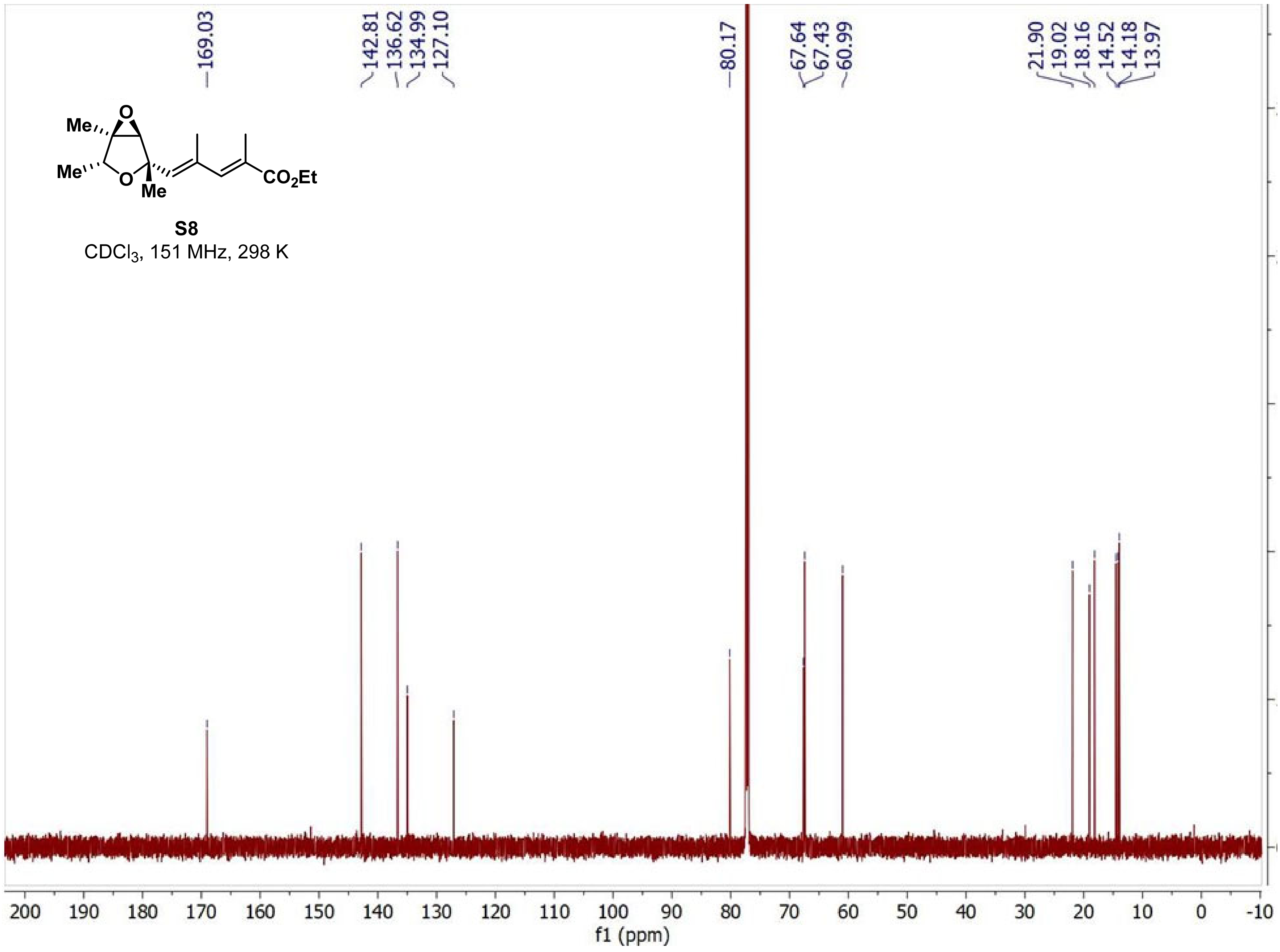

169.03
142.81
136.62
134.99
127.10
80.17
67.64
67.43
60.99
21.90
19.02
18.16
14.52
14.18
13.97
Me
O
Me
O
Me
CO_2Et
S8
$CDCl_3$, 151 MHz, 298 K
200 190 180 170 160 150 140 130 120 110 100 90 80 70 60 50 40 30 20 10 0 -10
f1 (ppm)

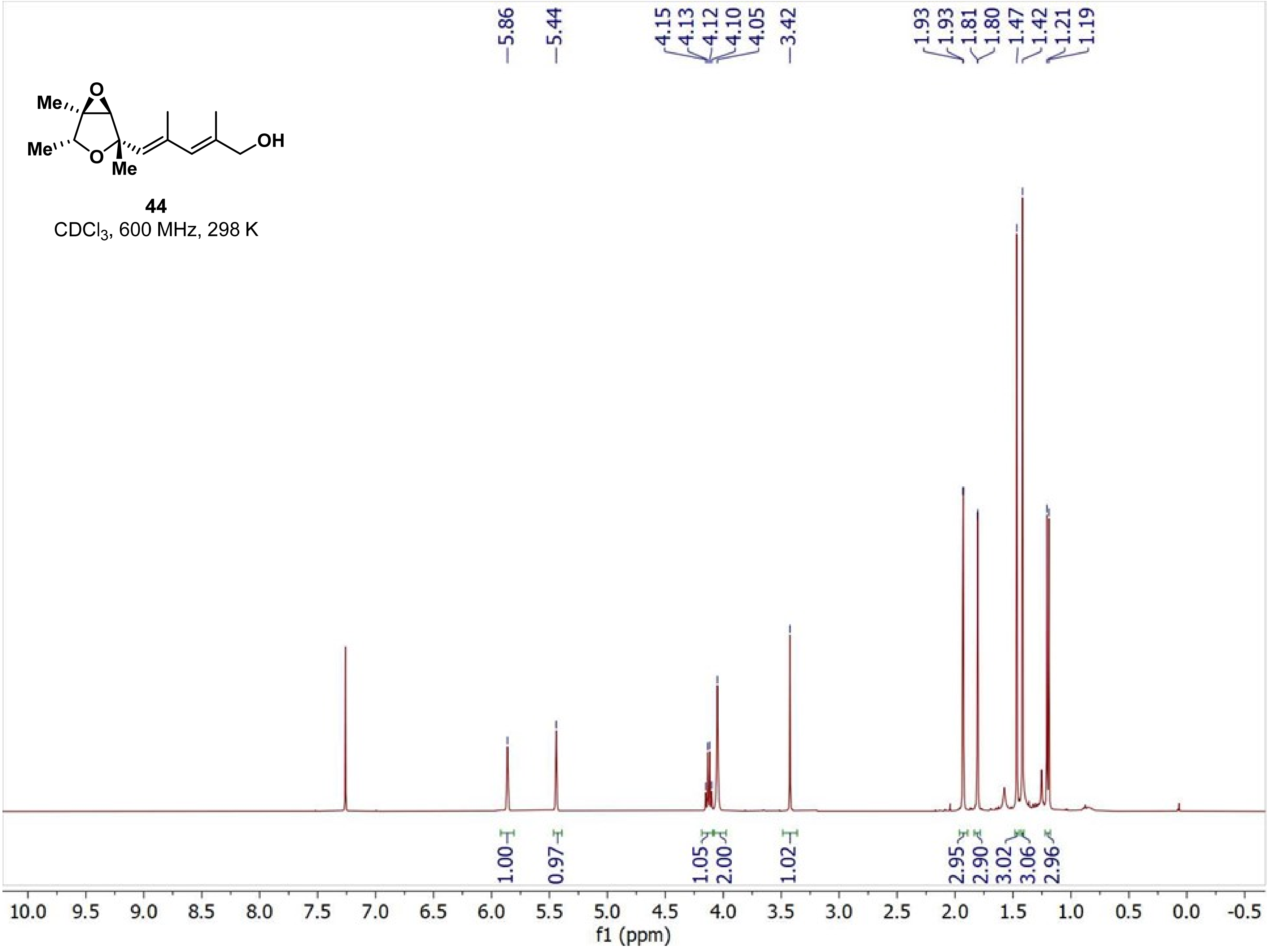

5.86
5.44
4.15
4.13
4.12
4.10
4.05
3.42
1.93
1.93
1.81
1.80
1.47
1.42
1.21
1.19
Me
O
Me
O
Me
OH
44
CDCl3, 600 MHz, 298 K
1.00
0.97
1.05
2.00
1.02
2.95
2.90
3.02
3.06
2.96
10.0
9.5
9.0
8.5
8.0
7.5
7.0
6.5
6.0
5.5
5.0
4.5
4.0
3.5
3.0
2.5
2.0
1.5
1.0
0.5
0.0
-0.5
f1 (ppm)

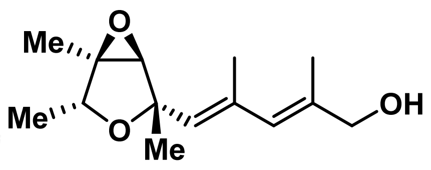

44

$CDCl_3$, 151 MHz, 298 K

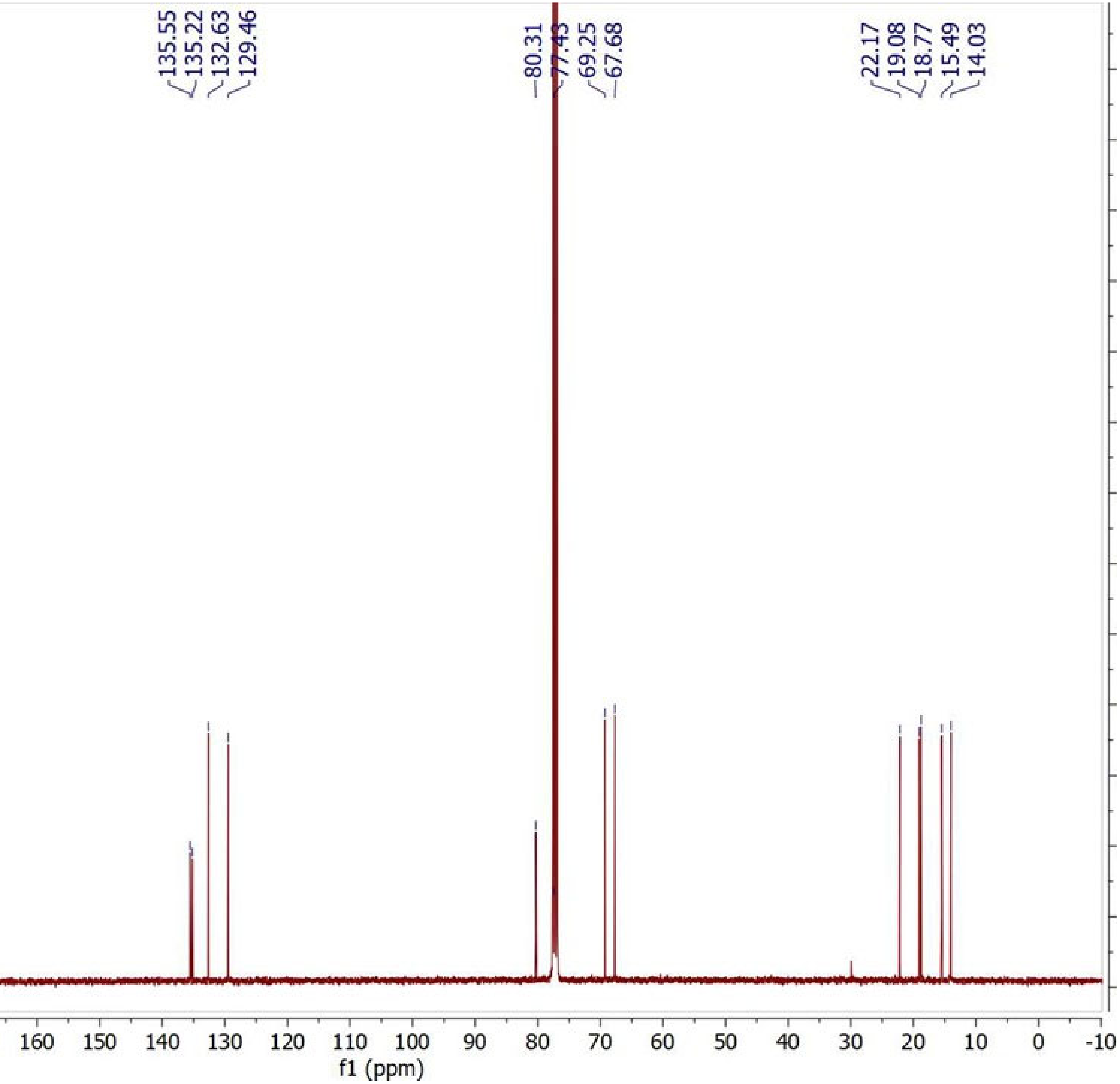

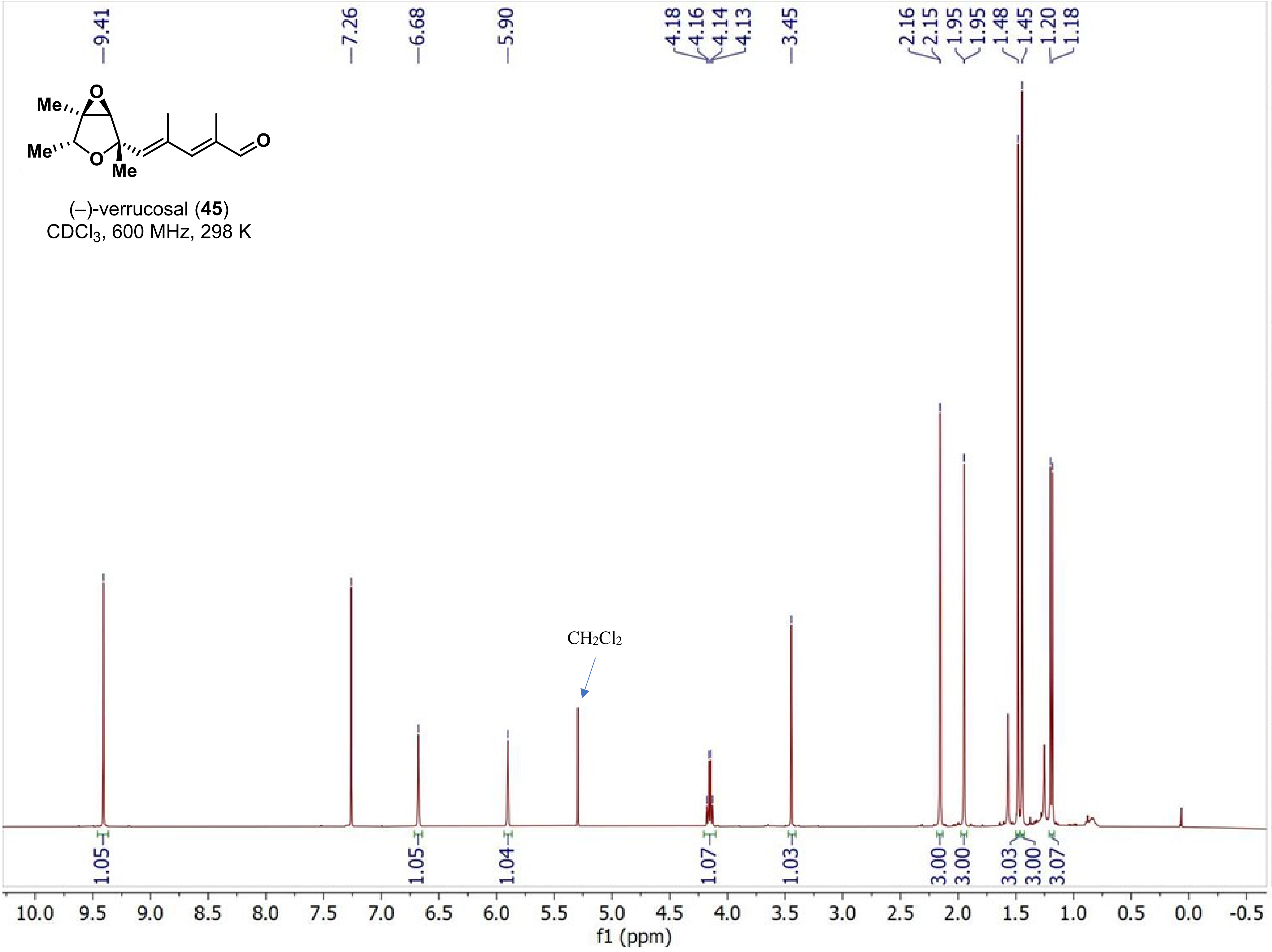

Me
Me
O
O
Me
O
(–)-verrucosal (45)
CDCl3, 600 MHz, 298 K
9.41
7.26
6.68
5.90
4.18
4.16
4.14
4.13
3.45
2.16
2.15
1.95
1.95
1.48
1.45
1.20
1.18
CH2Cl2
1.05
1.05
1.04
1.07
1.03
3.00
3.00
3.03
3.00
3.07
10.0
9.5
9.0
8.5
8.0
7.5
7.0
6.5
6.0
5.5
5.0
4.5
4.0
3.5
3.0
2.5
2.0
1.5
1.0
0.5
0.0
-0.5
f1 (ppm)

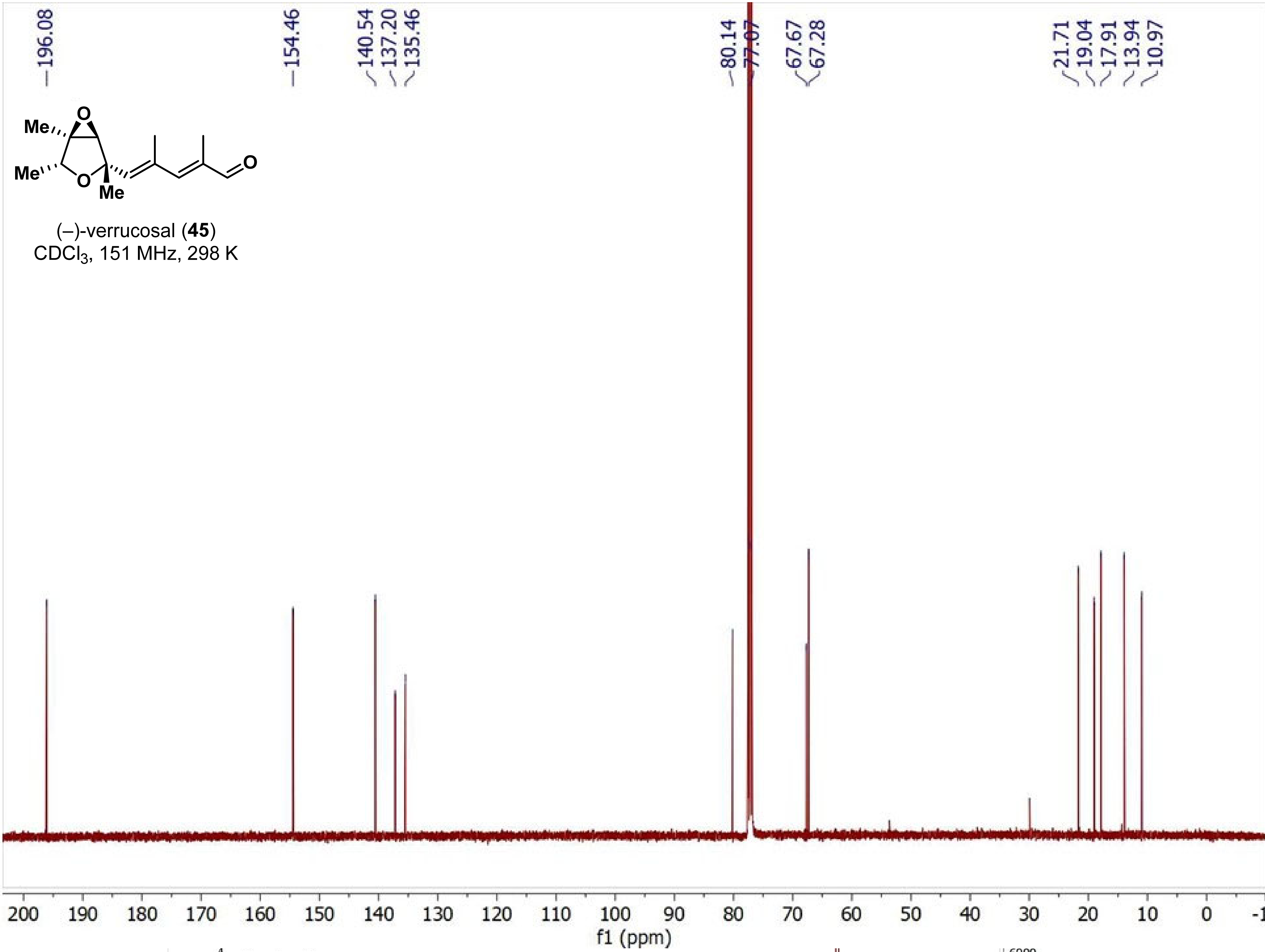

196.08
154.46
140.54
137.20
135.46
80.14
77.07
67.67
67.28
21.71
19.04
17.91
13.94
10.97
Me
Me
O
O
Me
O
(–)-verrucosal (45)
CDCl3, 151 MHz, 298 K
200
190
180
170
160
150
140
130
120
110
100
90
80
70
60
50
40
30
20
10
0
f1 (ppm)

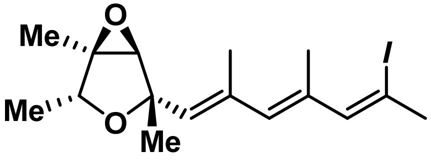

12
$CDCl_3$, 600 MHz, 298 K

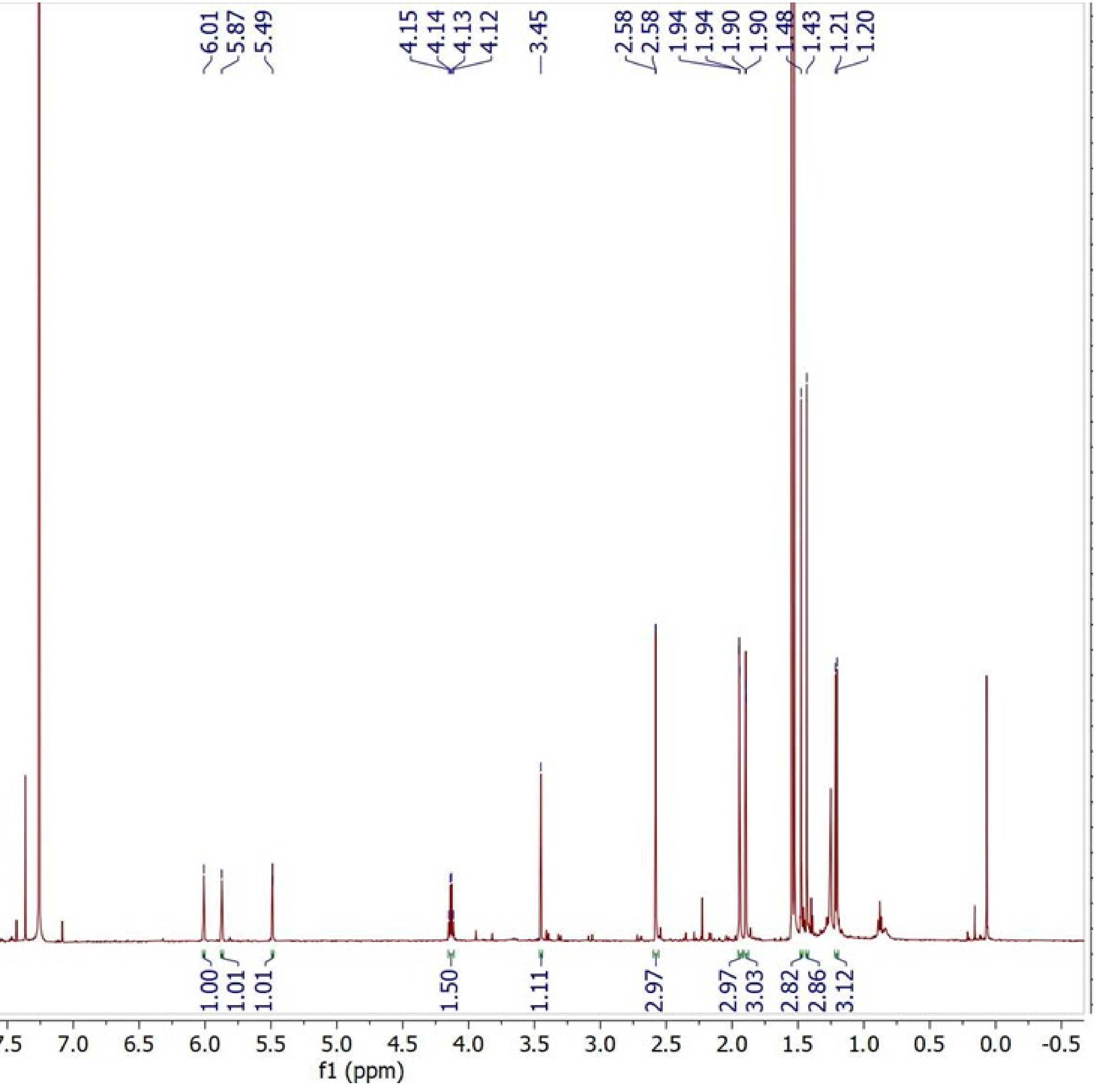

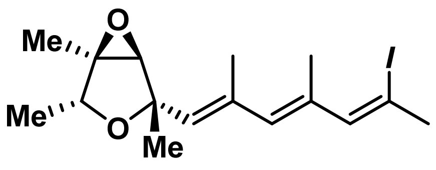

12

$CDCl_3$, 151 MHz, 298 K

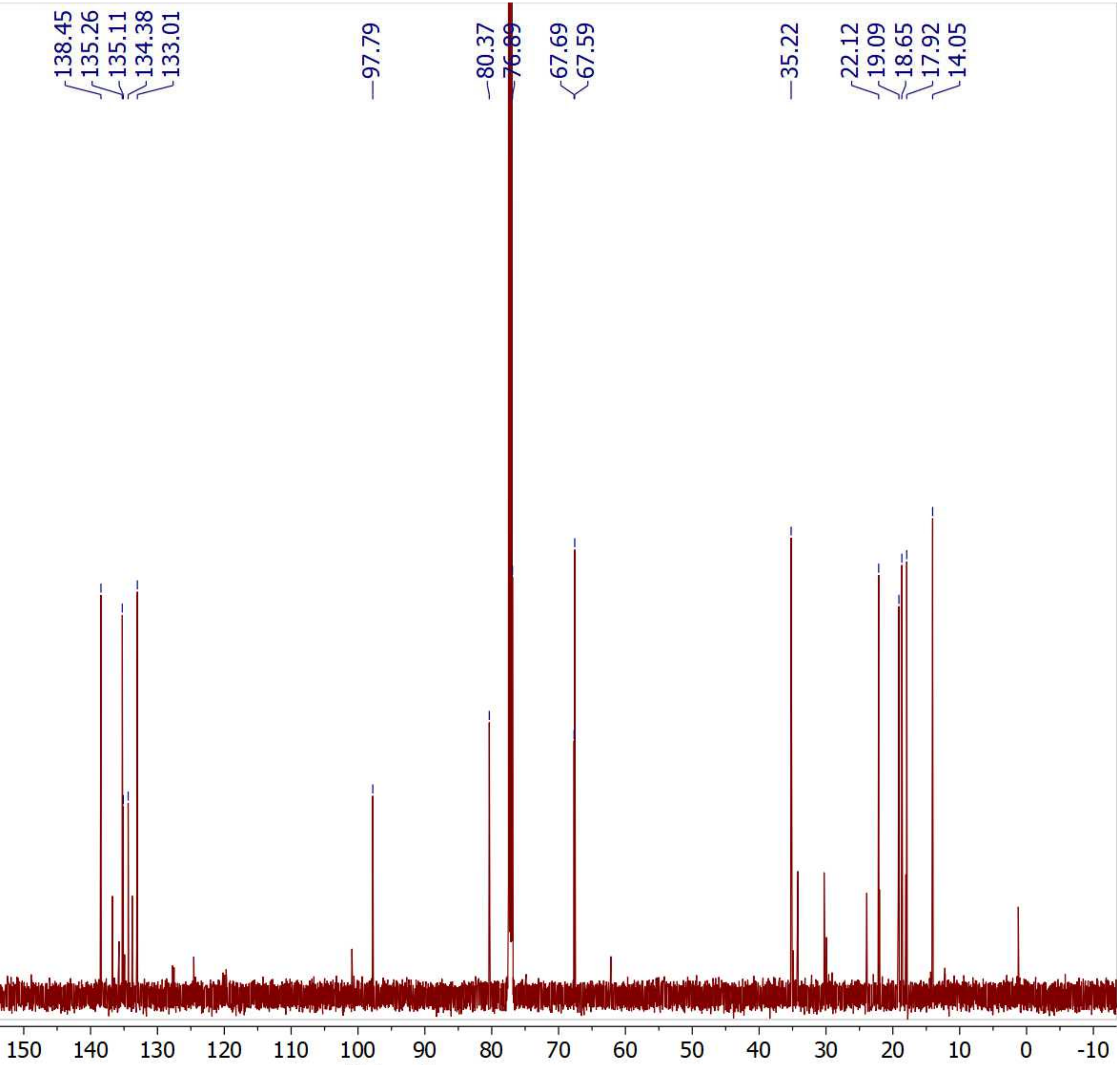

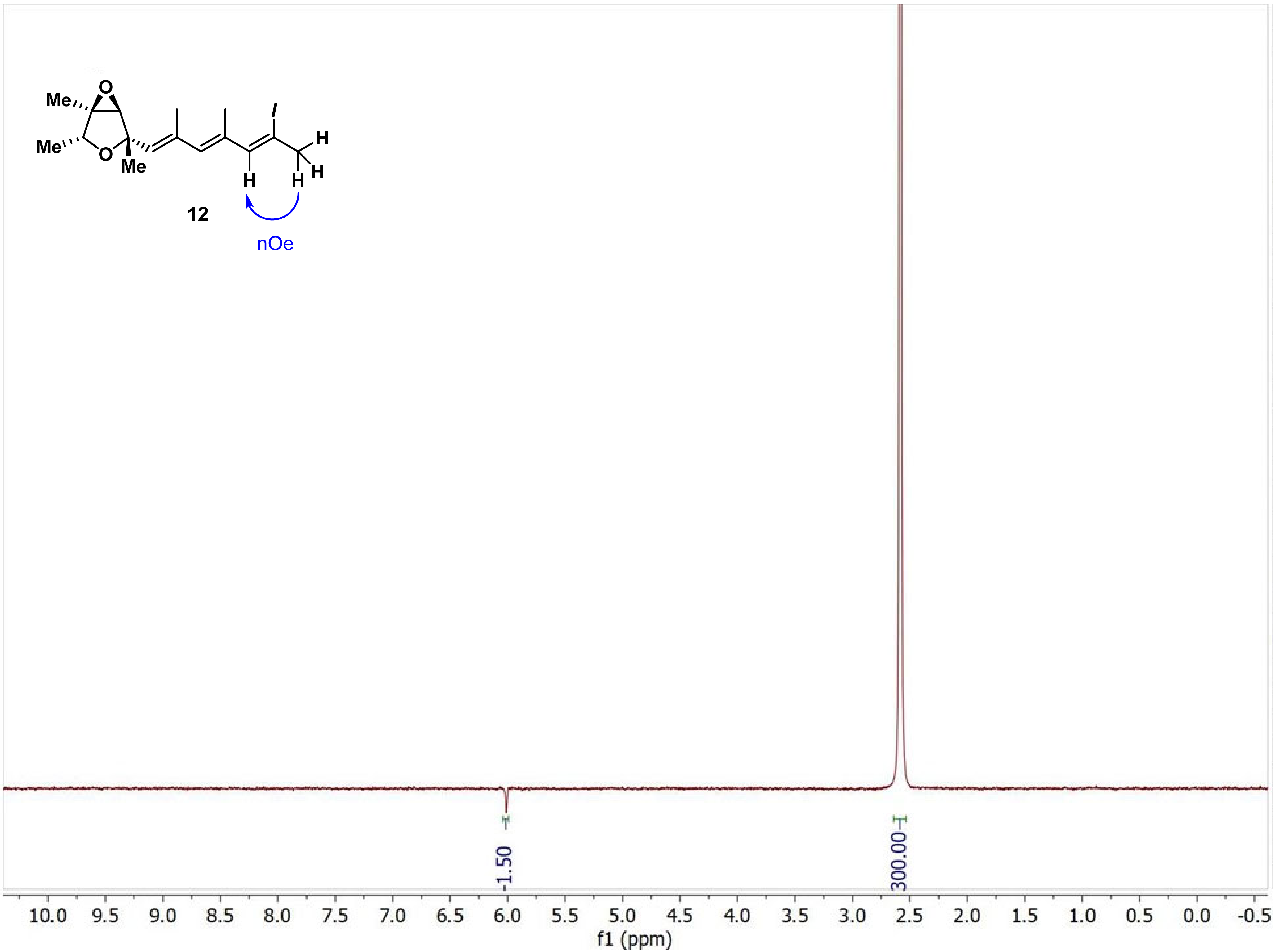
Me
O
Me
O
Me
I
H
H
H
H
12
nOe
-1.50
300.00
10.0 9.5 9.0 8.5 8.0 7.5 7.0 6.5 6.0 5.5 5.0 4.5 4.0 3.5 3.0 2.5 2.0 1.5 1.0 0.5 0.0 -0.5
f1 (ppm)

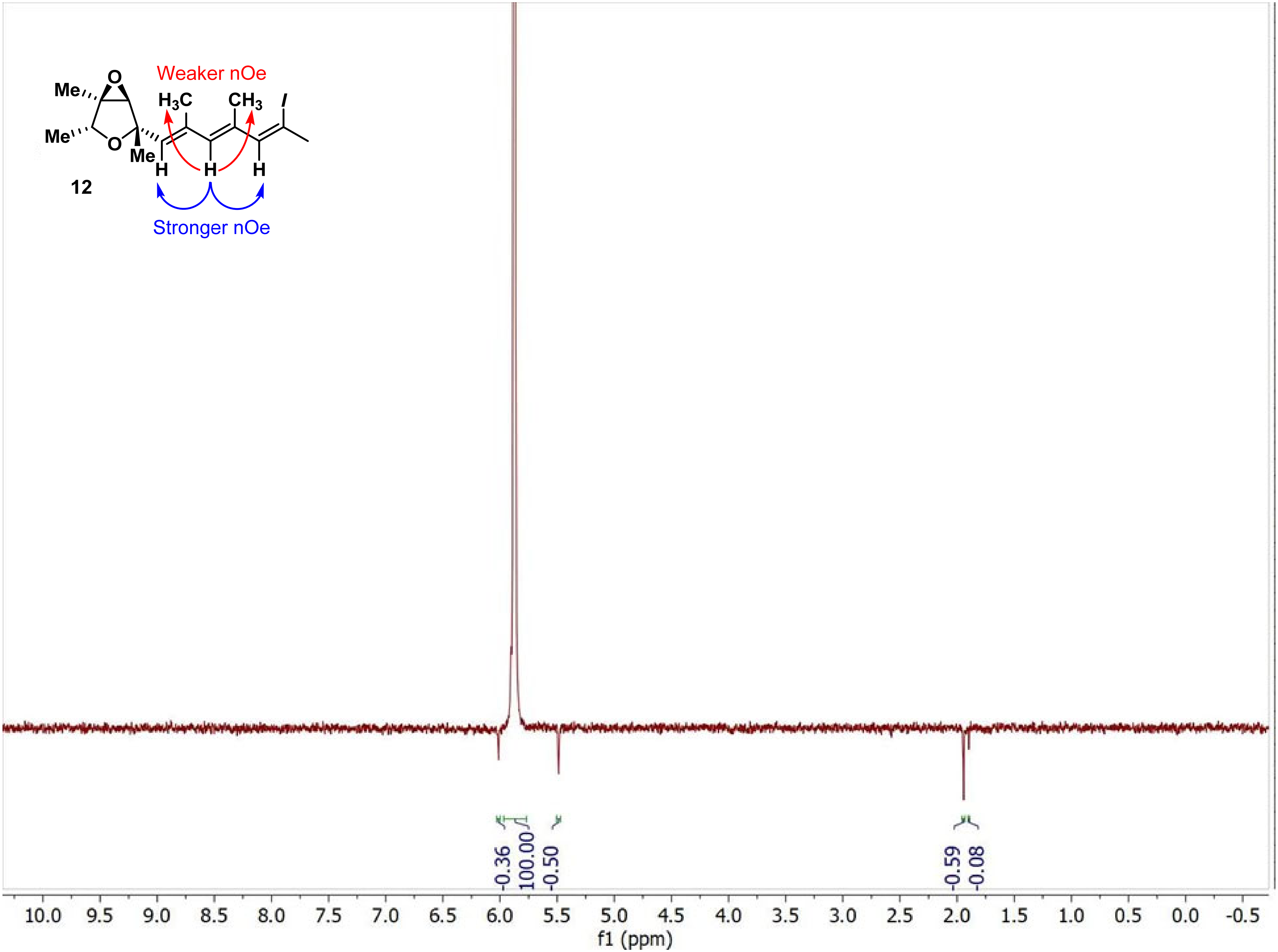

Weaker nOe
Me
O
H3C
CH3
I
Me
O
Me
H
H
H
12
Stronger nOe
-0.36
100.00
-0.50
-0.59
-0.08
10.0
9.5
9.0
8.5
8.0
7.5
7.0
6.5
6.0
5.5
5.0
4.5
4.0
3.5
3.0
2.5
2.0
1.5
1.0
0.5
0.0
-0.5
f1 (ppm)

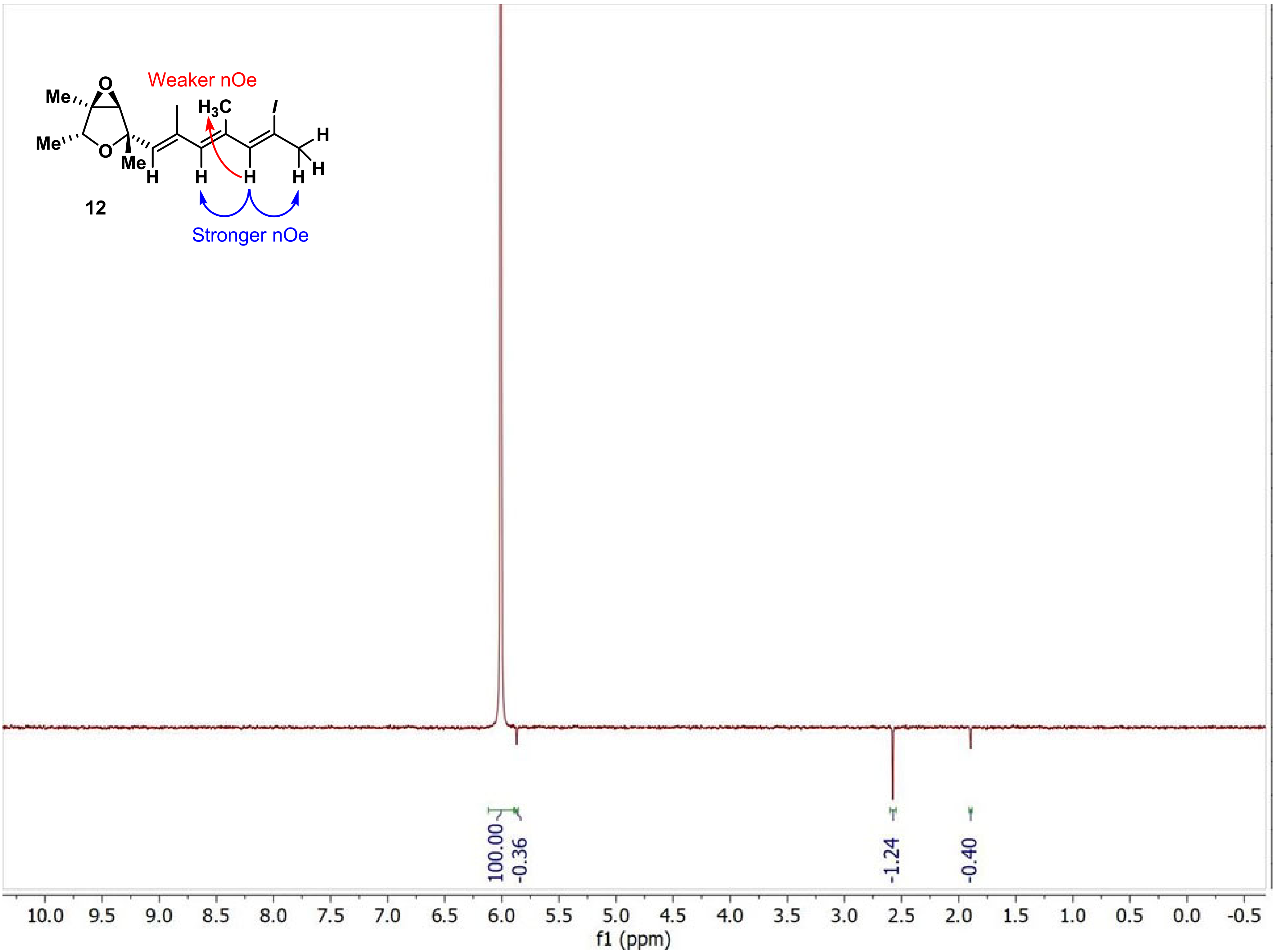

Weaker nOe
Stronger nOe
12
100.00
-0.36
-1.24
-0.40
10.0 9.5 9.0 8.5 8.0 7.5 7.0 6.5 6.0 5.5 5.0 4.5 4.0 3.5 3.0 2.5 2.0 1.5 1.0 0.5 0.0 -0.5
f1 (ppm)

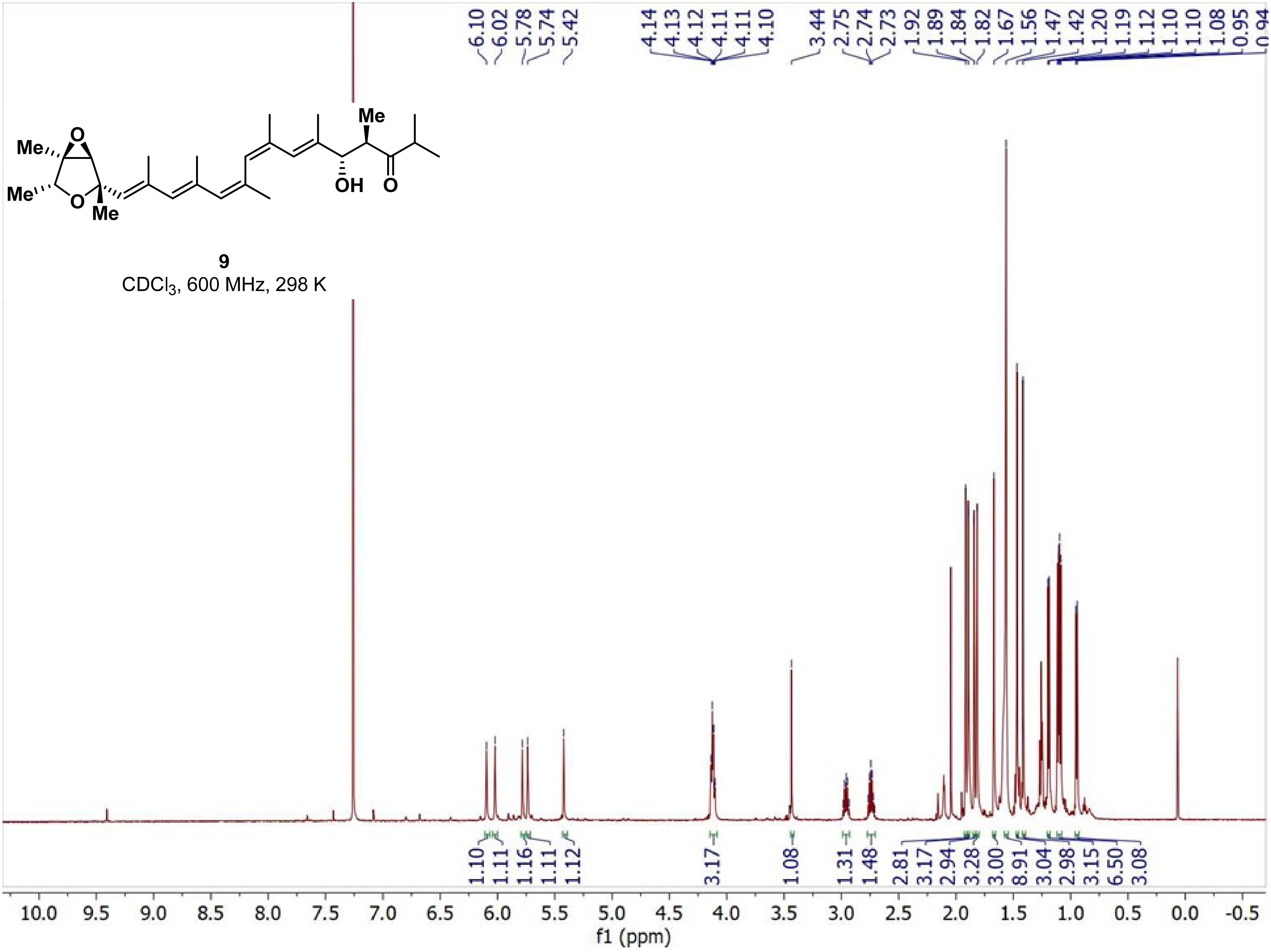
6.10 6.02 5.78 5.74 5.42 4.14 4.13 4.12 4.11 4.11 4.10 3.44 2.75 2.74 2.73 1.92 1.89 1.84 1.82 1.67 1.56 1.47 1.42 1.20 1.19 1.12 1.10 1.10 1.08 0.95 0.94
Me
O
OH
Me
Me
O
Me
9
$CDCl_3$, 600 MHz, 298 K
1.10 1.11 1.16 1.11 1.12 3.17 1.08 1.31 1.48 2.81 3.17 2.94 3.28 3.00 8.91 3.04 2.98 3.15 6.50 3.08
10.0 9.5 9.0 8.5 8.0 7.5 7.0 6.5 6.0 5.5 5.0 4.5 4.0 3.5 3.0 2.5 2.0 1.5 1.0 0.5 0.0 -0.5
f1 (ppm)

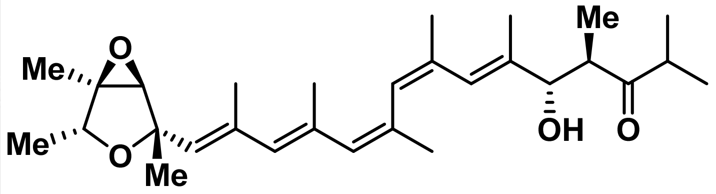

9

$CDCl_3$, 101 MHz, 298 K

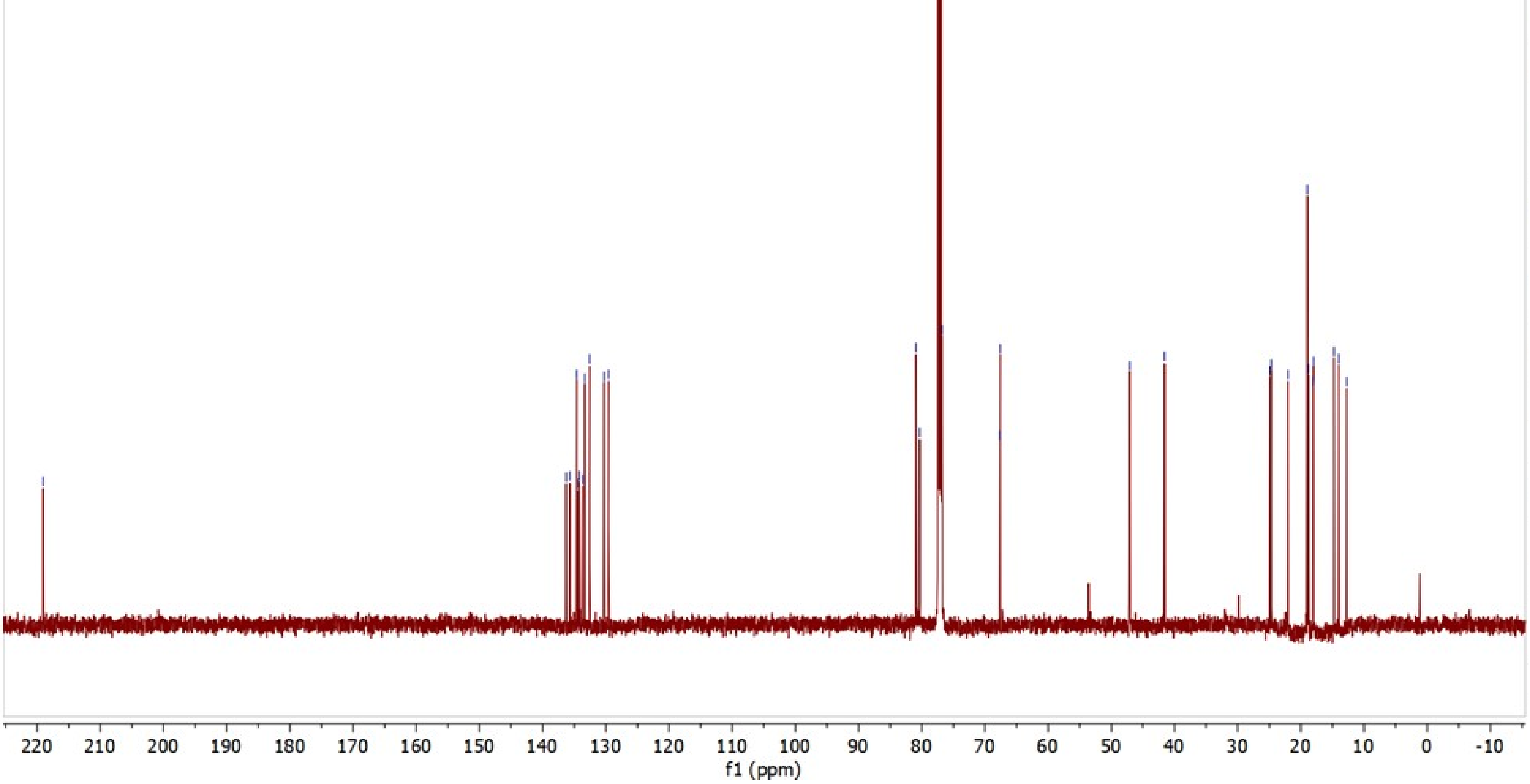

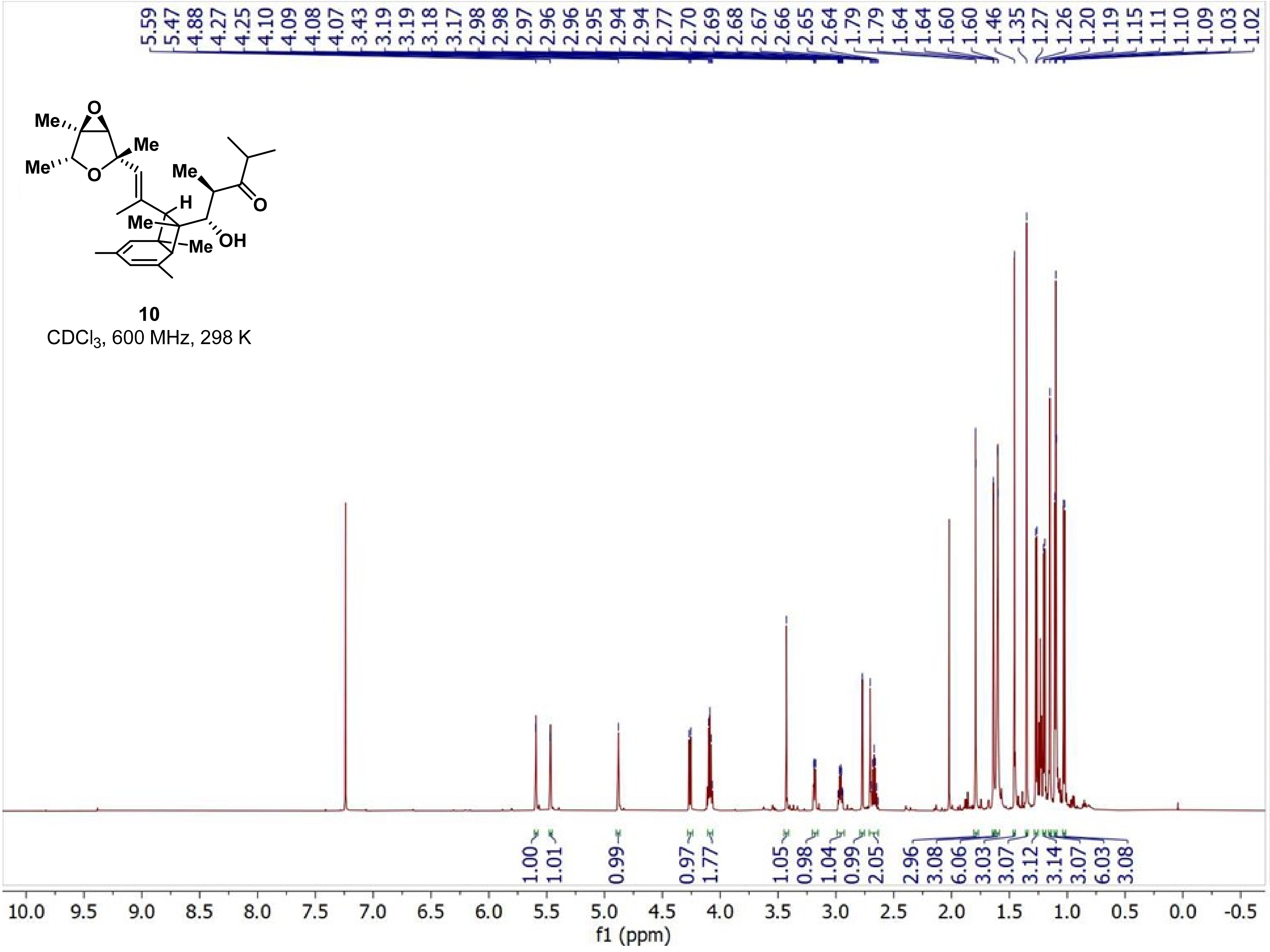
5.59 5.47 4.88 4.27 4.25 4.10 4.09 4.08 4.07 3.43 3.19 3.19 3.18 3.17 2.98 2.98 2.97 2.96 2.96 2.95 2.94 2.94 2.77 2.70 2.69 2.68 2.67 2.66 2.65 2.64 1.79 1.79 1.64 1.64 1.60 1.60 1.46 1.35 1.27 1.26 1.20 1.19 1.15 1.11 1.10 1.09 1.03 1.02
Me
O
Me
Me
O
Me
O
H
Me
Me
OH
10
$CDCl_3$, 600 MHz, 298 K
1.00 1.01 0.99 0.97 1.77 1.05 0.98 1.04 0.99 2.05 2.96 3.08 6.06 3.03 3.07 3.12 3.14 3.07 6.03 3.08
10.0 9.5 9.0 8.5 8.0 7.5 7.0 6.5 6.0 5.5 5.0 4.5 4.0 3.5 3.0 2.5 2.0 1.5 1.0 0.5 0.0 -0.5
f1 (ppm)

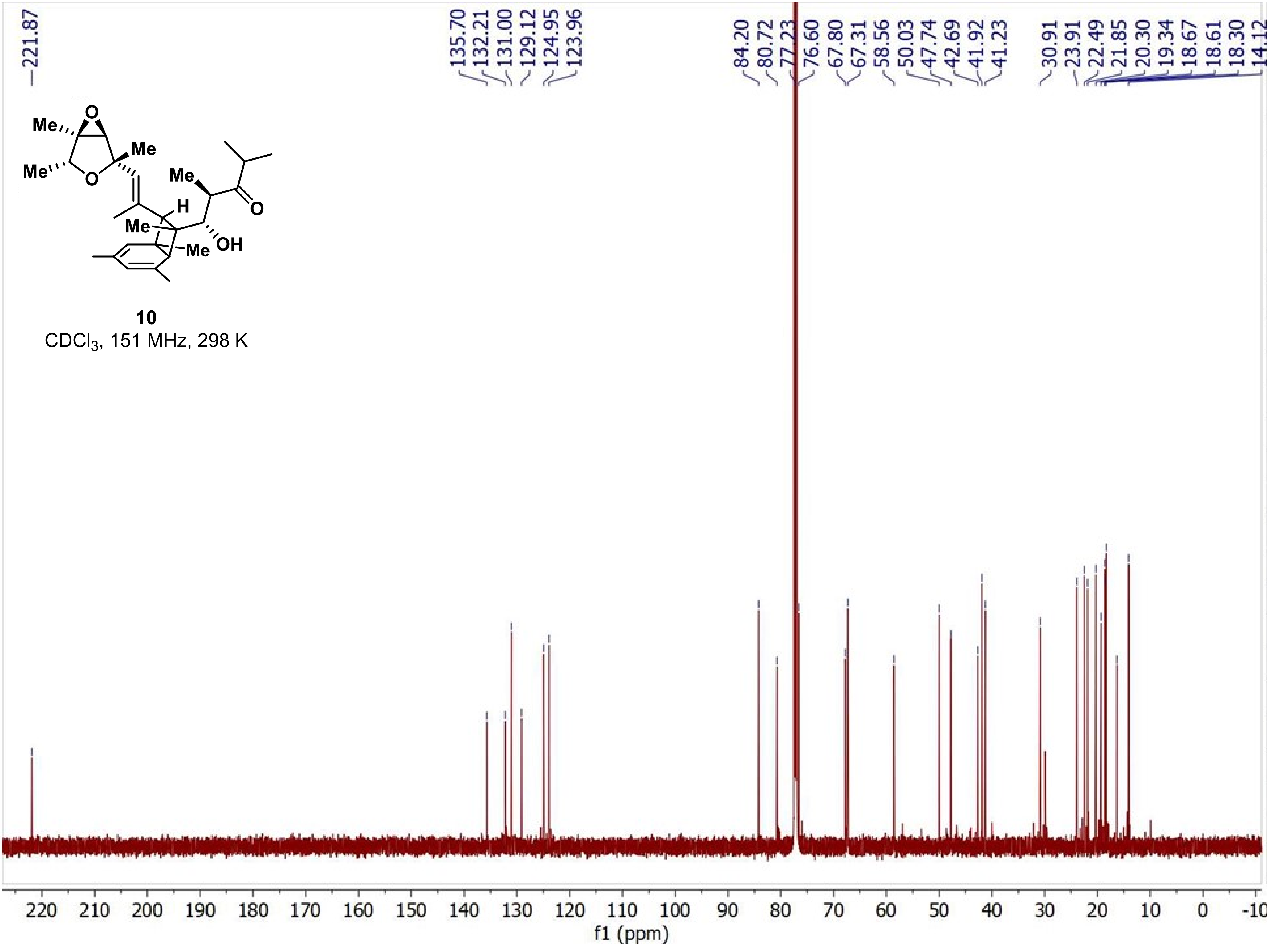

221.87
135.70
132.21
131.00
129.12
124.95
123.96
84.20
80.72
77.23
76.60
67.80
67.31
58.56
50.03
47.74
42.69
41.92
41.23
30.91
23.91
22.49
21.85
20.30
19.34
18.67
18.61
18.30
14.12
Me
Me
Me
Me
Me
Me
O
O
O
H
OH
10
$CDCl_3$, 151 MHz, 298 K
220 210 200 190 180 170 160 150 140 130 120 110 100 90 80 70 60 50 40 30 20 10 0 -10
f1 (ppm)

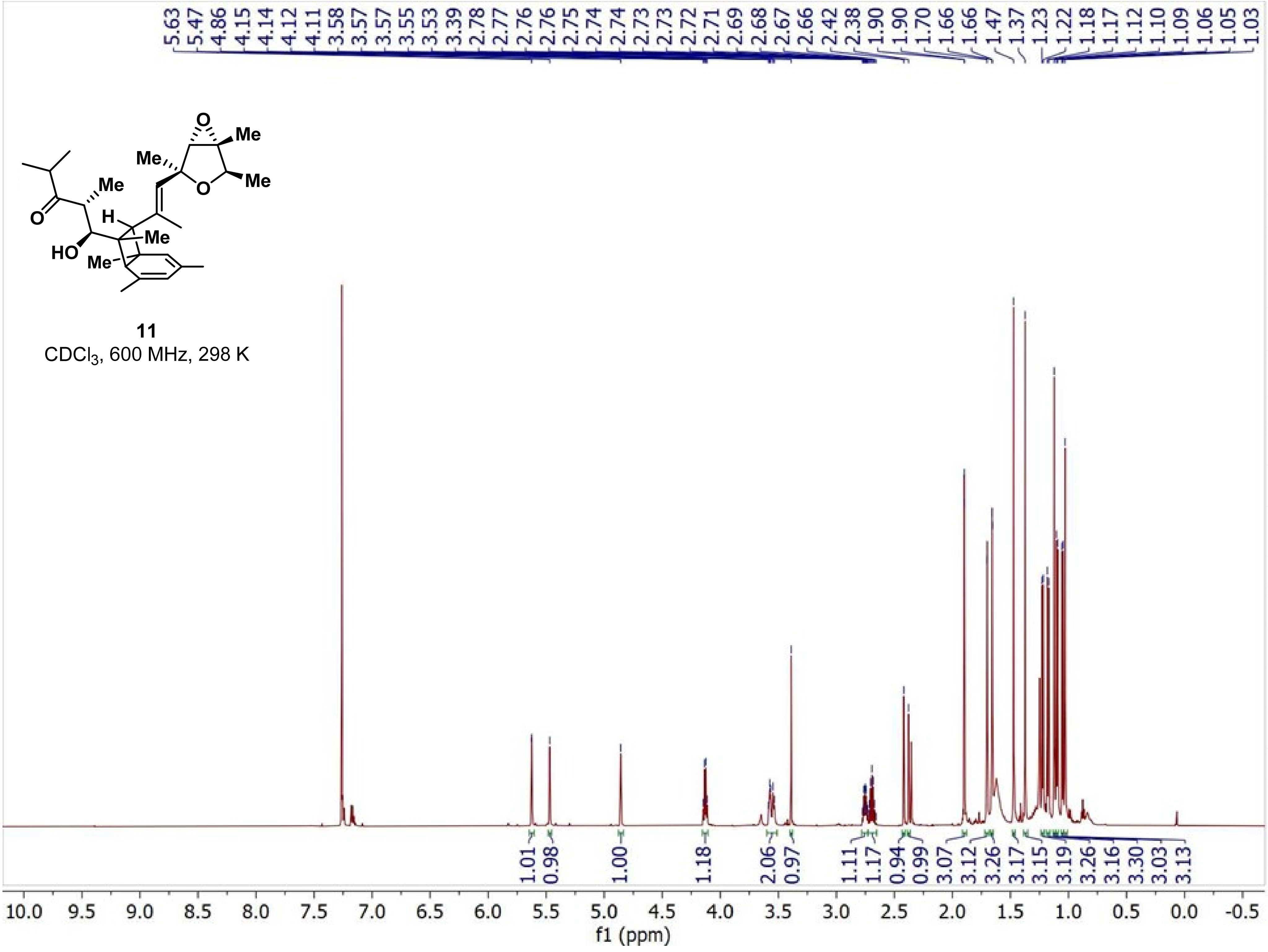

Me
Me
Me
Me
Me
O
O
O
H
HO
Me
Me
11
CDCl3, 600 MHz, 298 K
5.63 5.47 4.86 4.15 4.14 4.12 4.11 3.58 3.57 3.57 3.55 3.53 3.39 2.78 2.77 2.76 2.76 2.75 2.74 2.74 2.73 2.73 2.72 2.71 2.69 2.68 2.67 2.66 2.42 2.38 1.90 1.90 1.70 1.66 1.66 1.47 1.37 1.23 1.22 1.18 1.17 1.12 1.10 1.09 1.06 1.05 1.03
1.01 0.98 1.00 1.18 2.06 0.97 1.11 1.17 0.94 0.99 3.07 3.12 3.26 3.17 3.15 3.19 3.26 3.16 3.30 3.03 3.13
10.0 9.5 9.0 8.5 8.0 7.5 7.0 6.5 6.0 5.5 5.0 4.5 4.0 3.5 3.0 2.5 2.0 1.5 1.0 0.5 0.0 -0.5
f1 (ppm)

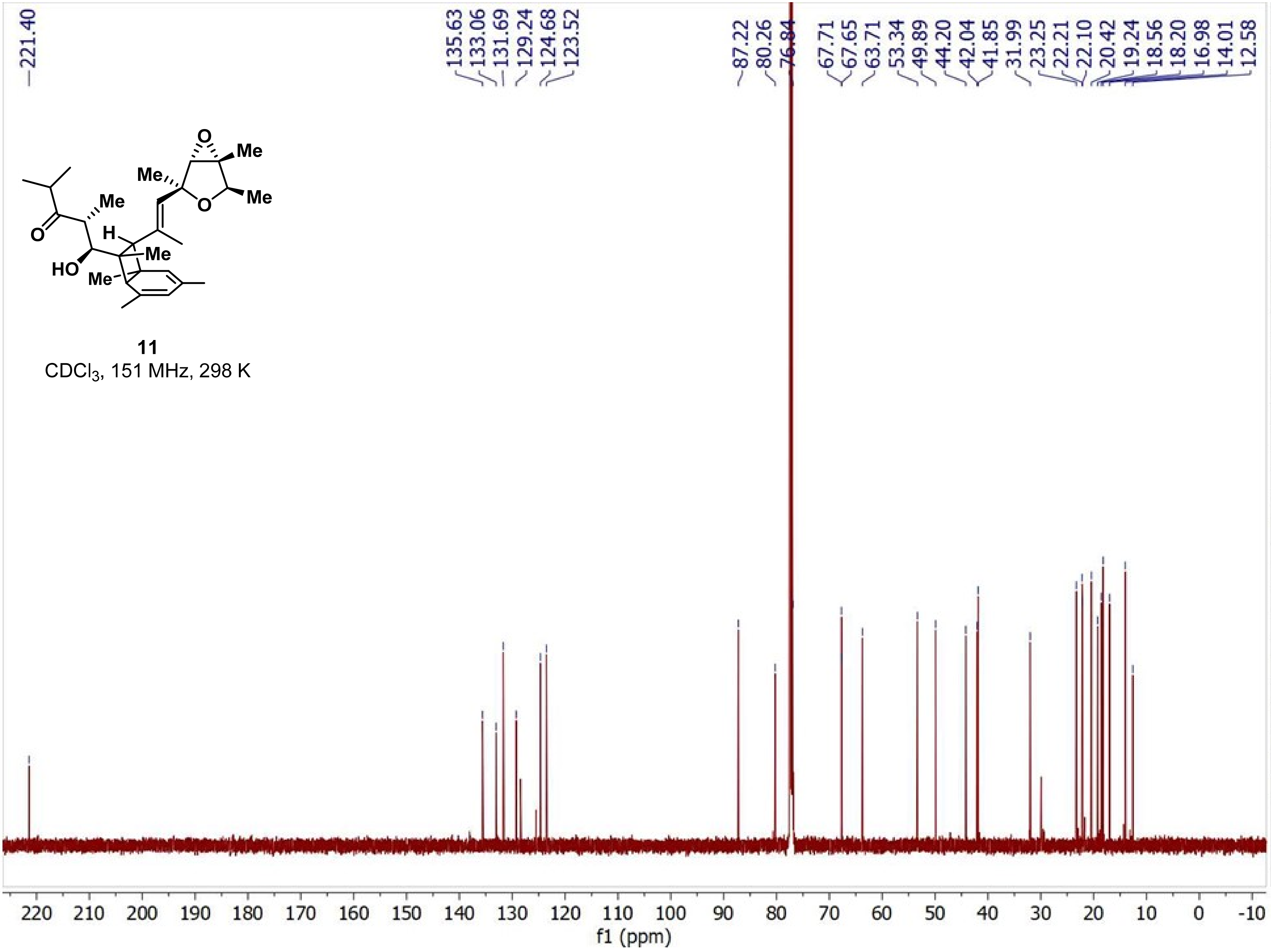

11
$CDCl_3$, 151 MHz, 298 K

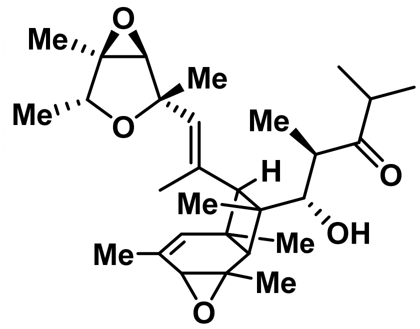

(–)-emerione A (**1**)
DMSO, 400 MHz, 298 K

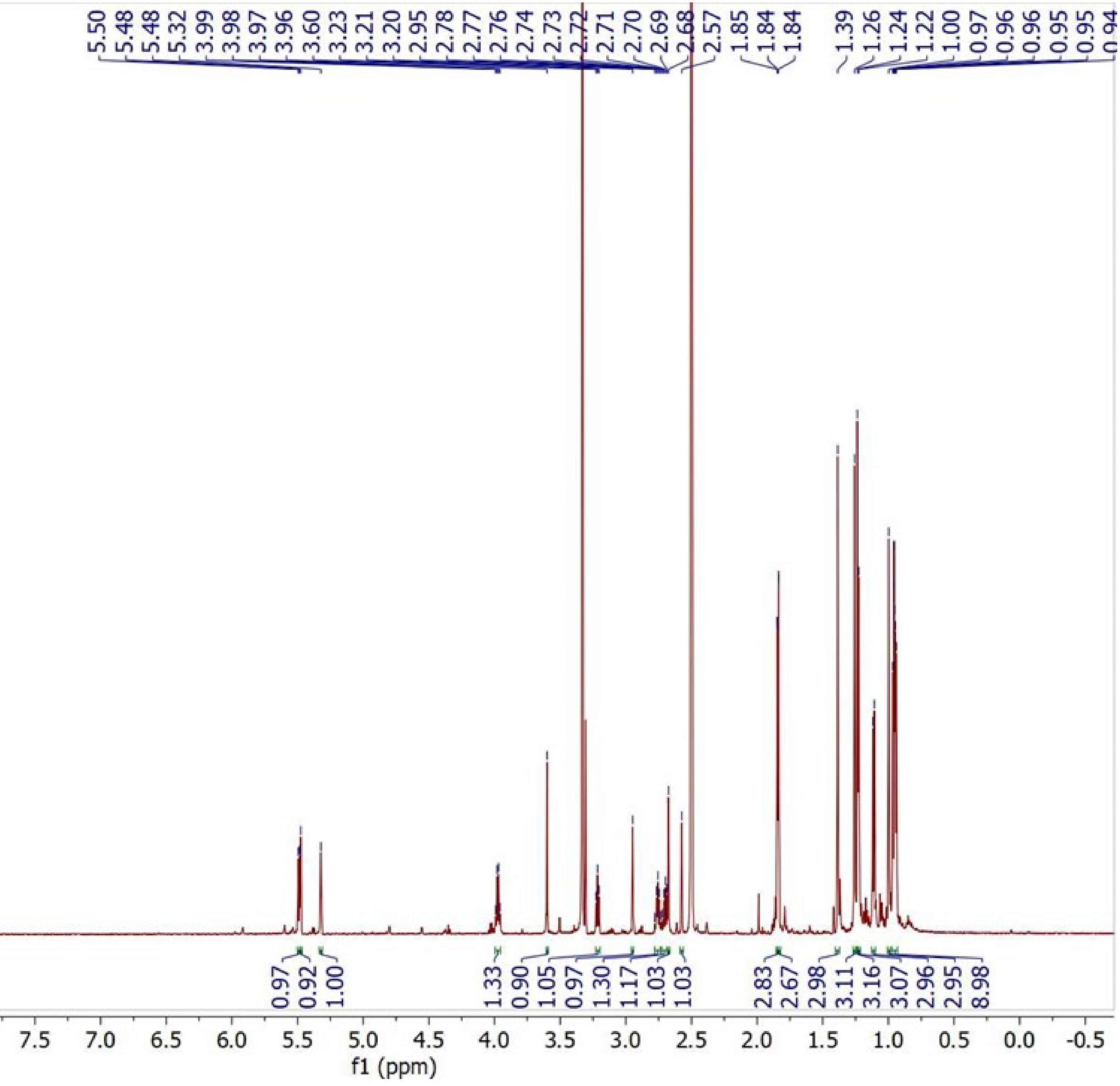

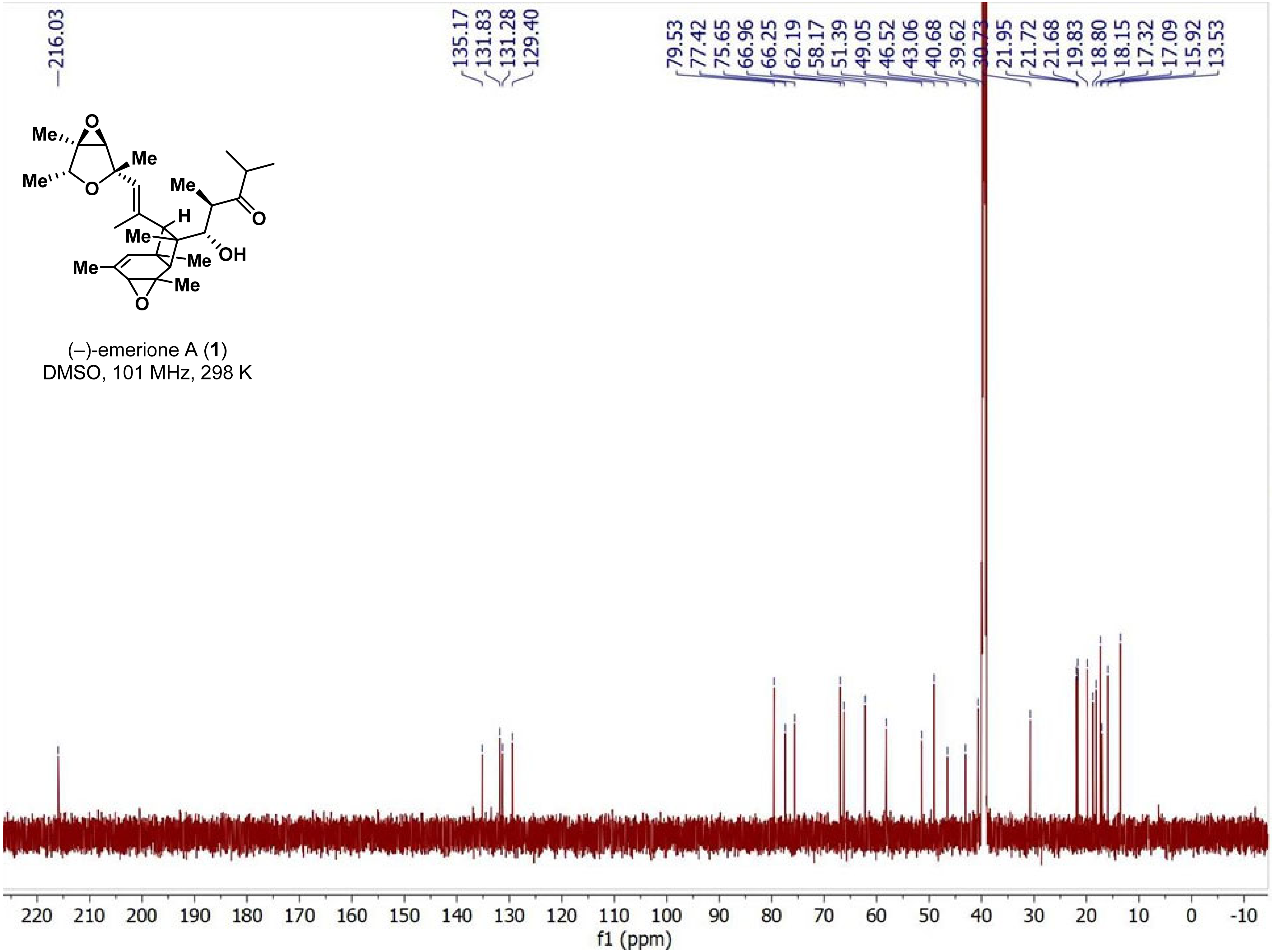

(–)-emerione A (**1**)
DMSO, 101 MHz, 298 K

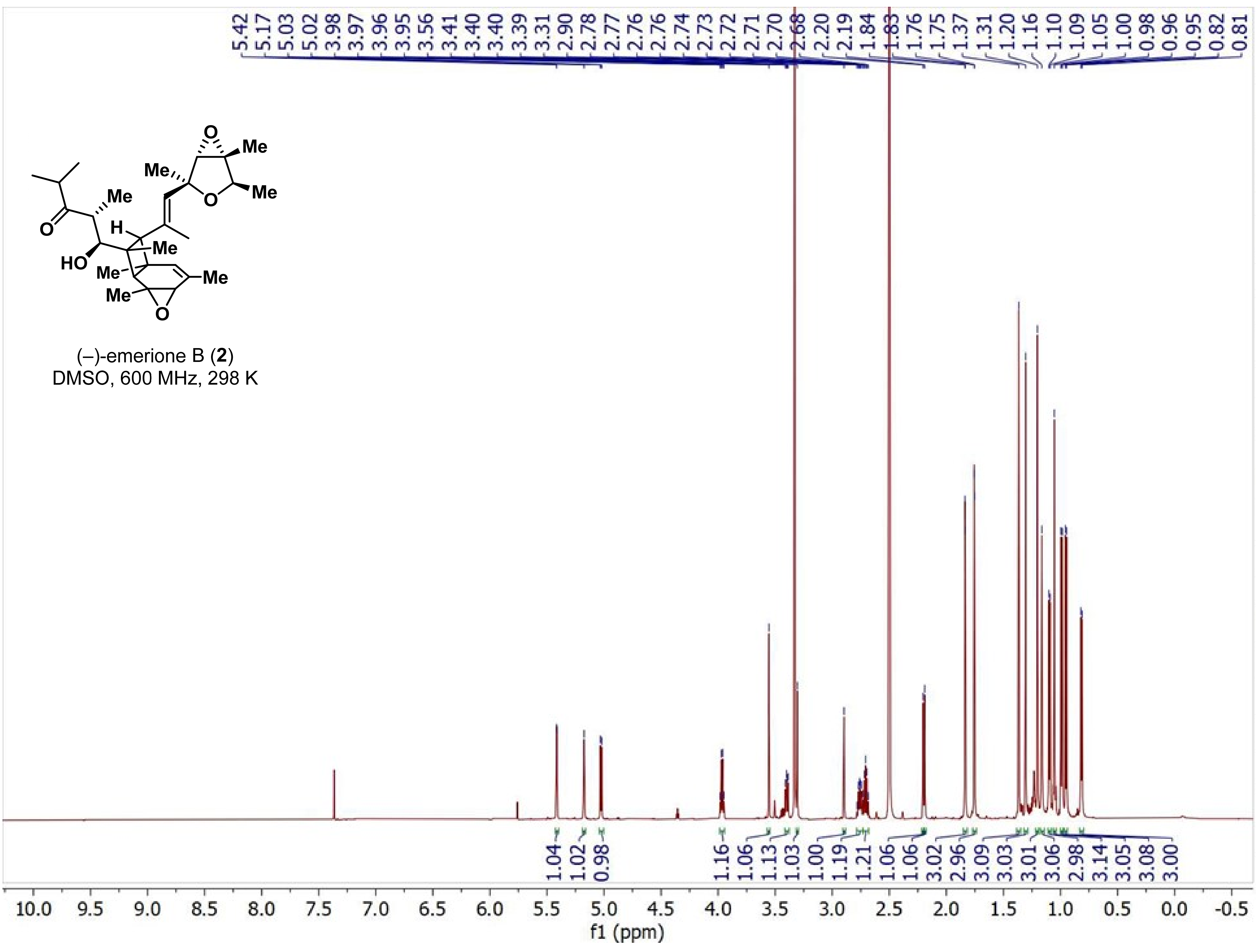

(–)-emerione B (2)
DMSO, 600 MHz, 298 K
Me
Me
Me
Me
Me
Me
Me
H
HO
O
O
O
5.42 5.17 5.03 5.02 3.98 3.97 3.96 3.95 3.56 3.41 3.40 3.40 3.39 3.31 2.90 2.78 2.77 2.76 2.76 2.74 2.73 2.72 2.71 2.70 2.68 2.20 2.19 1.84 1.83 1.76 1.75 1.37 1.31 1.20 1.16 1.10 1.09 1.05 1.00 0.98 0.96 0.95 0.82 0.81
1.04 1.02 0.98 1.16 1.06 1.13 1.03 1.00 1.19 1.21 1.06 1.06 3.02 2.96 3.09 3.03 3.01 3.06 2.98 3.14 3.05 3.08 3.00
10.0 9.5 9.0 8.5 8.0 7.5 7.0 6.5 6.0 5.5 5.0 4.5 4.0 3.5 3.0 2.5 2.0 1.5 1.0 0.5 0.0 -0.5
f1 (ppm)

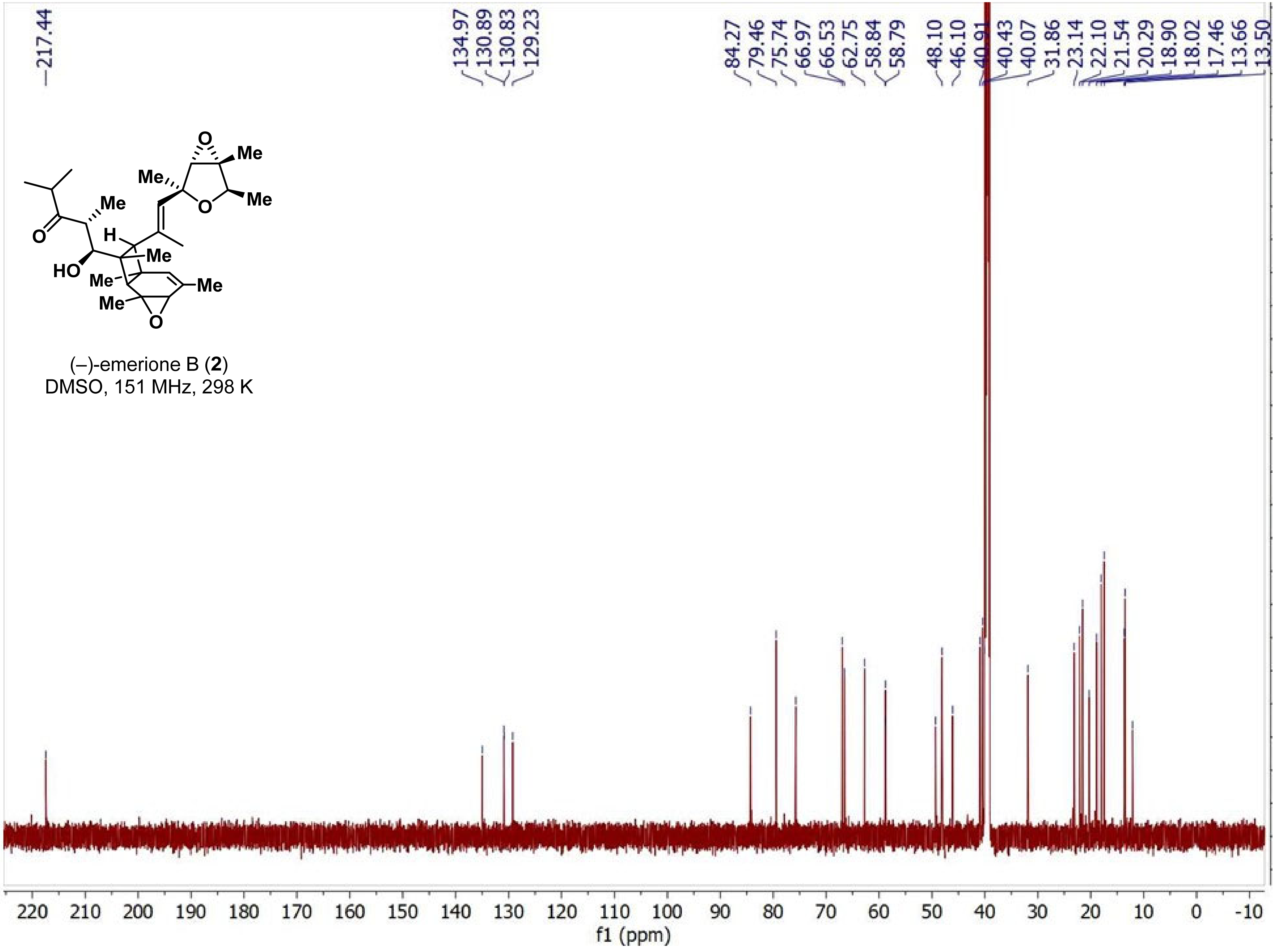

(–)-emerione B (**2**)
DMSO, 151 MHz, 298 K

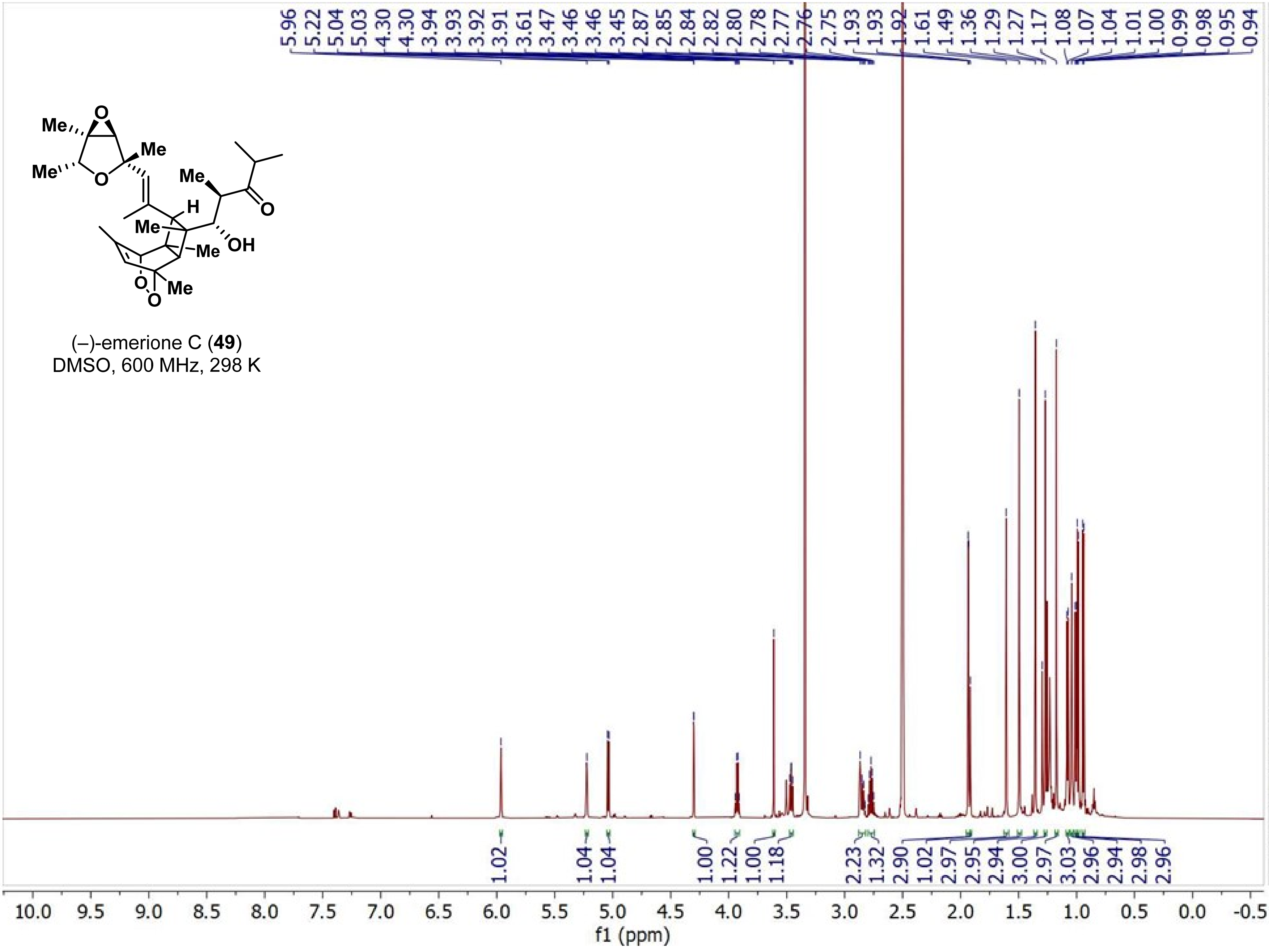

(–)-emerione C (**49**)
DMSO, 600 MHz, 298 K

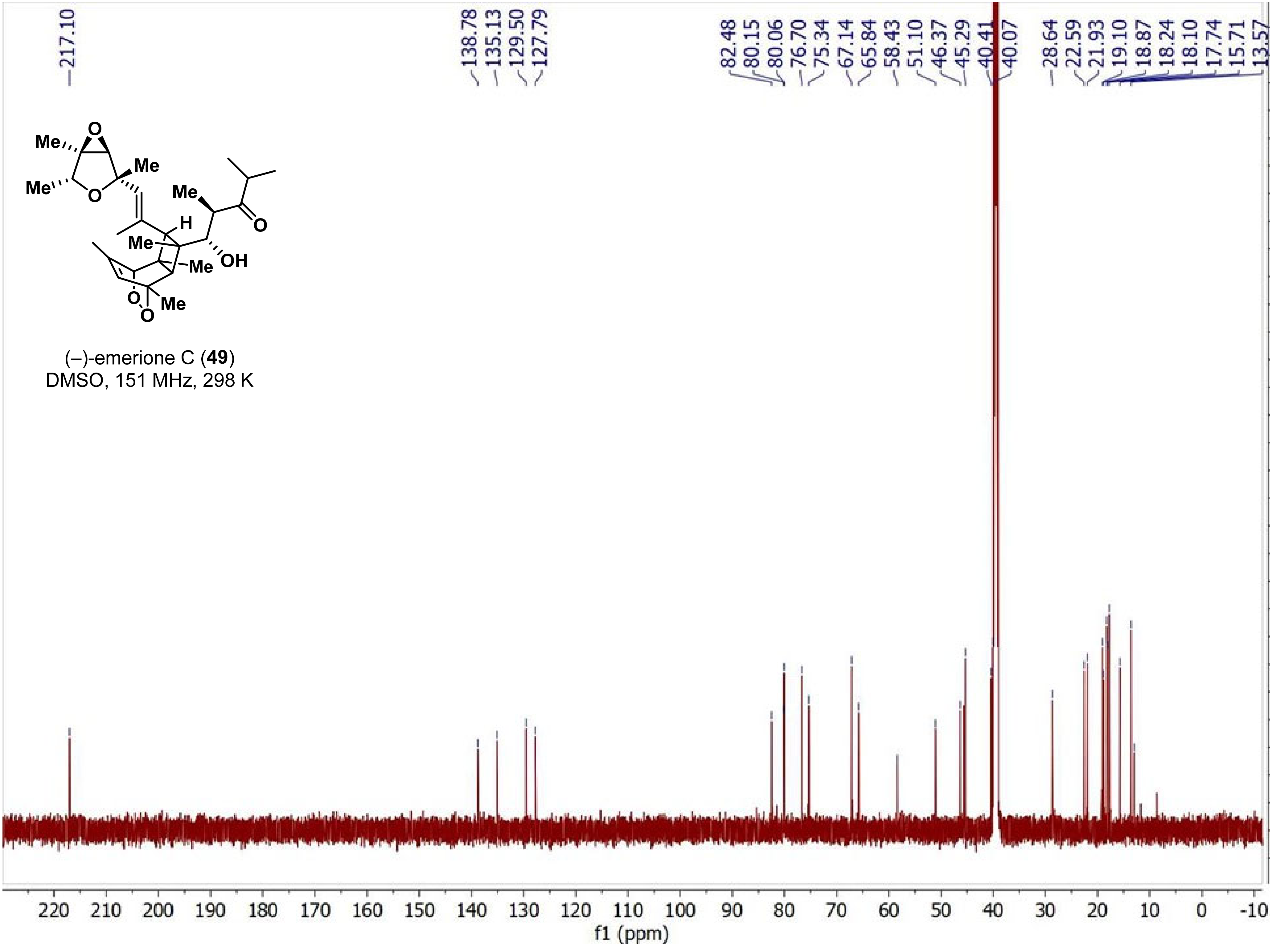
217.10
138.78
135.13
129.50
127.79
82.48
80.15
80.06
76.70
75.34
67.14
65.84
58.43
51.10
46.37
45.29
40.41
40.07
28.64
22.59
21.93
19.10
18.87
18.24
18.10
17.74
15.71
13.57
Me
O
Me
Me
O
Me
H
O
Me
Me
OH
O
O
Me
(–)-emerione C (49)
DMSO, 151 MHz, 298 K
220 210 200 190 180 170 160 150 140 130 120 110 100 90 80 70 60 50 40 30 20 10 0 -10
f1 (ppm)

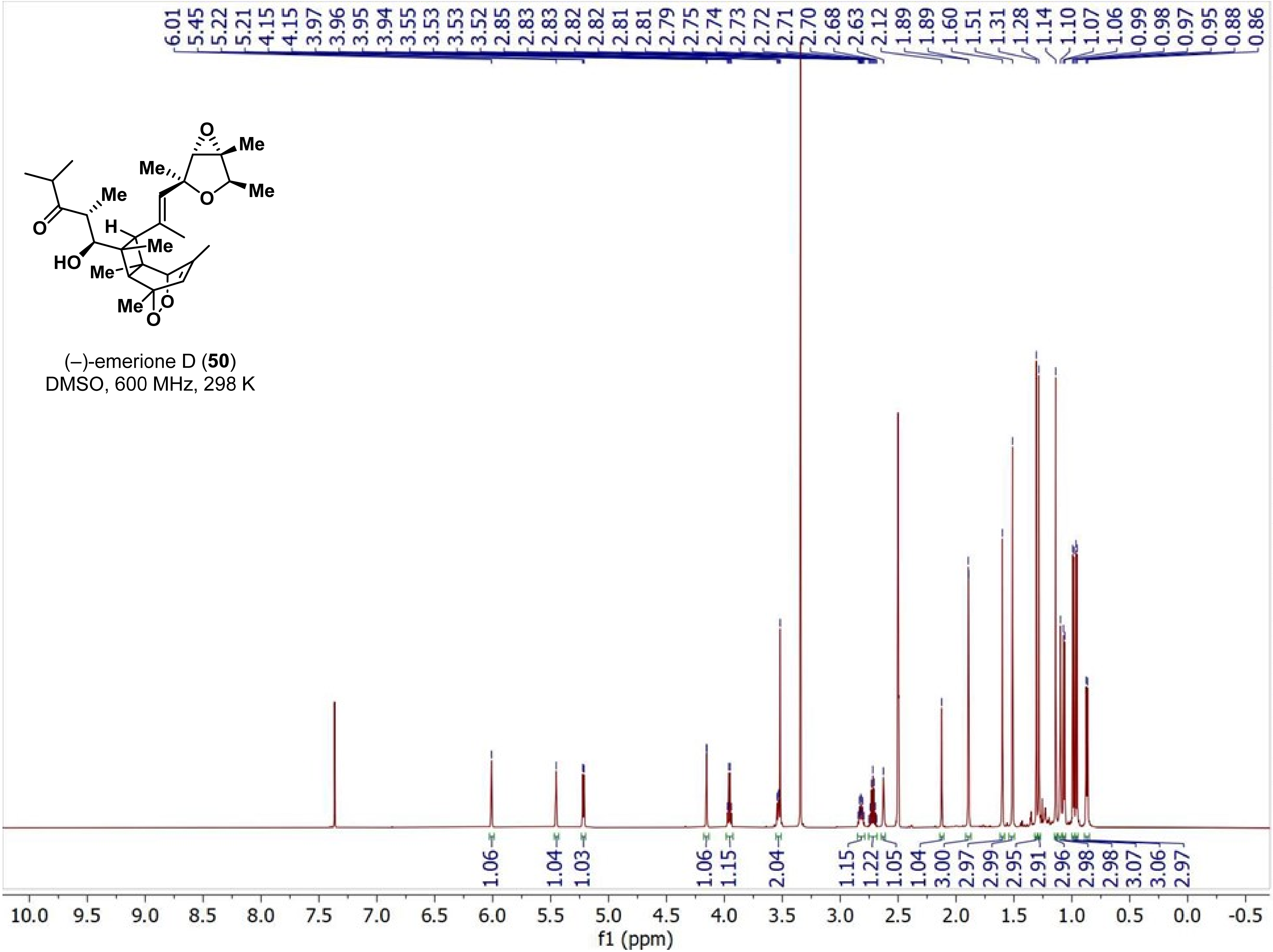

(–)-emerione D (**50**)
DMSO, 600 MHz, 298 K

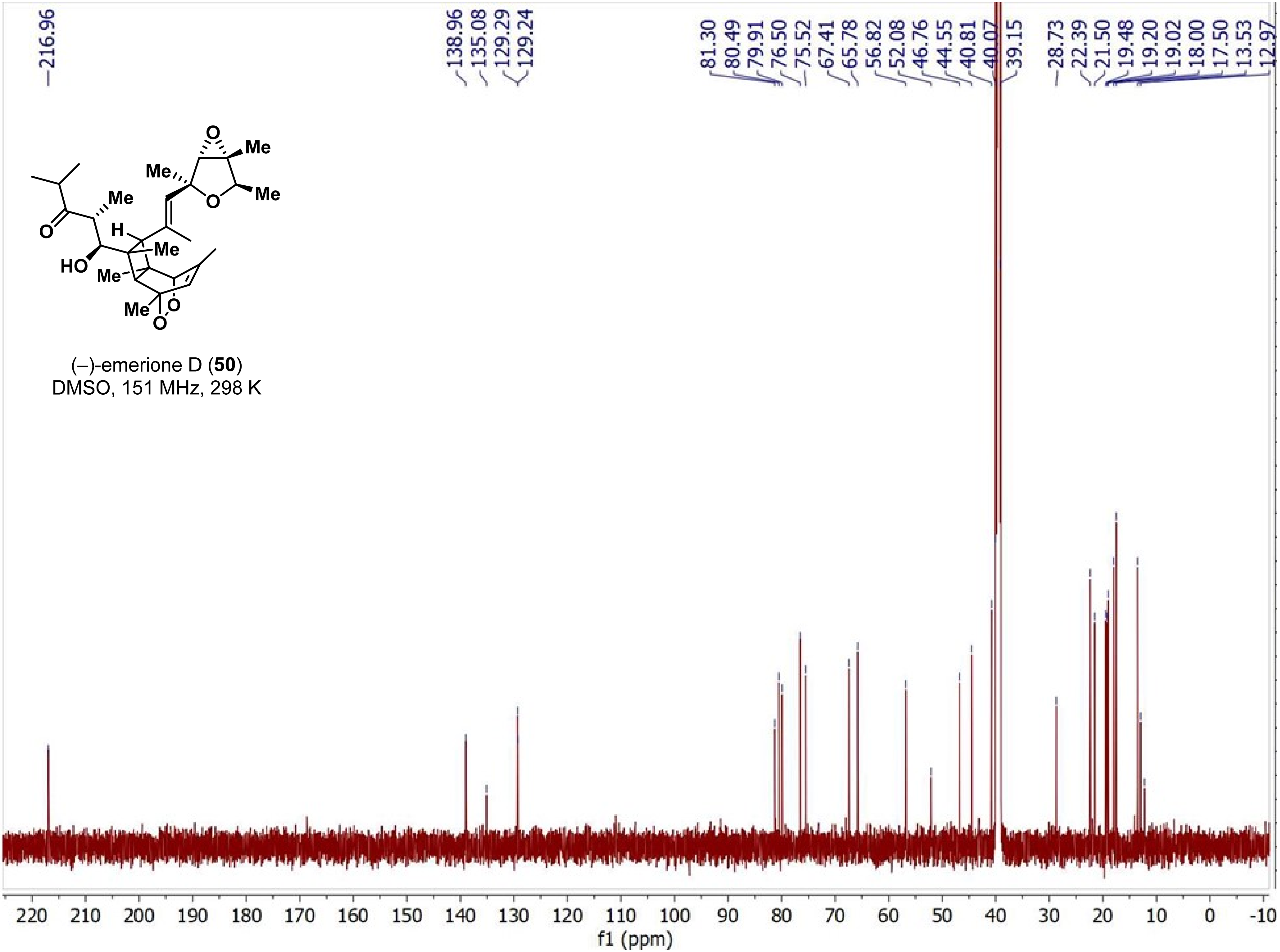

216.96
138.96
135.08
129.29
129.24
81.30
80.49
79.91
76.50
75.52
67.41
65.78
56.82
52.08
46.76
44.55
40.81
40.07
39.15
28.73
22.39
21.50
19.48
19.20
19.02
18.00
17.50
13.53
12.97
(–)-emerione D (**50**)
DMSO, 151 MHz, 298 K
220 210 200 190 180 170 160 150 140 130 120 110 100 90 80 70 60 50 40 30 20 10 0 -10
f1 (ppm)
